# β-Silyl alkynoates: Versatile reagents for biocompatible and selective amide bond formation

**DOI:** 10.1126/sciadv.adp7544

**Published:** 2024-09-18

**Authors:** Khokan Choudhuri, Zhenguo Zhang, Teck-Peng Loh

**Affiliations:** ^1^ College of Advanced Interdisciplinary Science and Technology, Henan University of Technology, Zhengzhou 450001, China.; ^2^ Division of Chemistry and Biological Chemistry, School of Chemistry Chemical Engineering & Biotechnology, Nanyang Technological University, 21 Nanyang Link, Singapore, 637371, Singapore.

## Abstract

The study introduces a previously unidentified method for amide bond formation that addresses several limitations of conventional approaches. It uses the β-silyl alkynoate molecule, where the alkynyl group activates the ester for efficient amide formation, while the bulky TIPS (triisopropylsilane) group prevents unwanted 1,4-addition reactions. This approach exhibits high chemoselectivity for amines, making the method compatible with a wide range of substrates, including secondary amines, and targets the specific ε-amino group of lysine among the native amino ester’s derivatives. It maintains stereochemistry during amide bond formation and TIPS group removal, allowing a versatile platform for postsynthesis modifications such as click reactions and peptide-drug conjugations. These advancements hold substantial promise for pharmaceutical development and peptide engineering, opening avenues for research applications.

## Teaser

An example of a catalyst-free biocompatible amide bond formation reaction using β-silyl alkynoates is reported.

## INTRODUCTION

**Fig. 1.**
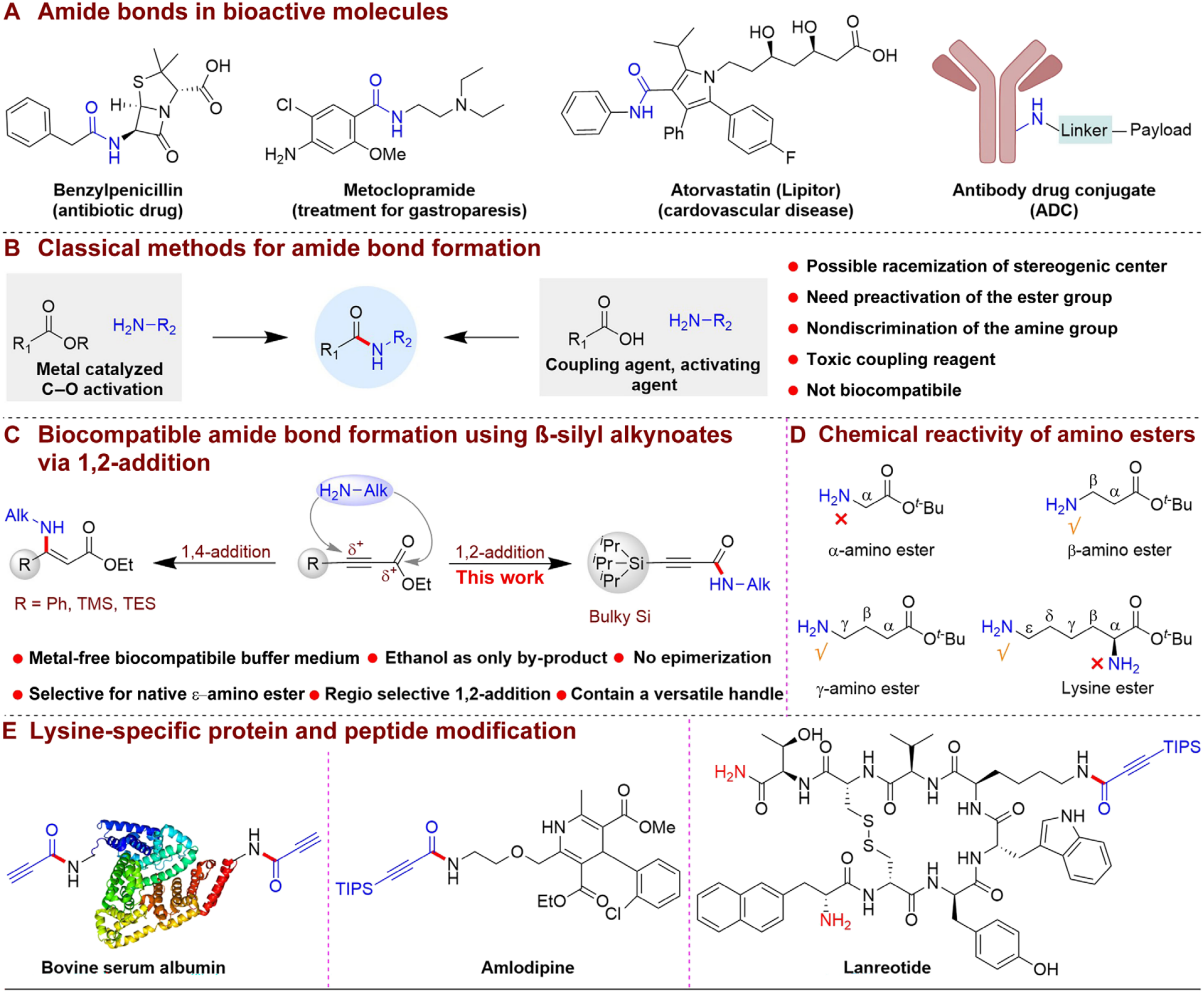
Background and reaction design. (**A**) Amide bonds in bioactive molecules. (**B**) Classical methods for amide bond formation. (**C**) This work: Biocompatible amide bond formation using β-silyl alkynoates via 1,2 additions. (**D**) Chemical reactivity of amino esters. (**E**) Application of the reaction in protein, drug, and peptide modification.

Amide bonds are pivotal in organic synthesis (*1*–*7*), forming the backbone of proteins (*8*) and a variety of synthetic polymers (*9*). These bonds are foundational in the structure of biomolecules and crucial in the development of innovative conjugated products such as antibody-drug conjugates (ADCs) (*10*, *11*), insulin analogs (*12*), and ubiquitin conjugates (*13*). It is estimated that around 25% of all Food and Drug Administration–approved pharmaceutical drugs contain an amide bond, underscoring its significance. Representative amide-containing marketed drugs include penicillin, metoclopramide, atorvastatin, etc. In addition, considerable attention is devoted to amide-containing payloads, particularly in ADCs, which are designed for the selective targeting of cancer cells (Fig. 1A). In consequence, a diverse array of synthetic methods (*14*–*16*) has been developed for amide bond formation (Fig. 1B). Among the various synthetic approaches, the direct coupling of carboxylic acids or alcohols with amines stands out as the most prevalent strategy, often aided by coupling reagents. In recent years, a multitude of coupling reagents, including carbodiimides (*17*), phosphonium salts (*18*), and aminium/uronium derivatives (*19*), have been developed and successfully brought into commercial use. In 1975, Bragg and Hou (*20*) introduced *N*-hydroxysuccinimide esters as reactive ends of homobifunctional cross-linkers, which revolutionized the amide bond construction in peptides and proteins. Zhao and colleagues recently introduced ynamide-mediated (*21*–*23*) and allenone-mediated (*24*) coupling reactions between carboxylic acid and amine. Nevertheless, these methods frequently lack specificity toward native amino acids and suffer from sub-stoichiometric efficiency, resulting in substantial waste and complicating the subsequent purification process to isolate the desired products. Another notable strategy for amide bond formation is native chemical ligation which was independently developed by Kent (*25*) and Tam (*26*) for assembling amide bonds in peptides and proteins inspired by Wieland’s observations (*27*). However, its applicability is limited to specific substrates only. Furthermore, traditional methods (*28*, *29*) for peptide syntheses rely heavily on the legacy reagents and technologies developed in the 1950 to 1980s, which are reaching their inherent limitations including the potential risk of racemization, and nonbiocompatible conditions. To address these issues, the American Chemical Society Green Chemistry Institute Pharmaceutical Roundtable has emphasized the importance of developing catalytic or catalyst-free methods for amide bond formation as a key initiative in Green Chemistry (*30*, *31*). Innovations in this field have been notable. In 2007, Milstein (*32*) and co-workers introduced a breakthrough with their ruthenium-catalyzed dehydrogenative coupling of amines and alcohols (*33*). This was followed by Madsen (*34*) and his team, who expanded the scope of dehydrogenative coupling with an alternative catalyst system (*35*). Hall (*36*) and colleagues recently presented a method for boronic acid–catalyzed amidation reactions using carboxylic acids (*37*). Direct aminolysis of the readily available, simple, and stable ester with an amine could represent an attractive method for amide formations because of its enhanced atom economy and the accessibility of starting materials. However, this strategy suffers from the prerequisite of C─O bond activation of the esters by transition metal catalysts. Therefore, amide bond formation by using stable ester under biocompatible conditions remains a substantial hurdle (*38*–*44*).

Our approach to amide bond formation addresses these challenges by forgoing conventional reagents and using β-silyl alkynoates (*45*–*47*) to couple with amines under biocompatible conditions (Fig. 1C). We anticipate that the bulky triisopropylsilyl group will suppress the 1,4-addition reactions (*48*–*50*), while the alkynyl group enhances the ester group’s reactivity and directs the nucleophiles toward forming the desired amide bond. The strategic removal of the silicon group without epimerization of the stereogenic center facilitates the postmodification of a diverse range of chemical reactions. Our approach not only offers selective amide bond formation that preserves stereogenic centers but also leverages the terminal amide’s alkyne anchor as a Michael acceptor. This capability will introduce additional functionality such as the execution of click reactions (*51*), peptide-drug conjugates (PDCs) (*52*), etc., which represent a substantial advancement in the synthesis and functionalization of biomolecules under biocompatible conditions (*53*–*55*). Furthermore, our method exhibits a selective affinity toward alkynyl esters over other alkane and alkene esters and is compatible with a wide array of aliphatic primary and secondary amines. A distinct feature of the reaction is its selectivity toward the β, γ, and long-chain amino esters. It uniquely maintains the integrity of stereogenic centers in peptides and specifically targets the ε-amine of lysine among native amino esters. However, it demonstrates resistance to the amine that is attached to the α-position of the electron-withdrawing groups such as esters or amides (Fig. 1D). Expanding upon this observation, we have demonstrated the applicability of our methodology in the realms of peptide chemistry, protein engineering, and drug development (Fig. 1E). Such developments have profound implications for biomedical research and biotechnology, marking a substantial leap forward in these fields.

## RESULTS AND DISCUSSION

In our continuous pursuit of developing a green method for amide bond formation, which is also suitable for amine conjugation in biological systems, we conducted an in-depth study of the reaction using ethyl 3-(triisopropylsilyl)propiolate (**1a**) with benzylamine (**2a**) under catalyst-free conditions, the results are summarized in Table 1. We performed this reaction in both aqueous media and a variety of buffer solutions at different pH levels while maintaining a constant temperature of 37°C (Table 1, entries 1 to 6). Notably, using a phosphate-buffered saline (PBS) at pH 7 resulted in an excellent yield of 80% within 48 hours, with the remaining substrate left unreacted (Table 1, entry 3). Our experiments with organic solvents, including dimethyl sulfoxide (DMSO), acetonitrile (CH_3_CN), ethanol (EtOH), tetrahydrofuran (THF), and others, yielded results that were less than satisfactory, refer to the Supplementary Materials. We expanded our studies by incorporating various organic solvents into a PBS buffer solution at pH 7 (Table 1, entries 7–9). A notable finding was that combining buffer with ethanol in a 4:1 ratio resulted in the most effective outcomes, achieving an 85% yield of the desired product within 48 hours (Table 1, entry 9). In addition, we observed an improvement in the reaction yield up to 92% for **3a** with slight temperature increases, reaching an optimum at 40°C (Table 1, entry 10). However, it is crucial to note that while elevating the temperature to 80°C, the reaction rate can be substantially hastened to complete it within 24 hours (Table 1, entry 11). Nevertheless, we are continuing our investigation at 40°C under biocompatible conditions, motivated by our keen interest in applying this method within the realm of protein and peptide chemistry. To eliminate any possibility of catalytic interference from glass silica, we opted to perform the reaction in a plastic reaction vial, achieving an outstanding 82% yield of **3a** (Table 1, entry 12). Subsequently, inductively coupled plasma mass spectrometry (ICP-MS) analysis confirmed that negligible metal presence in the reaction mixture was detected at parts pe r billion levels (details in the "ICP-MS for the determination of the metal ions" section in the Supplementary Materials).

**Table 1.**
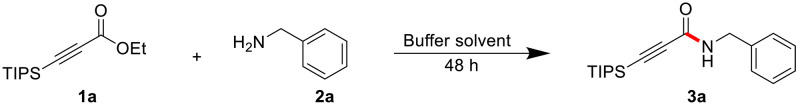
Optimization of reaction conditions. Condition: **1a** (50 mg, 0.196 mmol), **2a** (0.295 mmol, 1.5 equiv.), solvent (0.5 mL) for 48 hours.

Entry	Solvent	pH value	Temperature (°C)	3a (%)*
1	H_2_O	7	37	56
2	PBS buffer	6	37	30
3	PBS buffer	7	37	80
4	PBS buffer	8	37	54
5	Tris-HCl buffer	8	37	40
6	H_2_O/EtOH (4:1)	7	37	84
7	PBS/CH_3_CN	7	37	46
8	PBS/DMSO	7	37	48
9	PBS/EtOH	7	37	85
10	PBS/EtOH	7	40	92 (88)^†^
11	PBS/EtOH	7	80	90^‡^
12	PBS/EtOH	7	40	86^§^

*Yield was determined by ^1^H NMR using CH_2_Br_2_ as an internal standard.

†Isolated yield.

‡Reaction was conducted for 24 hours.

§Reaction was performed in a plastic vial. PBS buffer, phosphate-buffered saline; PBS/organic solvent = 4/1.

**Fig. 2.**
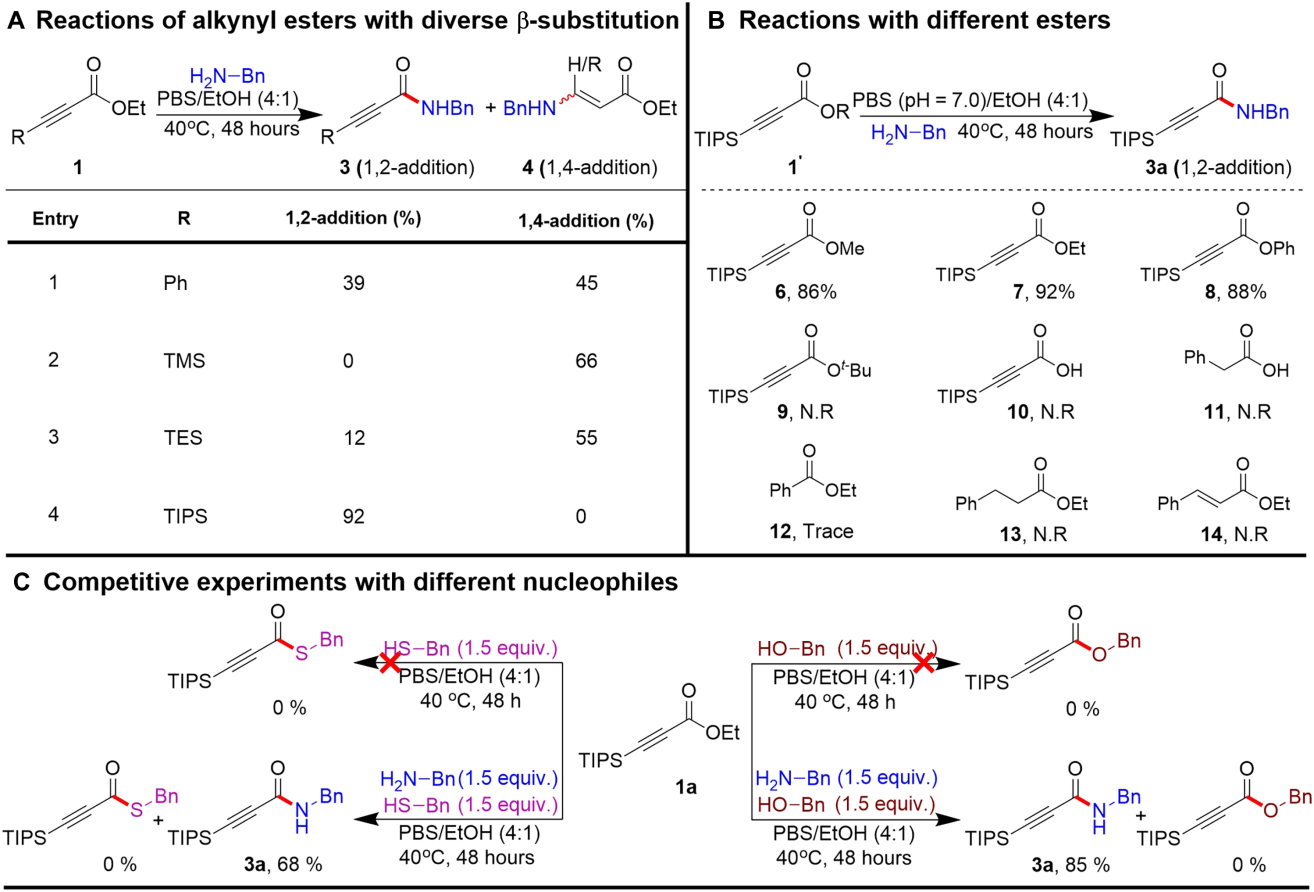
Control experiments. (**A**) Effect of β-substitution on chemoselective 1,2- versus 1,4-addition. (**B**) Comparison of ester reactivity. (**C**) Nucleophilic competitive experiment. Reaction yield was determined from the crude reaction mixture by ^1^H NMR using CH_2_Br_2_ as an internal standard. N.R, no reaction; PBS, phosphate-buffered saline (pH 7.0).

We next systematically investigated various β-substituted alkynyl esters, examining their reactions with benzylamine (**2a**) under optimal conditions. We observed that phenyl-substituted alkynyl esters produced both 1,2- and 1,4-addition products with 39 and 45% yields, respectively (Fig. 2A, entry 1). However, using of less bulky TMS-substituted alkynyl ester results in the formation of a desilylated 1,4-addition product in 66% yield (Fig. 2A, entry 2). A notable change was evident when we used the bulkier triisopropylsilane (TIPS)–substituted alkynyl ester, which shifted the reaction away from the 1,4-pathway, resulting in a predominant 1,2-addition product with a yield of 92% (Fig. 2A, entry 4). Subsequently, we focused on assessing the effect of various ester groups on these reactions. The results, presented in the accompanying table in Fig. 2B, showed that, regardless of the ester group variations, the reactions favored the formation of 1,2-addition products with commendable efficiency. However, the substitution of the ester group with either a tert-butyl or an acid group failed to produce any product formation (Fig. 2B, 9 to 11). A notable observation was found that the reactions of simple alkane or alkene esters did not yield the anticipated amide product formation, suggesting the pivotal role of the alkyne functionality in activating the ester carbonyl group for the 1,2-addition reaction (Fig. 2B, 13 and 14). This finding underscores the importance of the alkyne group in the reaction mechanism. Moreover, distinguishing between different ester compounds offers valuable insights for the functionalization of complex molecules containing multiple ester functionalities, paving the way for more tailored applications in chemical synthesis.

We then proceeded to conduct competitive experiments to assess the selectivity of the reaction toward amine in the presence of both benzyl thiol and alcohol under our optimized reaction conditions, as outlined in Fig. 2C. We were pleased to find that no C─S or C─O bond formation occurred, and only products resulting from C─N bond formation were observed. Notably, when we conducted separate reactions of β-silyl alkynoates with benzyl mercaptan and benzyl alcohol, no product formation was observed in either of the reactions. The results of these experiments emphasized the substantial chemoselectivity of the reaction toward amines.

### Scope of substrates

**Fig. 3.**
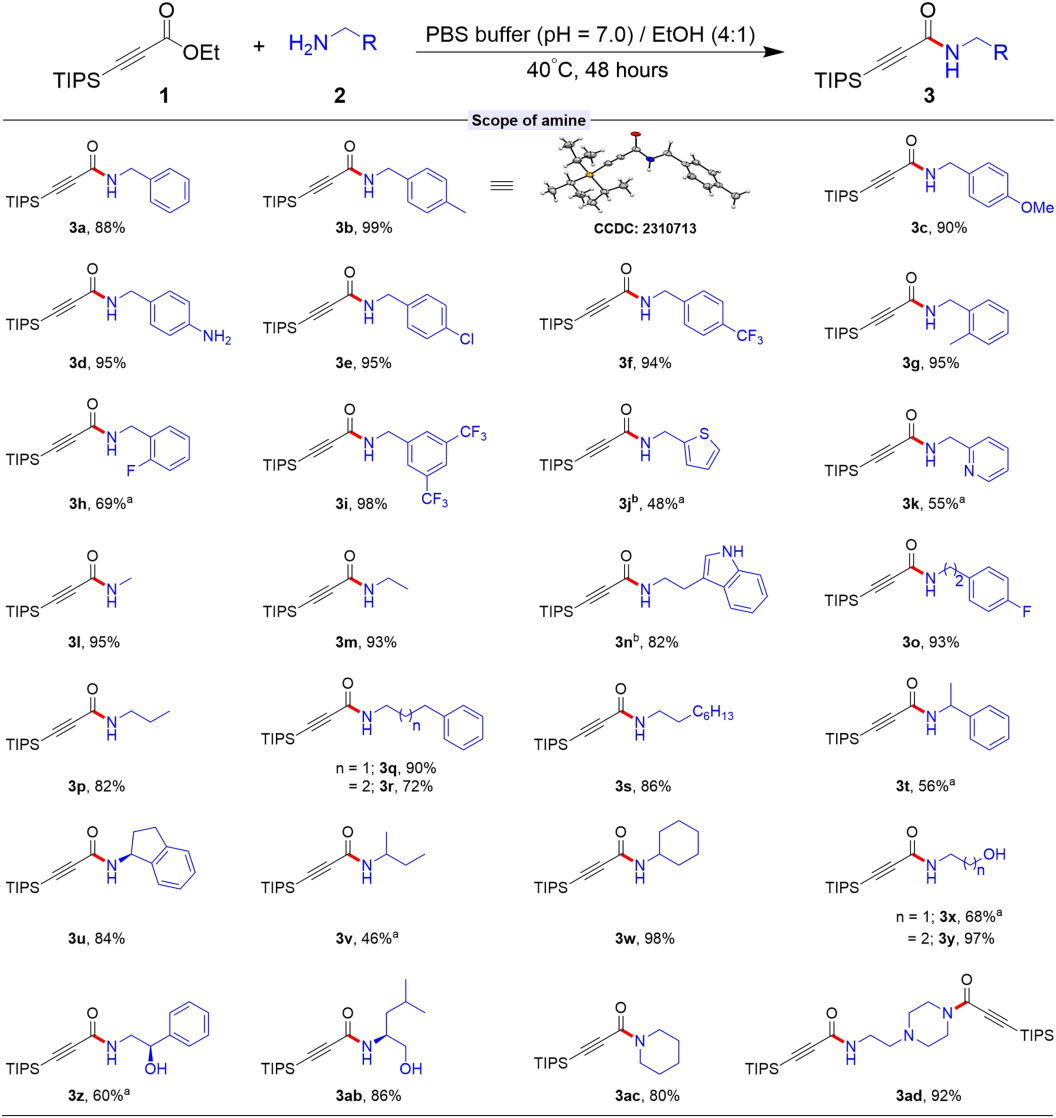
Substrate scope of amine nucleophiles to β-silyl alkynoates. **1a** (50 mg, 0.196 mmol), **2** (0.295 mmol, 1.5 equiv.), and solvent (0.5 ml) for 48 hours. Isolated yield. ^a^The remaining starting materials were recovered (for details, see Supplementary Materials). ^b^PBS buffer (pH = 7.0)/CH_3_CN (4:1) was used.

With our optimized reaction conditions in hand, we explored the substrate scope of amines in forming the amide bonds with TIPS-conjugated ester **1a**, as depicted in Fig. 3. Our experiments revealed that both linear and branched chain primary alkyl amines were effectively accommodated, resulting in the formation of adducts **3a** to **3ad** with moderate to excellent yields. The use of benzylic amines with various substituents at the ortho-, meta-, and para-positions on the phenyl rings led to the corresponding products **3a** to **3i** with excellent efficiency. Notably, the introduction of fluorine at the ortho position on the phenyl group yielded **3h** with a moderate outcome. This transformation proved to be equally effective with heteroarenes, affording products **3j** and **3k** with yields of 48 and 55%, respectively. In contrast, reactions with linear chain alkyl amine effectively produce the corresponding products **3l** to **3s** with yields ranging from 95 to 72%. Moreover, the branch alkyl amine also demonstrated remarkable effectiveness in producing the corresponding products **3t** to **3v**.

**Fig. 4.**
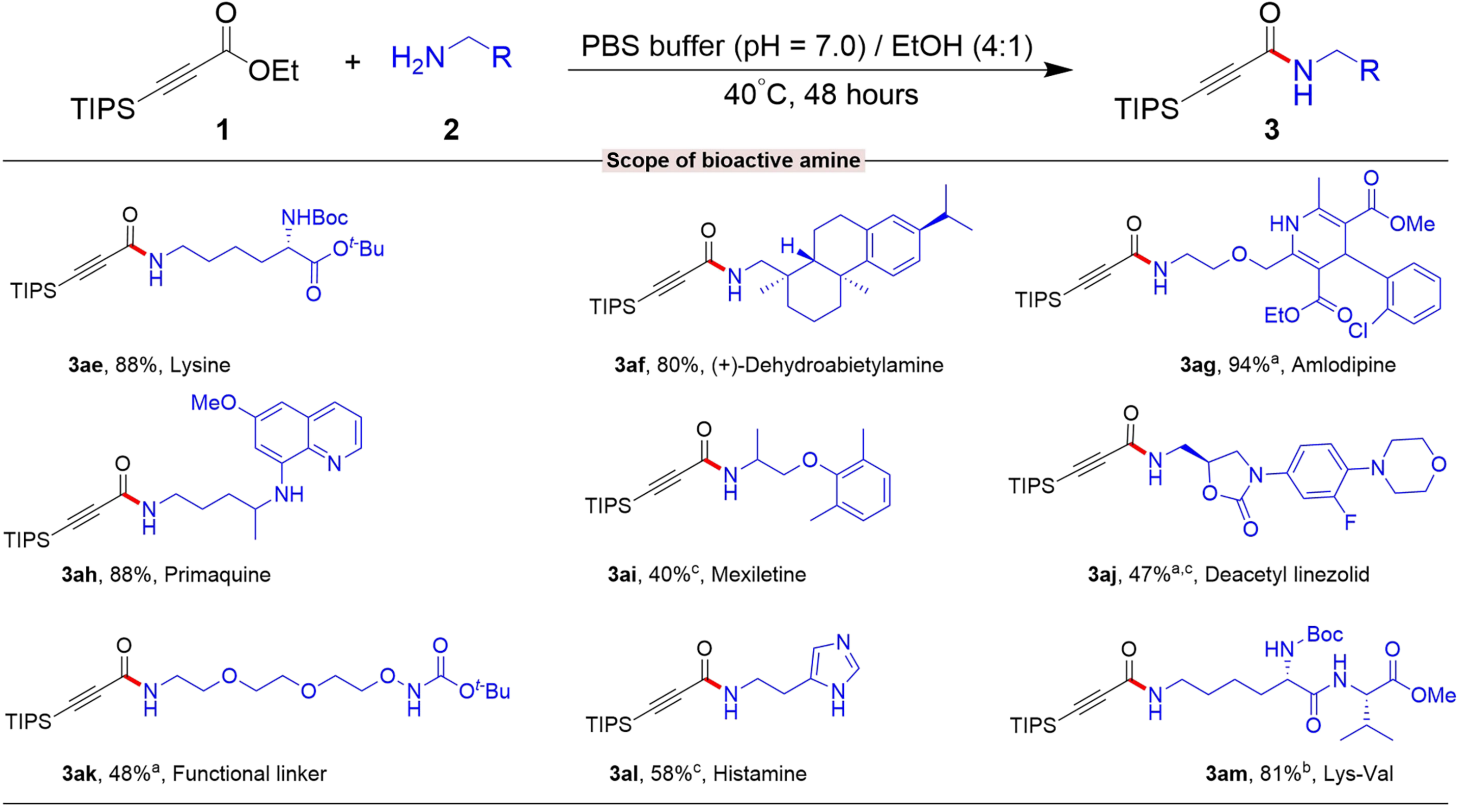
Scope of the amine-containing bioactive molecule and drug modification. **1a** (50 mg, 0.196 mmol), **2** (0.295 mmol, 1.5 equiv.), and solvent (0.5 ml) for 48 hours. Isolated yield. ^a^PBS buffer (pH = 7.0)/CH_3_CN (4:1) solvent was used. ^b^PBS buffer (pH = 7.0) was used as a solvent. ^c^The remaining starting materials were recovered (for details, see Supplementary Materials).

The use of cyclohexyl amine produced products **3w** with a notable yield of 98%. The reaction of amino alcohol with **1a** was selective, preserving the hydroxy group intact in the adduct **3x** to **3ab**. Secondary amines **2ac** and **2ad** also reacted favorably, producing products with 80 and 92% yields. However, this method failed to yield any amide product formation when aniline, a heteroaryl amine such as 3-aminopyridine, and α-amino esters were used. To further evaluate the versatility of this reaction, we reacted β-silyl alkynoates with various biomolecules and drugs containing free amines (Fig. 4). In an initial experiment, *N*-Boc-Lysine-tert-butyl ester was reacted with **1a** under standard conditions, successfully functionalizing this lysine derivative to yield adduct **3ae** with remarkable conversion. Encouraged by these results, we extended this approach to modify peptide and drug molecules containing free amines using **1a**. For example, amlodipine, commonly used for treating hypertension, was modified to produce **3ag** with an impressive 94% yield. In addition, drugs like dehydroabietylamine, deacetyl linezolid, mexiletine, primaquine, and histamine were efficiently modified, yielding their respective products with good to excellent efficiency. The functional linker Boc-Aminooxy-PEG2-C2-amine also reacted successfully with **1a**, resulting in product **3ak** with a commendable 48% yield.

**Fig. 5.**
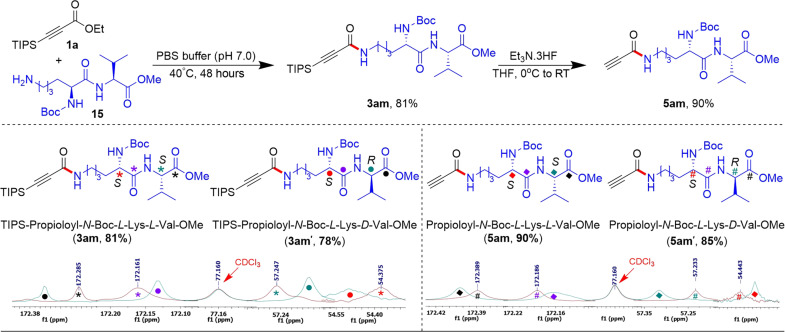
Stereoselectivity assessment in peptides using ^13^C NMR spectrum for epimer of amine-conjugated product.

Determining whether this method can be applied to peptides or proteins without causing epimerization at existing stereogenic centers is critical, as depicted in Fig. 5. This concern particularly applies during the amide bond formation step and the subsequent removal of the TIPS group. To evaluate this, we tested the method on both *L*-lysyl-*L*-valinate and *L*-lysyl-*D*-valinate derivatives of dipeptides **15** and **15′** and successfully obtained the desired amide products with yields of 81 and 78%, respectively. Subsequently, the removal of the TIPS group was efficiently achieved by using Et_3_N.3HF reagent in THF at room temperature. Nuclear magnetic resonance (NMR) analysis, including both ^1^H NMR and ^13^C NMR, confirmed that neither the amide bond formation nor the TIPS group removal led to any epimerization. The stacked plot of the ^13^C NMR spectrum revealed that none of the chiral carbon peaks overlapped with each other in either of the modified dipeptides **3am** and **3am′** or the desilylated dipeptides **5am** and **5am′** (Fig. 5). After successfully confirming the retention of configuration at the stereogenic centers throughout the processes of amide bond formation and the removal of the TIPS (triisopropylsilyl) group in the synthesized peptides, we proceeded to the next step to explore the potential synthetic applications of the alkyne anchor present in the amide products.

**Fig. 6.**
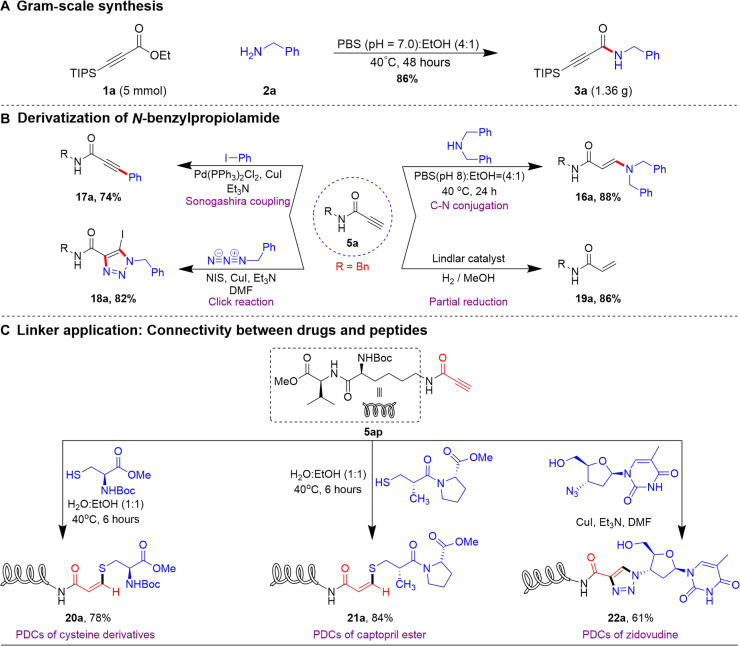
Application of *N*-substituted propiolamide. (**A**) Gram-scale synthesis. (**B**) Product derivatization of *N*-benzylpropiolamide (**C**) Application in linker chemistry for peptide-drug conjugation (PDCs).

The gram-scale synthesis of **3a** confirms the viability and practicality of the reaction, ensuring its potential for large-scale use in both research and industry (Fig. 6A). It is noteworthy that the *N*-propiolamide derivatives obtained from our method exhibit highly resourceful building blocks in organic synthesis (*56*–*58*) (Fig. 6B). The alkyne moiety of **5a** readily undergoes reaction with amines under biocompatible conditions, yielding a C-N–conjugated adduct **16a** with outstanding yield. Moreover, it effectively engages in Sonogashira coupling and azide-alkyne click reactions, yielding the desired products **17a** and **18a** with excellent efficiency. In addition, the alkyne group within these compounds (**5a**) can also be selectively reduced to the alkene derivative **19a** using a Lindlar catalyst.

During the last decades, peptide-drug conjugates (PDCs) (*59*) have received substantial attention as promising targeting therapies, akin to antibody-drug conjugates (ADCs). These conjugates typically consist of monoclonal peptides, drug payloads, and cleavage/noncleavage linkers. Capitalizing on the inherent versatility (*60*) of *N*-propiolamide derivatives, we carefully combine drug molecules with peptide (**5am**) using an alkyne anchor linker, making it simpler to blend them smoothly into the formation of PDCs (Fig. 6C). This approach harnesses the unique properties of *N*-propiolamide derivative to accurately join drugs with peptides, which could enhance the efficacy and specificity of targeted therapies. The addition of a mercaptan with **5am** provides a versatile platform for synthesizing sulfur-containing drug conjugation (**20a** and **21a**) under biocompatible conditions. Moreover, the alkyne group of **5am** readily engages in copper-catalyzed azide-alkyne cycloaddition (click) reactions, enabling the conjugation of the Zidovudine drug **22a** in 61% of yield. These findings underscore the efficacy of *N*-propiolamide derivatives as anchors for coupling with various biomolecules, leading to the production of valuable PDCs. Such versatility broadens the potential applications of this methodology in biochemical conjugation and drug development.

**Fig. 7.**
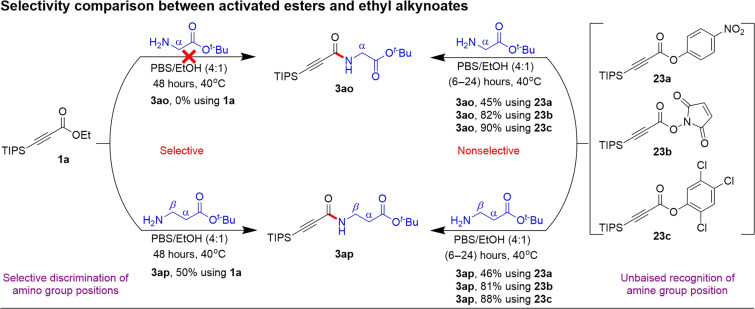
Selectivity comparison between activated esters and ethyl alkynoates.

**Fig. 8.**
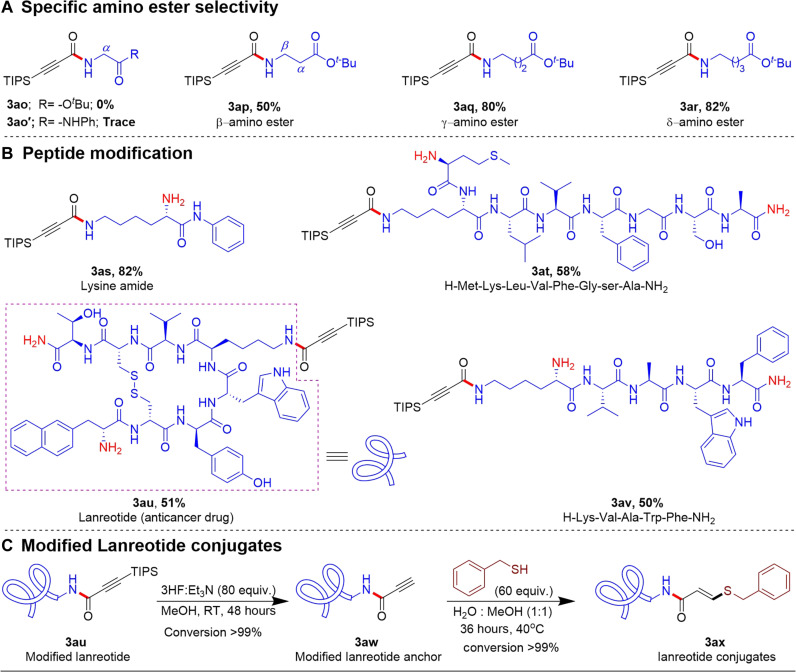
Amino ester selectivity and polypeptide modification with 1a. (**A**) Specific amino ester selectivity. (**B**) Polypeptide modification with **1a**. (**C**) Modified lanreotide conjugates.

As previously mentioned, we were intrigued to find that α-amino esters did not yield the desired amide product with **1a**, while linear alkyl amines did so with good efficiency. Therefore, we conducted a comparative study between the activated esters and β-silyl ethyl alkynoates regarding their specific reactivity toward amino esters (Fig. 7). Notably, most of the literature known activated esters are unstable under ambient biocompatibility conditions and are typically prepared in situ and used immediately for amidation reactions. Because of their high reactivity, activated esters readily react with both α- and β-amino esters to form the corresponding amide products. For a comprehensive comparison, we synthesized p-nitrophenyl (**23a**), *N*-hydroxysuccinimide (**23b**), and 2,4,5-trichlorophenyl (**23c**) silyl alkynoates. It is essential to highlight that all the activated alkynoates demonstrated equal reactivity toward both α- and β-amino esters, yielding the respective products with good to excellent efficiency. This observation highlights the lack of selectivity in the discrimination of the positions of the amine groups in amino ester derivatives. However, a systematic investigation into the reactivity of ethyl alkynoates toward various amino esters revealed that α-amino esters (**3ao**) displayed negligible reactivity, while α-amino amides (**3ao′**) yielded only traces of product. Conversely, β-amino esters, γ-amino esters, and δ-amino esters efficiently produced the desired products with yields of 50, 80, and 82%, respectively (Fig. 8A).

Further exploration of the reactivity of β-silyl alkynoates, we conducted experiments using lysine amide (**3as**), which contains two free amino groups (Fig. 8B). The results demonstrated that only the side chain ε-NH_2_ of lysine reacted with the β-silyl alkynoates, while the α-NH_2_ group remained unreacted. This indicates that the presence of an electron-withdrawing group adjacent to the amino group hinders the reaction. These findings highlight a notable selectivity for lysine conjugation over other native amino ester derivatives.

On the basis of the controlled experiment (Figs. 2B and 8A), a plausible mechanism for the amide bond formation reaction under biocompatible conditions has been rationalized. In β-silyl alkynoates, the sp-hybridized carbon of the alkyne moiety induces a partial positive charge at the ester’s carbonyl group by withdrawing electron density. This electron-deficient carbonyl carbon then becomes susceptible to nucleophilic attack by the amine, resulting in the formation of the amide product and the release of ethanol as a byproduct. The reaction favors the ε-amino group of lysine because of its higher nucleophilicity, while the limited nucleophilicity of the α-amino group in native amino esters hinders the desired outcome.

These initial findings encouraged us to expand our method to various polypeptide molecules containing different nucleophilic amino acid side chains (Fig. 8B). Notably, polypeptides with the ε-amine group of lysine underwent conjugation with **1a**, while other nucleophilic groups within the amino acid chains and the terminal amino group were mostly unaffected, as confirmed by liquid chromatography tandem mass spectrometry (LC-MS/MS) analysis of **3at**. Consequently, the desired amide products **3at**, **3au**, and **3av** were formed, achieving yields of 58%, 51%, and 50%, respectively. To verify the specific selectivity of lysine in cyclic peptides such as lanreotide, which contain multiple nucleophilic amino acid chains, we treated modified lanreotide with tris(2-carboxyethyl)phosphine to break the S─S linkage, forming a linear peptide chain. LC-MS/MS analysis revealed that only the ε-NH_2_ group of lysine in lanreotide was reacting by β-silyl alkynoates, while other nucleophilic amino acids, such as threonine, cysteine, tryptophan, tyrosine, and the terminal amine, remained mostly untouched. The desilylation of modified lanreotide could also be achieved using Et_3_N.3HF reagent in a polar protic methanol solvent at room temperature (Fig. 8C). This step enabled further conjugation with a benzyl mercaptan, forming the desired conjugated product (**3ax**).

**Fig. 9.**
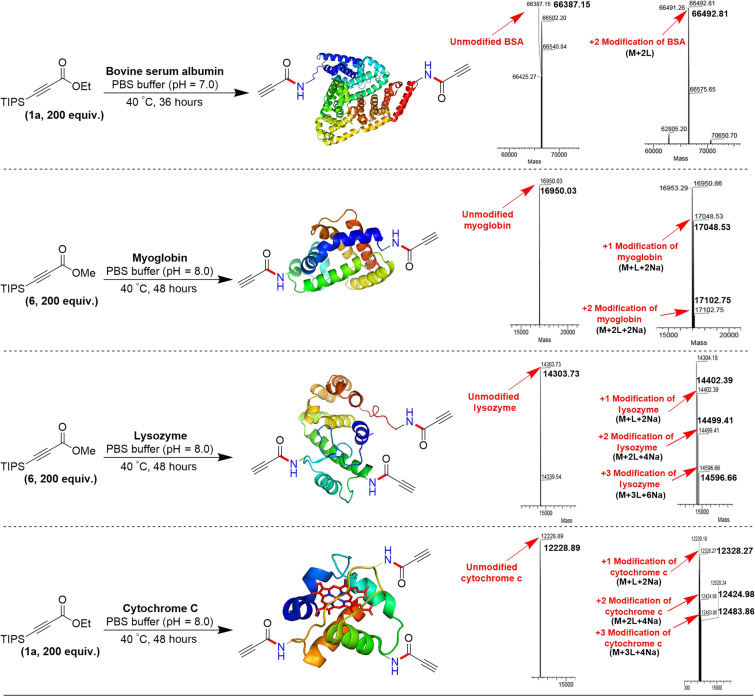
Modification of bovine serum albumin, myoglobin, lysozyme, and cytochrome c protein using β-silyl alkynoates.

Leveraging this success, we extended our methodology to protein modification (*61*) targeting lysine under biocompatible conditions, using bovine serum albumin (BSA, 90 μM) as a model protein (Fig. 9). The deconvoluted mass spectrum revealed that the molecular weight of unmodified BSA (66,387 Da) shifted to 66,491 Da, indicating the modification of BSA with two ligands and the release of the TIPS group. Furthermore, LC-MS/MS analysis identified all relevant peptide segments and modifications of a free lysine residue (K361) by a molecule (51.99492 Da) corresponding to **1a** after the TIPS group fell off. To further demonstrate the versatility of our method, we applied it to myoglobin protein from equine skeletal muscle, which contains 19 lysine residues. Incubation with 200 equivalents of β-silyl methyl alkynoates in PBS buffer (pH 8.0) resulted in conjugated products with a maximum of twofold modification, as identified by LC-MS analysis. The deconvoluted mass spectrum showed that unmodified myoglobin (16,950 Da) shifted to 17,048 Da for onefold modification, representing myoglobin modified with one desilylated ligand and the consumption of two sodium ions. For twofold modification, LC-MS showed a peak at 17,102 Da. Further LC-MS/MS analysis confirmed all relevant peptide segments and the modification of free lysine residues (K80 and K88) by a molecule (51.99492 Da) corresponding to **1a** after the deprotection of the TIPS group. Inspired by these results, we also modified lysozyme and cytochrome C using β-silyl alkynoates. Both proteins showed a maximum of threefold modification after the removal of the TIPS group. Both the ethyl and methyl alkynoates (**1a** and **6**) produced the same modified biopolymer products with myoglobin and lysozyme after TIPS group deprotection. This may be due to the presence of a free carboxylic acid group, which induces TIPS group hydrolysis over prolonged reaction times. Despite a relatively sluggish reaction rate, our approach exhibited remarkable selectivity for lysine, while preserving the integrity of other amino groups within the protein. This level of specificity underscores the potential of our method for targeted protein modifications.

In conclusion, our study introduces a pivotal β-silyl alkynoate-based methodology for amide bond formation, marking a substantial leap in organic chemistry and biotechnology. This technique is characterized by the activation of the ester group through an alkynyl group and the effective suppression of the 1,4-addition pathway by a bulky TIPS group, ensuring both high selectivity and efficiency. A distinctive feature of our method is its broad compatibility with various amines, including secondary amines, and its unique ability to differentiate the amino group in native amino ester derivatives. This specificity proves especially beneficial in targeting the ε-amine of lysine, crucial in peptide and protein engineering, while concurrently avoiding other native amino ester, substantially broadening the scope of this method. Furthermore, our approach preserves the integrity of stereogenic centers in peptides throughout the amide bond formation and the TIPS group removal, an aspect vital for the pharmaceutical industry. The creation of a versatile alkynyl anchor functional group in our amide products opens avenues for further functionalization, such as click reactions, PDCs, etc. Notably, our method exhibits exclusive reactivity with β-silyl alkynoates, as evidenced by no reaction or poor selectivity with other ester compounds, and efficiently yields ethanol as the only byproduct, underscoring its green chemistry credentials. This substantial advancement overcomes the limitations of traditional amide formation techniques and offers immense potential for revolutionizing biomolecule synthesis and modification, thereby catalyzing progress in drug development and biotechnological applications.

## MATERIALS AND METHODS

### General procedure for amine addition to β-silyl alkynoates

To a 4-ml glass vial, 0.1 ml of ethanol was added to the mixture of ethyl 3-(triisopropylsilyl) propiolate (**1a**, 50 mg, 0.196 mmol) and benzylamine (**2a**, 0.295 mmol). The mixture was stirred for 5 min to achieve a homogeneous solution. Subsequently, 0.4 ml of pH-neutral phosphate buffer was added, and the resulting mixture was vigorously stirred for 48 hours at 40°C. The mixture was diluted with ethyl acetate, washed with water, and dried over anhydrous sodium sulfate. The crude mixture was purified over silica gel column chromatography using 30% ethyl acetate inhexane as an eluent to afford the desired product (**3a**).

### General procedure for desilylation of propiolamide

To a 10-ml round-bottom flask, **3a** (60 mg, 0.190 mmol) and triethylamine trihydrofluoride (5 equiv.) were taken in 2 ml of THF at 0°C under an argon atmosphere. Then the mixture was vigorously stirred for 12 hours at room temperature. After that, the mixture was washed with water, extracted with ethyl acetate, and dried over anhydrous sodium sulfate, concentrated under reduced pressure. The yield of **5a** was calculated after purification of the crude mixture using silica gel flash column chromatography.

## Supplementary Materials

### The PDF file includes:

Supplementary Materials and Methods Supplementary Text Tables S1 to S5 Figs. S1 to S163 Legend for data S1
