# Supplementary material for: β-Silyl alkynoates: Versatile reagents for biocompatible and selective amide bond formation
Source: Sci Adv. 2024 Sep 18;10(38):eadp7544. doi: 10.1126/sciadv.adp7544 (PMC11421574; doi:10.1126/sciadv.adp7544)
Supplement: Supplementary file 1 — Supplementary Materials and Methods Supplementary Text Tables S1 to S5 Figs. S1 to S163 Legend for data S1 [file sciadv.adp7544_sm.pdf]

Science Advances
AAAS

Supplementary Materials for

β-Silyl alkynoates: Versatile reagents for biocompatible and selective amide bond formation

Khokan Choudhuri *et al.*

Corresponding author: Teck-Peng Loh, teckpeng@ntu.edu.sg

Sci. Adv. **10**, eadp7544 (2024)
DOI: 10.1126/sciadv.adp7544

The PDF file includes:

Supplementary Materials and Methods
Supplementary Text
Tables S1 to S5
Figs. S1 to S163
Legend for data S1

Other Supplementary Material for this manuscript includes the following:

Data S1

Table of Contents

General Information. S3

Substrate Preparation. S4

General procedure for amine addition to β-silyl alkynoates: S7

Optimization of Reaction Conditions[a]. S8

ICP-MS for the Determination of the Metal Ions. S14

Dipeptide Synthesis and Epimerization Experiment. S15

Gram Scale Synthesis and Product Derivatization. S20

Selectivity comparison between activated ethyl alkynoates and *β*-silyl alkynoates.......S28

Experiments of Peptide Modification. S31

Experiment for Protein Modification. S44

Crystallographic Investigation. S66

NMR Data for Compounds S67

NMR Spectra S78

General Information.

All the chemicals and solvents were purchased from commercial sources and used as received unless otherwise specified. All the reactions were carried out in an open atmosphere condition. Flash chromatography was performed using Merck 40-63 D 60Å silica gel. Phosphate buffer solution (pH 7, Cat No. 38712) was purchased from Alfa Aesar, which contains water (99.05% w), potassium dihydrogen phosphate monohydrate (0.90% w), and sodium hydroxide (0.05% w). ^{1}H, ^{13}C, and ^{19}F NMR spectra of the compounds were recorded on either Bruker 400 MHz, JEOL 400 MHz, and JEOL 500 MHz NMR spectrometer at 25 ºC. The chemical shift values in ppm (δ) were reported for the residual chloroform (7.26 for ^{1}H and 77.16 ppm for ^{13}C) and DMSO (2.50 for ^{1}H and 39.52 ppm for ^{13}C). High-resolution mass spectra (HRMS) were recorded on a Waters G2-XS Q-tof spectrometer with ESI mode unless otherwise stated.

Substrate Preparation.

All the alkyne ester was prepared according to the previously reported literature (45-47) method with slight modifications.

General procedure A.

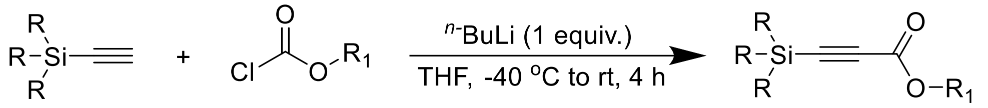

To a stirred solution of ethynyltriisopropylsilane (1 ml, 4.45 mmol) in anhydrous THF (20 mL), $^{n-}$BuLi (2 M in cyclohexane, 2.23 mL, 4.45 mmol) was added at -40 °C under Argon atmosphere. The resulting mixture was stirred at the same temperature for 1 hour. Then the corresponding chloroformate (4.90 mmol) in anhydrous THF (5 mL) was added dropwise to the reaction mixture at -40 °C. The reaction mixture was stirred for 90 minutes at the same temperature, then it was warmed to room temperature and allowed to be stirred for another 90 minutes. After that, the mixture was quenched with NH_4Cl (sat. aq, 20 mL). The organic layer was extracted with diethyl ether (3 × 10 mL), dried over anhydrous Na_2SO_4, and concentrated in *vacuo*. To afford the pure product, the crude reaction mixture was purified over silica gel column chromatography using ethyl acetate/hexane as eluent.

General procedure B.

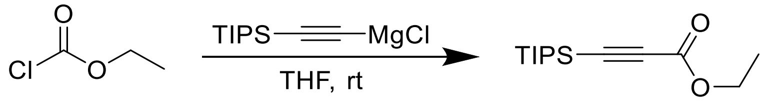

To a stirred solution of ethynyltriisopropylsilane (1 ml, 4.45 mmol) in anhydrous THF (15 ml), isopropyl magnesium chloride (2 M in THF, 2.68 mL, 5.35 mmol) was added dropwise to the solution while maintaining the internal temperature (10-15) °C. The mixture was stirred for 45 minutes at room temperature, and then ethyl chloroformate (5.35 mmol) was slowly added. The resulting solution was stirred for 12 hours at room temperature. The organic layer was extracted with diethyl ether (3 × 10 mL), dried over anhydrous Na_2SO_4, and concentrated in *vacuo*. To afford the pure product, the crude reaction mixture was purified over silica gel column chromatography using ethyl acetate/hexane as eluent.

General procedure C.

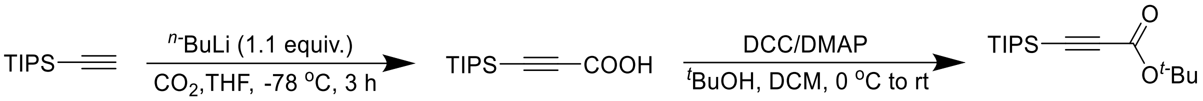

Step I: To a stirred solution of ethynyltriisopropylsilane (1 ml, 4.45 mmol) in anhydrous THF (40 ml), ^{n}-BuLi (2 M in cyclohexane, 2.45 mL, 4.90 mmol) was added at -40 °C under Argon atmosphere and then the mixture was cooled to -78 °C and stirred for 1 hour. Then CO_2 gas was bubbled into the solution for 2 h. The reaction mixture was quenched by 2.0 M aqueous $KHSO_4$ solution, warmed to rt, diluted with water, and extracted with ethyl acetate. The crude mixture was diluted with toluene poured onto a pad of SiO_2 and eluted with ethyl acetate. The filtrate was concentrated to afford 3-(triisopropylsilyl)propiolic acid which was further used for the next step.

Step II: To a stirred solution of 3-(triisopropylsilyl)propiolic acid (0.5 gm, 2.21 mmol) in DCM (10 ml), ^{t}-BuOH (0.251 mL, 2.65 mmol), DCC (0.501 gm, 2.43 mmol) and DMAP (0.027 gm, 0.221 mmol) was added at 0 °C. Then the resulting mixture was stirred for 12 h at room temperature. After that, the mixture was filtered, the filtrate was diluted with DCM and washed with water. The organic layer was dried over anhydrous Na_2SO_4 and concentrated in *vacuo*. To afford the pure product, the crude reaction mixture was purified over silica gel column chromatography using ethyl acetate/hexane as eluent.

Ethyl 3-(triisopropylsilyl)propiolate: ^{1}H NMR (400 MHz, $CDCl_3$) δ 4.23 (q, *J* = 6.8 Hz, 2H), 1.31 (t, *J* = 6.8 Hz, 3H), 1.11 (s, 21H); ^{13}C NMR (100 MHz, CDCl3) δ 153.3, 97.0, 90.9, 62.1, 18.6, 14.2, 11.1.

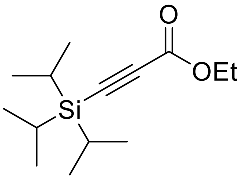

HRMS (ESI): Calcd for $C_{14}H_{26}O_2Si$ $[M+Na]^+$: 277.1600; found: 277.1588.

Ethyl 3-(trimethylsilyl)propiolate: ^{1}H NMR (400 MHz, $CDCl_3$) δ 4.21 (q, *J* = 7.2 Hz, 2H), 1.29 (t, *J* = 7.2 Hz, 3H), 0.23 (s, 9H); ^{13}C NMR (100 MHz, $CDCl_3$) δ 153.2, 94.8, 93.7, 62.1, 14.1, -0.7.

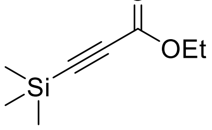

HRMS (ESI): Calcd for $C_8H_{14}O_2Si$ $[M+H]^+$: 171.0841; found: 171.0844.

Ethyl 3-(triethylsilyl)propiolate: ^{1}H NMR (400 MHz, $CDCl_3$) δ 4.23 (q, *J* = 7.2 Hz, 2H), 1.31 (t, *J* = 7.2 Hz, 3H), 1.01 (t, *J* = 8.0 Hz, 9H), 0.68 (q, *J* = 8.0 Hz, 6H); ^{13}C NMR (100 MHz, $CDCl_3$) δ 153.2, 96.1, 91.9, 62.1, 14.2, 7.4, 3.9.

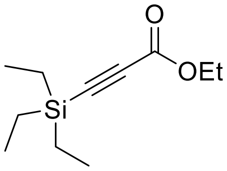

HRMS (ESI): Calcd for $C_{11}H_{20}O_2Si$ $[M+H]^+$: 213.1311; found: 213.1301.

Methyl 3-(triisopropylsilyl)propiolate: ^{1}H NMR (400 MHz, $CDCl_3$) δ 3.76 (s, 3H), 1.11 – 1.08 (m, 21H); ^{13}C NMR (100 MHz, $CDCl_3$) δ 153.6, 96.6, 91.6, 52.7, 18.6, 11.1.

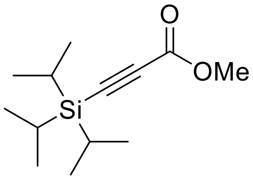

HRMS (ESI): Calcd for $C_{13}H_{24}O_2Si$ $[M+H]^+$: 241.1624; found: 241.1629.

Tert-butyl 3-(triisopropylsilyl)propiolate: ^{1}H NMR (400 MHz, $CDCl_3$) δ 1.49 (s, 9H), 1.11 – 1.08 (m, 21H); ^{13}C NMR (100 MHz, $CDCl_3$) δ 152.4, 98.5, 88.2, 83.3, 28.1, 18.6, 11.2;

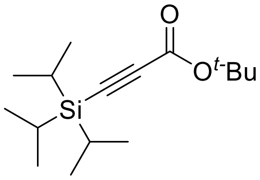

HRMS (ESI): Calcd for $C_{16}H_{30}O_2Si$ $[M+H]^+$: 283.2093; found: 283.2086.

Phenyl 3-(triisopropylsilyl)propiolate: ^{1}H NMR (400 MHz, $CDCl_3$) δ 7.42 – 7.36 (m, 2H), 7.28 – 7.23 (m, 1H), 7.20 – 7.12 (m, 2H), 1.18 – 1.08 (m, 21H); ^{13}C NMR (100 MHz, $CDCl_3$) δ 151.3, 150.3, 129.7, 126.5, 121.6, 96.1, 94.7, 18.6, 11.1.

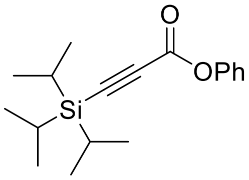

HRMS (ESI): Calcd for $C_{18}H_{26}O_2Si$ $[M+H]^+$: 303.1780; found: 303.1777.

General procedure for amine addition to β-silyl alkynoates:

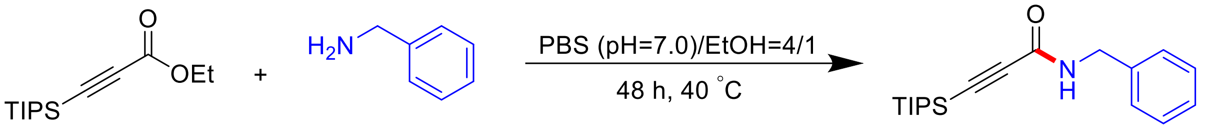

1a **2a** **3a**

To a 4 ml glass vial, 0.1 mL ethanol was added to the mixture of ethyl 3-(triisopropylsilyl) propiolate (**1a**, 50 mg, 0.196 mmol) and benzylamine (**2a**, 0.295 mmol). The mixture was stirred for 5 minutes to achieve a homogeneous solution. Subsequently, 0.4 mL of pH-neutral phosphate buffer was added, and the resulting mixture was vigorously stirred for 48 hours at 40°C. The mixture was diluted with ethyl acetate, washed with water, and dried over anhydrous sodium sulfate. The crude mixture was purified over silica gel column chromatography and 30% ethyl acetate/hexane was used as an eluent to afford the product (**3a**).

General Procedure for desilylation of propiolamide:

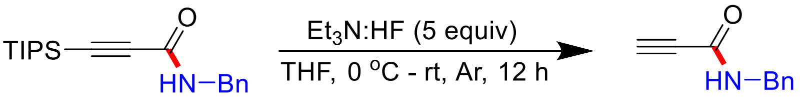

3a 91 %, **5a**

To a 10 mL round bottom flask, **3a** (60 mg, 0.190 mmol) and triethylamine trihydrofluoride (5 equiv.) were taken in 2 mL THF at 0°C under an argon atmosphere. Then the mixture was vigorously stirred for 12 hours at room temperature. After that, the mixture was washed with water, extracted with ethyl acetate, and dried over anhydrous sodium sulfate, concentrated under reduced pressure. The yield of **5a** was calculated after purification of the crude mixture using silica gel flash column chromatography.

Optimization of Reaction Conditions[a].

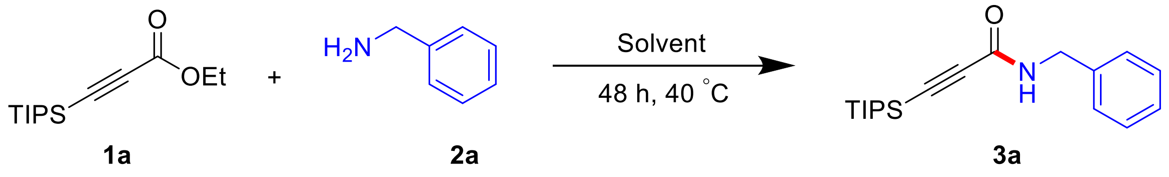

Entry	Solvent	pH value	Temperature (°C)	3aa (%)[b]
1	H_2O	7	37	56
2	Sodium acetate buffer	5.2	37	18
3	PBS buffer (pH=6.0)	6	37	30
4	PBS buffer (pH=7.0)	7	37	80
5	PBS buffer (pH=8.0)	8	37	54
6	PBS buffer (pH=9.0)	9	37	52
7	Tris-HCl Buffer (pH=8.0)	8	37	40
8	Tris-HCl Buffer (pH=9.22)	9.22	37	37
9	CH_3CN	---	37	17
10	DMSO	---	37	39
11	DMF	---	37	19
12	THF	---	37	14
13	EtOH	---	37	58
14	DCM	---	37	18
15	H_2O: EtOH (4:1)	---	37	84
16	H_2O: EtOH (2:1)	---	37	62
17	PBS (pH=7.0)/THF	7	37	75
18	PBS (pH=7.0)/CH_3CN	7	37	46
19	PBS (pH=7.0)/DMSO	7	37	48
20	PBS (pH=7.0)/DMF	7	37	30
21	PBS (pH=7.0)/EtOH	7	37	85
**22**	**PBS (pH=7.0)/EtOH**	**7**	**40**	**92 (88)[c]**
23	PBS (pH=7.0)/EtOH	7	40	86[d]
24	PBS (pH=7.0)/EtOH	7	40	89[e]

Table S1. Optimization of reaction condition[a]: Ethyl 3-(triisopropylsilyl)propiolate **1a** (50 mg, 0.196 mmol), **2a** (0.295 mmol, 1.5 equiv.), solvent (0.5 mL) for the indicated time; [b]yield was determined by ^1H NMR using CH_2Br_2 as an internal standard; [c]isolated yield; [d]reaction

was performed in a plastic vial. [e]used **1a** was synthesized from the Grignard method. PBS buffer: Phosphate saline buffer (pH=7); PBS/Organic solvent = 4/1.

Reaction time optimization.

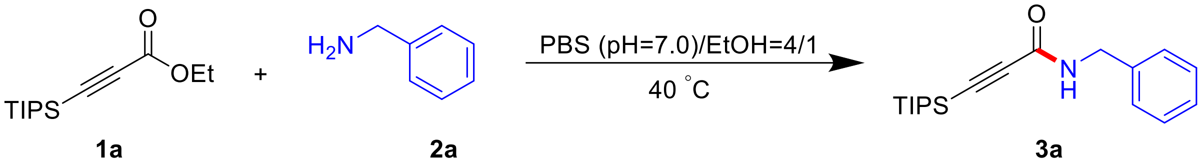

Entry	Time (Hours)	Yield (%)[b]
1	12 h	38
2	24 h	55
3	36 h	75
**4**	**48 h**	**92**

Table S2. Reaction time optimization[a]: Ethyl 3-(triisopropylsilyl)propiolate **1a** (50 mg, 0.196 mmol), benzylamine **2a** (0.295 mmol, 1.5 equiv.), PBS buffer (pH=7.0)/EtOH (4:1) = 0.5 ml at 40 ºC for the indicated time. [b]Yield was determined by ^{1}H NMR using CH_2Br_2 as an internal standard.

Reaction temperature optimization

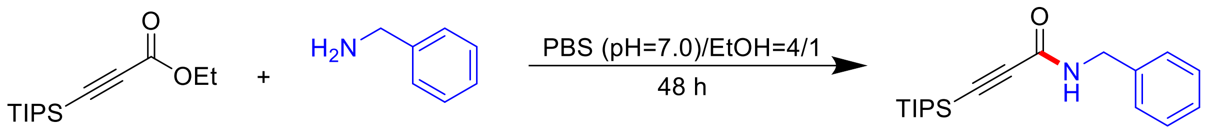

Entry	Temperature (°C)	Time	Yield (%)[b]
1	25 °C	48 h	40
2	37 °C	48 h	85
**3**	**40 °C**	**48 h**	**92**
4	80 °C	24 h	90

Table S3. Reaction temperature optimization[a]: Ethyl 3-(triisopropylsilyl)propiolate **1a** (50 mg, 0.196 mmol), benzylamine **2a** (0.295 mmol, 1.5 equiv.), PBS buffer (pH=7.0)/EtOH (4:1) = 0.5 ml. [b]Yield was determined by ^{1}H NMR using CH_2Br_2 as an internal standard.

Reactivity comparison with different ester groups.

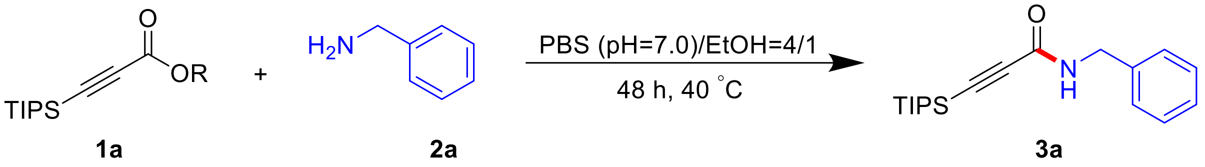

Entry	R-group	3aa (%)[b]
1	Methyl ester	86 %
2	Ethyl ester	92 %
3	Benzyl ester	88 %
4	Tert-butyl ester	0 %

Table S4. Reactivity comparison with different ester groups[a]: Ethyl 3-(triisopropylsilyl)propiolate **1a** (50 mg, 0.196 mmol), benzylamine **2a** (0.295 mmol, 1.5 equiv.), PBS buffer (pH=7.0)/EtOH (4:1) = 0.5 ml. [b]Yield was determined by ^{1}H NMR using CH_2Br_2 as an internal standard.

Regio-selectivity of the reaction using a different protecting group[a].

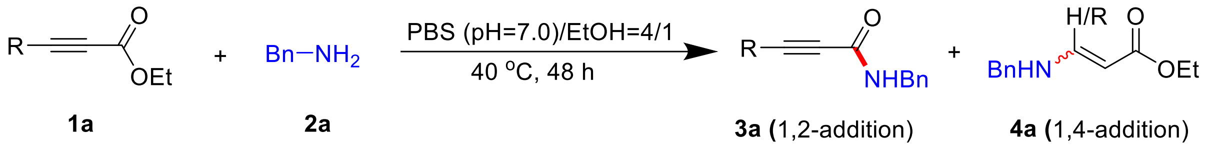

Entry	R-group	1,2-addition (%) [b]	1,4-addition (%) [b]
1	Ph	39	45
2	TMS[c]	0	66
3	TES[c]	12	55
4	TIPS	92	0

Table S5. Regio-selectivity of the reaction using a different protecting group[a]: **1a** (50 mg, 0.196 mmol), benzylamine **2a** (0.295 mmol, 1.5 equiv.), PBS buffer (pH=7.0)/EtOH (4:1) = 0.5 ml. [b]Yield was determined from the crude mixture by ^{1}H NMR using CH_2Br_2 as an internal standard. [c]The crude complex mixture may contain other possible byproducts.

ICP-MS for the Determination of the Metal Ions.

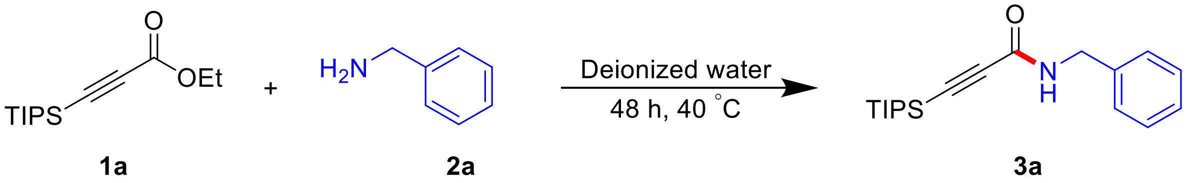

General procedure for ICP-MS experiments: In a 4ml glass vial, **1a** (60 mg, 0.236 mmol), and **2a** (0.354 mmol) were taken in 0.8 mL Deionized water. Then the reaction mixture was vigorously stirred at 40 °C for 48 h. After that, a 0.3 mL aliquot of the reaction mixture was placed to evaluate ICP-MS.

We have used the Agilent Technologies 7700 Series ICP-MS instrument to determine the metal concentration in the reaction mixture.

Sample preparation: 0.3 mL aliquot of reaction mixture was diluted by adding 4.7 mL of 3% HNO_3 matrix solution in water. Then we record the metal concentration in the reaction mixture. This experiment shows that the metal is present at the PPB level.

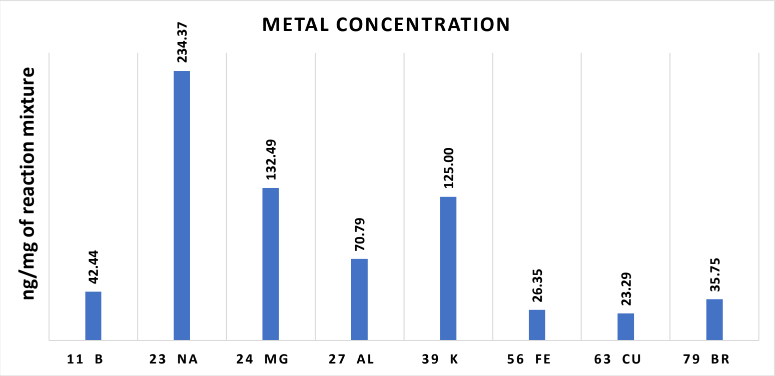

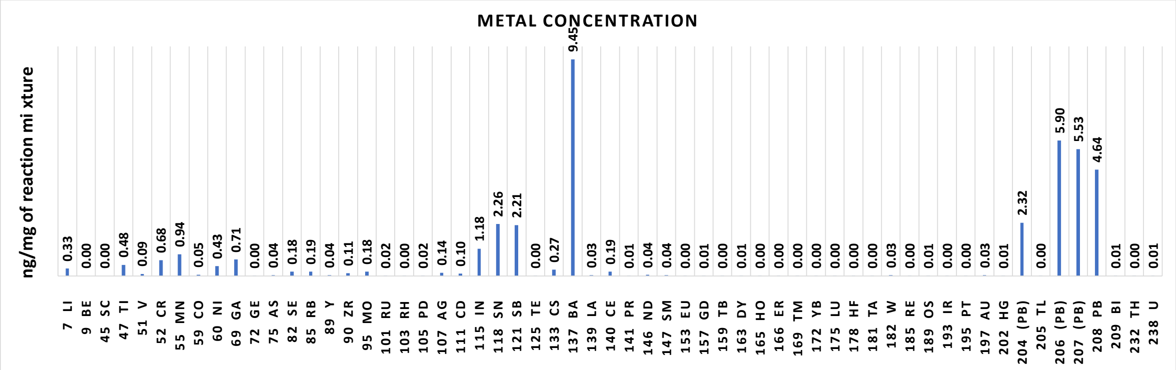

Fig S1. ICP-MS plot for metal concentration in the reaction mixture.

Dipeptide Synthesis and Epimerization Experiment.

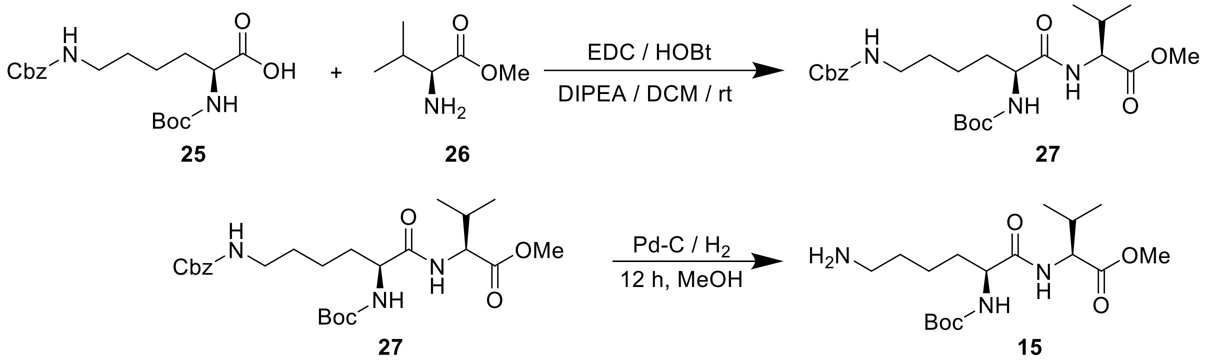

General procedure:

Step I: To a stirred solution of Boc-Lys(Z)-OH (0.5 gm, 1.315 mmol) and L-Valine methyl ester hydrochloride (0.242 gm, 1.447 mmol) in Dry CH_2Cl_2 (10 ml), 1-Ethyl-3-(3-dimethylaminopropyl) carbodiimide (EDC) (1.97 mmol), Hydroxybenzotriazole (HOBt) (1.97 mmol) and *N,N*-Diisopropylethylamine (DIPEA) (2.631 mmol) was added at room temperature. The resulting solution was stirred for 12 hours. Then the mixture was diluted with CH_2Cl_2 and washed with water. The combined organic was separated, dried over anhydrous Na_2SO_4, and concentrated under reduced pressure. The crude mixture was purified over silica gel flash column chromatography to afford the product **27**.

Step II: To a stirred solution of **27** (0.5 gm, 1.013 mmol) in methanol, 5 wt% of Pd-C was added in Hydrogen atmosphere (H_2 balloon). The resulting solution was stirred for 12 h at room temperature. Then the mixture was filtered through a pad of celite, the filtrate was dried to afford the pure product of **15**.

Exploring Epimerization at Chiral Centers Following Biocompatible Amide Bond Formation Reaction.

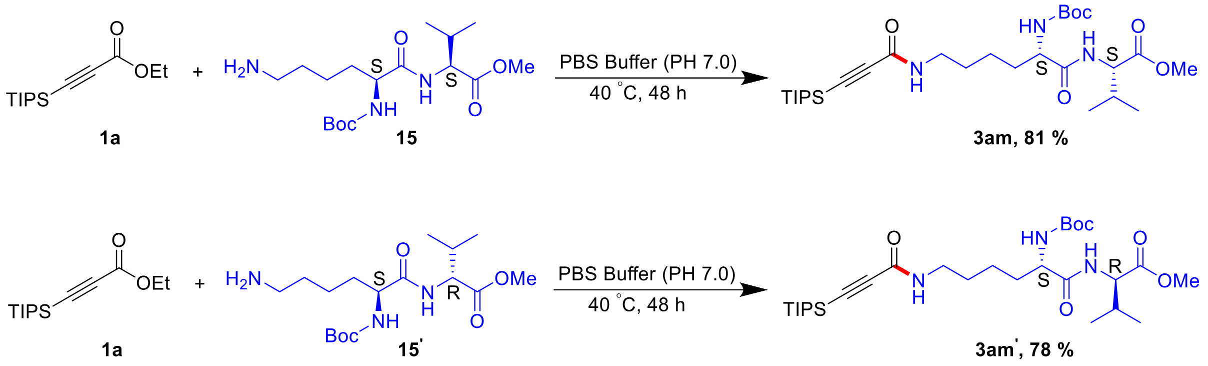

General procedure: To a 4 ml glass vial, ethyl 3-(triisopropylsilyl)propiolate (**1a**, 60 mg, 0.236 mmol) and **15** (127 mg, 0.354 mmol) was taken. Then 0.5 mL of pH-neutral phosphate buffer was added. The resulting solution was vigorously stirred for 48 h at 40 ºC. The mixture was diluted with ethyl acetate, washed with water, and dried over anhydrous sodium sulfate. The crude mixture was purified over silica gel column chromatography, and hexane/ethyl acetate was used as an eluent to afford the pure product methyl *N*2-(tert-butoxycarbonyl)-*N*6-(3-(triisopropylsilyl)propioloyl)-*L*-lysyl-*L*-valinate (**3am**).

Following the general procedure we have preparing the methyl *N*2-(tert-butoxycarbonyl)-*N*6-(3-(triisopropylsilyl)propioloyl)-*L*-lysyl-*L*-valinate (**3am**) and methyl methyl *N*2-(tert-butoxycarbonyl)-*N*6-(3-(triisopropylsilyl)propioloyl)-*L*-lysyl-*D*-valinate (**3am'**). Then, we made the stuck plot of ^{13}C NMR spectra of **3am** and **3am'**. The spectra clearly show that no carbon atom peak is getting overlaps of these two compounds. From the spectra, we conclude that no epimerization was observed at the chiral center of the silyl alkynyl amide products (**3am**, **3am'**).

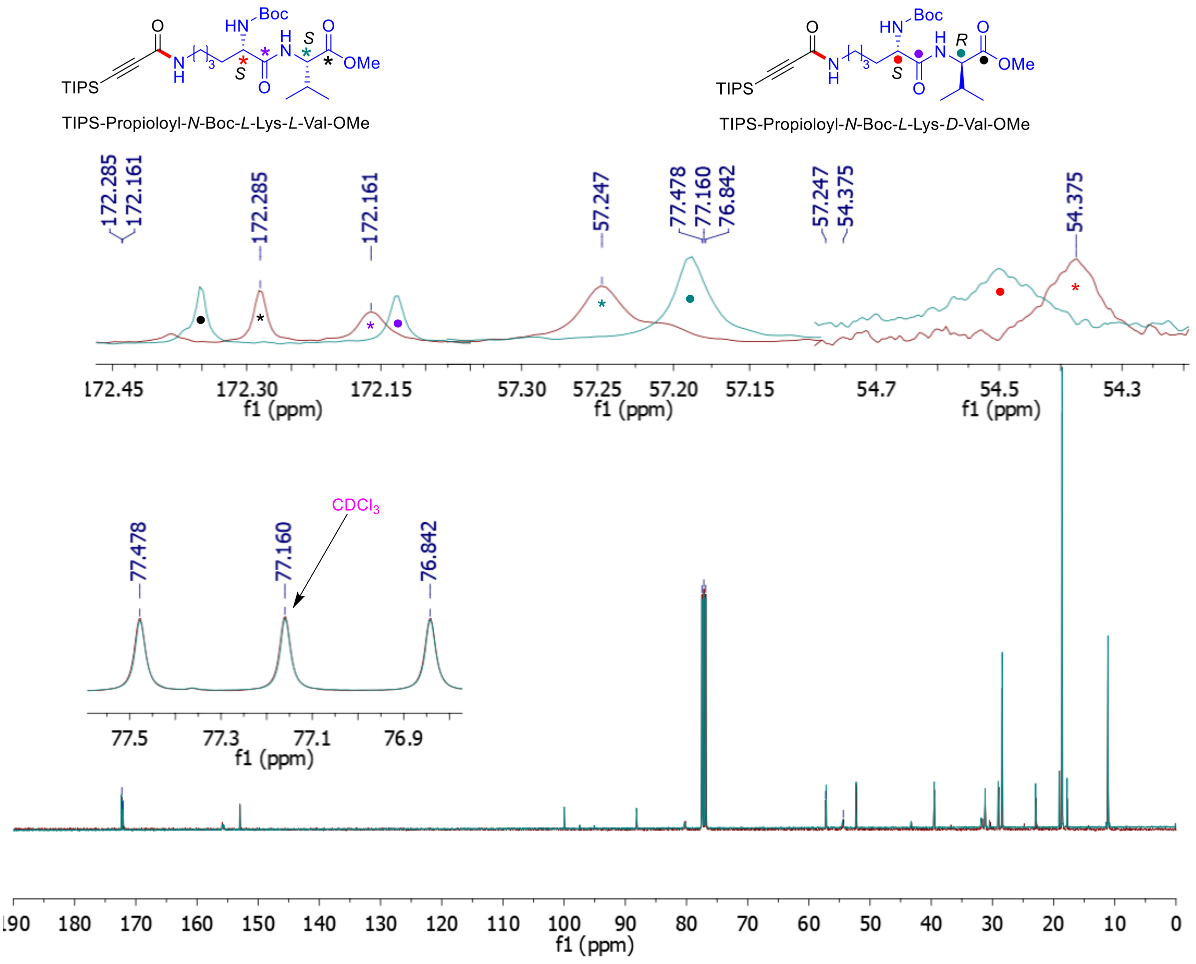

Fig S2. ^{13}C NMR stuck plot of **3am** and **3am'** to determine the epimerization at the chiral centre.

Exploring Epimerization at Chiral Centers after desilylation of the alkynyl amide.

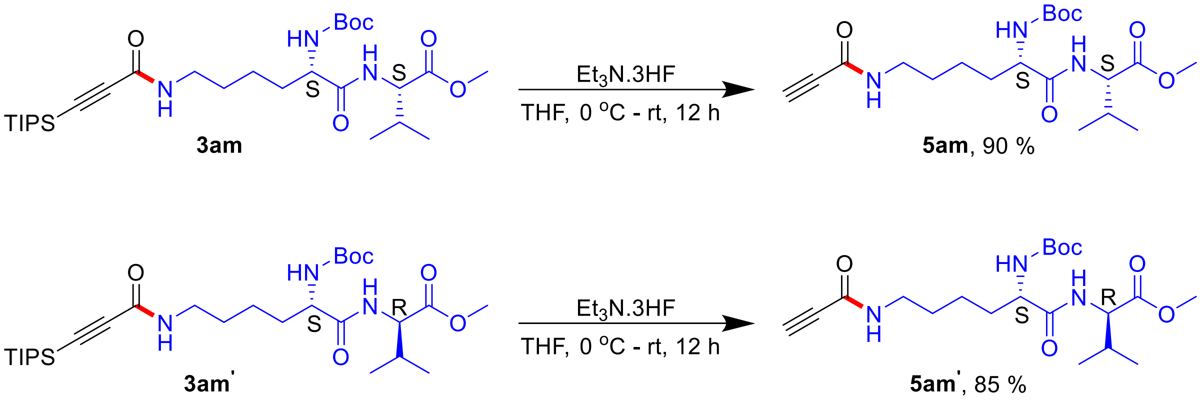

To a 10 mL round bottom flask, **3am** (60 mg, 0.105 mmol) and triethylamine trihydrofluoride (5 equiv.) were taken in 2 mL THF at 0°C. Then the mixture was vigorously stirred for 12 hours at room temperature under an argon atmosphere. After that, the organic layer was washed with water, extracted with ethyl acetate, and dried over anhydrous sodium sulfate, concentrated under reduced pressure. The crude mixture was purified over silica gel column chromatography to afford the pure product **5am**.

Following the general procedure, we have prepared the methyl *N*2-(tert-butoxycarbonyl)-*N*6-propioloyl-*L*-lysyl-*L*-valinate (**5am**) and methyl *N*2-(tert-butoxycarbonyl)-*N*6-propioloyl-*L*-lysyl-*D*-valinate (**5am'**). Then, we made the stuck plot of ^{13}C NMR spectra of **5am** and **5am'**. From the spectra, it was visible that no carbon atom is getting overlaps of these two compounds. After the desilylation step, no epimerization was observed at the chiral canter of the desilylated alkynyl amide.

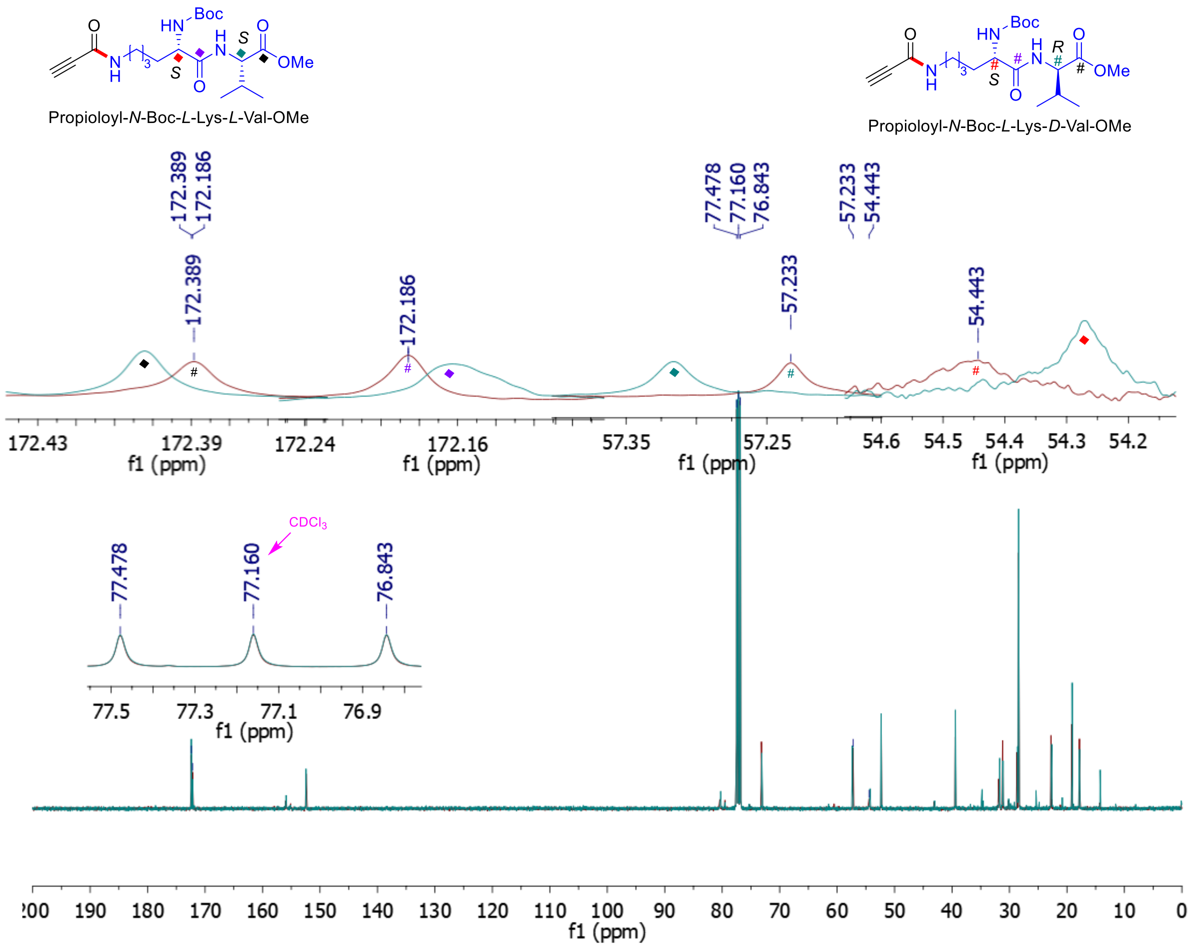

Fig S3. ^{13}C NMR stuck plot of **5am** and **5am'** to determine the epimerization at the chiral center.

Gram Scale Synthesis and Product Derivatization.

Gram Scale Synthesis.

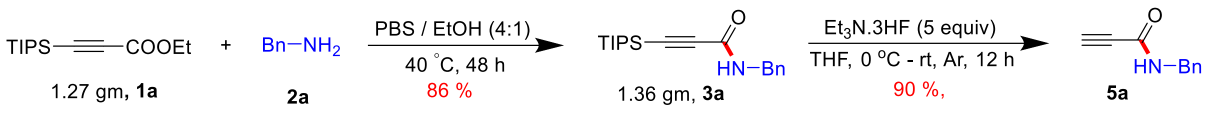

Step I: In a 50 mL round bottom flask, **1a** (1.27 gm, 5 mmol) and **2a** (0.82 ml, 7.5 mmol) were taken in 3 mL ethanol. Then, the solution was stirred for 5 minutes to achieve a homogeneous solution. Subsequently, 12 mL of pH-neutral phosphate buffer was added, and the resulting mixture was vigorously stirred at 40°C for 48 hours. After that, the mixture was settled down in a cold place to complete the precipitation of the product. The mixture was filtered, and the residue was washed with water and dried under vacuum to afford the pure product of **3a** (1.36 gm).

Step II: In a 50 mL round bottom flask, **3a** (1.36 gm, 4.3 mmol) and $Et_3N.3HF$ (5 equiv.) were taken in 15 ml THF at 0 °C in an argon atmosphere. The mixture was vigorously stirred for 12 hours at room temperature under an argon atmosphere. After that, the mixture was washed with water, extracted with ethyl acetate, and dried over anhydrous sodium sulfate, concentrated under reduced pressure. The yield of **5a** was calculated after purification of the crude mixture using silica gel flash column chromatography.

Derivatization of *N*-Benzylpropiolamide:

Biocompatible C-N bond formation reaction.

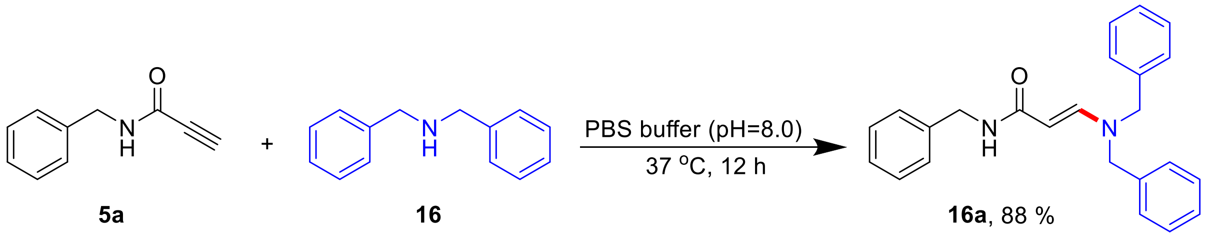

Procedure: To a 4 ml glass vial, **5a** (50 mg, 0.314 mmol) and **16** (0.377 mmol) were taken. Then add 1 mL PBS buffer (pH=8.0) to the mixture. The resulting solution was stirred at 40 °C for 12 hours. After that, the mixture was diluted with ethyl acetate, washed with water, and dried over anhydrous sodium sulfate, concentrated under reduced pressure. The crude mixture was washed with cold hexane to afford the pure product **16a** in 88% yield.

(*E*)-*N*-benzyl-3-(dibenzylamino)acrylamide (16a). White solid, yield 88% (99 mg); ^{1}H NMR

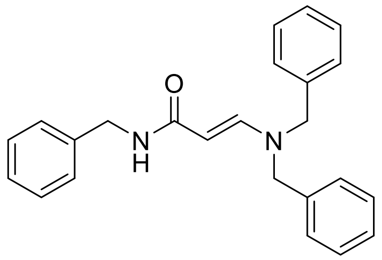

(400 MHz, $CDCl_3$) δ 7.85 (d, *J* = 12.8 Hz, 1H), 7.36 – 7.23 (m, 11H), 7.17 (d, *J* = 6.8 Hz, 4H), 5.34 (s, 1H), 4.68 (d, *J* = 12.8 Hz, 1H), 4.47 (d, *J* = 5.6 Hz, 2H), 4.27 (s, 4H); 13C NMR (100 MHz, $CDCl_3$) δ 168.9, 150.5, 139.5, 136.4, 128.8, 128.6, 127.9, 127.7, 127.5, 127.2, 88.4, 43.6; HRMS (ESI): Calcd for $C_{24}H_{24}N_2O$ $[M+H]^+$: 357.1967; found: 357.1973.

Sonogashira coupling reaction for the synthesis of *N*-benzyl-3-phenylpropiolamide.

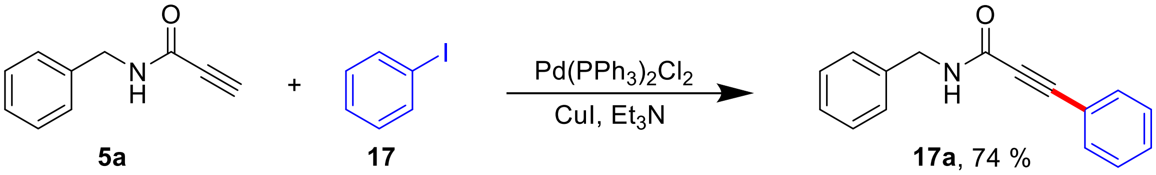

5a **17** **17a**, 74 %

Procedure: In an oven-dried seal tube, **5a** (60 mg, 0.377 mmol), **17** (0.566 mmol), $Pd(PPh_3)_2Cl_2$ (0.018 mmol), and CuI (0.037 mmol) were taken in triethylamine (1 mL). The resulting solution was refluxed at 70 ^{0}C for 6 h. Then the mixture was diluted with ethyl acetate and washed with a brine solution, the organic layer was separated, dried over anhydrous Na_2SO_4, and concentrated under reduced pressure. The crude mixture was separated over silica gel flash column chromatography to afford pure **17a** in 74% yield.

***N*-benzyl-3-phenylpropiolamide (17a):** Off white solid, yield 74% (66 mg); ^{1}H NMR (400 MHz, $CDCl_3$) δ 7.52 (d, *J* = 7.6 Hz, 2H), 7.43 – 7.31 (m, 8H), 6.19 (s, 1H), 4.71 (d, *J* = 6.4 Hz, 2H, rotamer), 4.55 (d, *J* = 5.8 Hz, 2H); ^{13}C NMR (100 MHz, $CDCl_3$) δ 153.4, 137.4, 132.7, 130.3, 129.0, 128.7, 128.1, 128.0, 120.3, 85.3, 83.0, 44.2; HRMS (ESI): Calcd for $C_{16}H_{13}NO$ $[M+H]^+$: 236.1075; found: 236.1065.

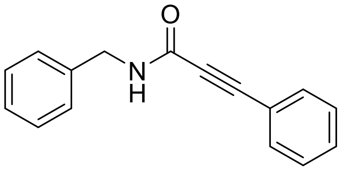

Synthesis of 5-iodo-1,2,3-triazoles compound *via* click reaction.

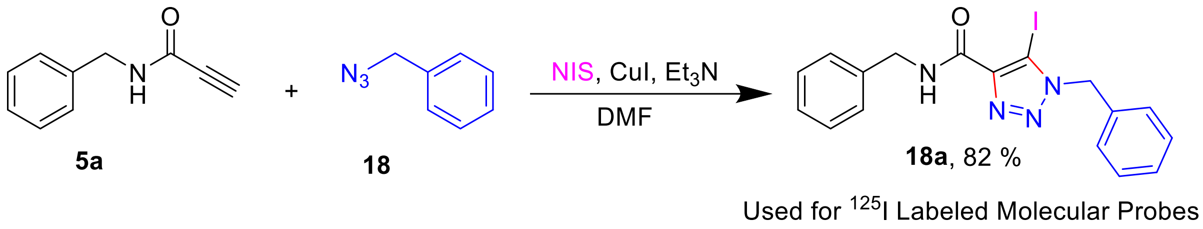

Procedure: To a stirred solution of **5a** (65 gm, 0.408 mmol) and **18** (0.45 mmol) in dry DMF, were added *N*-iodosuccinimide (0.490 mmol), copper (I) Iodide (0.4 mmol) and triethylamine (0.082 mmol). The resulting solution was stirred at room temperature under an argon atmosphere for 4 h. After that, the mixture was diluted with ethyl acetate and washed with water, and the organic layer was separated and dried over anhydrous Na_2SO_4, concentrated under reduced pressure. The crude mixture was purified by silica gel flash column chromatography to afford **18a** in 82% yield.

***N*,1-dibenzyl-5-iodo-1H-1,2,3-triazole-4-carboxamide (18a):** Brown solid, yield 82% (140 mg); ^{1}H NMR (400 MHz, $CDCl_3$) δ 7.52 (s, 1H), 7.36 – 7.25 (m, 10H), 5.64 (s, 2H), 4.63 (d, J = 6.0 Hz, 2H); ^{13}C NMR (100 MHz, $CDCl_3$) δ 159.5, 143.3, 137.9, 133.8, 129.1, 128.9, 128.1, 128.0, 127.7, 81.4, 54.4, 43.3; HRMS (ESI): Calcd for $C_{17}H_{15}N_4OI$ $[M+Na]^+$: 441.0188; found: 441.0197.

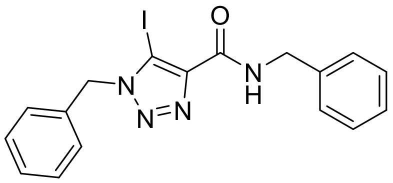

Reduction of *N*-benzylpropiolamide.

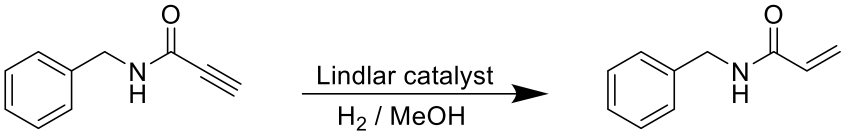

5a **19a**, 86 %

Procedure: To a Schlenk tube equipped with a magnetic stir bar **5a** (70 mg, 0.440 mmol), Lindlar catalyst (10 wt.%) in methanol under H_2 atmosphere (H_2 Balloon). Then the reaction was stirred for 18 h at room temperature. After that, the mixture was filtered through a pad of celite, and the filtrate was dried to afford the pure product of **19a** in 86% yield.

***N*-benzylacrylamide (19a):** White solid, yield 86% (61 mg); ^{1}H NMR (400 MHz, $CDCl_3$) δ 7.38 – 7.26 (m, 5H), 6.32 (d, *J* = 17.0 Hz, 1H), 6.11 (dd, *J* = 17.0, 10.2 Hz, 1H), 5.95 (s, 1H), 5.66 (d, *J* = 10.2 Hz, 1H), 4.51 (d, *J* = 5.6 Hz, 2H); ^{13}C NMR (100 MHz, $CDCl_3$) δ 165.5, 138.2, 130.8, 128.9, 128.1, 127.7, 126.9, 43.8; HRMS (ESI): Calcd for $C_{10}H_{11}NO$ $[M+H]^+$: 162.0919; found: 162.0925.

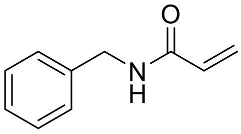

Linker Application: Conjugation between Drug and Peptide.

Biocompatible C-S bond formation.

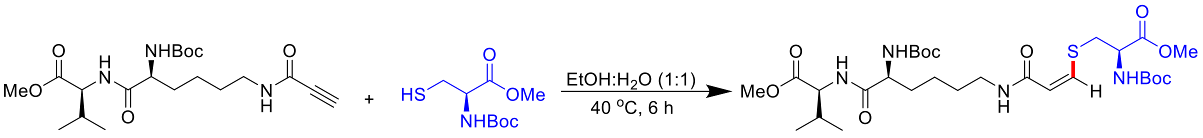

5am **20** **20a**

Procedure: To a 4 ml glass vial, **5am** (45 mg, 0.109 mmol) and **20** (0.1.32 mmol) were taken in 0.4 mL ethanol. Subsequently, add 0.4 ml of water to the mixture. Then the resulting solution was stirred at 40 °C for 6 hours. After that, the mixture was diluted with ethyl acetate, washed with water, and dried over anhydrous sodium sulfate. The crude mixture was purified over silica gel column chromatography to yield a pure product of **20a** in 78%.

Methyl (6*R*,17*S*,20*S*,*Z*)-17-((tert-butoxycarbonyl)amino)-20-isopropyl-6-(methoxycarbonyl)-2,2-dimethyl-4,11,18-trioxo-3-oxa-8-thia-5,12,19-triazahenicos-9-en-21-oate (20a). Semi-solid, yield 78% (55 mg); ^{1}H NMR (400 MHz, $CDCl_3$) δ 6.71 (d, *J* = 9.8 Hz, 2H), 5.96 (s, 1H), 5.77 (d, *J* = 9.8 Hz, 1H), 5.46 (d, *J* = 6.8 Hz, 1H), 5.14 (s, 1H), 4.56 (d, *J* = 6.8 Hz, 1H), 4.48 (dd, *J* = 8.4, 5.2 Hz, 1H), 4.10 (dd, *J* = 14.4, 7.2 Hz, 1H), 3.73 (s, 3H), 3.72 (s, 3H), 3.31 – 3.25 (m, 2H), 3.18 (s, 2H), 2.21 – 2.11 (m, 1H), 1.96 (s, 1H), 1.87 – 1.76 (m, 1H), 1.67 – 1.57 (m, 1H), 1.56 – 1.49 (m, 2H), 1.42 (s, 18H), 1.28 – 1.21 (m, 1H), 0.90 (dd, *J* = 9.6, 7.2 Hz, 6H); ^{13}C NMR (100 MHz, $CDCl_3$) δ 172.4, 172.2, 170.9, 166.4, 155.9, 155.2, 144.4, 116.4, 80.3, 80.2, 57.3, 54.4, 53.9, 52.8, 52.3, 38.8, 38.7, 31.1, 28.9, 28.4, 28.4, 22.8, 19.1, 17.8. HRMS (ESI): Calcd for $C_{29}H_{50}N_4O_{10}S$ $[M+H]^+$: 647.3326; found: 647.3334.

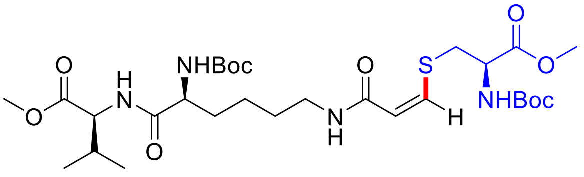

Captopril Drug Modification:

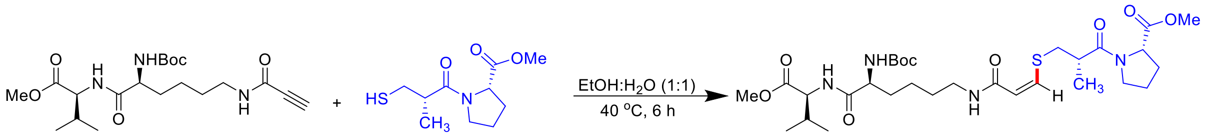

5am 21 21a

Procedure: **5am** (45 mg, 0.109 mmol) and **21** (0.1.32 mmol) were taken in a 4 mL glass vial. Subsequently, water: ethanol (1:1) mixture of solvent 0.8 mL was added to the vial. Then the resulting solution was stirred at 40 °C for 6 hours. After that, the mixture was diluted with ethyl acetate, washed with water, and dried over anhydrous sodium sulfate. The crude mixture was purified over silica gel column chromatography to yield a pure product of **21a** in 84%.

Methyl ((6*S*,17*S*,*Z*)-6-(((*S*)-1-methoxy-3-methyl-1-oxobutan-2-yl)carbamoyl)-2,2,17-trimethyl-4,12-dioxo-3-oxa-15-thia-5,11-diazaoctadec-13-en-18-oyl)-*L*-prolinate (21a).

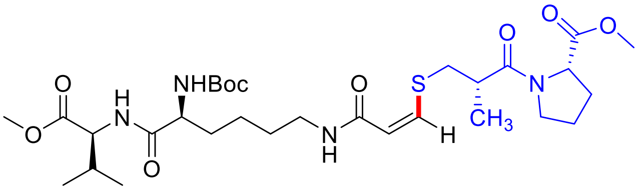

Semi-solid, yield 84% (59 mg); ^{1}H NMR (400 MHz, $CDCl_3$) δ 6.85 (d, *J* = 10.0 Hz, 1H), 6.67 (s, 1H), 5.96 (s, 1H), 5.72 (d, *J* = 10.0 Hz, 1H), 5.15 (s, 1H), 4.51 – 4.41 (m, 2H), 4.08 (d, *J* = 6.4 Hz, 1H), 3.71 (s, 3H), 3.69 (s, 3H), 3.66 – 3.58 (m, 2H), 3.32 – 3.20 (m, 2H), 3.11 – 3.01 (m, 1H), 2.89 – 2.80 (m, 1H), 2.75 – 2.67 (m, 1H), 2.21 – 2.10 (m, 3H), 2.05 – 1.92 (m, 3H), 1.83 – 1.75 (m, 1H), 1.64 – 1.49 (m, 4H), 1.42 (s, 9H), 1.21 (d, *J* = 6.8 Hz, 3H), 0.93 – 0.87 (m, 6H); ^{13}C NMR (100 MHz, $CDCl_3$) δ 173.5, 172.8, 172.4, 172.2, 166.6, 155.8, 145.7, 115.4, 80.2, 58.8, 57.3, 52.3, 52.3, 47.1, 39.9, 39.9, 38.8, 31.7, 31.1, 29.1, 29.1, 28.4, 24.8, 22.8, 19.1, 17.8, 17.5; HRMS (ESI): Calcd for $C_{30}H_{50}N_4O_9S$ $[M+H]^+$: 643.3377; found: 643.3377.

Zidovudine Drug Modification:

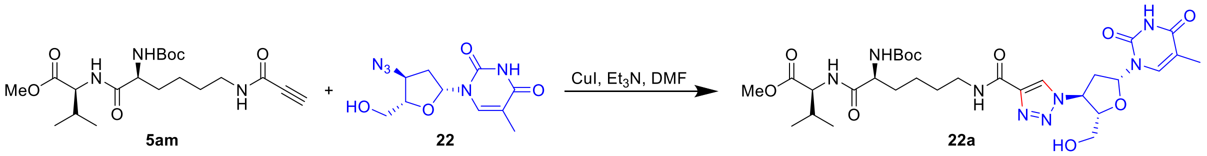

Procedure: In a Schlenk tube, **5am** (45 mg, 0.109 mmol), Zidovudine (0.120 mmol), copper iodide (0.109 mmol), and triethylamine (0.109 mmol) were taken in dry DMF. The resulting solution was stirred at room temperature under an argon atmosphere for 4 hours. After that, the mixture was diluted with ethyl acetate and washed with water (4 times), the organic layer was separated and dried over anhydrous Na_2SO_4, concentrated under reduced pressure. The crude mixture was purified over silica gel flash column chromatography to afford the pure product **22a** in 61% yield.

Methyl *N*2-(tert-butoxycarbonyl)-*N*6-(1-((2*S*,3*S*,5*R*)-2-(hydroxymethyl)-5-(5-methyl-2,4-dioxo-3,4-dihydropyrimidin-1(2H)-yl)tetrahydrofuran-3-yl)-1H-1,2,3-triazole-4-carbonyl)-*L*-lysyl-*L*-valinate (22a). White solid, yield 61% (45 mg); ^{1}H NMR (400 MHz,

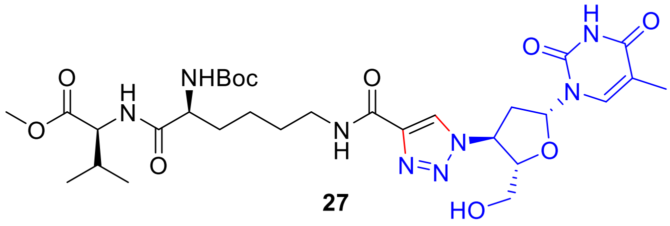

27

$CDCl_3$) δ 9.04 (s, 1H), 7.46 (s, 1H), 7.43 – 7.35 (m, 1H), 6.79 (d, *J* = 7.6 Hz, 1H), 6.34 (t, *J* = 6.8 Hz, 1H), 5.62 – 5.48 (m, 1H), 5.17 (d, *J* = 7.2 Hz, 1H), 4.53 (dd, *J* = 8.8, 5.2 Hz, 1H), 4.47 – 4.42 (m, 1H), 4.18 – 4.09 (m, 1H), 4.02 (d, *J* = 10.4 Hz, 1H), 3.87 (d, *J* = 10.4 Hz, 1H), 3.73 (s, 3H), 3.44 (dd, *J* = 13.2, 6.8 Hz, 2H), 3.12 – 2.99 (m, 1H), 2.93 - 2.85 (m,1H), 2.22 – 2.13 (m, 1H), 1.94 (s, 3H), 1.91 – 1.80 (m, 2H), 1.74 – 1.56 (m, 4H), 1.43 (s, 9H), 1.30 – 1.23 (m, 3H), 0.91 (dd, *J* = 9.6, 6.8 Hz, 6H). ^{13}C NMR (100 MHz, $CDCl_3$) δ 172.3, 172.2, 163.6, 159.4, 159.3, 155.8, 150.5, 143.2, 138.0, 111.5, 89.6, 85.5, 82.1, 62.3, 60.6, 57.1, 52.2, 38.8, 37.1, 31.2, 29.7, 29.2, 28.3, 22.9, 18.9, 17.7, 12.5; HRMS (ESI): Calcd for $C_{30}H_{46}N_8O_{10}$ $[M+H]^+$: 679.3415; found: 679.3412.

Selectivity comparison between activated ethyl alkynoates and *β*-silyl alkynoates.

Reaction of activated ethyl alkynoates with amino esters:

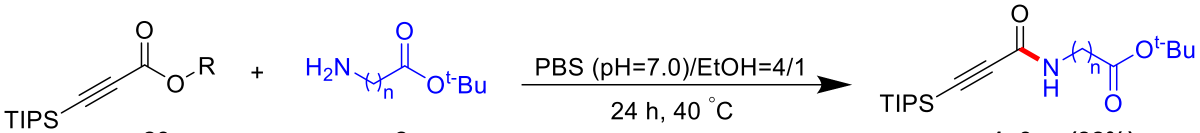

23 **2** **n = 1, 3ao (82%)** **n = 2, 3ap (81%)**

23a = R = -4,$NO_2C_6H_4$
23b = R = -NHS
23c = R = -2,4,5-$Cl_3C_6H_2$

General Procedure: To a 4 ml glass vial, 0.1 mL ethanol was added to the mixture of NHS alkynoates (**23**, 50 mg, 0.154 mmol) and the corresponding amino ester (**2**, 0.232 mmol). The mixture was stirred for 5 minutes to achieve a homogeneous solution. Subsequently, 0.4 mL of pH-neutral phosphate buffer was added, and the resulting mixture was vigorously stirred for 24 hours at 40°C. The mixture was diluted with ethyl acetate, washed with water, and dried over anhydrous sodium sulfate. The crude mixture was purified over silica gel column chromatography and hexane/ethyl acetate was used as an eluent to afford the corresponding product (**3ao** or **3ap**).

Tert-butyl (3-(triisopropylsilyl)propioloyl)glycinate (3ao): White solid, yield 82% (43 mg)

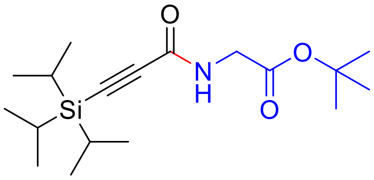

^1H NMR (400 MHz, $CDCl_3$) δ 6.29 (s, 1H), 3.98 (d, J = 5.2 Hz, 2H), 1.48 (s, 9H), 1.10 – 1.06 (m, 21H); ^{13}C NMR (100 MHz, $CDCl_3$) δ 168.5, 152.6, 99.3, 89.4, 82.9, 42.3, 28.2, 18.6, 11.1. HRMS (ESI): Calcd for $C_{18}H_{34}NO_3Si$ $[M+H]^+$: 340.2318; found: 340.2311

Reaction of Ethyl alkynoates with amino esters:

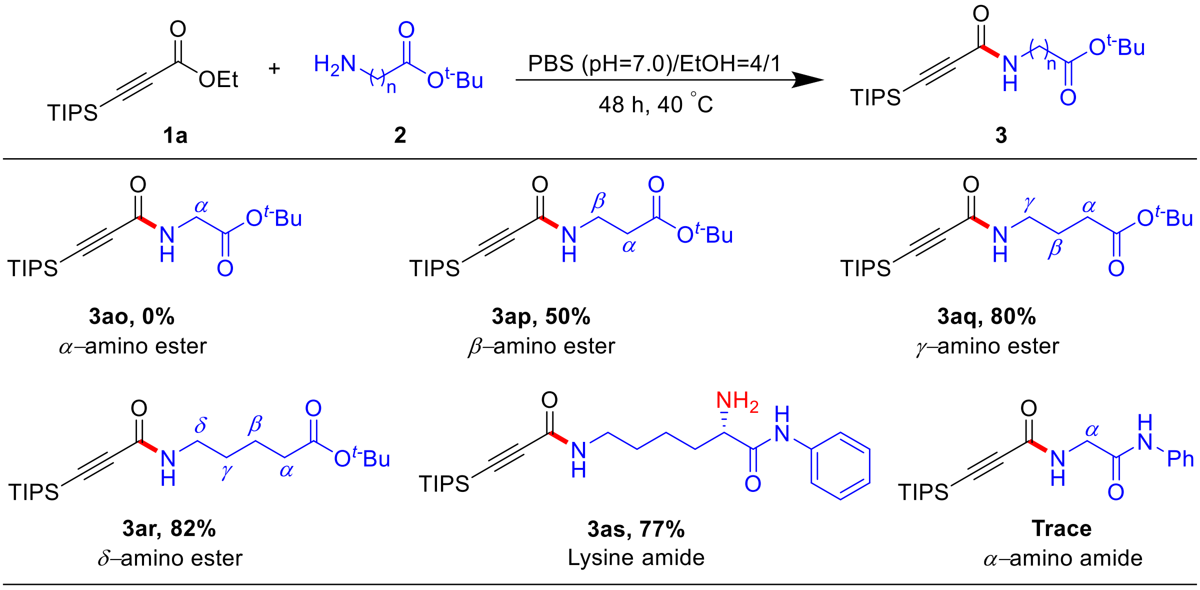

General Procedure: To a 4 ml glass vial, 0.1 mL ethanol was added to the mixture of ethyl 3-(triisopropylsilyl) propiolate (**1a**, 50 mg, 0.196 mmol) and the corresponding amino ester (**2**, 0.295 mmol). The mixture was stirred for 5 minutes to achieve a homogeneous solution. Subsequently, 0.4 mL of pH-neutral phosphate buffer was added, and the resulting mixture was vigorously stirred for 48 hours at 40°C. The mixture was diluted with ethyl acetate, washed with water, and dried over anhydrous sodium sulfate. The crude mixture was purified over silica gel column chromatography and hexane/ethyl acetate was used as an eluent to afford product **3** with the corresponding yield.

Similarly, under standard conditions, we conducted the experiments between ethyl 3-(triisopropylsilyl) propiolate (1a) with α-amino amide. Notable, only a trace amount (<10%) of product formation was observed.

Tert-butyl 3-(3-(triisopropylsilyl)propiolamido)propanoate (3ap): White solid, yield 50% (35 mg); Recovered starting material 44% (22 mg); ^{1}H NMR (400 MHz, $CDCl_3$, 50:7 mixture of rotamers) δ 6.36 (s, 1H), 3.67 (dd, J = 12.8, 6.4 Hz, 2H, rotamer), 3.51 (dd, J = 12.8, 6.4 Hz, 2H), 2.63 – 2.37 (m, 2H), 1.46 (s, 9H), 1.10 – 1.06 (m, 21H); ^{13}C NMR (100 MHz, $CDCl_3$) δ 171.8, 152.8, 99.9, 88.5, 81.5, 35.4, 34.9, 28.2, 18.6, 11.1; HRMS (ESI): Calcd for $C_{19}H_{35}NO_3Si$ $[M+H]^+$: 354.2464; found: 354.2477.

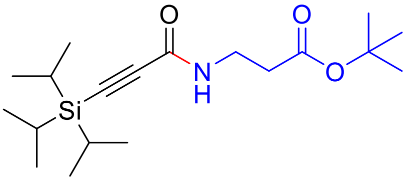

Tert-butyl 4-(3-(triisopropylsilyl)propiolamido)butanoate (3aq): White solid, yield 80%

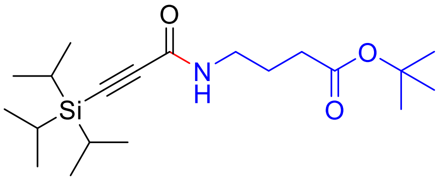

(58 mg); ^{1}H NMR (400 MHz, $CDCl_3$) δ 6.19 (s, 1H), 3.48 (q, *J* = 6.8 Hz, 2H, rotamer), 3.33 (q, *J* = 6.8 Hz, 2H, rotamer), 2.30 (t, *J* = 7.0 Hz, 2H), 1.87 – 1.79 (m, 2H), 1.44 (s, 9H), 1.11 – 1.07 (m, 21H); ^{13}C NMR (100 MHz, $CDCl_3$) δ 173.0, 153.0, 99.9, 88.2, 80.9, 39.7, 33.3, 28.2, 24.4, 18.6, 11.1; HRMS (ESI): Calcd for $C_{20}H_{37}NO_3Si$ $[M+H]^+$: 368.2621; found: 368.2634.

Tert-butyl 5-(3-(triisopropylsilyl)propiolamido)pentanoate (3ar): White solid, yield 82%

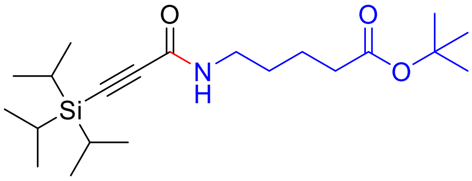

(62 mg); ^{1}H NMR (400 MHz, $CDCl_3$, 25:4 mixture of rotamers) δ 5.96 (s, 1H), 3.44 (q, *J* = 6.4 Hz, 2H, rotamer), 3.29 (q, *J* = 6.4 Hz, 2H), 2.24 (t, *J* = 6.8 Hz, 2H), 1.65 – 1.55 (m, 5H), 1.44 (s, 9H), 1.10 – 1.07 (d, *J* = 6.8 Hz, 21H); ^{13}C NMR (100 MHz, $CDCl_3$) δ 172.9, 152.9, 100.0, 80.5, 39.6, 35.0, 28.7, 28.2, 22.3, 18.6, 11.2; HRMS (ESI): Calcd for $C_{21}H_{39}NO_3Si$ $[M+H]^+$: 382.2777; found: 382.2788.

(S)-2-amino-N-phenyl-6-(3-(triisopropylsilyl)propiolamido)hexanamide (3as): White

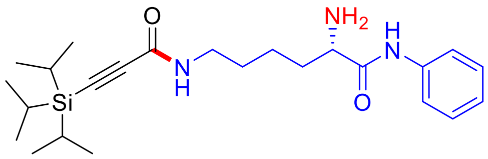

solid, yield 77% (65 mg), Crude product was isolated using preparative HPLC. ^{1}H NMR (400 MHz, DMSO, 25:6 mixture of rotamers) δ 10.45 (s, 1H), 8.60 (t, J = 5.6 Hz, 1H), 8.23 (s, 2H), 7.60 – 7.55 (m, 2H), 7.35 (t, J = 7.6 Hz, 2H), 7.12 (t, J = 7.2 Hz, 1H), 7.06 (t, J = 7.2 Hz, 1H, rotamer), 3.90 (t, J = 6.0 Hz, 1H), 3.12 – 3.02 (m, 2H), 1.84 – 1.75 (m, 2H), 1.49 – 1.41 (m, 2H), 1.38 – 1.30 (m, 2H), 1.08 – 1.03 (m, 21H); ^{13}C NMR (100 MHz, MeOD) δ 168.4, 155.1, 138.9, 130.0, 125.9, 121.3, 100.9, 89.3, 55.1, 40.1, 32.4, 29.9, 23.3, 18.9, 12.2; HRMS (ESI): Calcd for $C_{24}H_{39}N_3O_2Si$ $[M+H]^+$: 430.2890; found: 430.2895.

Experiments of Peptide Modification.

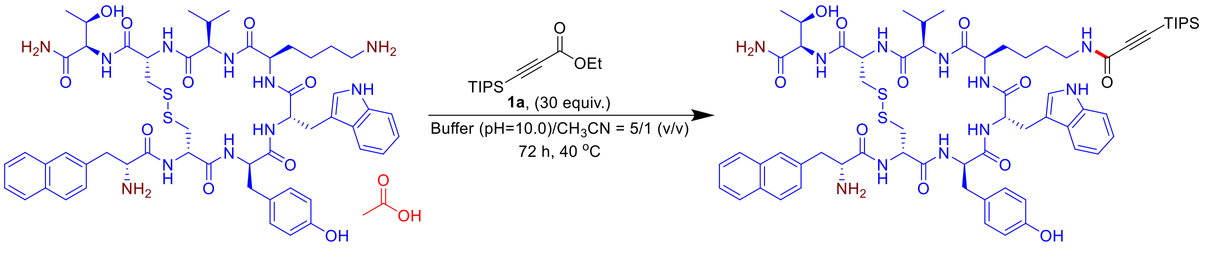

Lanreotide acetate (CAS: 2378114-72-6) was purchased from MedChem Express and used without further purification. ESI-MS was performed on a linear quadrupole ion trap detector mass spectrometer (LTQ XL from Thermo Fisher Scientific) coupled to an Ultimate 3000 UPLC. Data were processed using X-Calibur software.

HPLC methods: HPLC column was performed using Shim-pack Scepter C18-120 (5 μm, 10 × 250 mm) columns with a mobile phase of water with 0.1% trifluoroacetic acid (A) and acetonitrile with 0.1% trifluoroacetic acid (B) at a flow rate of 2.5 mL/min. Gradient used Solvent B (70 – 90)% over 90 min. λ = 220 nm, tR = 14.776 min.

General Procedure: To a 4 ml glass vial, 0.1 mL acetonitrile was added to the mixture of Lanreotide acetate (5 mg, 4.3 μmol) and ethyl 3-(triisopropylsilyl)propiolate (**1a**, 30 equiv.). Subsequently, 0.5 mL of buffer solution (pH=10.0) was added, and the resulting mixture was vigorously stirred for 72 hours at 40°C. After that 20 μL aliquot of the reaction mixture was evaluated by LC-MS to calculate the conjugation rate based on the consumption of the unmodified lanreotide. The conjugated species was obtained with one ligand modification, which was confirmed by ESI-MS. The residue was purified by preparative HPLC to obtain the modified pure products (2.8 mg) as an off-white solid. The regio-selectivity of the modification was analysed by LC-MS/MS after treatment with TCEP [(tris(2-carboxyethyl)phosphine)] (2.0 equiv.) for 30 min.

Elemental Composition Report

Page 1

Single Mass Analysis
Tolerance = 10.0 PPM / DBE: min = -1.5, max = 50.0
Element prediction: Off
Number of isotope peaks used for i-FIT = 2

Monoisotopic Mass, Even Electron Ions
664 formula(e) evaluated with 1 results within limits (all results (up to 1000) for each mass)
Elements Used:
C: 0-66 H: 0-90 N: 1-11 O: 1-11 Si: 1-2 S: 0-2
C66H89N11O11S2Si
KC-Lan 4 (0.104) Cm (3:9)

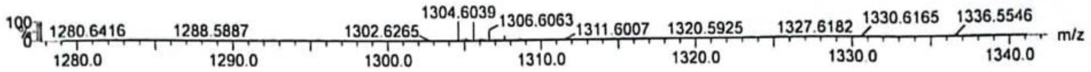

Minimum:				-1.5				
Maximum:		5.0	10.0	50.0				
Mass	Calc. Mass	mDa	PPM	DBE	i-FIT	Norm	Conf(%)	Formula
1304.6039	1304.6032	0.7	0.5	28.5	482.6	n/a	n/a	C66 H90 N11 O11 Si S2

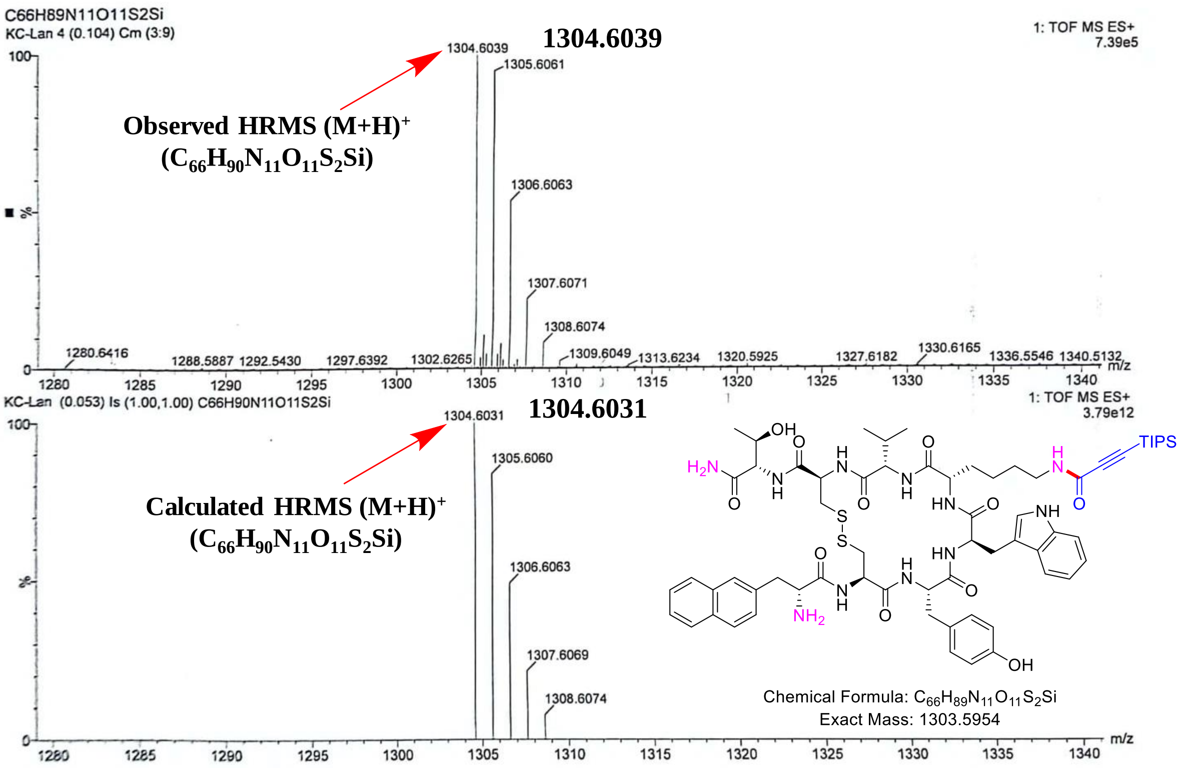

Fig S4. HRMS spectra of modified Lanreotide.

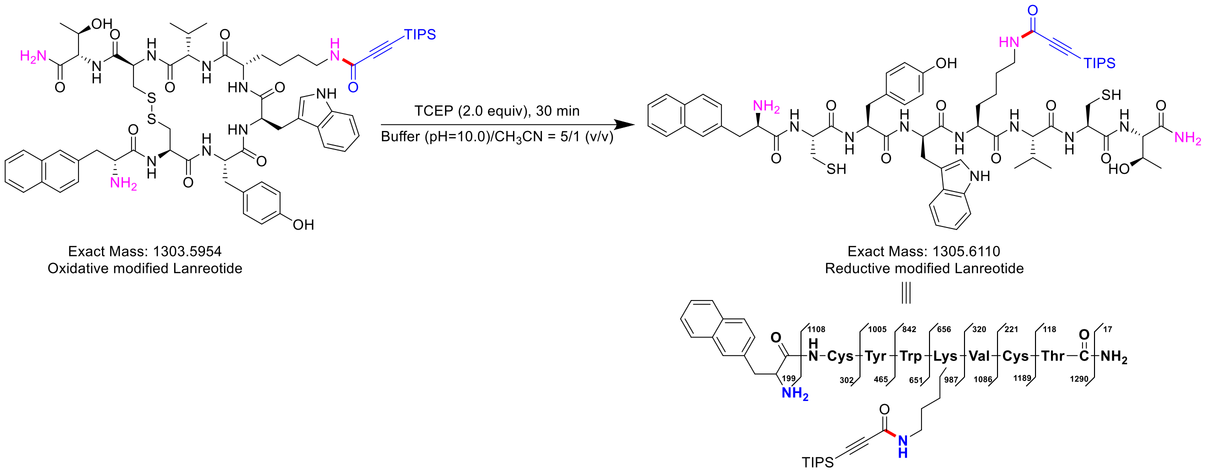

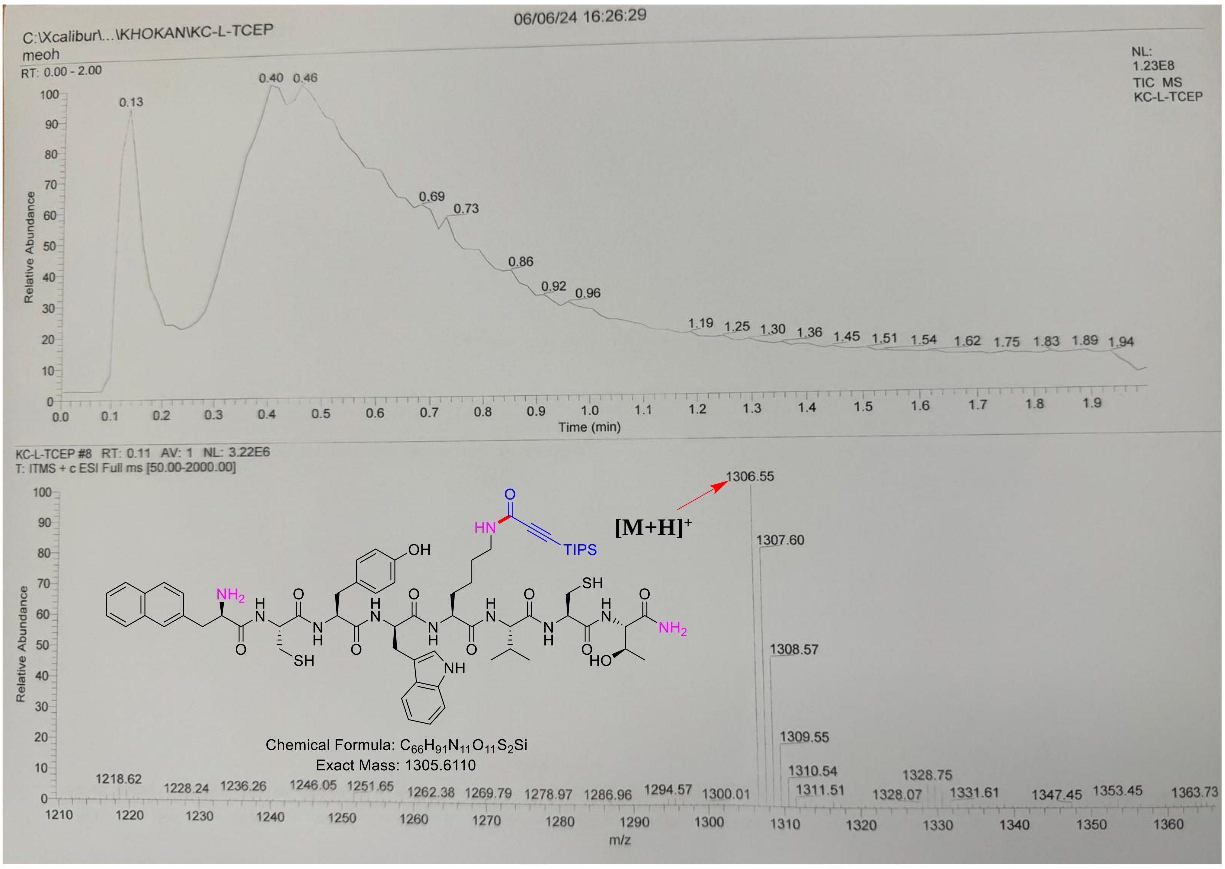

Fig S5. LCMS spectra for modified Lanreotide after treatment with TCEP.

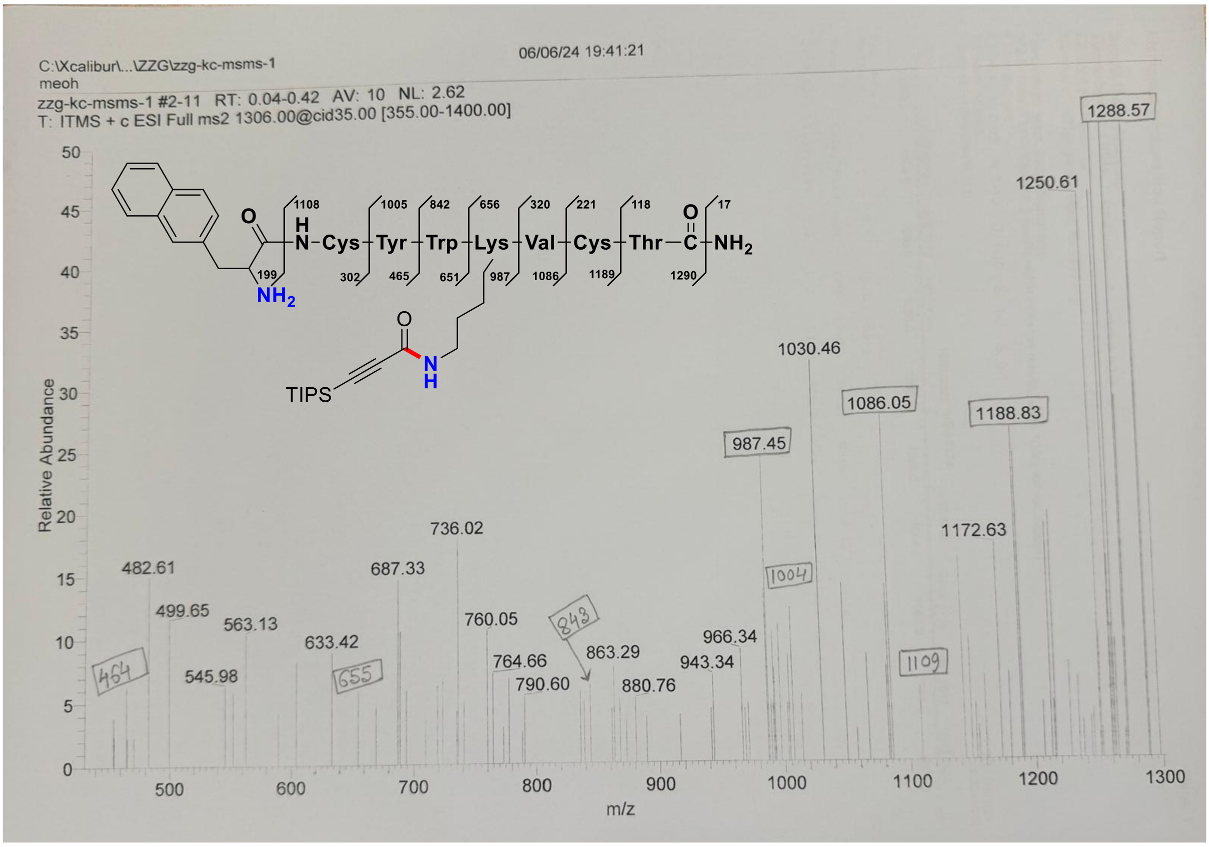

Fig S6. LC-Ms/Ms spectra for reductive modification Lanreotide.

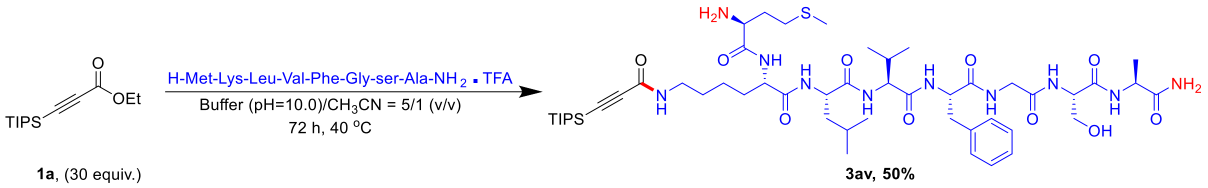

Procedure for the modification of peptides ***H-Met-Lys-Leu-Val-Phe-Gly-ser-Ala-NH2*** *with* ***1a****:* To a 4 ml glass vial, 0.1 mL acetonitrile was added to the mixture of **3v** (TFA salt) (3 mg, 3.1 µmol) and ethyl 3-(triisopropylsilyl)propiolate (**1a**, 30 equiv.). Subsequently, 0.5 mL of buffer solution (pH=10.0) was added, and the resulting mixture was vigorously stirred for 72 hours at 40°C. After that, 20 µL aliquot of the reaction mixture was evaluated by LC-MS to calculate the conjugation rate based on the consumption of the unmodified **3av**. The conjugated species was obtained with one ligand modification, which was confirmed by ESI-MS. The residue was purified by preparative HPLC to obtain the pure modified products **3av** (1.6 mg) in 50% of yield.

HPLC methods: HPLC column was performed using Shim-pack Scepter C18-120 (5 µm, 10 × 250 mm) columns with a mobile phase of water with 0.1% trifluoroacetic acid (A) and acetonitrile with 0.1% trifluoroacetic acid (B) at a flow rate of 2.5 mL/min. The gradient used: solvent B (0-15)% for (0-2) min, then (15-95)% for (2-72) min, then 95% for (72-85) min, then (95-15)% for (85-87) min then 15% for (87-90) min; λ = 220 nm, t_R = 51.47 min

Elemental Composition Report **Page 1**

Single Mass Analysis
Tolerance = 10.0 PPM / DBE: min = -1.5, max = 50.0
Element prediction: Off
Number of isotope peaks used for i-FIT = 2

Monoisotopic Mass, Even Electron Ions
382 formula(e) evaluated with 1 results within limits (all results (up to 1000) for each mass)
Elements Used:
C: 0-51 H: 0-90 N: 1-10 O: 1-10 Si: 1-2 S: 0-1
C51H86N10O10SSi
KC-P2 135 (2.399) Cm (58:137) 1: TOF MS ES+ 8.32e+006

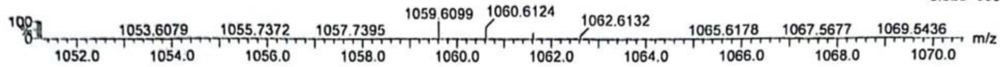

Minimum:				-1.5				
Maximum:		5.0	10.0	50.0				
Mass	Calc. Mass	mDa	PPM	DBE	i-FIT	Norm	Conf(%)	Formula
1059.6099	1059.6097	0.2	0.2	14.5	805.1	n/a	n/a	C51 H87 N10 O10 Si S

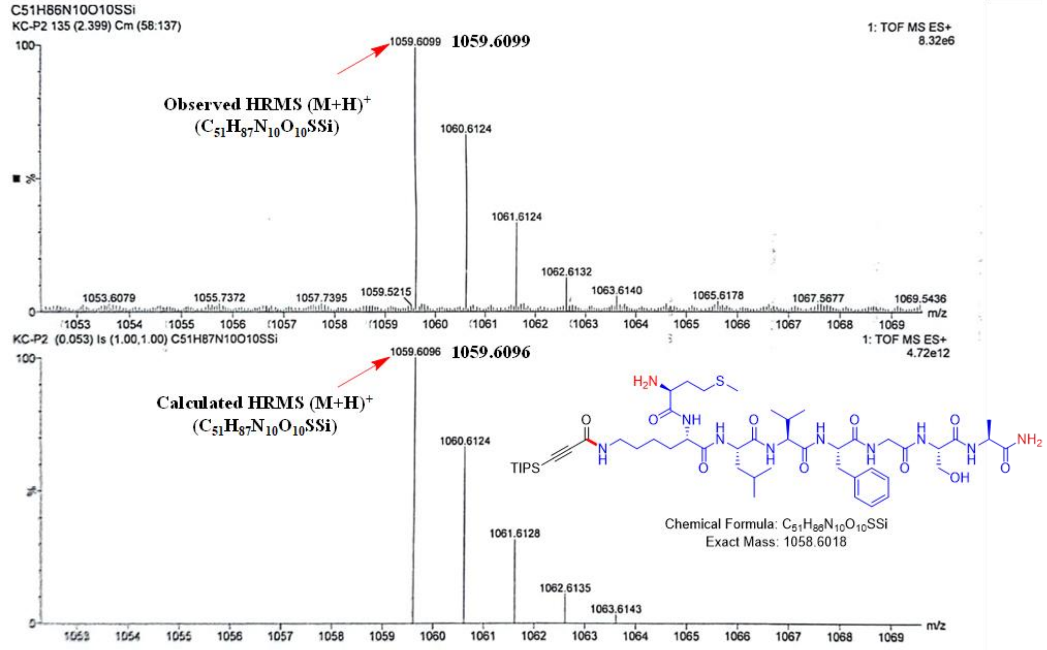

Fig S7. HRMS spectra of modified H-Met-Lys-Leu-Val-Phe-Gly-ser-Ala-NH_2 with **1a**.

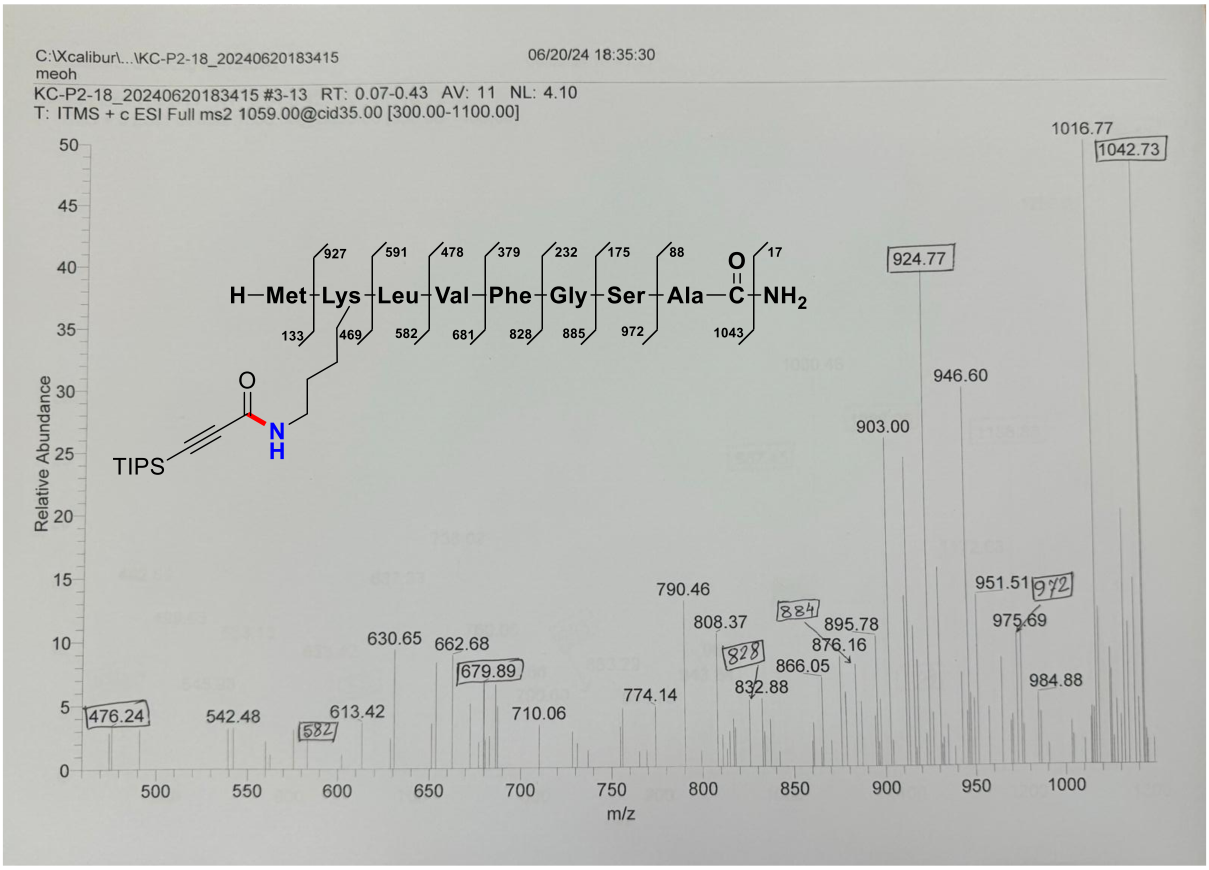

Fig S8. LC-Ms/Ms spectra for H-Met-Lys-Leu-Val-Phe-Gly-ser-Ala-NH_2 with **1a**.

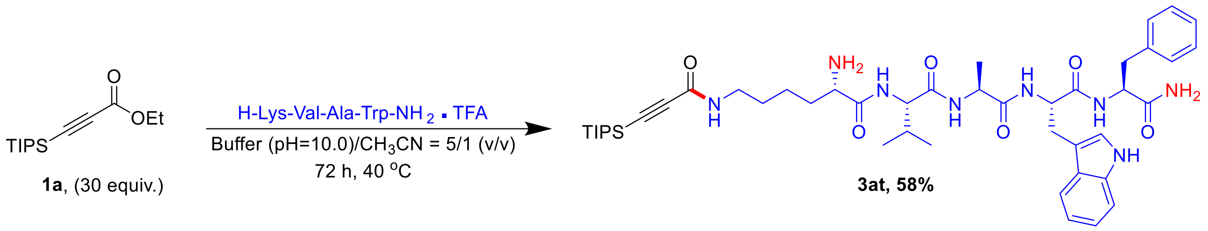

Procedure for the modification of peptides ***H-Lys-Val-Ala-Trp-Phe-NH2*** *with* ***1a****:* To a 4 ml glass vial, 0.1 mL acetonitrile was added to the mixture of **3t** (TFA salt) (3 mg, 3.9 μmol) and ethyl 3-(triisopropylsilyl)propiolate (**1a**, 30 equiv.). Subsequently, 0.5 mL of buffer solution (pH=10.0) was added, and the resulting mixture was vigorously stirred for 72 hours at 40°C. After that, 20 μL aliquot of the reaction mixture was evaluated by LC-MS to calculate the conjugation rate based on the consumption of the unmodified **3at**. The conjugated species was obtained with one ligand modification, which was confirmed by ESI-MS. The residue was purified by preparative HPLC to obtain the modified products **3at** (1.9 mg) in 58% of yield.

HPLC methods: HPLC column was performed using Shim-pack Scepter C18-120 (5 μm, 4.6 × 250 mm) columns with a mobile phase of water with 0.1% trifluoroacetic acid (A) and acetonitrile with 0.1% trifluoroacetic acid (B) at a flow rate of 1.0 mL/min. The gradient used: solvent B (5–60)% for (0-10) min, then (60-90)% for (10-30) min, then 90% for (30-35) min, then (90-15)% for (35-36) min, then 15 for (36-45) min. λ = 220 nm, Retention time (t_R) = 15.784 min at 45 °C.

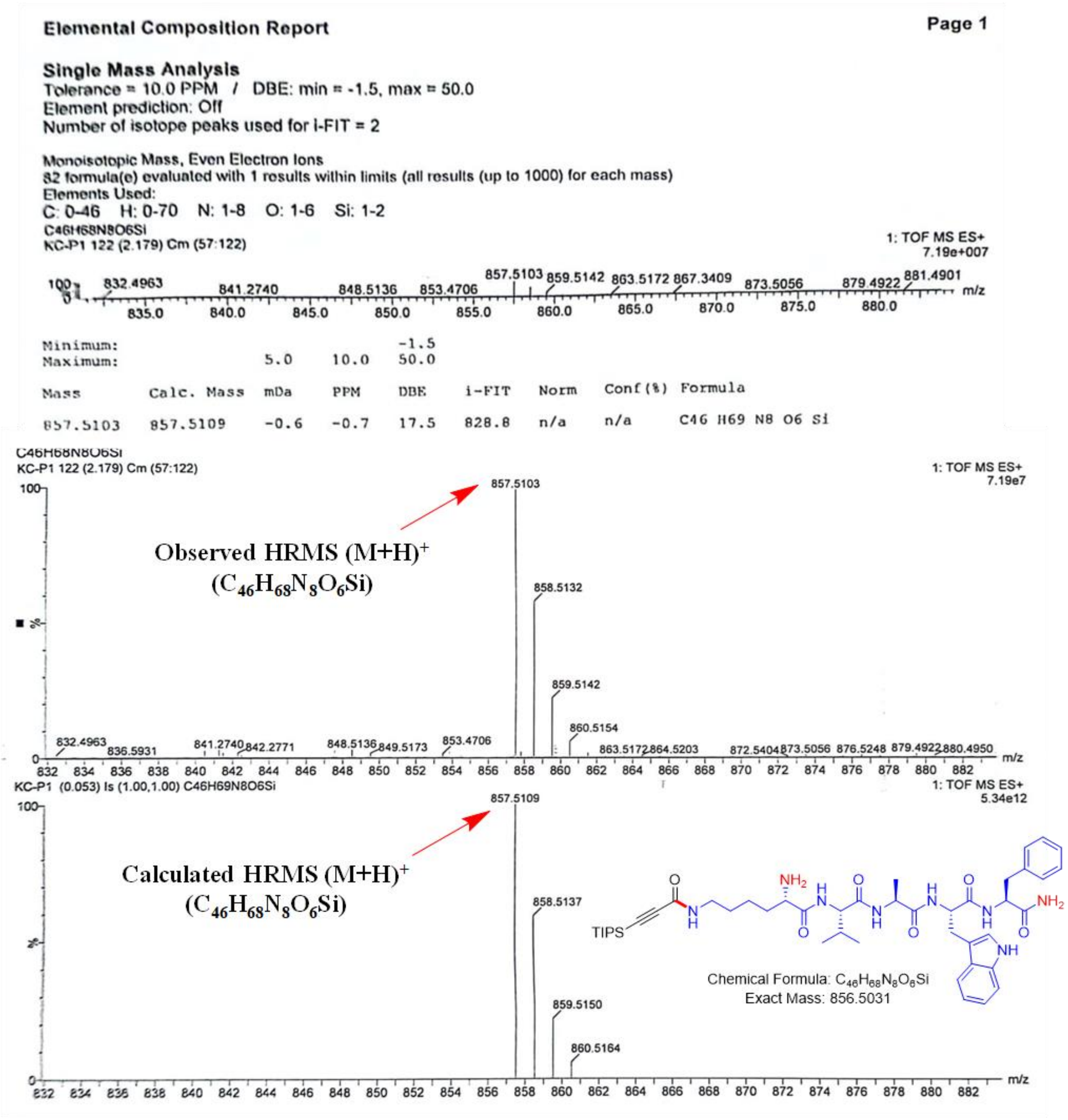

Elemental Composition Report **Page 1**

Single Mass Analysis
Tolerance = 10.0 PPM / DBE: min = -1.5, max = 50.0
Element prediction: Off
Number of isotope peaks used for i-FIT = 2

Monoisotopic Mass, Even Electron Ions
82 formula(e) evaluated with 1 results within limits (all results (up to 1000) for each mass)
Elements Used:
C: 0-46 H: 0-70 N: 1-8 O: 1-6 Si: 1-2

		mDa	PPM	DBE				
Minimum:				-1.5				
Maximum:		5.0	10.0	50.0				

Mass	Calc. Mass	mDa	PPM	DBE	i-FIT	Norm	Conf(%)	Formula
857.5103	857.5109	-0.6	-0.7	17.5	828.8	n/a	n/a	C46 H69 N8 O6 Si

Fig S9. HRMS spectra of modified H-Lys-Val-Ala-Trp-Phe-NH_2 with **1a**.

De-silylation and conjugation of modified Lanreotide

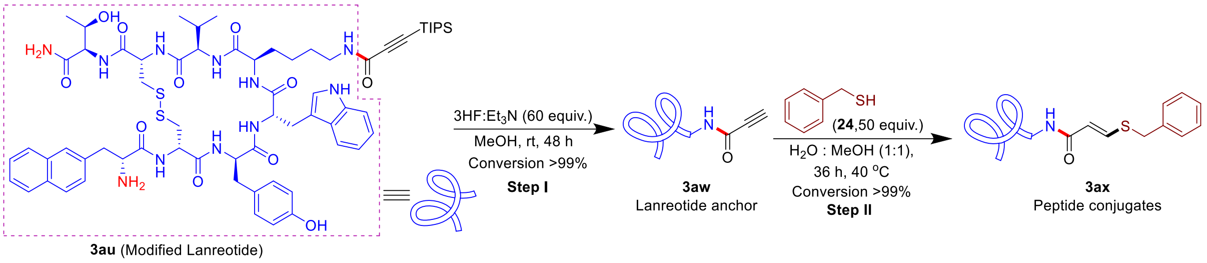

General Procedure for the de-silylation of the modified Lanreotide: To a 5 mL round-bottom flask, Modified Lanreotide (**3au**, 2.5 mg, 1.91 µmol) was added to 0.5 mL of methanol. Subsequently, 3HF.Et_3N (60 equiv.) was slowly added to the reaction mixture. The resulting mixture was vigorously stirred for 48 hours at room temperature. Afterward, an aliquot of the sample was analyzed using analytical HPLC to identify the product formation and confirm the complete conversion of **3au** to **3aw**. Without purifying the crude mixture **3aw**, we proceeded to the next step for C-S conjugation.

To a 10 mL round bottom flask, benzyl mercaptan (**24**, 50 equiv.) was added to the crude **3aw** mixture, followed by the addition of MeOH: H_2O (1:1) solvent (1 mL). The resulting mixture was vigorously stirred for 36 hours at 40°C. An aliquot of the sample was then analyzed using analytical HPLC to identify the product formation and confirm the complete conversion of **3aw** to **3ax**. To identify the products, we purified small amounts of **2aw** and **3ax** using analytical HPLC and recorded their HRMS spectra.

HPLC Method for 3au, 3aw, and 3ax: HPLC column was performed using Shim-pack Scepter C18-120 (5 µm, 4.6 × 250 mm) columns with a mobile phase of water with 0.1% trifluoroacetic acid (A) and acetonitrile with 0.1% trifluoroacetic acid (B) at a flow rate of 1.0 mL/min. The gradient used: solvent B (5–60)% for (0-10) min, then (60-90)% for (10-30) min, then 90% for (30-35) min, then (90-15)% for (35-36) min, then 15 for (36-45) min. λ = 220 nm, 45 ºC.

Retention time (t_R) for **3au** is = 18.98 min

Retention time (t_R) for **3av** is = 11.816 min

Retention time (t_R) for **3aw** is = 12.612 min

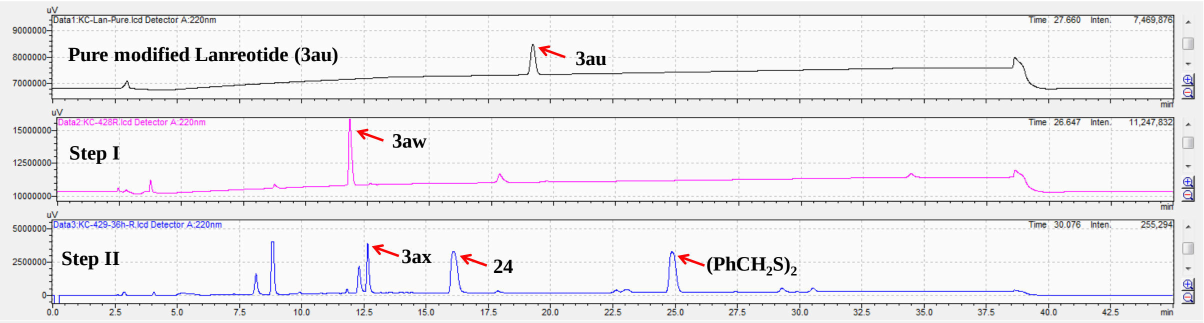

Figure S10. HPLC spectra to determine the conversion of **3au** to **3aw** in two steps.

Elemental Composition Report

Page 1

Single Mass Analysis
Tolerance = 10.0 PPM / DBE: min = -1.5, max = 50.0
Element prediction: Off
Number of isotope peaks used for i-FIT = 2

Monoisotopic Mass, Even Electron Ions
342 formula(e) evaluated with 1 results within limits (all results (up to 1000) for each mass)
Elements Used:
C: 0-57 H: 0-75 N: 1-11 O: 1-11 S: 0-2
C57H69N11O11S2
KC-428 4 (0.104) Cm (4:25)

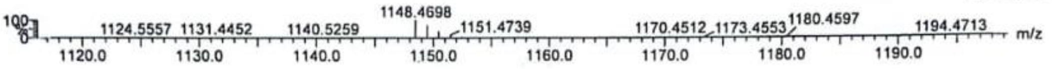

Minimum:				-1.5				
Maximum:		5.0	10.0	50.0				
Mass	Calc. Mass	mDa	PPM	DBE	i-FIT	Norm	Conf(%)	Formula
1148.4698	1148.4698	0.0	0.0	28.5	550.2	n/a	n/a	C57 H70 N11 O11 S2

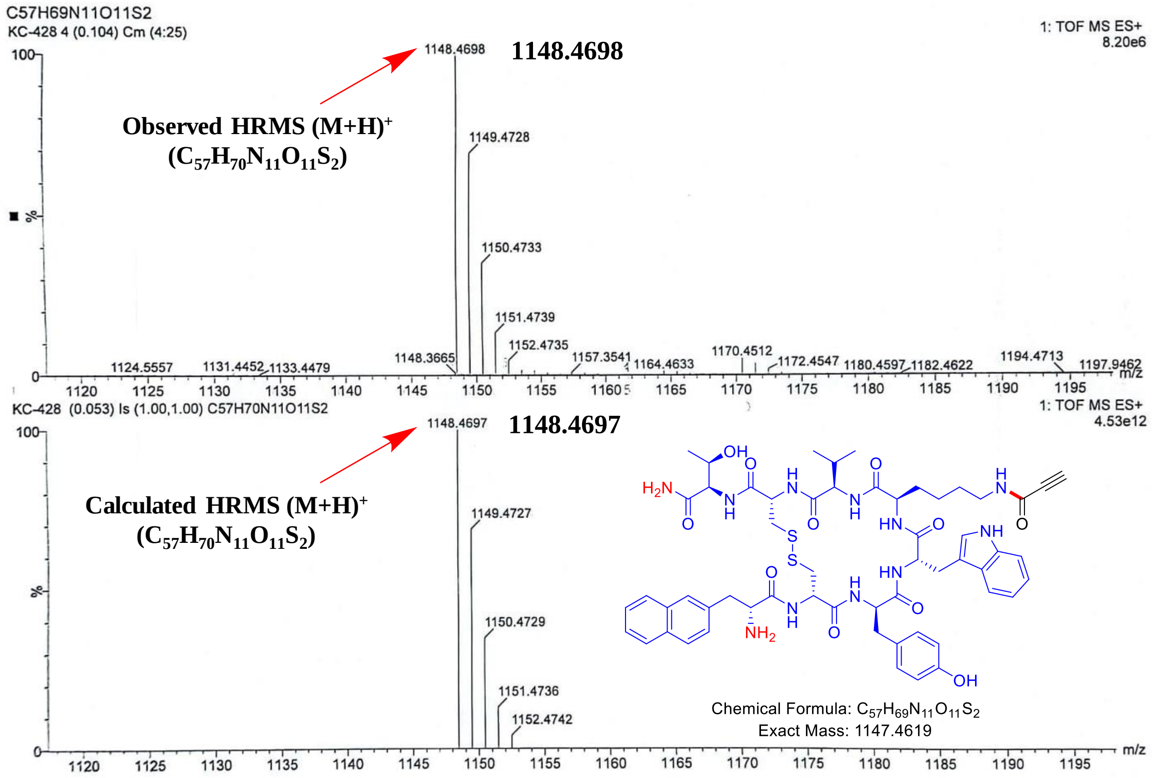

Figure 11. HRMS spectra of modified desilylated Lanreotide (**3aw**).

Elemental Composition Report Page 1

Single Mass Analysis
Tolerance = 10.0 PPM / DBE: min = -1.5, max = 50.0
Element prediction: Off
Number of isotope peaks used for i-FIT = 2

Monoisotopic Mass, Even Electron Ions
463 formula(e) evaluated with 1 results within limits (all results (up to 1000) for each mass)
Elements Used:
C: 0-64 H: 0-80 N: 1-11 O: 1-11 S: 0-3
C64H77N11O11S3
KC-429-raw 80 (1.429) Cm (80:86) 1: TOF MS ES+ 1.79e+006

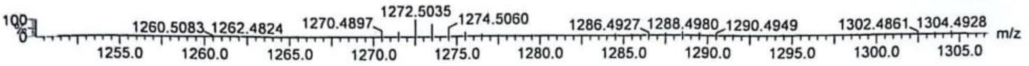

Minimum:				-1.5				
Maximum:		5.0	10.0	50.0				
Mass	Calc. Mass	mDa	PPM	DBE	i-FIT	Norm	Conf(%)	Formula
1272.5035	1272.5044	-0.9	-0.7	31.5	464.7	n/a	n/a	C64 H78 N11 O11 S3

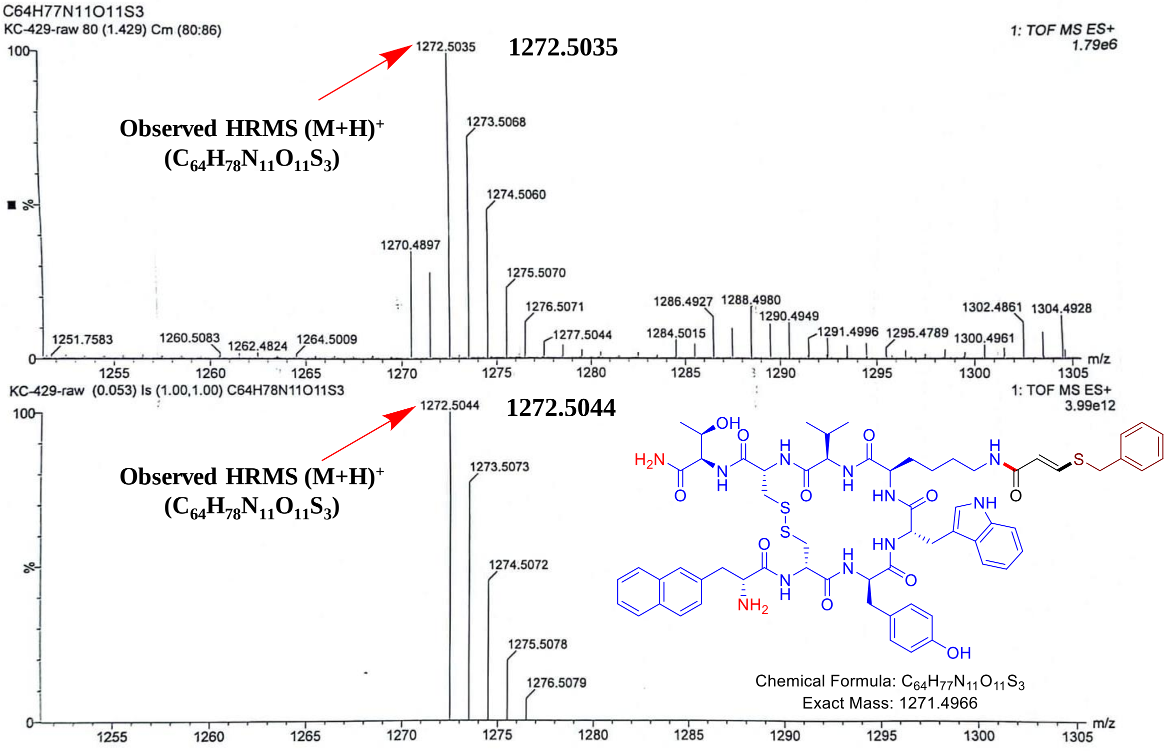

Figure 12. HRMS spectra for desilylation/conjugation of modified Lanreotide with benzyl mercaptan (**3ax**).

Experiment for Protein Modification.

Bovine Serum Albumin protein modification.

ESI-MS was performed on a linear quadrupole ion trap detector mass spectrometer (LTQ XL from Thermo-Fisher Scientific) coupled to Vanquish UHPLC (from Thermo-Fisher Scientific). Data were processed using Thermo BioPharma Finder 3.1. Bovine Serum Albumin (BSA) was purchased from the Sigma-Aldrich Catalog no. (A2153-10G). Protein was used without further purification.

The sequence of Bovine Serum Albumin (61): BSA is composed of 583 amino acids and contains one free cysteine (Cys34) and 59 lysins.

DTHKSEIAHRFKDLGEEHFKGLVLIAFSQYLQQCPFDEHVKLVNELTEFAKTCVADES
HAGCEKSLHTLFGDELCKVASLRETYGDMADCCEKQEPERNECFLSHKDDSPDLPKL
KPDPNTLCDEFKADEKKFWGKYLYEIARRHPYFYAPELLYYANKYNGVFQECCQAE
DKGACLLPKIETMREKVLTSSARQRLRCASIQKFGERALKAWSVARLSQKFPKAEFV
EVTKLVTDLTKVHKECCHGDLLECADDRADLAKYICDNQDTISSKLKECCDKPLLEK
SHCIAEVEKDAIPENLPPLTADFAEDKDVCKNYQEAKDAFLGSFLYEYSRRHPEYAVS
VLLRLAKEYEATLEECCAKDDPHACYSTVFDKLKHLVDEPQNLIKQNCDQFEKLGEY
GFQNALIVRYTRKVPQVSTPTLVEVSRSLGKVGTRCCTKPESERMPCTEDYLSLILNR
LCVLHEKTPVSEKVTKCCTESLVNRRPCFSALTPDETYVPKAFDEKLFTFHADICTLP
DTEKQIKKQTALVELLKHKPKATEEQLKTVMENFVAFVDKCCAADDKEACFAVEGP
KLVVSTQTALA.

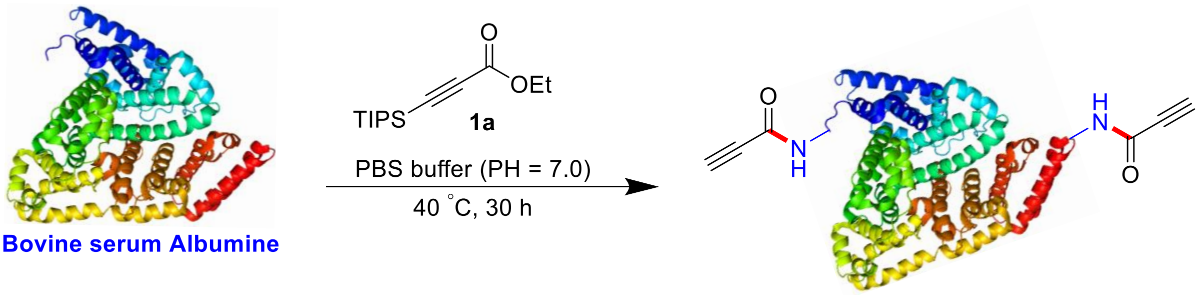

Reaction procedure of Bovine serum albumin with 1a:

To a 4-mL reaction tube, Bovine Serum Albumin (BSA) (3 mg, $4.5x10^{-5}$ mmol) was added with **1a** (20 mg) in PBS buffer (pH=7.0) (0.5 mL) solvents. After stirring for 36 hours at 40 °C, a 20

μL aliquot of the reaction mixture was placed for evaluation by performing ESI-MS, and the di-conjugated adduct with the loss of TIPS (triisopropylsilane) group was detected.

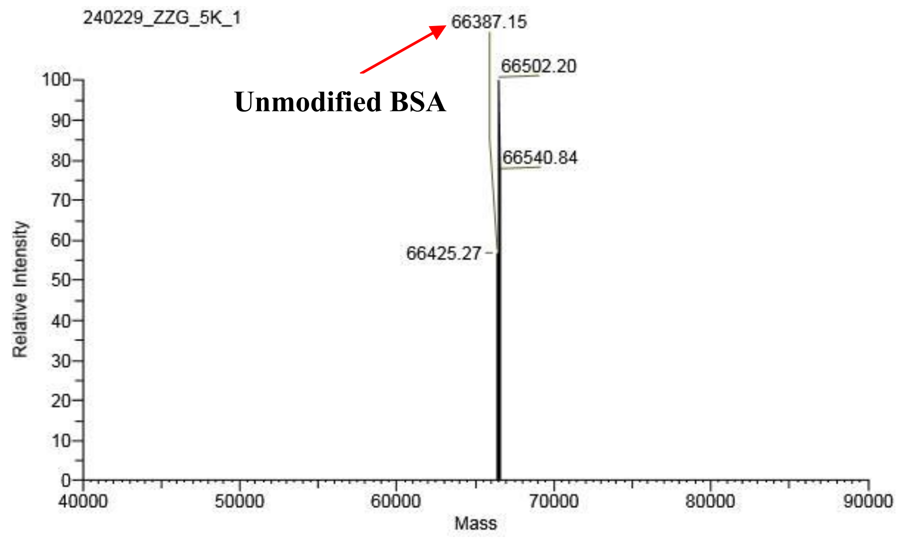

Fig S13. Deconvoluted mass spectrum of unmodified Bovine Serum Albumin

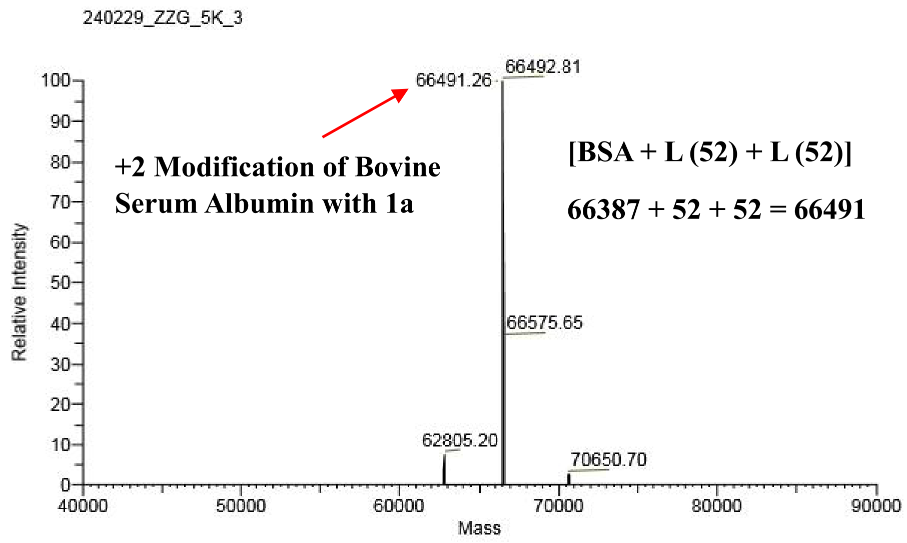

Fig S14. Deconvoluted mass spectrum of modified Bovine Serum Albumin

Sliding Windows ReSpect Masses Table																
Row Number	Average Mass	Sum Intensity	Relative Abundance	Fractional Abundance	Score	Number of Charge States	Charge State Distribution	Number of Detected Intervals	Mass Std Dev	PPM Std Dev	Delta Mass	Scan Range	Start Time (min)	Stop Time (min)	Apex RT
1	66492.81	6187358.88	100.00	20.99	67.78	16	56 - 71	3	4.61	69.27	0.00	324 - 408	5.220	6.520	5.873
2	66575.65	5458295.30	88.22	18.51	40.38	17	45 - 62	7	4.41	66.21	82.85	324 - 465	5.220	7.399	6.088
3	66491.26	5213062.13	84.25	17.68	64.06	25	48 - 72	8	5.83	87.74	-1.55	366 - 562	5.868	8.909	6.301
4	66540.66	4435469.88	71.69	15.05	49.08	10	45 - 54	3	3.79	56.95	47.85	324 - 408	5.220	6.520	5.873
5	66497.98	2929567.44	47.35	9.94	116.89	35	37 - 71	3	6.50	97.72	5.18	310 - 479	4.993	7.616	5.430
6	66505.18	2079016.14	33.60	7.05	46.70	16	42 - 60	4	4.56	68.56	12.37	352 - 521	5.655	8.268	6.088
7	66487.29	1694937.50	27.39	5.75	47.29	14	59 - 72	4	5.51	82.86	-5.52	395 - 521	6.307	8.268	6.960
8	62805.20	1110447.00	17.95	3.77	36.44	9	44 - 52	4	3.45	54.92	-3687.61	381 - 521	6.095	8.268	6.745
9	70650.70	373001.30	6.03	1.27	32.20	6	52 - 57	3	3.87	54.84	4157.90	409 - 521	6.520	8.268	6.960

Fig S15. Relative abundance values of components from the modified Bovine Serum Albumin.

Sample information for tandem MS analysis for BSA: To a 4-mL reaction tube, Bovine Serum Albumin (BSA) (3 mg, 4.5x10^{-5} mmol) was added with **1a** (20 mg) in PBS buffer (pH=7.0) (0.5 mL) solvents. After stirring for 36 hours at 40 °C. After that, an aliquot amount of the sample was subjected to in-gel digestion and was digested by trypsin, identified by applying the nanoLC-MS/MS platform.

Method information for Liquid chromatography with tandem mass spectroscopy:

The peptides were separated and analyzed using a Vanquish Neo UHPLC System coupled to an Orbitrap Exploris 480 (Thermo Fisher Scientific, MA, USA). Separation was performed on a EASY-Spray 75 µm × 15 cm column packed with PepMap Neo C18 2 µm, 100 Å (Thermo Fisher Scientific) using solvent A (0.1% formic acid) and solvent B (0.1% formic acid in 80% ACN) at flow rate of 300 nL/min with a 60 min gradient. Peptides were then analyzed on a Orbitrap Exploris 480 apparatus with an EASY nanospray source (Thermo Fisher Scientific) at an electrospray potential of 2.0 kV. Raw data files were processed and searched using Proteome Discoverer 2.1 (Thermo Fisher Scientific). The Sequest algorithm was then used for data searching to identify proteins.

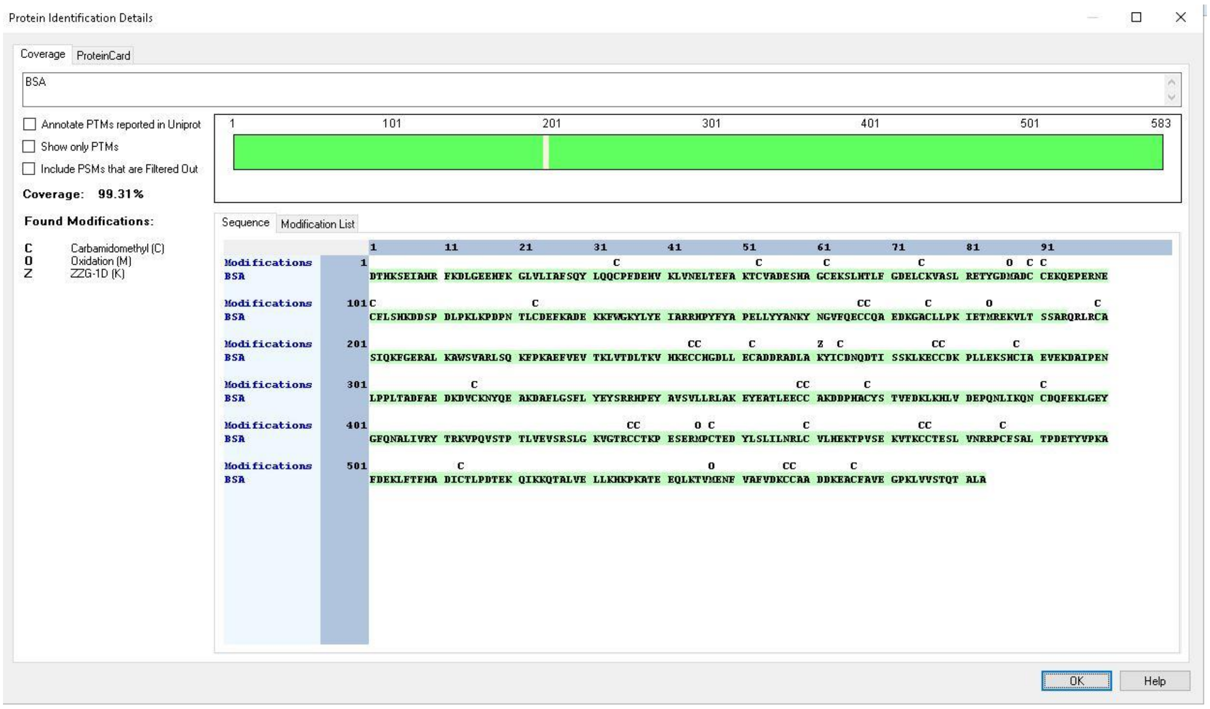

Fig. 16. Tandem MS result for the modification of BSA with **1a** (ZZG-1D).

Dynamic Modifications:

1. Dynamic Modification: Oxidation / +15.995 Da (M)

2. Dynamic Modification: Phospho / +79.966 Da (S, T, Y)

3. Dynamic Modification: Carbamidomethyl/ +57.02146 Da (C)

3. Dynamic Modification: ZZG-1D (K)/ +51.99492 Da (K)

Fragment Matches

Value Type: Theo. Mass [Da]

Ion Series | Neutral Losses | Precursor Ions

#1	b^+	b^{2+}	b^{3+}	Seq.	y^+	y^{2+}	y^{3+}	#2
1	130.04987	65.52857	44.02147	E				19
2	290.08052	145.54390	97.36502	C-Carbami...	2170.89505	1085.95116	724.30320	18
3	450.11117	225.55922	150.70857	C-Carbami...	2010.86440	1005.93584	670.95965	17
4	587.17008	294.08868	196.39488	H	1850.83375	925.92051	617.61610	16
5	644.19154	322.59941	215.40203	G	1713.77484	857.39106	571.92980	15
6	759.21848	380.11288	253.74435	D	1656.75338	828.88033	552.92264	14
7	872.30255	436.65491	291.43903	L	1541.72643	771.36686	514.58033	13
8	985.38661	493.19694	329.13372	L	1428.64237	714.82482	476.88564	12
9	1114.42921	557.71824	372.14792	E	1315.55831	658.28279	439.19095	11
10	1274.45985	637.73357	425.49147	C-Carbami...	1186.51571	593.76149	396.17676	10
11	1345.49697	673.25212	449.17051	A	1026.48506	513.74617	342.83321	9
12	1460.52391	730.76559	487.51282	D	955.44795	478.22761	319.15417	8
13	1575.55085	788.27907	525.85514	D	840.42101	420.71414	280.81185	7
14	1731.65196	866.32962	577.88884	R	725.39406	363.20067	242.46954	6
15	1802.68908	901.84818	601.56788	A	569.29295	285.15012	190.43584	5
16	1917.71602	959.36165	639.91019	D	498.25584	249.63156	166.75680	4
17	2030.80009	1015.90368	677.60488	L	383.22890	192.11809	128.41448	3
18	2101.83720	1051.42224	701.28392	A	270.14483	135.57605	90.71980	2
19				K-ZZG-1D	199.10772	100.05750	67.04076	1

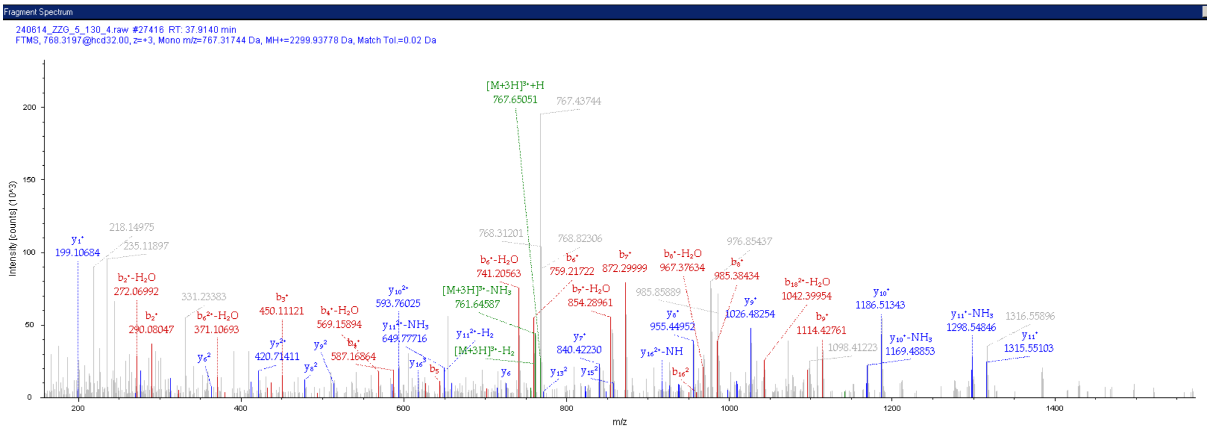

Fig. 17. Tandem MS analysis for Sequence: Sequence: ECCHGDLLECADDRADLAK, C2-Carbamidomethyl (57.02146 Da), C3-Carbamidomethyl (57.02146 Da), C10-Carbamidomethyl (57.02146 Da), K19-ZZG-1D (51.99492 Da) Charge: +3, Monoisotopic m/z: 767.31744 Da (+0.05 mmu/+0.06 ppm), $(M+H)^+$: 2299.93778 Da, RT: 37.9140 min, Identified with: Sequest HT (v1.17); XCorr:5.15, Ions matched by search engine: 0/0 Fragment match tolerance used for the search: 0.02 Da Fragments used for search: b; b-H_2O; b-NH_3; y; y-H_2O; y-NH_3 Protein references (1):- BSA

Fragment Matches

Value Type: Theo. Mass [Da]

Ion Series | Neutral Losses | Precursor Ions

#1	b⁺	b²⁺	b³⁺	Seq.	y⁺	y²⁺	y³⁺	#2
1	100.07569	50.54148	34.03008	V				22
2	237.13460	119.07094	79.71639	H	2565.09152	1283.04940	855.70202	21
3	365.22957	183.11842	122.41471	K	2428.03260	1214.51994	810.01572	20
4	494.27216	247.63972	165.42890	E	2299.93764	1150.47246	767.31740	19
5	654.30281	327.65504	218.77245	C-Carbami...	2170.89505	1085.95116	724.30320	18
6	814.33346	407.67037	272.11600	C-Carbami...	2010.86440	1005.93584	670.95965	17
7	951.39237	476.19982	317.80231	H	1850.83375	925.92051	617.61610	16
8	1008.41383	504.71055	336.80946	G	1713.77484	857.39106	571.92980	15
9	1123.44077	562.22403	375.15178	D	1656.75338	828.88033	552.92264	14
10	1236.52484	618.76606	412.84646	L	1541.72643	771.36686	514.58033	13
11	1349.60890	675.30809	450.54115	L	1428.64237	714.82482	476.88564	12
12	1478.65149	739.82939	493.55535	E	1315.55831	658.28279	439.19095	11
13	1638.68214	819.84471	546.89890	C-Carbami...	1186.51571	593.76149	396.17676	10
14	1709.71926	855.36327	570.57794	A	1026.48506	513.74617	342.83321	9
15	1824.74620	912.87674	608.92025	D	955.44795	478.22761	319.15417	8
16	1939.77314	970.39021	647.26257	D	840.42101	420.71414	280.81185	7
17	2095.87425	1048.44077	699.29627	R	725.39406	363.20067	242.46954	6
18	2166.91137	1083.95932	722.97531	A	569.29295	285.15012	190.43584	5
19	2281.93831	1141.47279	761.31762	D	498.25584	249.63156	166.75680	4
20	2395.02237	1198.01483	799.01231	L	383.22890	192.11809	128.41448	3
21	2466.05949	1233.53338	822.69135	A	270.14483	135.57605	90.71980	2
22				K-ZZG-1D	199.10772	100.05750	67.04076	1

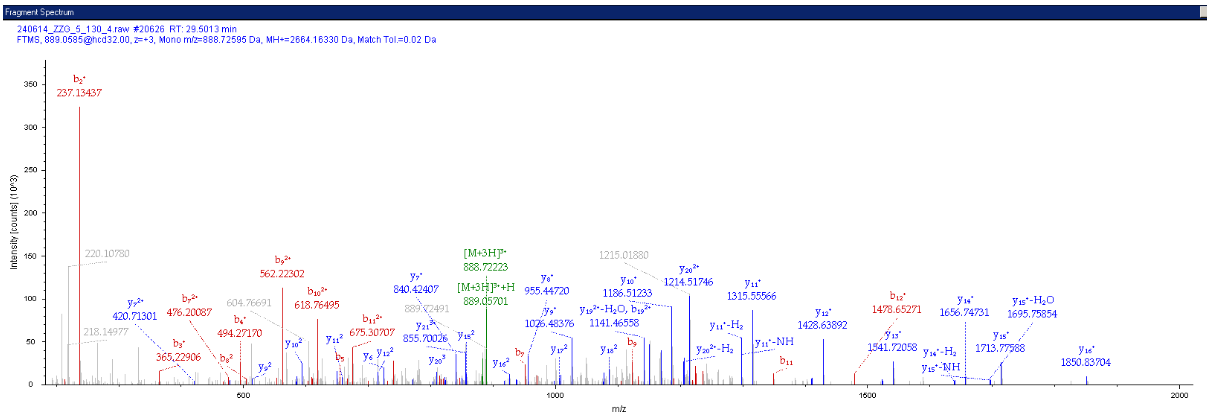

Fig. 18. Tandem MS analysis for Sequence: VHKECCHGDLLECADDRADLAK, C5-Carbamidomethyl (57.02146 Da), C6-Carbamidomethyl (57.02146 Da), C13-Carbamidomethyl (57.02146 Da), K22-ZZG-1D (51.99492 Da) Charge: +3, Monoisotopic m/z: 888.72595 Da (+1.13 mmu/+1.27 ppm), $(M+H)^+$: 2664.16330 Da, RT: 29.5013 min, Identified with: Sequest HT (v1.17); XCorr:7.46, Ions matched by search engine: 0/0 Fragment match tolerance used for search: 0.02 Da Fragments used for search: b; b-H_2O; b-NH_3; y; y-H_2O; y-NH_3; Protein references (1):- BSA

Fragment Matches

Value Type: Theo. Mass [Da]

Ion Series | Neutral Losses | Precursor Ions

#1	b+	b2+	b3+	b4+	Seq.	y+	y2+	y3+	y4+	#2
1	100.07569	50.54148	34.03008	25.77438	V					22
2	237.13460	119.07094	79.71639	60.03911	H	2565.09152	1283.04940	855.70202	642.02834	21
3	365.22957	183.11842	122.41471	92.06285	K	2428.03260	1214.51994	810.01572	607.76361	20
4	494.27216	247.63972	165.42890	124.32350	E	2299.93764	1150.47246	767.31740	575.73987	19
5	654.30281	327.65504	218.77245	164.33116	C-Carbami...	2170.89505	1085.95116	724.30320	543.47922	18
6	814.33346	407.67037	272.11600	204.33882	C-Carbami...	2010.86440	1005.93584	670.95965	503.47156	17
7	951.39237	476.19982	317.80231	238.60355	H	1850.83375	925.92051	617.61610	463.46390	16
8	1008.41383	504.71055	336.80946	252.85892	G	1713.77484	857.39106	571.92980	429.19917	15
9	1123.44077	562.22403	375.15178	281.61565	D	1656.75338	828.88033	552.92264	414.94380	14
10	1236.52484	618.76606	412.84646	309.88667	L	1541.72643	771.36686	514.58033	386.18707	13
11	1349.60890	675.30809	450.54115	338.15768	L	1428.64237	714.82482	476.88564	357.91605	12
12	1478.65149	739.82939	493.55535	370.41833	E	1315.55831	658.28279	439.19095	329.64503	11
13	1638.68214	819.84471	546.89890	410.42599	C-Carbami...	1186.51571	593.76149	396.17676	297.38439	10
14	1709.71926	855.36327	570.57794	428.18527	A	1026.48506	513.74617	342.83321	257.37672	9
15	1824.74620	912.87674	608.92025	456.94201	D	955.44795	478.22761	319.15417	239.61745	8
16	1939.77314	970.39021	647.26257	485.69874	D	840.42101	420.71414	280.81185	210.86071	7
17	2095.87425	1048.44077	699.29627	524.72402	R	725.39406	363.20067	242.46954	182.10397	6
18	2166.91137	1083.95932	722.97531	542.48330	A	569.29295	285.15012	190.43584	143.07870	5
19	2281.93831	1141.47279	761.31762	571.24004	D	498.25584	249.63156	166.75680	125.31942	4
20	2395.02237	1198.01483	799.01231	599.51105	L	383.22890	192.11809	128.41448	96.56268	3
21	2466.05949	1233.53338	822.69135	617.27033	A	270.14483	135.57605	90.71980	68.29167	2
22					K-ZZG-1D	199.10772	100.05750	67.04076	50.53239	1

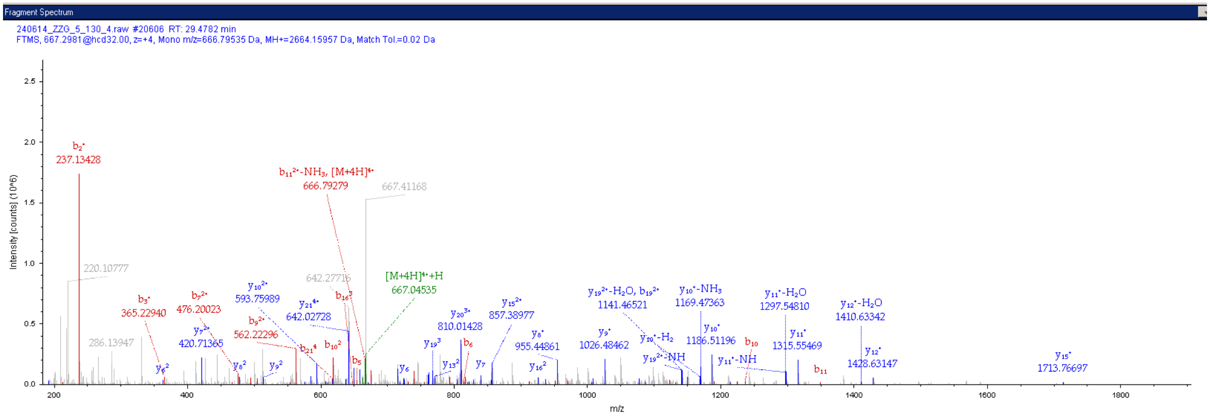

Fig. 19. Tandem MS analysis for Sequence: VHKECCHGDLLECADDRADLAK, C5-Carbamidomethyl (57.02146 Da), C6-Carbamidomethyl (57.02146 Da), C13-Carbamidomethyl (57.02146 Da), K22-ZZG-1D (51.99492 Da) Charge: +4, Monoisotopic m/z: 666.79535 Da (-0.09 mmu/-0.14 ppm), MH+: 2664.15957 Da, RT: 29.4782 min, Identified with: Sequest HT (v1.17); XCorr:5.70, Ions matched by search engine: 0/0 Fragment match tolerance used for search: 0.02 Da Fragments used for search: b; b-H_2O; b-NH_3; y; y-H_2O; y-NH_3; Protein references (1):- BSA

Myoglobin protein modification.

Myoglobin protein (Equine skeletal) was purchased from the Sigma-Aldrich Catalog no. (M0630-250MG). Protein was used without further purification. The sequence of Myoglobin consists of 154 amino acids and contains 19 lysin units.

MGLSDGEWQQVLNVWGKVEADIAGHGQEVLIRLFTGHPETLEKFDKFKHLKTEAE
MKASEDLKKHGTVVLTALGGILKKKGHHEAELKPLAQSHATKHKIPIKYLEFISDAIIH
VLHSKHPGDFGADAQGAMTKALELFRNDIAAKYKELGFQG.

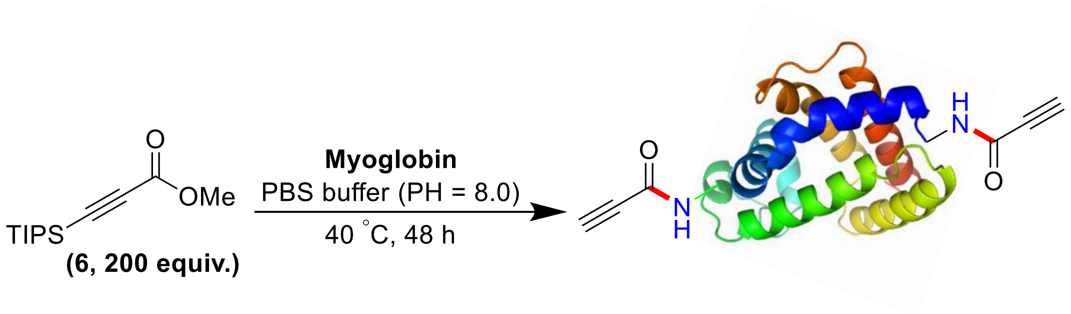

Reaction procedure of Myoglobin with 6:

To a 4-mL reaction tube, Myoglobin (6 mg, 0.35 μmol) was added with *β*-silyl methyl alkynoate **6** (200 equiv.) in PBS buffer (pH=8.0) (0.5 mL) solvents. After stirring for 48 hours at 40 °C, a 20 μL aliquot of the reaction mixture was placed for evaluation by performing ESI-MS, and the two-fold conjugated adduct with the loss of TIP (triisopropylsilane) group was detected.

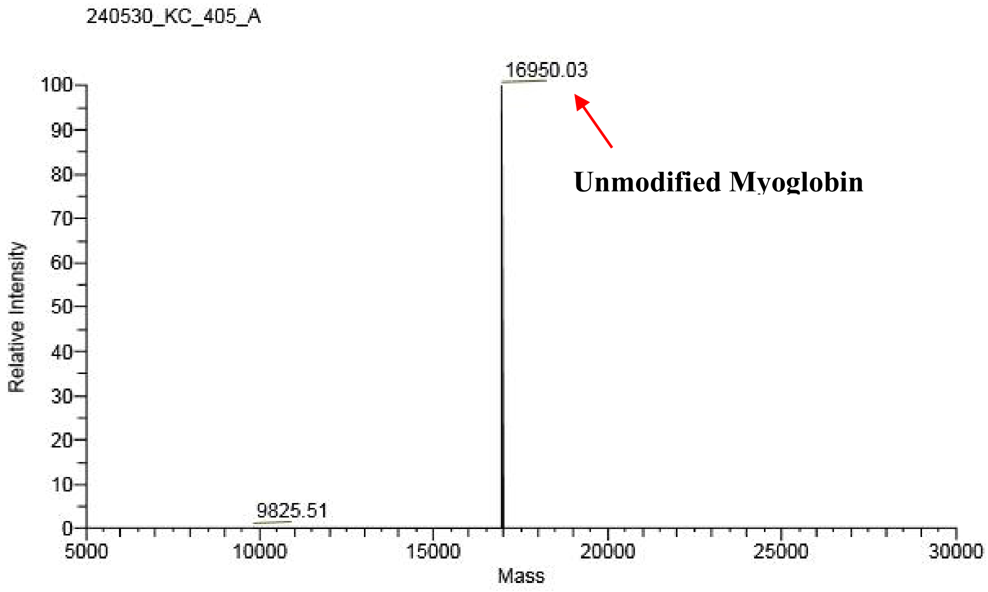

Fig S20. Deconvoluted mass spectrum of unmodified Myoglobin

Sliding Windows ReSpect Masses Table															
Row Number	Average Mass	Sum Intensity	Relative Abundance	Fractional Abundance	Score	Number of Charge States	Charge State Distribution	Number of Detected Intervals	Mass Std Dev	PPM Std Dev	Delta Mass	Scan Range	Start Time (min)	Stop Time (min)	Apex RT
1	16950.03	319868499.75	100.00	99.93	64.99	23	10 - 32	15	1.59	93.66	0.00	270 - 623	4.357	9.819	6.377
2	9825.51	220437.84	0.07	0.07	26.52	11	7 - 17	3	0.74	75.37	-7124.52	217 - 323	3.500	5.231	4.074

Fig21. Relative abundance values of components from the unmodified Myoglobin.

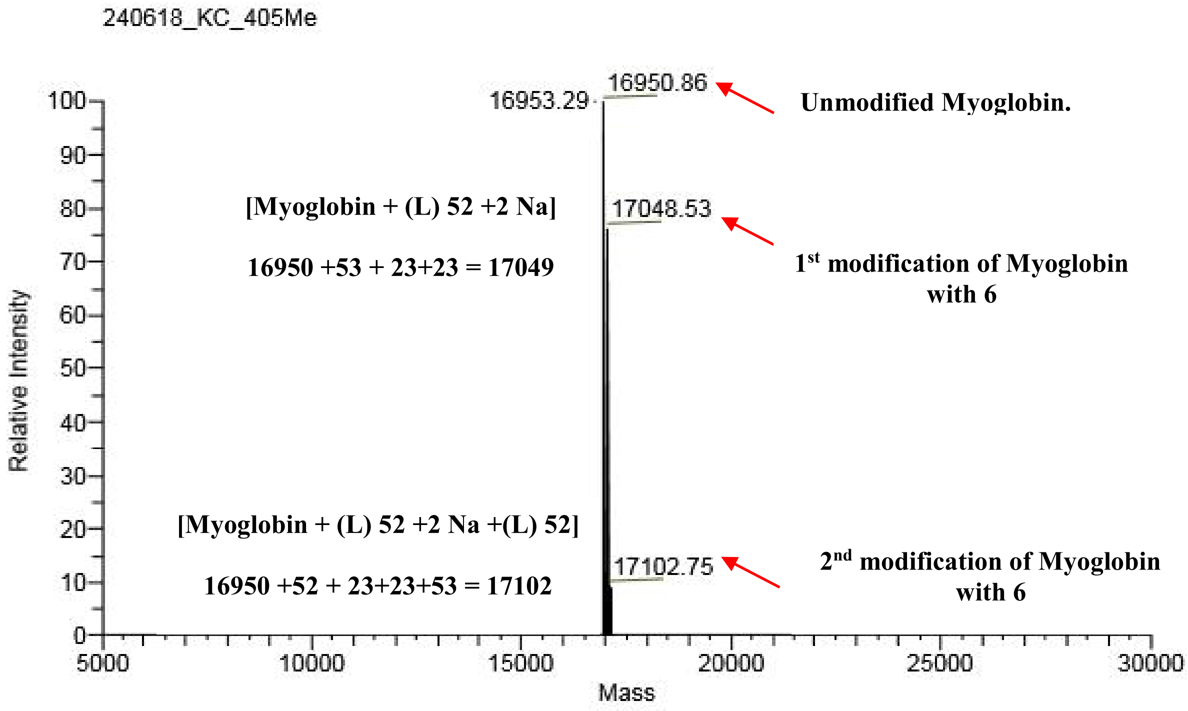

Fig S22. Deconvoluted mass spectrum of modified Myoglobin with β-silyl methyl alkynoate **(6)**

Sliding Windows ReSpect Masses Table															
Row Number	Average Mass	Sum Intensity	Relative Abundance	Fractional Abundance	Score	Number of Charge States	Charge State Distribution	Number of Detected Intervals	Mass Std Dev	PPM Std Dev	Delta Mass	Scan Range	Start Time (min)	Stop Time (min)	Apex RT
1	16950.86	42327059.00	100.00	43.97	64.22	23	9 - 31	4	1.60	94.21	0.00	386 - 501	6.000	7.717	6.487
2	17048.53	36971581.19	87.35	38.41	62.56	22	9 - 30	9	0.53	30.97	97.67	386 - 582	6.000	8.936	6.487
3	16953.29	8281355.13	19.57	8.60	75.27	19	13 - 31	4	1.46	86.33	2.42	452 - 582	6.973	8.936	7.718
4	17102.75	4425868.00	10.46	4.60	65.54	16	14 - 29	3	1.75	102.20	151.89	436 - 534	6.738	8.212	7.465
5	17149.17	4251530.69	10.04	4.42	53.08	14	12 - 25	3	1.16	67.76	198.31	436 - 550	6.738	8.453	7.227

Fig S23. Relative abundance values of components from the modified Myoglobin with β-silyl methyl alkynoate (**6**).

Reaction procedure of Myoglobin with *β*-silyl ethyl alkynoate (1a):

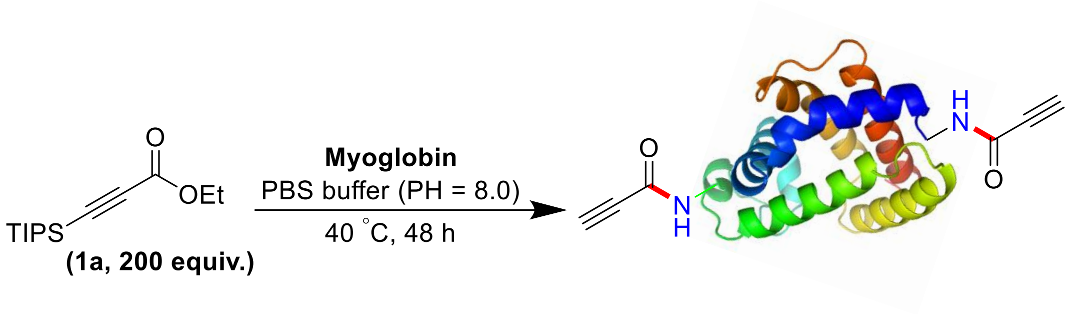

To a 4-mL reaction tube, Myoglobin (6 mg, 0.35 μmol) was added with **1a** (200 equiv.) in PBS buffer (pH=8.0) (0.5 mL) solvents. After stirring for 48 hours at 40 °C, a 20 μL aliquot of the reaction mixture was placed for evaluation by performing ESI-MS, and the two-fold conjugated adduct with the loss of TIP (triisopropylsilane) group was detected.

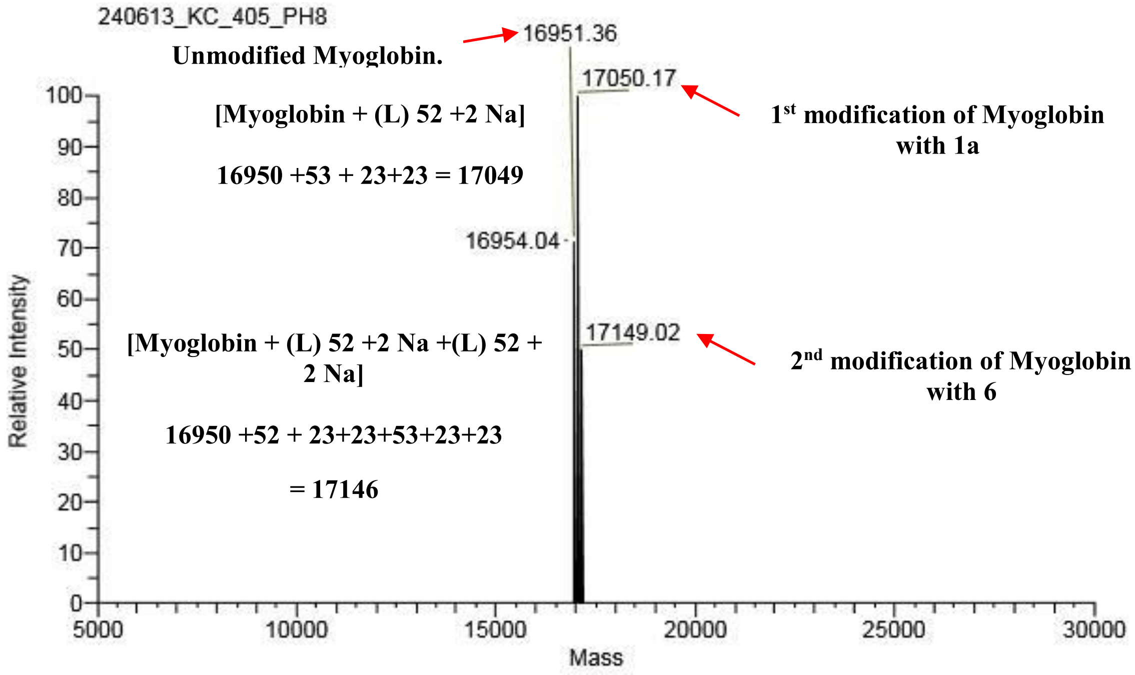

Fig S24. Deconvoluted mass spectrum of modified Myoglobin with *β*-silyl ethyl alkynoate **1a**

Sliding Windows ReSpect Masses Table

Row Number	Average Mass	Sum Intensity	Relative Abundance	Fractional Abundance	Score	Number of Charge States	Charge State Distribution	Number of Detected Intervals	Mass Std Dev	PPM Std Dev	Delta Mass	Scan Range	Start Time (min)	Stop Time (min)	Apex RT
1	17050.17	23997299.50	100.00	35.60	69.30	21	10 - 30	5	0.77	45.21	0.00	346 - 477	5.507	7.536	6.265
2	16951.36	15470614.00	64.47	22.95	69.51	23	9 - 31	3	1.60	94.18	-98.82	346 - 444	5.507	7.028	6.017
3	17149.02	14794584.59	61.65	21.95	62.31	20	10 - 29	10	0.71	41.60	98.85	346 - 560	5.507	8.823	6.265
4	16954.04	7134248.63	29.73	10.58	73.61	19	13 - 31	5	1.41	83.33	-96.13	428 - 560	6.769	8.823	7.537
5	17051.27	6015820.84	25.07	8.92	81.37	18	13 - 30	5	0.76	44.42	1.10	428 - 560	6.769	8.823	7.537

Fig S25. Relative abundance values of components from the modified Myoglobin with β-silyl ethyl alkynoate **1a**.

Sample information for tandem MS: To a 4-mL reaction tube, Myoglobin (6 mg, 3.5 μmol) was added with **6** (200 equiv.) in PBS buffer (pH=8.0) (0.5 mL) solvents. Then the mixture was stirred for 48 hours at 40 °C. After that, an aliquot amount of the sample was subjected to in-gel digestion and was digested by trypsin, identified by applying the nanoLC-MS/MS platform.

Method information for Liquid chromatography with tandem mass spectroscopy:
The peptides were separated and analyzed using a Vanquish Neo UHPLC System coupled to an Orbitrap Exploris 480 (Thermo Fisher Scientific, MA, USA). Separation was performed on a EASY-Spray 75 μm × 15 cm column packed with PepMap Neo C18 2 μm, 100 Å (Thermo Fisher Scientific) using solvent A (0.1% formic acid) and solvent B (0.1% formic acid in 80% ACN) at flow rate of 300 nL/min with a 60 min gradient. Peptides were then analyzed on a Orbitrap Exploris 480 apparatus with an EASY nanospray source (Thermo Fisher Scientific) at an electrospray potential of 2.0 kV. Raw data files were processed and searched using Proteome Discoverer 2.1 (Thermo Fisher Scientific). The Sequest algorithm was then used for data searching to identify proteins.

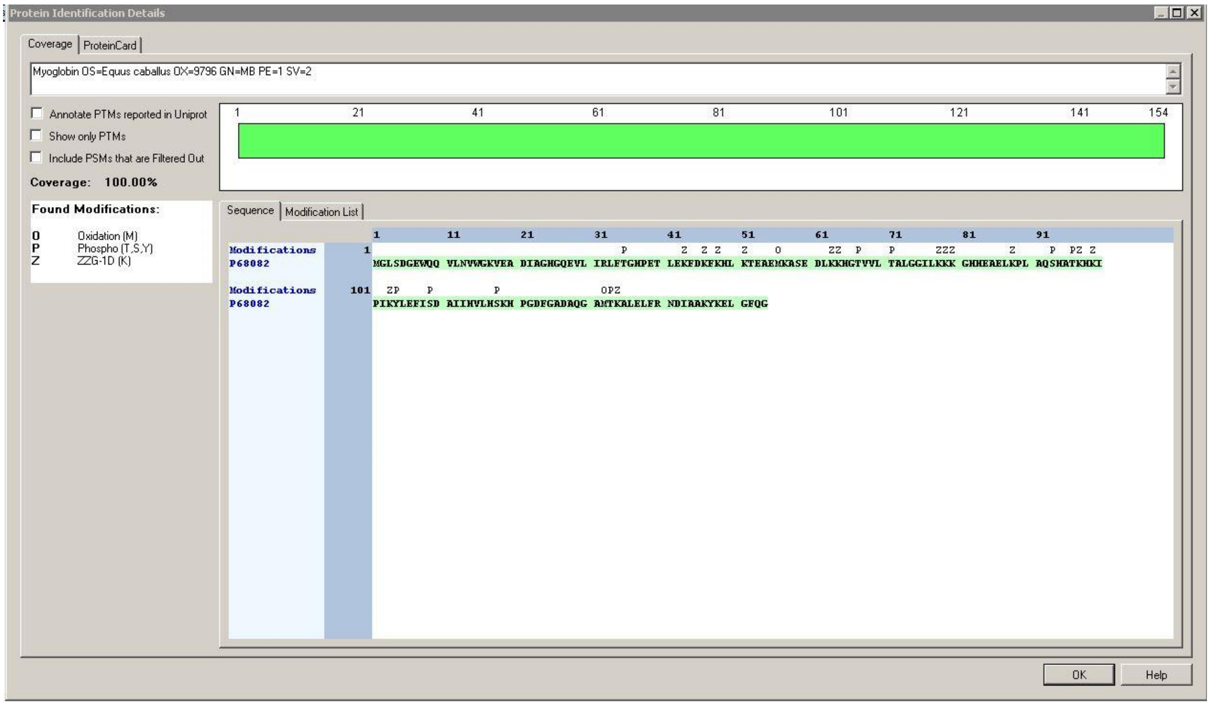

Fig S26. Tandem MS results for the modification of Myoglobin with *β*-silyl methyl alkynoates (**6**).

Dynamic Modifications:

1. Dynamic Modification: Oxidation / +15.995 Da (M)

2. Dynamic Modification: Phospho / +79.966 Da (S, T, Y)

3. Dynamic Modification: ZZG-1D (K)/ +51.99492 Da (K)

Fragment Matches

Value Type: Theo. Mass [Da]

Ion Series | Neutral Losses | Precursor Ions

#1	b^+	b^{2+}	b^{3+}	b^{4+}	Seq.	y^+	y^{2+}	y^{3+}	y^{4+}	#2
1	181.09715	91.05222	61.03724	46.02975	K-ZZG-1D					18
2	238.11862	119.56295	80.04439	60.28511	G	1853.96167	927.48447	618.65874	464.24587	17
3	375.17753	188.09240	125.73069	94.54984	H	1796.94021	898.97374	599.65159	449.99051	16
4	512.23644	256.62186	171.41700	128.81457	H	1659.88129	830.44429	553.96528	415.72578	15
5	641.27903	321.14316	214.43120	161.07522	E	1522.82238	761.91483	508.27898	381.46105	14
6	712.31615	356.66171	238.11023	178.83449	A	1393.77979	697.39353	465.26478	349.20040	13
7	841.35874	421.18301	281.12443	211.09514	E	1322.74268	661.87498	441.58574	331.44113	12
8	954.44281	477.72504	318.81912	239.36616	L	1193.70008	597.35368	398.57155	299.18048	11
9	1082.53777	541.77252	361.51744	271.38990	K	1080.61602	540.81165	360.87686	270.90946	10
10	1179.59053	590.29890	393.86836	295.65309	P	952.52106	476.76417	318.17854	238.88572	9
11	1292.67460	646.84094	431.56305	323.92411	L	855.46829	428.23778	285.82761	214.62253	8
12	1363.71171	682.35949	455.24209	341.68338	A	742.38423	371.69575	248.13293	186.35151	7
13	1491.77029	746.38878	497.92828	373.69803	Q	671.34711	336.17720	224.45389	168.59224	6
14	1578.80232	789.90480	526.93896	395.45604	S	543.28854	272.14791	181.76770	136.57759	5
15	1715.86123	858.43425	572.62526	429.72076	H	456.25651	228.63189	152.75702	114.81958	4
16	1786.89834	893.95281	596.30430	447.48004	A	319.19760	160.10244	107.07072	80.55486	3
17	1887.94602	944.47665	629.98686	472.74196	T	248.16048	124.58388	83.39168	62.79558	2
18					K	147.11280	74.06004	49.70912	37.53366	1

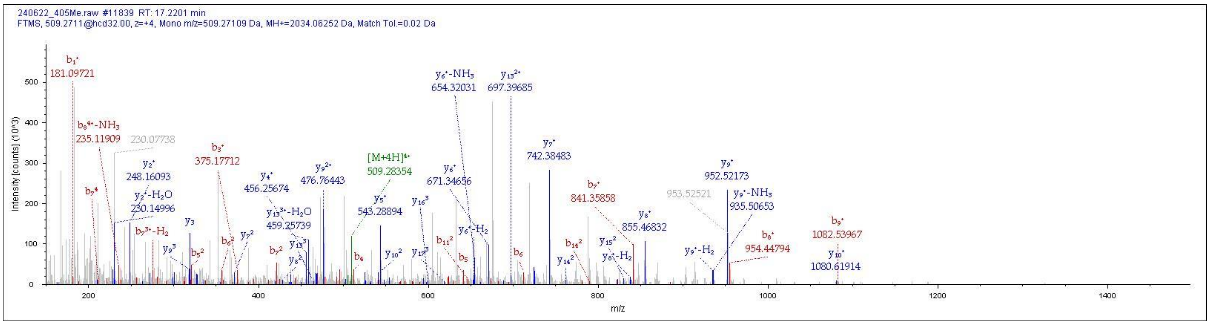

Figure S27. Tandem MS analysis for Sequence: KGHHEAELKPLAQSHATK, K1-ZZG-1D (51.99492 Da) Charge: +4, Monoisotopic m/z: 509.27109 Da (+2.75 mmu/+5.39 ppm), $(M+H)^+$: 2034.06252 Da, RT: 17.2201 min, Identified with: Sequest HT (v1.17); XCorr: 6.38, Ions matched by the search engine: 0/0 Fragment match tolerance used for search: 0.02 Da Fragments used for search: b; b-H_2O; b-NH_3; y; y-H_2O; y-NH_3 Protein references (1): - Myoglobin OS=Equus caballus OX=9796 GN=MB PE=1 SV=2

Fragment Matches

Value Type: Theo. Mass [Da]

Ion Series | Neutral Losses | Precursor Ions

#1	b+	b2+	b3+	Seq.	y+	y2+	y3+	#2
1	129.10224	65.05476	43.70560	K				18
2	186.12370	93.56549	62.71275	G	1905.95658	953.48193	635.99038	17
3	323.18262	162.09495	108.39906	H	1848.93512	924.97120	616.98322	16
4	460.24153	230.62440	154.08536	H	1711.87621	856.44174	571.29692	15
5	589.28412	295.14570	197.09956	E	1574.81730	787.91229	525.61062	14
6	660.32123	330.66426	220.77860	A	1445.77470	723.39099	482.59642	13
7	789.36383	395.18555	263.79279	E	1374.73759	687.87243	458.91738	12
8	902.44789	451.72758	301.48748	L	1245.69500	623.35114	415.90318	11
9	1082.53777	541.77252	361.51744	K-ZZG-1D	1132.61093	566.80911	378.20850	10
10	1179.59053	590.29890	393.86836	P	952.52106	476.76417	318.17854	9
11	1292.67460	646.84094	431.56305	L	855.46829	428.23778	285.82761	8
12	1363.71171	682.35949	455.24209	A	742.38423	371.69575	248.13293	7
13	1491.77029	746.38878	497.92828	Q	671.34711	336.17720	224.45389	6
14	1578.80232	789.90480	526.93896	S	543.28854	272.14791	181.76770	5
15	1715.86123	858.43425	572.62526	H	456.25651	228.63189	152.75702	4
16	1786.89834	893.95281	596.30430	A	319.19760	160.10244	107.07072	3
17	1887.94602	944.47665	629.98686	T	248.16048	124.58388	83.39168	2
18				K	147.11280	74.06004	49.70912	1

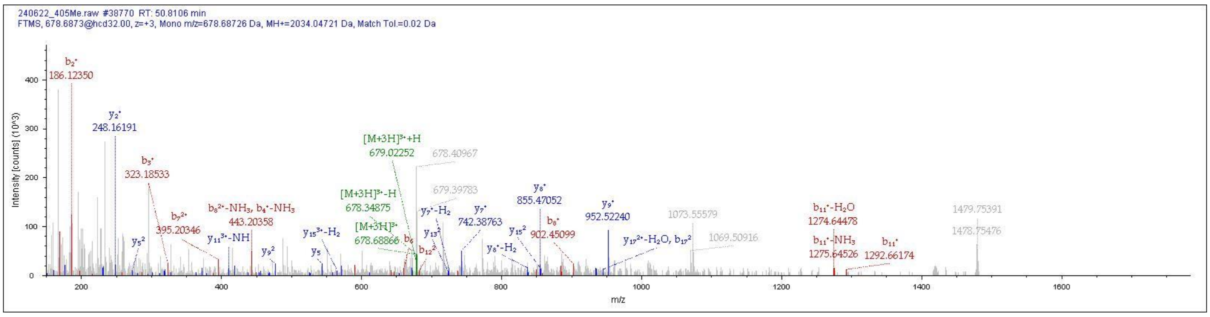

Figure S28. Tandem MS analysis for Sequence: KGHHEAELKPLAQSHATK, K9-ZZG-1D (51.99492 Da) Charge: +3, Monoisotopic m/z: 678.68726 Da (-1.45 mmu/-2.13 ppm), $(M+H)^+$: 2034.04721 Da, RT: 50.8106 min, Identified with: Sequest HT (v1.17); XCorr:2.98, Ions matched by the search engine: 0/0 Fragment match tolerance used for the search: 0.02 Da Fragments used for the search: b; b-H_2O; b-NH_3; y; y-H_2O; y-NH_3; Protein references (1): Myoglobin OS=Equus caballus OX=9796 GN=MB PE=1 SV=2

Fragment Matches

Value Type: Theo. Mass [Da]

Ion Series | Neutral Losses | Precursor Ions

#1	b+	b2+	b3+	b4+	Seq.	y+	y2+	y3+	y4+	#2
1	181.09715	91.05222	61.03724	46.02975	K-ZZG-1D					18
2	238.11862	119.56295	80.04439	60.28511	G	1905.95658	953.48193	635.99038	477.24460	17
3	375.17753	188.09240	125.73069	94.54984	H	1848.93512	924.97120	616.98322	462.98924	16
4	512.23644	256.62186	171.41700	128.81457	H	1711.87621	856.44174	571.29692	428.72451	15
5	641.27903	321.14316	214.43120	161.07522	E	1574.81730	787.91229	525.61062	394.45978	14
6	712.31615	356.66171	238.11023	178.83449	A	1445.77470	723.39099	482.59642	362.19913	13
7	841.35874	421.18301	281.12443	211.09514	E	1374.73759	687.87243	458.91738	344.43986	12
8	954.44281	477.72504	318.81912	239.36616	L	1245.69500	623.35114	415.90318	312.17921	11
9	1134.53268	567.76998	378.84908	284.38863	K-ZZG-1D	1132.61093	566.80911	378.20850	283.90819	10
10	1231.58545	616.29636	411.20000	308.65182	P	952.52106	476.76417	318.17854	238.88572	9
11	1344.66951	672.83839	448.89469	336.92284	L	855.46829	428.23778	285.82761	214.62253	8
12	1415.70663	708.35695	472.57373	354.68211	A	742.38423	371.69575	248.13293	186.35151	7
13	1543.76520	772.38624	515.25992	386.69676	Q	671.34711	336.17720	224.45389	168.59224	6
14	1630.79723	815.90225	544.27059	408.45477	S	543.28854	272.14791	181.76770	136.57759	5
15	1767.85614	884.43171	589.95690	442.71949	H	456.25651	228.63189	152.75702	114.81958	4
16	1838.89326	919.95027	613.63594	460.47877	A	319.19760	160.10244	107.07072	80.55486	3
17	1939.94093	970.47411	647.31850	485.74069	T	248.16048	124.58388	83.39168	62.79558	2
18					K	147.11280	74.06004	49.70912	37.53366	1

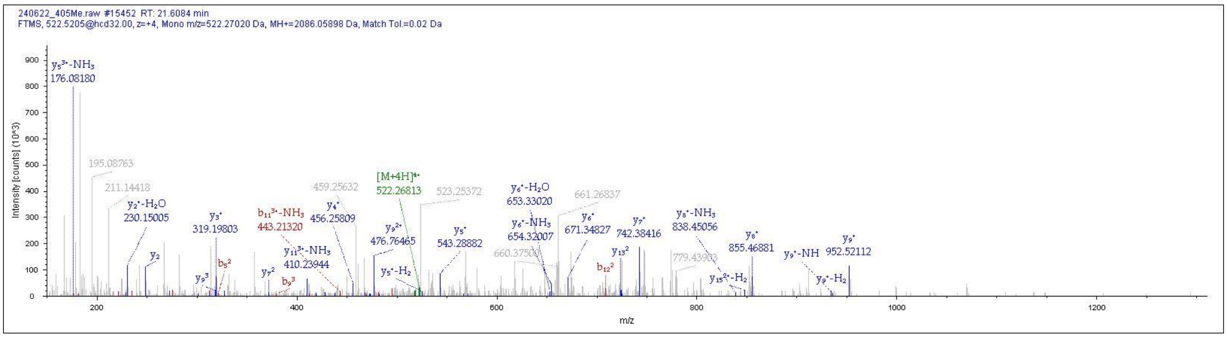

Figure S29. Tandem MS analysis for Sequence: Sequence: KGHHEAELKPLAQSHATK, K1-ZZG-1D (51.99492 Da), K9-ZZG-1D (51.99492 Da) Charge: +4, Monoisotopic m/z: 522.27020 Da (+3.13 mmu/+6 ppm), $(M+H)^+$: 2086.05898 Da, RT: 21.6084 min, Identified with: Sequest HT (v1.17); XCorr:2.89, Ions matched by search engine: 0/0 Fragment match tolerance used for the search: 0.02 Da Fragments used for search: b; b-H_2O; b-NH_3; y; y-H_2O; y-NH_3; Protein references (1): Myoglobin OS=Equus caballus OX=9796 GN=MB PE=1 SV=2

Fragment Matches

Value Type: Theo. Mass [Da]

Ion Series | Neutral Losses | Precursor Ions

#1	b^+	b^{2+}	b^{3+}	b^{4+}	b^{5+}	Seq.	y^+	y^{2+}	y^{3+}	y^{4+}	y^{5+}	#2
1	181.09715	91.05222	61.03724	46.02975	37.02525	K-ZZG-1D						18
2	238.11862	119.56295	80.04439	60.28511	48.42954	G	1905.95658	953.48193	635.99038	477.24460	381.99714	17
3	375.17753	188.09240	125.73069	94.54984	75.84133	H	1848.93512	924.97120	616.98322	462.98924	370.59285	16
4	512.23644	256.62186	171.41700	128.81457	103.25311	H	1711.87621	856.44174	571.29692	428.72451	343.18106	15
5	641.27903	321.14316	214.43120	161.07522	129.06163	E	1574.81730	787.91229	525.61062	394.45978	315.76928	14
6	712.31615	356.66171	238.11023	178.83449	143.26905	A	1445.77470	723.39099	482.59642	362.19913	289.96076	13
7	841.35874	421.18301	281.12443	211.09514	169.07757	E	1374.73759	687.87243	458.91738	344.43986	275.75334	12
8	954.44281	477.72504	318.81912	239.36616	191.69438	L	1245.69500	623.35114	415.90318	312.17921	249.94482	11
9	1134.53268	567.76998	378.84908	284.38863	227.71236	K-ZZG-1D	1132.61093	566.80911	378.20850	283.90819	227.32801	10
10	1231.58545	616.29636	411.20000	308.65182	247.12291	P	952.52106	476.76417	318.17854	238.88572	191.31003	9
11	1344.66951	672.83839	448.89469	336.92284	269.73972	L	855.46829	428.23778	285.82761	214.62253	171.89948	8
12	1415.70663	708.35695	472.57373	354.68211	283.94715	A	742.38423	371.69575	248.13293	186.35151	149.28267	7
13	1543.76520	772.38624	515.25992	386.69676	309.55886	Q	671.34711	336.17720	224.45389	168.59224	135.07524	6
14	1630.79723	815.90225	544.27059	408.45477	326.96527	S	543.28854	272.14791	181.76770	136.57759	109.46353	5
15	1767.85614	884.43171	589.95690	442.71949	354.37705	H	456.25651	228.63189	152.75702	114.81958	92.05712	4
16	1838.89326	919.95027	613.63594	460.47877	368.58447	A	319.19760	160.10244	107.07072	80.55486	64.64534	3
17	1939.94093	970.47411	647.31850	485.74069	388.79401	T	248.16048	124.58388	83.39168	62.79558	50.43792	2
18						K	147.11280	74.06004	49.70912	37.53366	30.22838	1

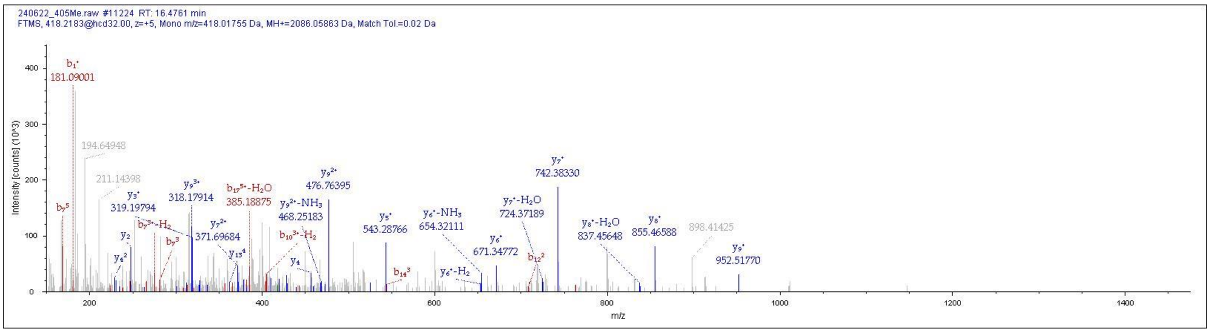

Figure S30. Tandem MS analysis for Sequence: Sequence: KGHHEAELKPLAQSHATK, K1-ZZG-1D (51.99492 Da), K9-ZZG-1D (51.99492 Da) Charge: +5, Monoisotopic m/z: 418.01755 Da (+2.44 mmu/+5.83 ppm), $(M+H)^+$: 2086.05863 Da, RT: 16.4761 min, Identified with: Sequest HT (v1.17); XCorr:3.78, Ions matched by the search engine: 0/0 Fragment match tolerance used for the search: 0.02 Da Fragments used for the search: b; b-H_2O; b-NH_3; y; y-H_2O; y-NH_3; Protein references (1):- Myoglobin OS=Equus caballus OX=9796 GN=MB PE=1 SV=2

Lysozyme Modification.

Lysozyme was purchased from the Sigma-Aldrich Catalog no. (L6876-1G). Protein was used without further purification. Sequence of Lysozyme: It consists of 147 amino acids and contains 7 lysine units.

MRSLLILVLCFLPLAALGKVFGRCELAAAMKRHGLDNYRGYSLGNWVCAAKFESNF
NTQATNRNTDGSTDYGILQINSRWWCNDGRTPGSRNLCNIPCSALLSSDITASVNCAK
KIVSDGNGMNAWVAWRNRCKGTDVQAWIRGCRL

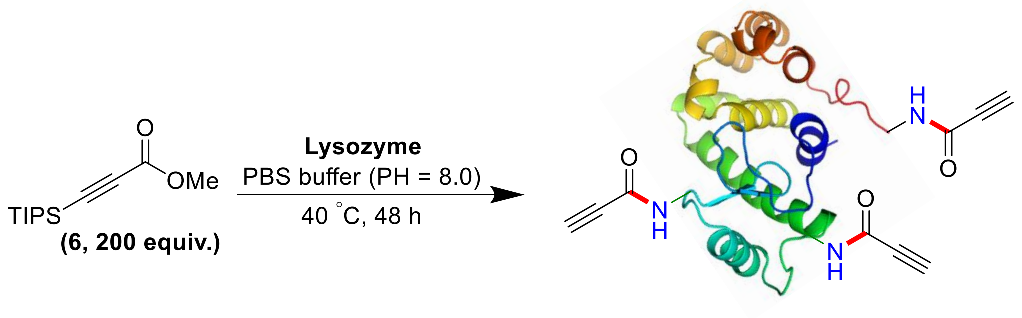

Reaction procedure for Lysozyme modification with *β*-silyl methyl alkynoate (6).

To a 4-mL reaction tube, Lysozyme (6 mg, 0.42 μmol) was added with **6** (200 equiv.) in PBS buffer (pH=8.0) (0.5 mL) solvents. After stirring for 48 hours at 40 °C, a 20 μL aliquot of the reaction mixture was placed for evaluation by performing ESI-MS. A maximum three-fold conjugated adduct with the loss of the TIP (triisopropylsilane) group was detected.

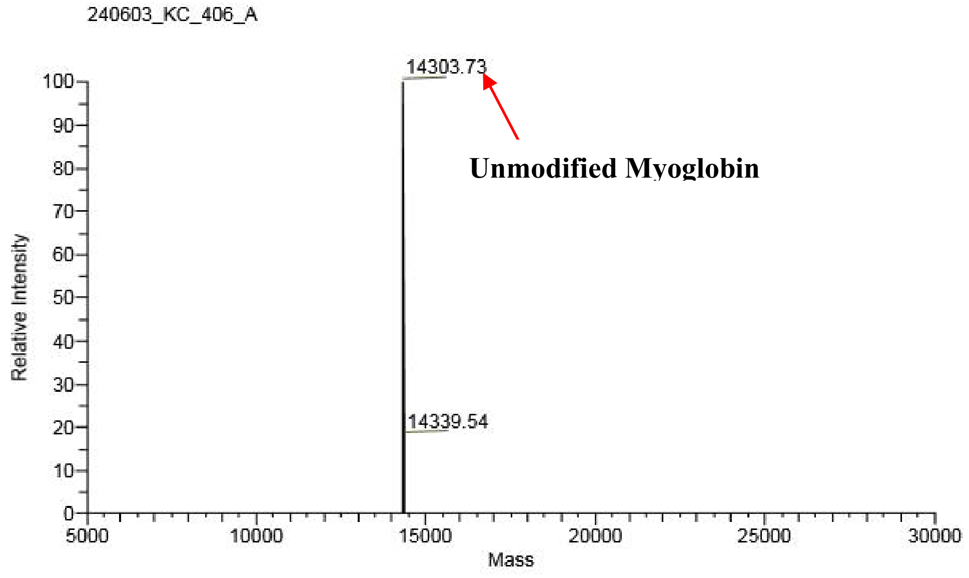

Fig S31. Deconvoluted mass spectrum of unmodified Lysozyme

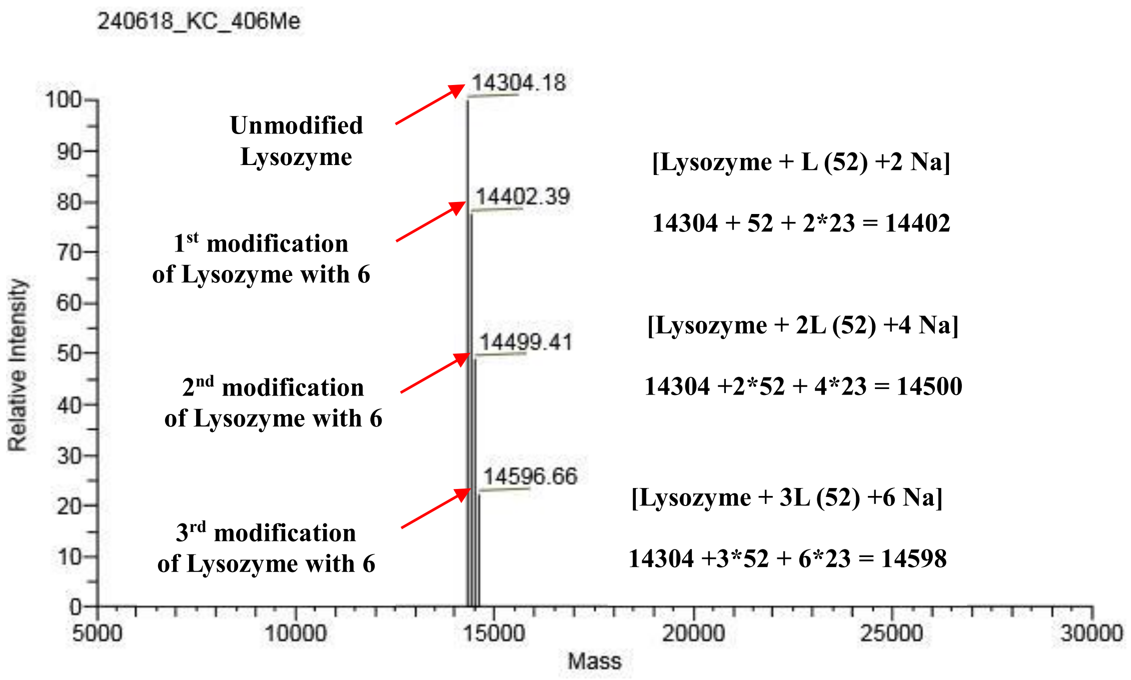

Fig S32. Deconvoluted mass spectrum of modified Lysozyme with β-silyl methyl alkynoate (**6**).

Sliding Windows ReSpect Masses Table

Row Number	Average Mass	Sum Intensity	Relative Abundance	Fractional Abundance	Score	Number of Charge States	Charge State Distribution	Number of Detected Intervals	Mass Std Dev	PPM Std Dev	Delta Mass	Scan Range	Start Time (min)	Stop Time (min)	Apex RT
1	14304.18	6691609.13	100.00	40.29	35.33	8	8 - 15	8	0.86	60.04	0.00	148 - 449	2.293	6.914	5.244
2	14402.39	5174673.38	77.33	31.16	33.73	8	8 - 15	8	0.64	44.37	98.21	148 - 449	2.293	6.914	3.563
3	14499.41	3263127.19	48.76	19.65	27.94	8	8 - 15	7	0.53	36.56	195.23	148 - 423	2.293	6.504	3.137
4	14596.66	1479170.00	22.10	8.91	27.24	5	8 - 12	4	0.33	22.28	292.47	148 - 341	2.293	5.242	3.137

Fig S33. Relative abundance values of components from the modified Lysozyme with **6**.

Reaction procedure for Lysozyme modification with *β*-silyl ethyl alkynoate (1a).

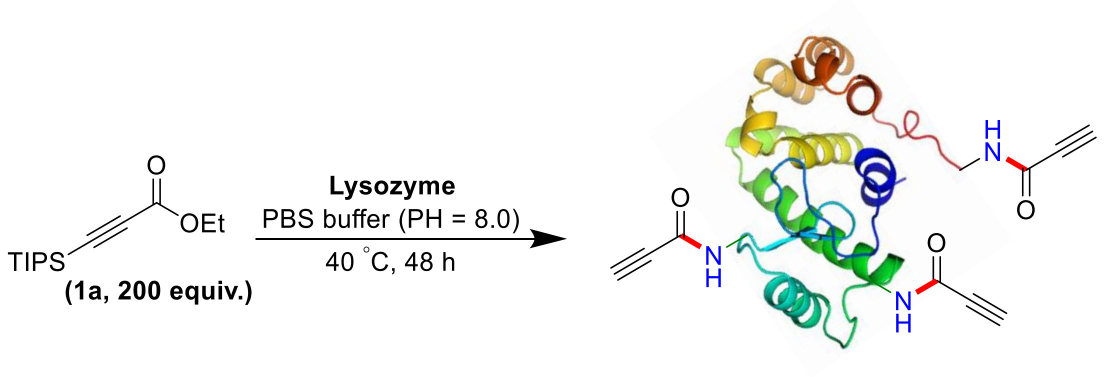

Reaction procedure for Lysozyme modification with *β*-silyl ethyl alkynoate (1a).

To a 4-mL reaction tube, Lysozyme (6 mg, 0.42 μmol) was added with **1a** (200 equiv.) in PBS buffer (pH=8.0) (0.5 mL) solvents. After stirring for 48 hours at 40 °C, a 20 μL aliquot of the reaction mixture was placed for evaluation by performing ESI-MS. A maximum three-fold conjugated adduct with the loss of the TIP (triisopropylsilane) group was detected.

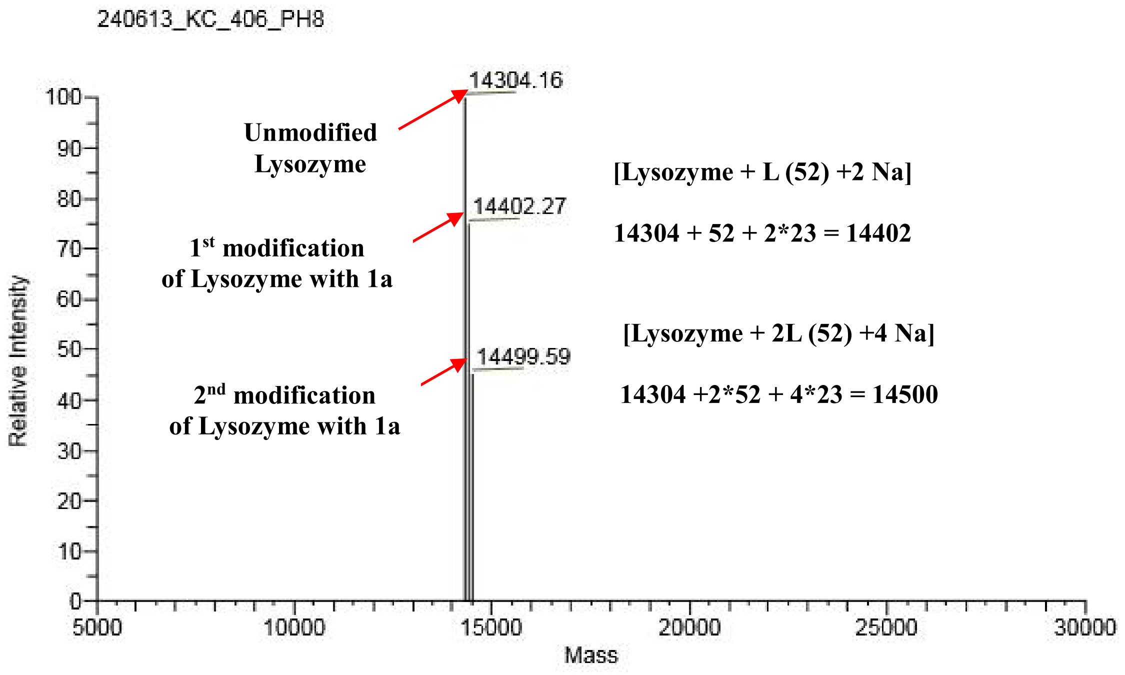

Fig S34. Deconvoluted mass spectrum of modified Lysozyme with *β*-silyl ethyl alkynoate **(1a).**

Sliding Windows ReSpect Masses Table															
Row Number	Average Mass	Sum Intensity	Relative Abundance	Fractional Abundance	Score	Number of Charge States	Charge State Distribution	Number of Detected Intervals	Mass Std Dev	PPM Std Dev	Delta Mass	Scan Range	Start Time (min)	Stop Time (min)	Apex RT
1	14304.16	5038717.91	100.00	45.48	35.04	8	8 - 15	8	0.79	55.45	0.00	63 - 486	1.006	7.715	3.443
2	14402.27	3767068.41	74.76	34.00	33.17	7	8 - 14	8	0.64	44.53	98.10	63 - 486	1.006	7.715	3.443
3	14499.59	2272574.95	45.10	20.51	30.50	8	8 - 15	7	0.51	35.43	195.43	63 - 448	1.006	7.100	2.831

Fig S35. Relative abundance values of components from the modified Lysozyme with **1a**.

Cytochrome C protein modification.

Cytochrome C was purchased from the MedChemExpress Catalog no. (HY – 125857). Protein was used without further purification. Sequence of Cytochrome C: It consists of 117 amino acids and contains 19 lysin units.

MGDVEKGKKIFVQKCAQCHTVEKGGKHKTGPNLHGLFGRKTGQAPGYSYTAANKN
KGIIWGEDTLMEYLADVYEKMKDRNTHEEKYIPGTKMPMIFAGIKKKEERADLIAYL
KKEE

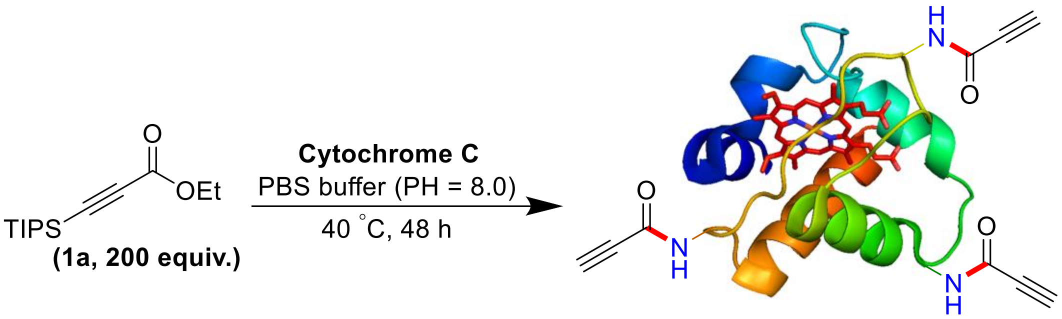

Reaction procedure for Cytochrome C modification with 1a:

To a 4-mL reaction tube, Cytochrome C (6 mg, 0.49 µmol) was added with **6** (200 equiv.) in PBS buffer (pH=8.0) (0.5 mL) solvents. After stirring for 48 hours at 40 °C, a 20 µL aliquot of the reaction mixture was placed for evaluation by performing ESI-MS. A maximum three-fold conjugated adduct with the loss of the TIPS (triisopropylsilane) group was detected.

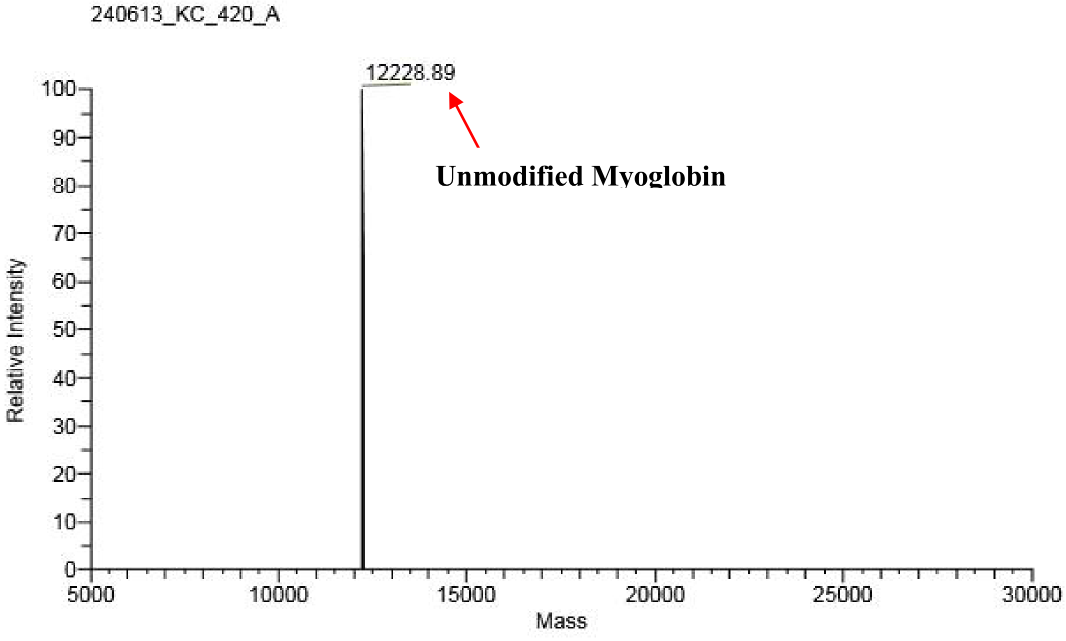

Fig S36. Deconvoluted mass spectrum of unmodified Cytochrome C

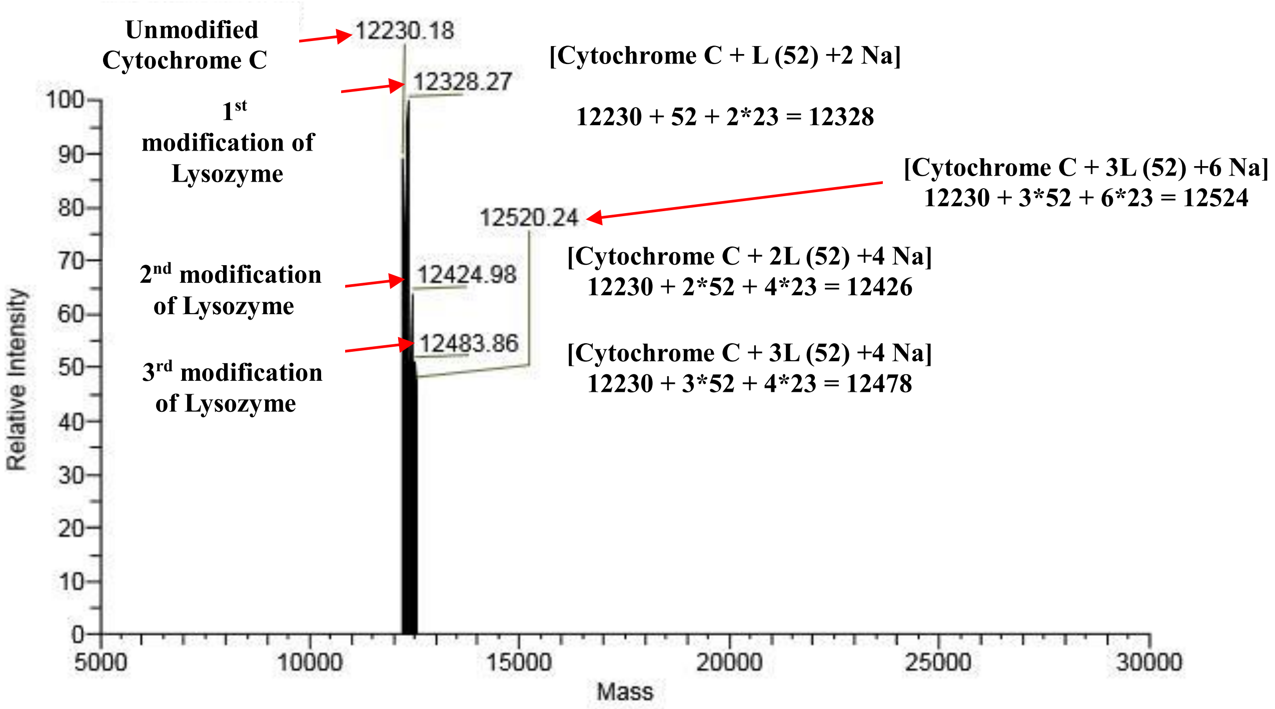

Fig S37. Deconvoluted mass spectrum of modified Cytochrome C with **1a**

Sliding Windows ReSpect Masses Table														
Row Number	Average Mass	Sum Intensity	Relative Abundance	Fractional Abundance	Score	Number of Charge States	Charge State Distribution	Number of Detected Intervals	Mass Std Dev	PPM Std Dev	Delta Mass	Scan Range	Start Time (min)	Stop Time (min)	Apex RT
1	12328.27	13840158.38	100.00	25.98	48.27	15	8 - 22	8	0.65	52.80	0.00	226 - 446	3.495	6.794	4.101
2	12230.18	12327200.88	89.07	23.14	45.03	16	8 - 23	6	0.92	75.09	-98.09	226 - 446	3.495	6.794	4.101
3	12424.98	8797356.56	63.56	16.52	40.64	14	8 - 21	4	0.54	43.37	96.71	226 - 367	3.495	5.598	4.101
4	12483.86	7057656.84	50.99	13.25	43.49	15	8 - 22	6	0.85	68.35	155.59	267 - 446	4.104	6.794	5.303
5	12520.24	6533774.50	47.21	12.27	41.44	15	7 - 21	3	0.42	33.36	191.97	226 - 347	3.495	5.299	4.101
6	12465.23	4711438.91	34.04	8.84	40.08	15	9 - 23	6	0.59	47.03	136.96	267 - 446	4.104	6.794	5.303

Fig S38. Relative abundance values of components from the modified Cytochrome C.

Crystallographic Investigation.

The compounds **3b** were crystallized by the slow evaporation of chloroform and hexane mixture (ca. 30%).

Compound **3b** (CCDC 2310713)

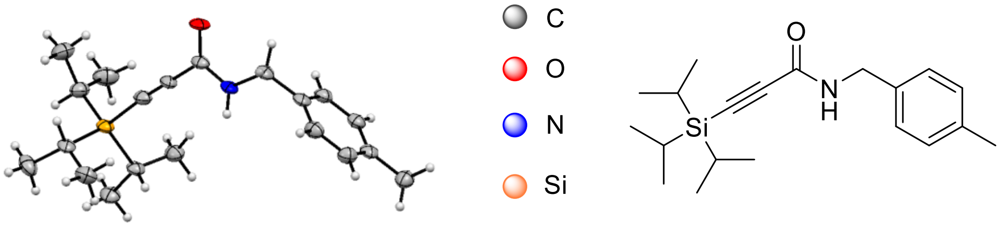

Crystal structure of **3b**

Empirical formula	$C_{20}H_{31}NOSi$	
Formula weight	329.55 g/mol	
Crystal system	monoclinic	
Crystal habit	colourless block	
Space group	P 1 21/c 1	
Unit cell dimensions	a =18.524(2) Å	α = 90°
	b = 12.5421(18) Å	β = 98.355(6)°
	c = 8.5118(10) Å	γ = 90°
Volume	1956.6(4) Å3	
Z	4	
Density (calculated)	1.119 g/cm3	
Crystal size	0.060 x 0.200 x 0.220 mm	
Final R indice [I>2sigma(I)]	R1 = 0.0832, wR2 = 0.1926	
R indices (all data)	R1 = 0.1410, wR2 = 0.2309	

NMR Data for Compounds.

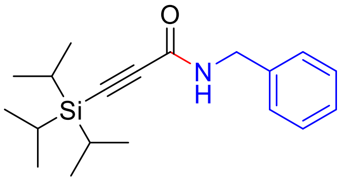

***N*-benzyl-3-(triisopropylsilyl)propiolamide (3a):** Off-white solid, yield 88% (55 mg); ^{1}H NMR (400 MHz, $CDCl_3$) δ 7.39 – 7.28 (m, 5H), 6.07 (s, 1H), 4.66 (d, *J* = 6.4 Hz, 2H, rotamer), 4.49 (d, *J* = 6.0 Hz, 2H), 1.12 – 1.06 (m, 21H); ^{13}C NMR (100 MHz, $CDCl_3$) δ 152.7, 137.5, 128.9, 128.2, 127.9, 99.7, 88.9, 44.0, 18.6, 11.1; HRMS (ESI): Calcd for $C_{19}H_{29}NOSi$ $[M+H]^+$: 316.2097; found: 316.2093.

***N*-(4-methylbenzyl)-3-(triisopropylsilyl)propiolamide (3b):** White solid, yield 99% (64 mg);

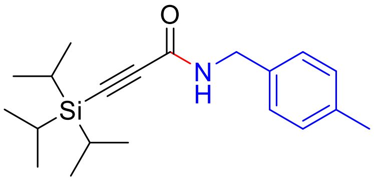

^{1}H NMR (400 MHz, $CDCl_3$, 25:3 mixture of rotamers) δ 7.22 – 7.15 (m, 1H), 6.01 (s, 1H), 4.61 (d, *J* = 6.0 Hz, 2H, rotamers), 4.44 (d, *J* = 6.0 Hz, 2H), 2.35 (s, 3H), 1.13 – 1.03 (m, 21H); ^{13}C NMR (100 MHz, $CDCl_3$) δ 152.7, 137.8, 134.4, 129.6, 128.3, 99.8, 88.8, 43.8, 21.2, 18.6, 11.2; HRMS (ESI): Calcd for $C_{20}H_{31}NOSi$ $[M+Na]^+$: 352.2073; found: 352.2083.

***N*-(4-methoxybenzyl)-3-(triisopropylsilyl)propiolamide (3c):** Off-white solid, yield 90%

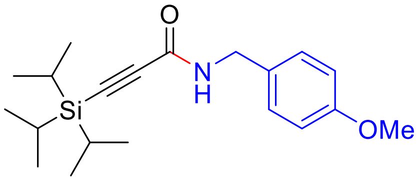

(61 mg); ^{1}H NMR (400 MHz, $CDCl_3$, 25:3 mixture of rotamers) δ 7.25 – 7.18 (m, 2H), 6.91 – 6.85 (m, 2H), 6.01 (s, 1H), 4.59 (d, *J* = 6.4 Hz, 2H, rotamers), 4.41 (d, *J* = 6.0 Hz, 2H), 3.80 (s, 3H), 1.12 – 1.04 (m, 21H); ^{13}C NMR (100 MHz, $CDCl_3$) δ 159.4, 152.6, 129.6, 129.5, 114.3, 99.8, 88.7, 55.4, 43.5, 18.6, 11.1; HRMS (ESI): Calcd for $C_{20}H_{31}NO_2Si$ $[M+Na]^+$: 368.2022; found: 368.2033.

***N*-(4-aminobenzyl)-3-(triisopropylsilyl)propiolamide (3d):** Light yellow solid, yield 95%

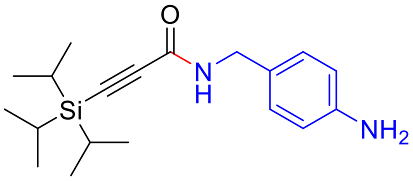

(62 mg); ^{1}H NMR (400 MHz, $CDCl_3$, 50:7 mixture of rotamers) δ 7.10 (d, *J* = 7.8 Hz, 2H), 6.66 (d, *J* = 7.8 Hz, 2H), 5.95 (s, 1H), 4.53 (d, *J* = 6.4 Hz, 2H, rotamers), 4.35 (d, *J* = 5.6 Hz, 2H), 3.69 (s, 2H), 1.09 (d, *J* = 5.6 Hz, 21H); ^{13}C NMR (100 MHz, $CDCl_3$) δ 152.6, 146.3, 129.6, 127.2, 115.4, 99.8, 88.6, 43.7, 18.6, 11.2; HRMS (ESI): Calcd for $C_{19}H_{30}N_2OSi$ $[M+Na]^+$: 353.2025; found: 353.2050.

***N*-(4-chlorobenzyl)-3-(triisopropylsilyl)propiolamide (3e):** White solid, yield 95% (65 mg);

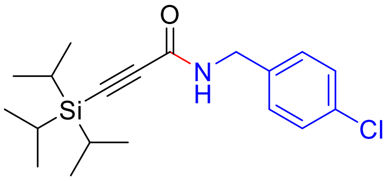

^{1}H NMR (400 MHz, $CDCl_3$, 25:3 mixture of rotamers) δ 7.33 – 7.30 (m, 2H), 7.24 (d, *J* = 8.4 Hz, 2H), 6.11 (s, 1H), 4.63 (d, *J* = 6.4 Hz, 2H, rotamers), 4.44 (d, *J* = 6.4 Hz, 2H), 1.10 – 1.06 (m, 21H); ^{13}C NMR (100 MHz, $CDCl_3$) δ 152.8, 136.1, 133.8,

129.5, 129.1, 99.5, 89.4, 43.3, 18.6, 11.1; HRMS (ESI): Calcd for $C_{19}H_{28}NOClSi$ $[M+H]^+$: 350.1707; found: 350.1705.

***N*-(4-(trifluoromethyl)benzyl)-3-(triisopropylsilyl)propiolamide (3f):** White solid, yield 94% (71 mg); ^{1}H NMR (400 MHz, $CDCl_3$, 50:7 mixture of rotamers) δ 7.60 (d, *J* = 8.0 Hz, 2H), 7.42 (d, *J* = 8.0 Hz, 2H), 6.20 (s, 1H), 4.72 (d, *J* = 6.8 Hz, 2H, rotamers), 4.54 (d, *J* = 6.0 Hz, 2H), 1.12 – 1.06 (m, 21H); ^{13}C NMR (100 MHz, $CDCl_3$) δ 152.9, 141.6, 130.2 (q, *J* = 32.6 Hz), 128.3, 127.2, 125.8 (q, *J* = 3.8 Hz), 99.4, 89.7, 43.4, 18.6, 11.1; ^{19}F NMR (375 MHz, $CDCl_3$) δ -62.6, -62.6 (rotamers); HRMS (ESI): Calcd for $C_{20}H_{28}NOF_3Si$ $[M+H]^+$: 384.1971; found: 384.1956.

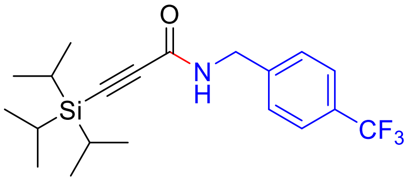

***N*-(2-methylbenzyl)-3-(triisopropylsilyl)propiolamide (3g):** White solid, yield 95% (62 mg); ^{1}H NMR (400 MHz, $CDCl_3$, 25:3 mixture of rotamers) δ 7.26 – 7.24 (m, 1H), 7.24 – 7.17 (m, 3H), 5.87 (s, 1H), 4.64 (d, *J* = 6.4 Hz, 2H, rotamers), 4.49 (d, *J* = 5.6 Hz, 2H), 2.35 (s, 3H), 1.11 – 1.07 (m, 21H); ^{13}C NMR (100 MHz, $CDCl_3$) δ 152.6, 136.8, 135.1, 130.8, 129.1, 128.3, 126.5, 99.7, 88.8, 42.2, 19.2, 18.6, 11.2; HRMS (ESI): Calcd for $C_{20}H_{31}NOSi$ $[M+Na]^+$: 352.2073; found: 352.2090.

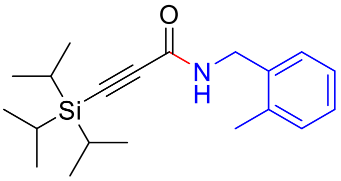

***N*-(2-fluorobenzyl)-3-(triisopropylsilyl)propiolamide (3h):** White solid, yield 69% (46 mg); Recovered starting materials 20% (10 mg); ^{1}H NMR (400 MHz, $CDCl_3$, 25:3 mixture of rotamers) δ 7.37 (td, *J* = 7.5, 1.4 Hz, 1H), 7.33 – 7.26 (m, 1H), 7.13 (t, *J* = 7.6 Hz, 1H), 7.06 (t, *J* = 9.2 Hz, 1H), 6.15 (s, 1H), 4.71 (d, *J* = 6.8 Hz, 2H, rotamers), 4.53 (d, *J* = 6.0 Hz, 2H), 1.10 – 1.06 (m, 21H); ^{13}C NMR (100 MHz, $CDCl_3$) δ 161.16 (d, J_{CF1} = 246.3 Hz), 152.8, 130.7 (d, J_{CF3} = 4.1 Hz), 129.76 (d, J_{CF3} = 8.2 Hz), 124.57 (d, J_{CF4} = 3.5 Hz), 124.5 (d, J_{CF2} = 15.0 Hz), 115.58 (d, J_{CF2} = 21.2 Hz), 99.6, 89.2, 37.8 (d, J_{CF3} = 3.9 Hz), 18.6, 11.2; ^{19}F NMR (375 MHz, $CDCl_3$) δ -118.65 (rotamers), -118.89; HRMS (ESI): Calcd for $C_{19}H_{28}NOFSi$ $[M+Na]^+$: 356.1822; found: 356.1830.

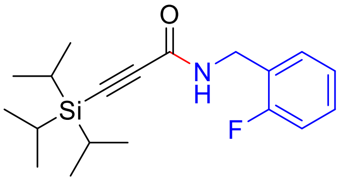

***N*-(3,5-bis(trifluoromethyl)benzyl)-3-(triisopropylsilyl)propiolamide (3i):** White solid, yield 98% (87 mg); ^{1}H NMR (400 MHz, $CDCl_3$, 25:3 mixture of rotamers) δ 7.81 (s, 1H), 7.76 (s, 2H), 6.26 (s, 1H), 4.80 (d, *J* = 6.8 Hz, 2H, rotamer), 4.60 (d, *J* = 6.4 Hz, 2H), 1.17 – 1.06 (m, 21H); 13C NMR (100 MHz, $CDCl_3$) δ 153.0, 140.3, 132.2 (q, J_{CF} = 32.0 Hz), 128.2, 128.2, 124.6, 121.9 (q, J_{CF} = 3.7 Hz), 99.0, 90.5, 43.0, 18.6, 11.1; ^{19}F NMR (375 MHz, $CDCl_3$) δ -62.87, -62.95 (rotamer); HRMS (ESI): Calcd for $C_{21}H_{27}NOF_6Si$ $[M+H]^+$: 452.1844; found: 452.1844.

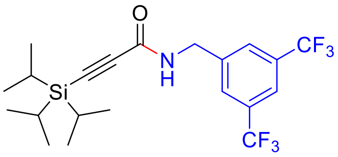

N-(thiophen-2-ylmethyl)-3-(triisopropylsilyl)propiolamide (3j): Off-white solid, yield 48% (30 mg); Recover starting materials 35% (17.5 mg); ^{1}H NMR (400 MHz, $CDCl_3$, 25:3 mixture of rotamers) δ 7.25 (dd, J = 5.2, 1.2 Hz, 1H), 7.01 (d, J = 2.8 Hz, 1H), 6.97 (dd, J = 5.2, 3.2 Hz, 1H), 6.15 (s, 1H), 4.82 (d, J = 6.4 Hz, 2H, rotamer), 4.64 (d, J = 5.6 Hz, 2H), 1.10 – 1.07 (m, 21H); ^{13}C NMR (100 MHz, $CDCl_3$) δ 152.5, 139.7, 127.2, 126.8, 125.7, 99.5, 89.3, 38.5, 18.6, 11.1; HRMS (ESI): Calcd for $C_{17}H_{27}NOSSi$ $[M+Na]^+$: 344.1480; found: 344.1472.

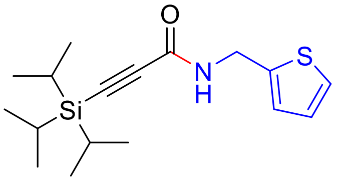

N-(pyridin-2-ylmethyl)-3-(triisopropylsilyl)propiolamide (3k): Off-white solid, yield 55% (34 mg); Recover starting materials 31% (15.5 mg); ^{1}H NMR (400 MHz, $CDCl_3$, 25:2 mixture of rotamers) δ 8.56 (d, J = 4.4 Hz, 1H), 7.68 (td, J = 7.6, 1.6 Hz, 1H), 7.28 (d, J = 7.6 Hz, 1H), 7.24 – 7.19 (m, 1H), 7.13 (s, 1H), 4.77 (d, J = 5.2 Hz, 2H, rotamers), 4.60 (d, J = 5.2 Hz, 2H), 1.13 – 1.07 (m, 21H); ^{13}C NMR (100 MHz, $CDCl_3$,) δ 155.6, 152.9, 149.3, 137.1, 122.7, 122.4, 99.8, 88.8, 44.8, 18.6, 11.2**;** HRMS (ESI): Calcd for $C_{18}H_{28}N_2OSi$ $[M+Na]^+$: 339.1869; found: 339.1857.

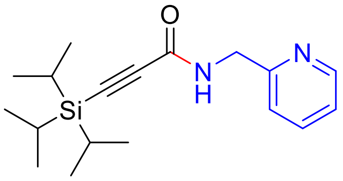

N-methyl-3-(triisopropylsilyl)propiolamide (3l): Viscus liquid, yield 95% (45 mg); ^{1}H NMR (400 MHz, $CDCl_3$, 10:1 mixture of rotamers) δ 5.85 (s, 1H), 3.06 (d, J = 5.2 Hz, 3H, rotamer), 2.86 (d, J = 5.2 Hz, 3H), 1.10 (m, 21H); ^{13}C NMR (100 MHz, $CDCl_3$, Major) δ 153.6, 99.9, 88.2, 26.6, 18.6, 11.1; HRMS (ESI): Calcd for $C_{13}H_{25}NOSi$ $[M+H]^+$: 240.1784; found: 240.1785.

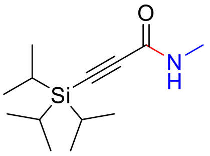

N-ethyl-3-(triisopropylsilyl)propiolamide (3m): Viscus liquid, yield 93% (46 mg); ^{1}H NMR (400 MHz, $CDCl_3$, 50:9 mixture of rotamers) δ 5.79 (s, 1H), 3.53 – 3.43 (m, 2H, rotamers), 3.39 – 3.29 (m, 2H), 1.18 (t, J = 7.2 Hz, 3H), 1.11 – 1.06 (m, 21H); ^{13}C NMR (100 MHz, $CDCl_3$) δ 152.8, 100.1, 87.9, 34.9, 18.6, 14.7, 11.1; HRMS (ESI): Calcd for $C_{14}H_{27}NOSi$ $[M+H]^+$: 254.1940; found: 254.1933.

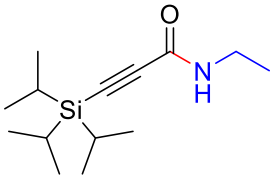

N-(2-(1H-indol-3-yl)ethyl)-3-(triisopropylsilyl)propiolamide (3n): off-white solid, yield 82% (60 mg); ^{1}H NMR (400 MHz, $CDCl_3$, 25:4 mixture of rotamers) δ 8.07 (s, 1H), 7.62 (d, J = 8.0 Hz, 1H), 7.39 (d, J = 8.0 Hz, 1H), 7.24 – 7.18 (m, 1H), 7.16 – 7.10 (m, 1H), 7.07 (d, J = 2.0 Hz, 1H), 5.88 (s, 1H), 3.78 (q, J = 6.8 Hz, 2H, rotamer), 3.64 (q, J = 6.8 Hz, 2H), 3.02 (t, J = 6.8 Hz, 2H), 1.13 – 1.10 (m, 3H), 1.08 – 1.05 (m, 18H); ^{13}C NMR (100 MHz, $CDCl_3$, Major) δ 152.9,

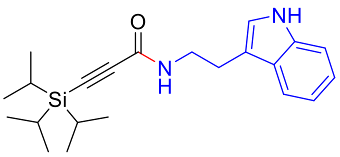

136.5, 127.4, 122.4, 122.3, 119.7, 118.8, 112.8, 111.4, 100.0, 88.4, 40.4, 25.2, 18.6, 11.1; (minor) δ 152.9, 136.6, 127.1, 122.5, 122.4, 119.7, 118.6, 112.8, 111.5, 97.5, 95.3, 43.7, 26.7, 18.7, 11.2; HRMS (ESI): Calcd for $C_{22}H_{32}N_2OSi$ $[M+H]^+$: 369.2362; found: 369.2375.

***N*-(4-fluorophenethyl)-3-(triisopropylsilyl)propiolamide (3o):** White solid, yield 93% (64 mg); ^1H NMR (400 MHz, $CDCl_3$, 50:9 mixture of rotamers) δ 7.18 – 7.12 (m, 2H), 7.02 – 6.97 (m, 2H), 5.78 (s, 1H), 3.69 (q, *J* = 6.8 Hz, 2H, rotamers), 3.51 (dd, *J* = 13.6, 7.2 Hz, 2H), 2.82 (t, *J* = 7.2 Hz, 2H), 1.13 – 1.06 (m, 21H); 13C NMR (100 MHz, $CDCl_3$) δ 161.86 (d, J_{CF1} = 244.7 Hz), 152.8, 134.27 (d, J_{CF4} = 3.2 Hz), 130.32 (d, J_{CF3} = 8.0 Hz), 115.59 (d, J_{CF2} = 21.2 Hz), 99.9, 88.5, 41.2, 34.7, 18.6, 11.1; ^{19}F NMR (375 MHz, $CDCl_3$) δ -116.1 (rotamers), -116.4; HRMS (ESI): Calcd for $C_{20}H_{30}NOFSi$ $[M+Na]^+$: 370.1978; found: 370.1964.

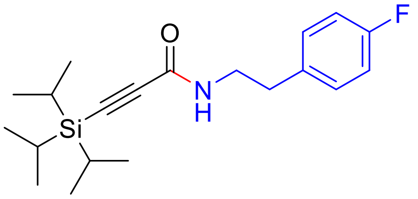

***N*-propyl-3-(triisopropylsilyl)propiolamide (3p):** White solid, yield 82% (43 mg); ^1H NMR (400 MHz, DMSO, 50:9 mixture of rotamers) δ 8.59 (t, *J* = 5.4 Hz, 1H), 3.23 (dd, *J* = 13.6, 6.8 Hz, 2H, rotamers), 3.02 (dd, *J* = 13.6, 6.8 Hz, 2H), 1.51 – 1.36 (m, 1H), 1.09 – 1.05 (m, 21H), 0.86 – 0.80 (t, *J* = 7.3 Hz, 3H); ^{13}C NMR (100 MHz, DMSO) δ 151.6, 101.1, 85.1, 40.7, 21.9, 18.3, 11.4, 10.5; HRMS (ESI): Calcd for $C_{15}H_{29}NOSi$ $[M+H]^+$: 268.2097; found: 268.2094.

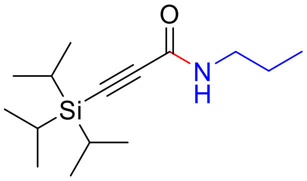

***N*-(3-phenylpropyl)-3-(triisopropylsilyl)propiolamide (3q):** White solid, yield 90% (61 mg); ^1H NMR (400 MHz, $CDCl_3$, 50:9 mixture of rotamers) δ 7.32 – 7.26 (m, 2H), 7.22 – 7.14 (m, 3H), 5.73 (s, 1H), 3.47 (dd, *J* = 13.6, 7.2 Hz, 2H, rotamers), 3.33 (dd, *J* = 13.6, 7.2 Hz, 2H), 2.67 (t, *J* = 7.6 Hz, 2H), 1.89 (dt, *J* = 14.8, 7.6 Hz, 2H), 1.16 – 1.03 (m, 21H); ^{13}C NMR (100 MHz, $CDCl_3$) δ 152.9, 141.3, 128.6, 128.5, 126.2, 99.9, 88.1, 39.6, 33.4, 30.9, 18.6, 11.1; HRMS (ESI): Calcd for $C_{21}H_{33}NOSi$ $[M+Na]^+$: 366.2229; found: 366.2215.

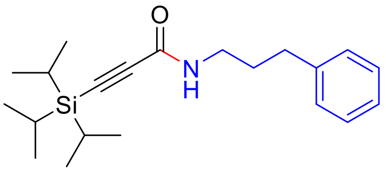

***N*-(4-phenylbutyl)-3-(triisopropylsilyl)propiolamide (3r):** White solid, yield 72% (51 mg); ^1H NMR (400 MHz, $CDCl_3$, 50:9 mixture of rotamers) δ 7.31 – 7.26 (m, 2H), 7.21 – 7.13 (m, 3H), 5.74 (s, 1H), 3.45 (q, J = 6.8 Hz, 2H, rotamer), 3.31 (dd, J = 13.3, 7.0 Hz, 2H), 2.64 (dd, J = 9.6, 5.2 Hz, 2H), 1.72 – 1.63 (m, 2H), 1.62 – 1.53 (m, 2H), 1.19 – 0.94 (m, 21H); ^{13}C NMR (100 MHz, $CDCl_3$, Major) δ 152.9, 142.1, 128.6, 128.5,

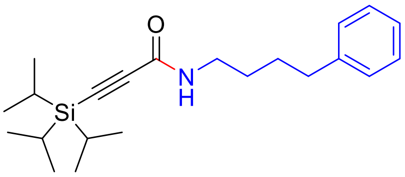

126.0, 100.0, 88.2, 39.9, 35.5, 29.0, 28.7, 18.6, 11.2; (minor) δ 155.6, 141.9, 128.54, 128.4, 126.1, 97.4, 95.2, 43.5, 35.6, 30.6, 28.6, 18.6, 11.2; HRMS (ESI): Calcd for $C_{22}H_{35}NOSi$ $[M+H]^+$: 358.2566; found: 358.2580.

***N*-octyl-3-(triisopropylsilyl)propiolamide (3s):** Viscus liquid, yield 86% (57 mg); ^{1}H NMR (400 MHz, $CDCl_3$, 50:11 mixture of rotamers) δ 5.82 (s, 1H), 3.42 (dd, *J* = 14.0, 6.8 Hz, 2H, rotamers), 3.27 (dd, *J* = 13.6, 6.8 Hz, 2H), 1.57 – 1.47 (m, 2H), 1.33 – 1.23 (m, 10H), 1.10 – 1.07 (m, 21H), 0.87 (t, *J* = 6.8 Hz, 3H); ^{13}C NMR (100 MHz, $CDCl_3$) δ 152.8, 100.1, 87.9, 40.1, 31.9, 29.4, 29.3, 29.3, 27.0, 22.7, 18.6, 14.2, 11.1; HRMS (ESI): Calcd for $C_{20}H_{39}NOSi$ $[M+Na]^+$: 360.2699; found: 360.2705.

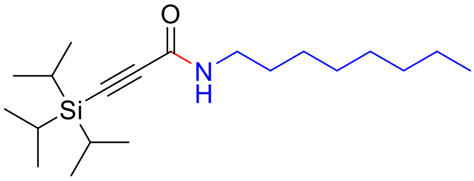

***N*-(1-phenylethyl)-3-(triisopropylsilyl)propiolamide (3t):** White solid, yield 56% (37 mg); Recover starting materials 34% (17 mg); ^{1}H NMR (400 MHz, $CDCl_3$, 5:1 mixture of rotamers) δ 7.39 – 7.32 (m, 4H), 7.31 – 7.27 (m, 1H), 5.97 (d, *J* = 6.8 Hz, 1H), 5.22 – 5.13 (m, 1H), 1.59 (d, *J* = 6.8 Hz, 3H, rotamer), 1.54 (d, *J* = 6.8 Hz, 3H), 1.11 – 1.06 (m, 21H); ^{13}C NMR (100 MHz, $CDCl_3$) δ 151.9, 142.4, 128.9, 127.8, 126.5, 99.9, 88.5, 49.4, 21.5, 18.7, 11.1; HRMS (ESI): Calcd for $C_{20}H_{31}NOSi$ $[M+Na]^+$: 352.2073; found: 352.2080.

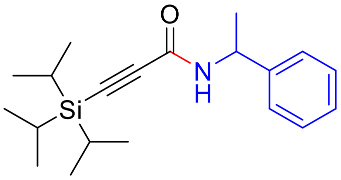

(*R*)-*N*-(2,3-dihydro-1H-inden-1-yl)-3-(triisopropylsilyl)propiolamide (3u): Off-white solid, yield 84% (57 mg); ^{1}H NMR (400 MHz, $CDCl_3$, 25:3 mixture of rotamers) δ 7.40 – 7.31 (m, 1H), 7.28 – 7.21 (m, 3H), 5.99 (d, *J* = 7.6 Hz, 1H), 5.78 (d, *J* = 7.6 Hz, 1H, rotamers), 5.60 – 5.45 (m, 1H), 3.07 – 2.96 (m, 1H), 2.94 – 2.80 (m, 1H), 2.70 – 2.56 (m, 1H), 1.97 – 1.81 (m, 1H), 1.16 – 1.04 (m, 21H); ^{13}C NMR (100 MHz, $CDCl_3$) δ 152.6, 143.7, 142.5, 128.4, 127.0, 125.0, 124.4, 99.8, 88.7, 55.2, 33.9, 30.4, 18.6, 11.2; HRMS (ESI): Calcd for $C_{21}H_{31}NOSi$ $[M+Na]^+$: 364.2073; found: 364.2064.

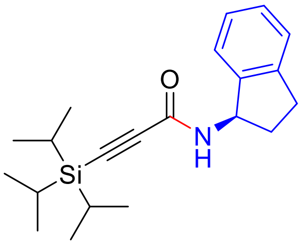

***N*-(sec-butyl)-3-(triisopropylsilyl)propiolamide (3v):** White solid, yield 46% (26 mg); Recover starting materials 37% (18.5 mg); ^{1}H NMR (400 MHz, $CDCl_3$, 25:2 mixture of rotamers) δ 5.54 (s, 1H), 4.01 – 3.83 (m, 1H), 1.56 – 1.44 (m, 2H), 1.16 (d, J = 6.6 Hz, 3H), 1.12 – 1.06 (m, 21H), 0.93 (q, J = 7.2 Hz, 3H); ^{13}C NMR (100 MHz, $CDCl_3$, Major) δ 152.3, 100.2, 87.7, 47.4, 29.6, 20.3, 18.6, 11.2, 10.5; ^{13}C NMR (100 MHz, $CDCl_3$, Minor) δ 154.9, 97.8, 94.7, 51.5, 30.7, 21.6, 18.6, 11.1, 10.5; HRMS (ESI): Calcd for $C_{16}H_{31}NOSi$ $[M+Na]^+$: 304.2073; found: 304.2069.

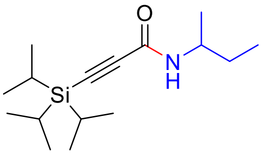

N-cyclohexyl-3-(triisopropylsilyl)propiolamide (3w): White solid, yield 98% (59 mg); ^{1}H NMR (400 MHz, $CDCl_3$, 25:3 mixture of rotamers) δ 5.60 (s, 1H), 3.87 – 3.72 (m, 1H), 2.03 – 1.90 (m, 2H), 1.81 – 1.69 (m, 2H), 1.67 – 1.57 (m, 3H), 1.43 – 1.20 (m, 3H), 1.19 – 1.04 (m, 21H); ^{13}C NMR (100 MHz, $CDCl_3$, Major) δ 151.9, 100.3, 87.7, 48.9, 33.0, 25.6, 24.9, 18.7, 11.2; ^{13}C NMR (minor) δ 154.6, 97.8, 94.3, 52.7, 34.2, 25.3, 24.9, 18.6, 11.2; HRMS (ESI): Calcd for $C_{18}H_{33}NOSi$ $[M+Na]^+$: 330.2229; found: 330.2226.

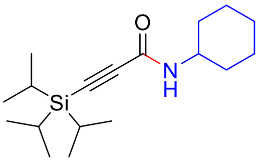

N-(2-hydroxyethyl)-3-(triisopropylsilyl)propiolamide (3x): Colourless liquid, yield 68% (36 mg); Recovered starting materials 20% (10 mg) ^{1}H NMR (400 MHz, $CDCl_3$, 25:3 mixture of rotamers) δ 6.38 (s, 1H), 3.82 – 3.73 (m, 2H), 3.62 (dd, J = 10.8, 5.2 Hz, 2H, rotamers), 3.49 (dd, J = 10.8, 5.2 Hz, 2H), 2.52 (s, 1H), 1.14 – 1.09 (m, 21H); ^{13}C NMR (100 MHz, $CDCl_3$) δ 153.7, 99.5, 89.3, 61.6, 42.6, 18.6, 11.1; HRMS (ESI): Calcd for $C_{14}H_{27}NO_2Si$ $[M+Na]^+$: 292.1709; found: 292.1718.

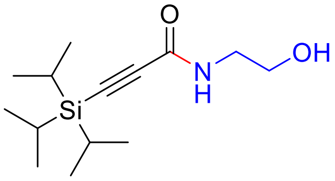

N-(3-hydroxypropyl)-3-(triisopropylsilyl)propiolamide (3y): Colourless liquid, yield 97% (54 mg); ^{1}H NMR (400 MHz, $CDCl_3$, 50:2 mixture of rotamers) δ 6.20 (s, 1H), 3.77 (q, J = 5.6 Hz, 2H, rotamer), 3.70 (q, J = 5.6 Hz, 2H), 3.61 (dd, J = 13.0, 6.5 Hz, 2H, rotamer), 3.47 (dd, J = 12.4, 6.4 Hz, 2H), 2.58 (s, 1H), 1.74 (dt, J = 11.6, 6.0 Hz, 2H), 1.12 – 1.06 (m, 21H); ^{13}C NMR (100 MHz, $CDCl_3$, Major) δ 153.8, 99.6, 89.0, 59.8, 37.1, 32.0, 18.6, 11.1; HRMS (ESI): Calcd for $C_{15}H_{29}NO_2Si$ $[M+Na]^+$: 306.1865; found: 306.1873.

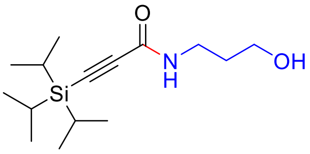

(R)-N-(2-hydroxy-2-phenylethyl)-3-(triisopropylsilyl)propiolamide (3z): White solid, yield 60% (41 mg); Recovered starting materials 20% (10 mg); ^{1}H NMR (400 MHz, $CDCl_3$) δ 7.40 – 7.35 (m, 4H), 7.34 – 7.29 (m, 1H), 6.24 (s, 1H), 4.93 – 4.83 (m, 1H), 3.80 – 3.71 (m, 1H), 3.39 – 3.28 (m, 1H), 2.81 – 2.68 (m, 1H), 1.23 – 0.97 (m, 21H); ^{13}C NMR (100 MHz, $CDCl_3$) δ 153.7, 141.5, 128.8, 128.3, 125.9, 99.6, 89.6, 73.4, 47.5, 18.6, 11.2; HRMS (ESI): Calcd for $C_{20}H_{31}NO_2Si$ $[M+Na]^+$: 368.2022; found: 368.1999.

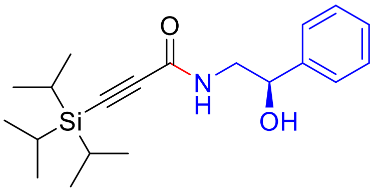

(*S*)-N-(1-hydroxy-4-methylpentan-2-yl)-3-(triisopropylsilyl)propiolamide (3ab): Off-white solid, yield 86% (55 mg); ^{1}H NMR (400 MHz, $CDCl_3$) δ 5.90 (s, 1H), 4.14 – 4.01 (m, 1H), 3.76 – 3.67 (m, 1H), 3.63 – 3.54 (m, 1H), 2.42 (s, 1H), 1.72 – 1.59 (m, 1H), 1.50 – 1.31 (m, 2H), 1.16 – 1.03 (m, 21H), 0.94 (dd, *J* = 6.4, 4.4 Hz, 6H); ^{13}C NMR (100 MHz, $CDCl_3$) δ 153.3, 99.7, 89.1, 65.5, 50.5, 40.2, 25.0, 23.1, 22.4, 18.6, 11.2; HRMS (ESI): Calcd for $C_{18}H_{35}NO_2Si$ [M+Na]$^+$: 348.2335; found: 348.2341.

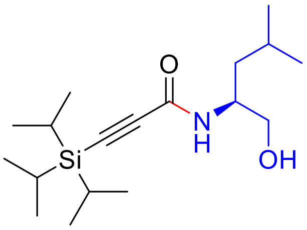

1-(piperidin-1-yl)-3-(triisopropylsilyl)prop-2-yn-1-one (3ac): Colourless liquid, yield 80% (46 mg); ^{1}H NMR (400 MHz, $CDCl_3$) δ 3.77 – 3.72 (m, 2H), 3.60 – 3.54 (m, 2H), 1.72 – 1.50 (m, 6H), 1.18 – 1.00 (m, 21H); ^{13}C NMR (100 MHz, $CDCl_3$) δ 152.3, 98.2, 93.9, 48.3, 42.3, 26.4, 25.4, 24.6, 18.6, 11.1; HRMS (ESI): Calcd for $C_{17}H_{31}NOSi$ [M+Na]$^+$: 294.2253; found: 294.2255.

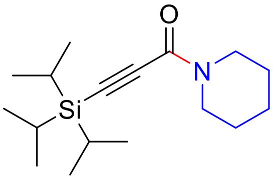

3-(triisopropylsilyl)-N-(2-(4-(3-(triisopropylsilyl)propioloyl)piperazin-1-yl)ethyl) propiolamide (3ad): Semi solid, yield 92% (49 mg); ^{1}H NMR (400 MHz, $CDCl_3$) δ 6.30 (s, 1H), 3.83 – 3.76 (m, 2H), 3.68 – 3.59 (m, 2H), 3.53 (dd, *J* = 11.6, 5.6 Hz, 2H, rotamer), 3.39 (q, *J* = 6.0 Hz, 2H), 2.57 – 2.42 (m, 7H), 1.11 – 1.06 (m, 42H); ^{13}C NMR (100 MHz, $CDCl_3$) δ 152.8, 152.4, 99.9, 97.6, 95.0, 88.5, 56.1, 52.8, 52.5, 47.1, 41.4, 36.4, 18.6, 18.6, 11.1; HRMS (ESI): Calcd for $C_{30}H_{55}N_3O_2Si_2$ [M+Na]$^+$: 568.3731; found: 568.3740.

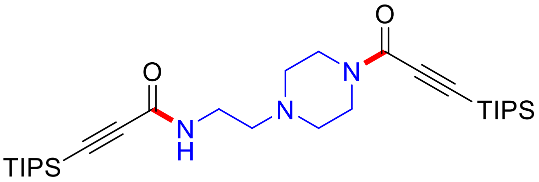

Tert-butyl N2-(tert-butoxycarbonyl)-N6-(3-(triisopropylsilyl)propioloyl)-L-lysinate (3ae): Viscus liquid, yield 88% (88 mg); ^{1}H NMR (400 MHz, $CDCl_3$, 25:4 mixture of rotamers) δ 5.89 (s, 1H), 5.04 (d, *J* = 8.0 Hz, 1H), 4.14 (d, *J* = 5.6 Hz, 1H), 3.41 (q, *J* = 6.8 Hz, 2H, rotamer), 3.27 (td, *J* = 7.2, 2.0 Hz, 2H), 1.81 – 1.73 (m, 1H), 1.63 – 1.51 (m, 3H), 1.44 (s, 9H), 1.42 (s, 9H), 1.39 – 1.28 (m, 2H), 1.09 – 1.06 (m, 21H); ^{13}C NMR (100 MHz, $CDCl_3$) δ 171.9, 155.5, 152.9, 99.9, 88.1, 82.0, 79.8, 53.8, 39.7, 32.8, 29.0, 28.4, 28.1, 22.7, 18.6, 11.1; HRMS (ESI): Calcd for $C_{27}H_{50}N_2O_5Si$ [M+Na]$^+$: 533.3387; found: 533.3389.

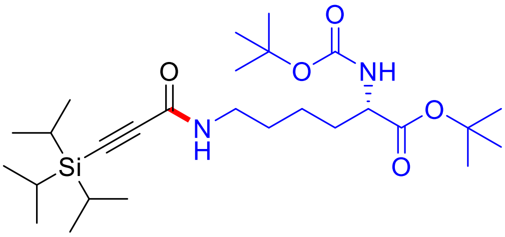

***N*-(((1R,4aS,10aR)-7-isopropyl-1,4a-dimethyl-1,2,3,4,4a,9,10,10a octahydrophenanthren-1-yl)methyl)-3-(triisopropylsilyl)propiolamide (3af):** White solid, yield 80% (78 mg); ^{1}H NMR (400 MHz, $CDCl_3$, 25:4 mixture of rotamers) δ 7.17 (t, J = 8.8 Hz, 1H), 7.00 (dd, J = 8.0, 1.6 Hz, 1H), 6.93 – 6.85 (m, 1H), 5.73 (t, J = 6.0 Hz, 1H), 3.53 – 3.37 (m, 2H, rotamers), 3.28 – 3.14 (m, 2H), 2.91 – 2.80 (m, 2H), 2.29 (d, J = 13.2 Hz, 1H), 1.82 – 1.63 (m, 5H), 1.51 – 1.37 (m, 4H), 1.26 – 1.19 (m, 9H), 1.14 – 1.05 (m, 21H), 0.96 (s, 3H); ^{13}C NMR (100 MHz, $CDCl_3$) δ 153.2, 147.2, 145.8, 134.9, 127.1, 124.3, 124.0, 100.0, 88.7, 54.9, 50.4, 45.5, 38.4, 37.7, 37.6, 36.3, 33.6, 30.3, 25.4, 24.1, 19.1, 18.8, 18.6, 11.1; HRMS (ESI): Calcd for $C_{32}H_{51}NOSi$ $[M+Na]^+$: 516.3638; found: 516.3636.

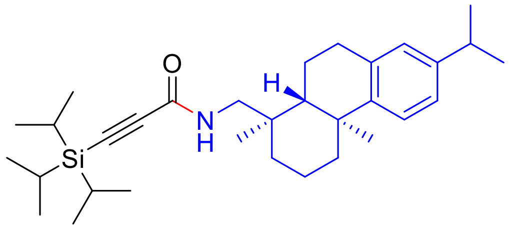

3-Ethyl 5-methyl 4-(2-chlorophenyl)-6-methyl-2-((2-(3 (triisopropylsilyl)propiolamido) ethoxy)methyl)-1,4-dihydropyridine-3,5-dicarboxylate (3ag): Off-white solid, yield 94% (114 mg); ^{1}H NMR (400 MHz, $CDCl_3$) δ 7.37 (dd, J = 7.6, 1.6 Hz, 1H), 7.25 – 7.16 (m, 2H), 7.12 (td, J = 7.6, 1.2 Hz, 1H), 7.02 (td, J = 7.6, 1.6 Hz, 1H), 6.23 (t, J = 5.2 Hz, 1H), 5.39 (s, 1H), 4.81 – 4.63 (m, 2H), 4.10 – 3.96 (m, 2H), 3.70 – 3.61 (m, 3H), 3.60 (s, 3H), 3.56 – 3.43 (m, 1H), 2.36 (s, 3H), 1.17 (t, J = 7.2 Hz, 3H), 1.12 – 1.05 (m, 21H); ^{13}C NMR (100 MHz, $CDCl_3$) δ 168.2, 167.3, 153.5, 145.9, 145.1, 144.6, 132.4, 131.6, 129.3, 127.4, 127.0, 103.9, 101.7, 99.6, 89.4, 70.2, 68.1, 59.9, 50.8, 39.6, 37.2, 19.4, 18.6, 14.4, 11.1; HRMS (ESI): Calcd for $C_{32}H_{45}N_2O_6ClSi$ $[M+H]^+$: 617.2814; found: 617.2818.

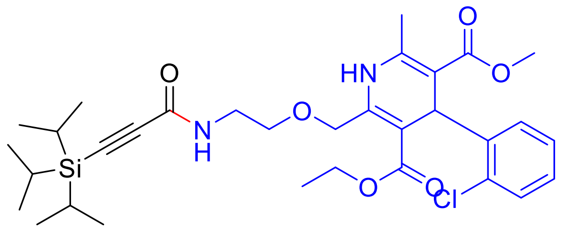

***N*-(4-((6-methoxyquinolin-8-yl)amino)pentyl)-3-(triisopropylsilyl)propiolamide (3ah):** Brown colour liquid, yield 88% (81 mg); ^{1}H NMR (400 MHz, $CDCl_3$) δ 8.52 (dd, J = 4.4, 1.6 Hz, 1H), 7.92 (dd, J = 8.4, 1.6 Hz, 1H), 7.30 (dd, J = 8.4, 4.4 Hz, 1H), 6.34 (d, J = 2.4 Hz, 1H), 6.29 – 6.25 (m, 1H), 5.99 (d, J = 8.4 Hz, 1H), 5.91 (s, 1H), 3.88 (s, 3H), 3.63 (dd, J = 8.0, 6.4 Hz, 1H), 3.50 – 3.21 (m, 2H), 1.79 – 1.62 (m, 4H), 1.30 (d, J = 6.4 Hz, 3H), 1.13 – 1.02 (m, 21H); ^{13}C NMR (100 MHz, $CDCl_3$) δ 159.6, 152.9, 145.0, 144.4, 135.5, 134.9, 130.0, 121.9, 99.9, 96.9, 91.9, 88.2, 55.3, 47.9, 40.0, 34.2, 26.3, 20.7, 18.6, 11.1; HRMS (ESI): Calcd for $C_{27}H_{41}N_3O_2Si$ $[M+Na]^+$: 490.2866; found: 490.2857.

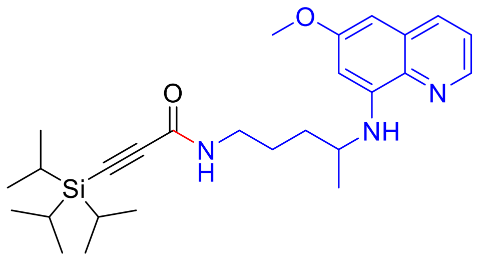

N-(1-(2,6-dimethylphenoxy)propan-2-yl)-3-(triisopropylsilyl)propiolamide (3ai): White solid, yield 40% (81 mg); Recovered starting material 41% (20 mg); ^{1}H NMR (400 MHz, $CDCl_3$) δ 7.01 (d, J = 7.6 Hz, 2H), 6.93 (dd, J = 8.4, 6.4 Hz, 1H), 6.28 (d, J = 8.4 Hz, 1H), 4.48 – 4.29 (m, 1H), 3.81 (dd, J = 9.2, 3.6 Hz, 1H), 3.75 (dd, J = 9.2, 3.6 Hz, 1H), 2.27 (s, 6H), 1.45 (d, J = 6.8 Hz, 3H), 1.17 – 1.02 (m, 21H); ^{13}C NMR (100 MHz, $CDCl_3$) δ 154.8, 152.3, 130.9, 129.2, 124.4, 99.9, 88.4, 73.6, 45.9, 18.6, 17.6, 16.3, 11.2; HRMS (ESI): Calcd for $C_{23}H_{37}NO_2Si$ $[M+Na]^+$: 410.2491; found: 410.2485.

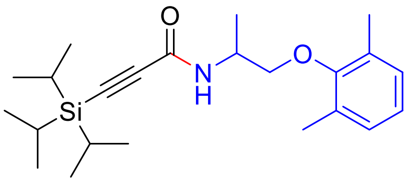

(S)-N-((3-(3-fluoro-4-morpholinophenyl)-2-oxooxazolidin-5-yl)methyl)-3-(triisopropylsilyl)propiolamide (3aj): White solid, yield 47% (47 mg); Recovered starting materials 37% (18.5 mg); ^{1}H NMR (400 MHz, $CDCl_3$) δ 7.41 (dd, J = 14.4, 2.4 Hz, 1H), 7.10 – 7.03 (m, 1H), 6.90 (t, J = 9.2 Hz, 1H), 6.71 – 6.54 (m, 1H), 4.83 – 4.72 (m, 1H), 4.03 (t, J = 9.0 Hz, 1H), 3.88 – 3.82 (m, 4H), 3.79 – 3.70 (m, 2H), 3.65 – 3.53 (m, 1H), 3.06 – 2.99 (m, 4H), 1.09 – 1.03 (m, 21H); ^{13}C NMR (100 MHz, $CDCl_3$) δ 155.6 (d, J_{CF1} = 246.6 Hz), 154.3, 153.6, 136.7 (d, J_{CF3} = 9.0 Hz), 132.9 (d, J_{CF2} = 10.4 Hz), 118.9 (d, J_{CF4} = 4.2 Hz), 114.2 (d, J_{CF4} = 3.3 Hz), 107.8 (d, J_{CF2} = 26.3 Hz), 98.8, 90.7, 71.7, 67.1, 51.1 (d, J_{CF4} = 3.1 Hz), 47.9, 42.4, 18.6, 11.1; ^{19}F NMR (375 MHz, $CDCl_3$) δ -120.1; HRMS (ESI): Calcd for $C_{26}H_{38}N_3O_4FSi$ $[M+H]^+$: 504.2694; found: 504.2698.

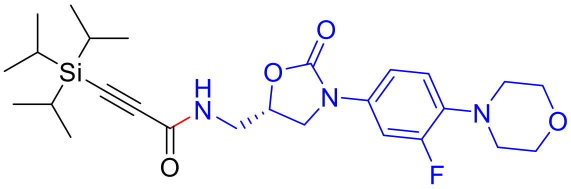

Tert-butyl ((13,13-diisopropyl-14-methyl-10-oxo-3,6-dioxa-9-aza-13-silapentadec-11-yn-1-yl)oxy)carbamate (3ak): Light yellow liquid, yield 48% (45 mg); ^{1}H NMR (400 MHz, $CDCl_3$, 25:3 mixture of rotamers) δ 7.63 (s, 1H), 6.59 (s, 1H), 4.06 – 4.04 (m, 2H, rotamer), 4.04 – 4.00 (m, 2H), 3.74 – 3.71 (m, 2H), 3.69 – 3.65 (m, 2H), 3.65 – 3.63 (m, 2H), 3.61 – 3.58 (m, 2H), 3.49 (dd, J = 10.4, 5.2 Hz, 2H), 1.47 (s, 9H), 1.09 – 1.07 (m, 21H); ^{13}C NMR (100 MHz, $CDCl_3$) δ 156.9, 153.0, 99.9, 88.4, 81.9, 75.5, 70.5, 70.3, 69.7, 69.4, 39.7, 28.4, 18.6, 11.2; HRMS (ESI): Calcd for $C_{23}H_{44}N_2O_6Si$ $[M+Na]^+$: 495.2866; found: 495.2859.

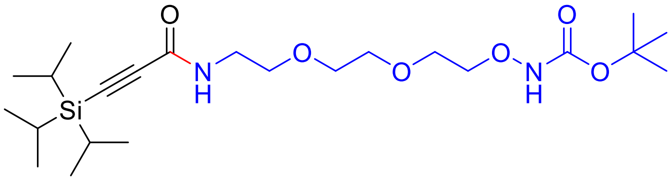

N-(2-(1H-imidazol-4-yl)ethyl)-3-(triisopropylsilyl)propiolamide (3al): Viscus liquid, yield 58% (37 mg); Recovered starting material 27% (13.5 mg); ^{1}H NMR (400 MHz, $CDCl_3$) δ 7.55 (s, 1H), 7.14 (t, *J* = 5.6 Hz, 1H), 6.82 (s, 1H), 3.74 (q, *J* = 6.4 Hz, 2H, rotamers), 3.55 (q, *J* = 6.4 Hz, 2H), 2.88 – 2.78 (m, 2H), 1.08 – 1.04 (m, 21H); ^{13}C NMR (100 MHz, $CDCl_3$) δ 153.3, 135.9, 135.0, 115.9, 99.9, 88.7, 39.9, 26.7, 18.6, 11.1; HRMS (ESI): Calcd for $C_{17}H_{29}N_3OSi$ $[M+Na]^+$: 342.1978; found: 342.1990.

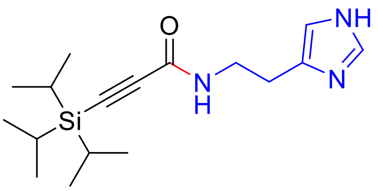

Methyl N2-(tert-butoxycarbonyl)-N6-(3-(triisopropylsilyl)propioloyl)-L-lysyl-L-valinate (3am): Viscus liquid, yield 81% (90 mg); ^{1}H NMR (400 MHz, $CDCl_3$, 50:9 mixture of rotamers) δ 6.68 (s, 1H), 6.06 (s, 1H), 5.13 (s, 1H), 4.49 (dd, *J* = 8.4, 4.8 Hz, 1H), 4.16 – 4.02 (m, 1H), 3.71 (s, 3H), 3.41 (q, *J* = 6.8 Hz, 2H, rotamer), 3.26 (q, *J* = 6.8 Hz, 2H), 2.20 – 2.12 (m, 1H), 1.88 – 1.78 (m, 1H), 1.67 – 1.50 (m, 4H), 1.42 (s, 9H), 1.40 – 1.38 (m, 1H), 1.08 – 1.06 (s, 21H), 0.90 (dd, *J* = 10.0, 6.8 Hz, 6H); ^{13}C NMR (100 MHz, $CDCl_3$) δ 172.3, 172.2, 155.9, 152.9, 100.0, 88.1, 80.2, 57.3, 54.4, 52.3, 39.5, 31.2, 28.9, 28.4, 22.9, 19.0, 18.6, 17.8, 11.1; HRMS (ESI): Calcd for $C_{29}H_{53}N_3O_6Si$ $[M+H]^+$: 568.3782; found: 568.3794.

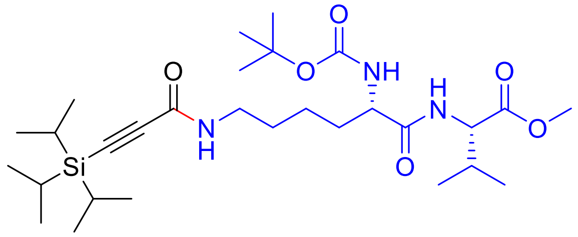

Methyl N2-(tert-butoxycarbonyl)-N6-(3-(triisopropylsilyl)propioloyl)-L-lysyl-D-valinate (3am'): Viscus liquid, yield 78% (87 mg); ^{1}H NMR (400 MHz, $CDCl_3$, 25:4 mixture of rotamers) δ 6.76 (s, 1H), 6.01 (t, J = 5.6 Hz, 1H), 5.11 (d, J = 6.8 Hz, 1H), 4.49 (dd, J = 8.8, 5.0 Hz, 1H), 4.11 (s, 1H), 3.71 (s, 3H), 3.42 (q, J = 6.8 Hz, 2H, rotamer), 3.32 – 3.21 (m, 2H), 2.22 – 2.10 (m, 1H), 1.96 – 1.80 (m, 2H), 1.66 – 1.52 (m, 3H), 1.43 (s, 9H), 1.41 – 1.36 (m, 1H), 1.10 – 1.06 (m, 21H), 0.90 (dd, J = 17.6, 6.8 Hz, 6H); ^{13}C NMR (100 MHz, $CDCl_3$) δ 172.4, 172.1, 155.9, 153.0, 99.9, 88.2, 80.4, 57.2, 54.5, 52.3, 39.5, 31.2, 29.1, 28.4, 22.9, 19.1, 18.6, 17.8, 11.1; HRMS (ESI): Calcd for $C_{29}H_{53}N_3O_6Si$ $[M+H]^+$: 568.3782; found: 568.3790.

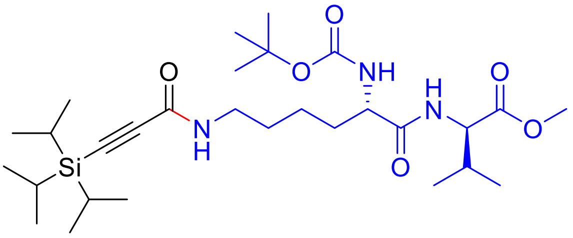

Methyl N2-(tert-butoxycarbonyl)-N6-propioloyl-L-lysyl-L-valinate (5am): Viscus liquid, yield 90% (39 mg); ^{1}H NMR (400 MHz, $CDCl_3$) δ 6.67 (s, 1H), 6.46 (s, 1H), 5.20 (d, *J* = 4.8 Hz, 1H), 4.52 (dd, *J* = 8.6, 4.8 Hz, 1H), 4.13 (d, *J* = 6.0 Hz, 1H), 3.75 (s, 3H), 3.44 (q, *J* = 6.4 Hz, 2H, rotamer), 3.32 (q, *J* = 6.4

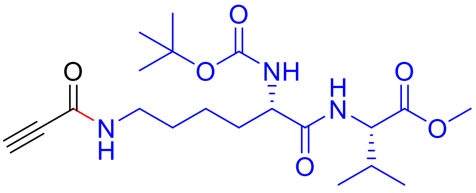

Hz, 2H), 2.79 (s, 1H), 2.24 – 2.14 (m, 1H), 1.90 – 1.80 (m, 1H), 1.72 – 1.54 (m, 4H), 1.45 (s, 9H), 1.42 – 1.39 (m, 1H), 0.93 (dd, *J* = 10.8, 6.8 Hz, 6H); ^{13}C NMR (100 MHz, $CDCl_3$) δ 172.4, 172.2, 155.9, 152.4, 80.3, 77.6, 73.1, 57.3, 54.3, 52.4, 39.4, 31.7, 31.1, 28.4, 22.7, 19.1, 17.8; HRMS (ESI): Calcd for $C_{20}H_{33}N_3O_6$ $[M+Na]^+$: 434.2267; found: 434.2261.

Methyl *N*2-(tert-butoxycarbonyl)-*N*6-propioloyl-*L*-lysyl-*D*-valinate (5am'): Viscus liquid,

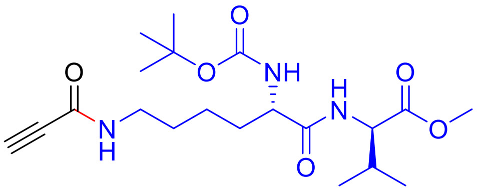

yield 85% (37 mg); ^{1}H NMR (400 MHz, $CDCl_3$, 10:1 mixture of rotamers) δ 6.79 (s, 1H), 6.37 (s, 1H), 5.15 (d, J = 7.2 Hz, 1H), 4.49 (dd, J = 8.8, 5.0 Hz, 1H), 4.16 – 4.06 (m, 1H), 3.72 (s, 3H), 3.42 (q, J = 6.8 Hz, 2H, rotamer), 3.29 (q, J = 6.8 Hz, 2H), 2.78 (s, 1H), 2.22 – 2.11 (m, 1H), 1.92 – 1.80 (m, 2H), 1.68 – 1.52 (m, 3H), 1.44 (s, 9H), 1.40 – 1.38 (m, 1H), 0.91 (dd, J = 17.2, 6.8 Hz, 6H); ^{13}C NMR (100 MHz, $CDCl_3$) δ 172.4, 172.2, 155.9, 152.4, 80.4, 77.6, 73.2, 57.2, 54.4, 52.3, 39.5, 31.9, 31.2, 28.4, 22.8, 19.1, 17.8; HRMS (ESI): Calcd for $C_{20}H_{33}N_3O_6$ $[M+Na]^+$: 434.2267; found: 434.2270.

***N*-benzylpropiolamide (5a)**: White solid, yield 90%; ^{1}H NMR (400 MHz, $CDCl_3$, 25:2

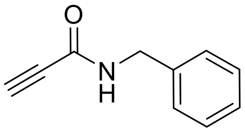

mixture of rotamers) δ 7.43 – 7.21 (m, 5H), 6.26 (s, 1H), 4.63 (d, J = 6.8 Hz, 2H, rotamer), 4.48 (d, J = 6.0 Hz, 2H), 3.14 (s, 1H, rotamer), 2.80 (s, 1H); ^{13}C NMR (100 MHz, $CDCl_3$) δ 152.1, 137.1, 128.9, 128.1, 128.0, 77.3, 73.7, 44.0. HRMS (ESI): Calcd for $C_{10}H_9NO$ $[M+Na]^+$: 182.0582; found: 182.0581.

NMR Spectra

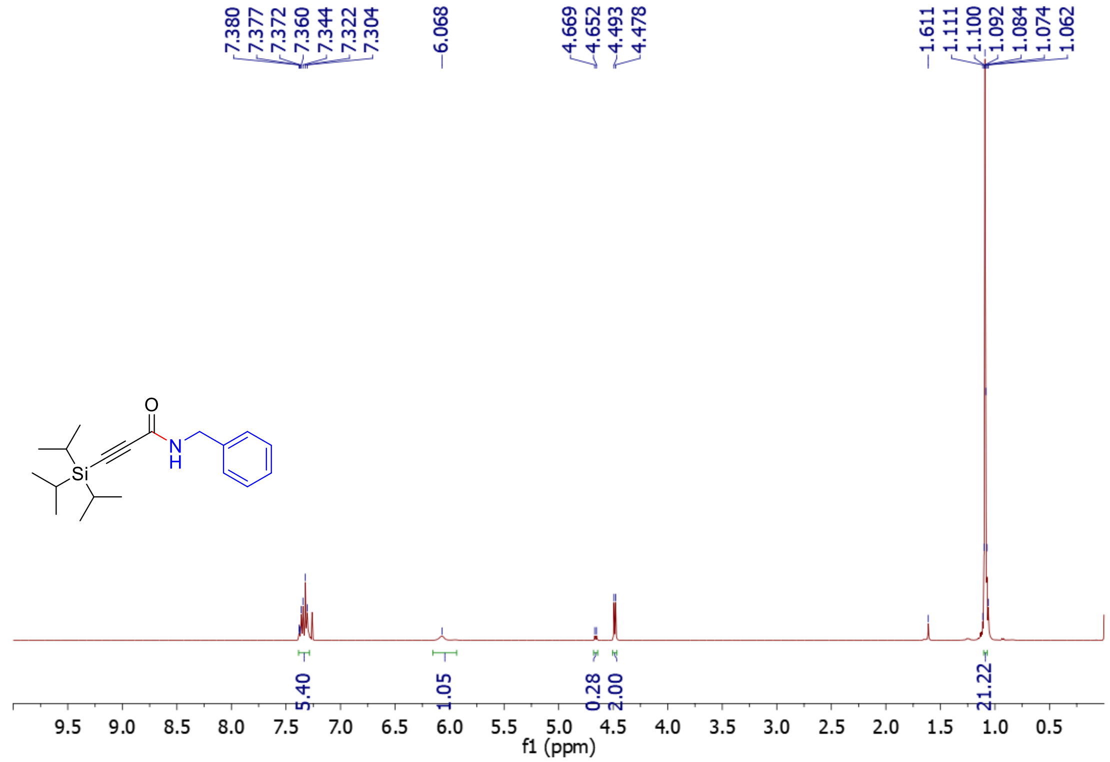

Fig. S39. ^{1}H NMR spectrum of N-benzyl-3-(triisopropylsilyl)propiolamide **(3a)** in $CDCl_3$.

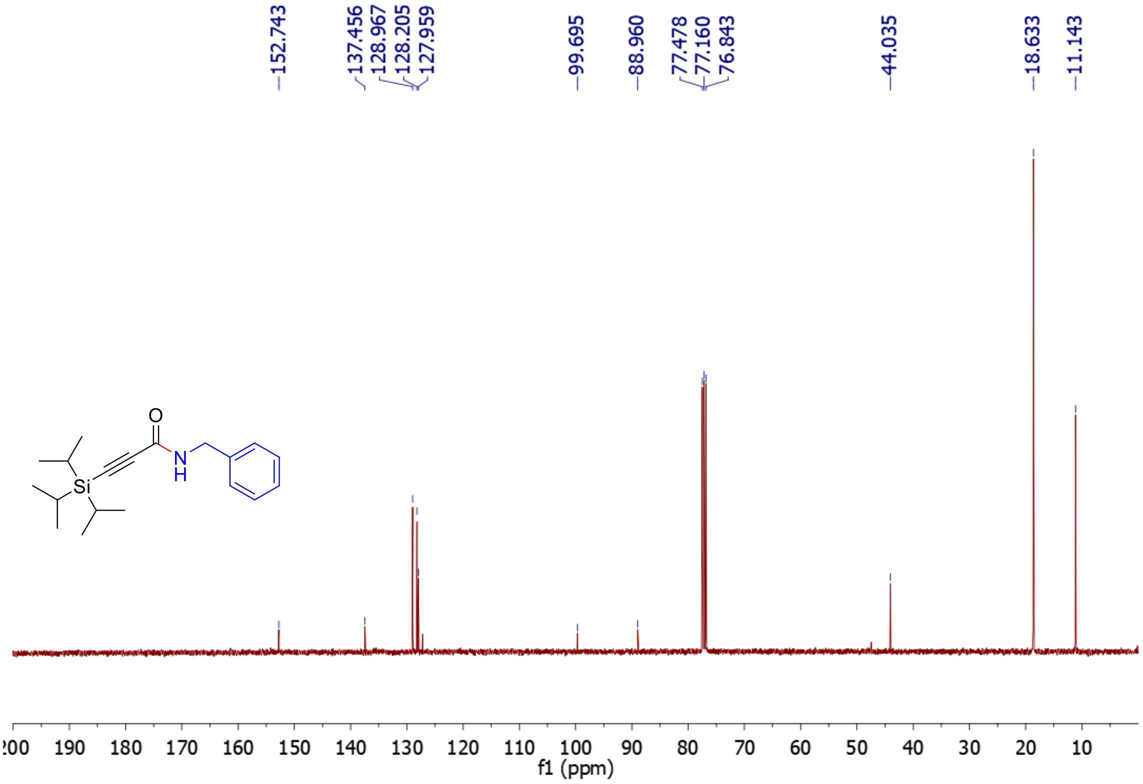

Fig. S40. ^{13}C NMR spectrum of N-benzyl-3-(triisopropylsilyl)propiolamide **(3a)** in $CDCl_3$.

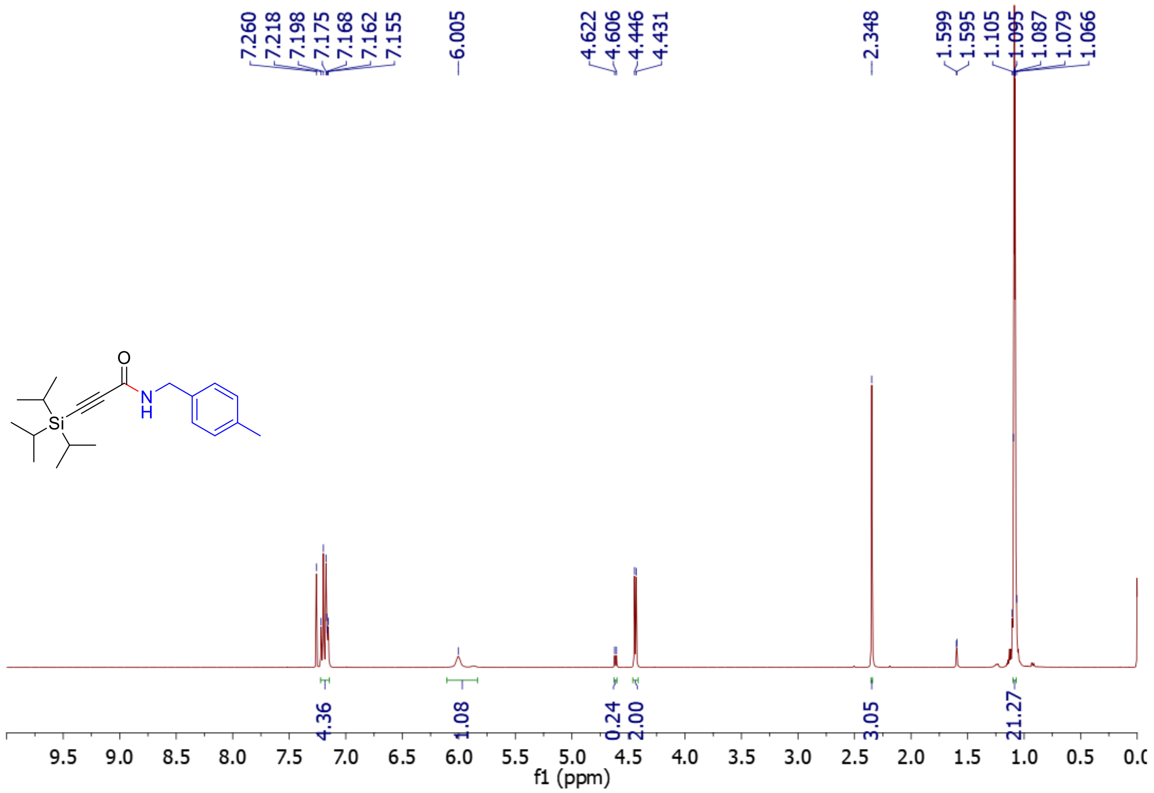

Fig. S41. ^{1}H NMR spectrum of N-(4-methylbenzyl)-3-(triisopropylsilyl)propiolamide **(3b)** in $CDCl_3$.

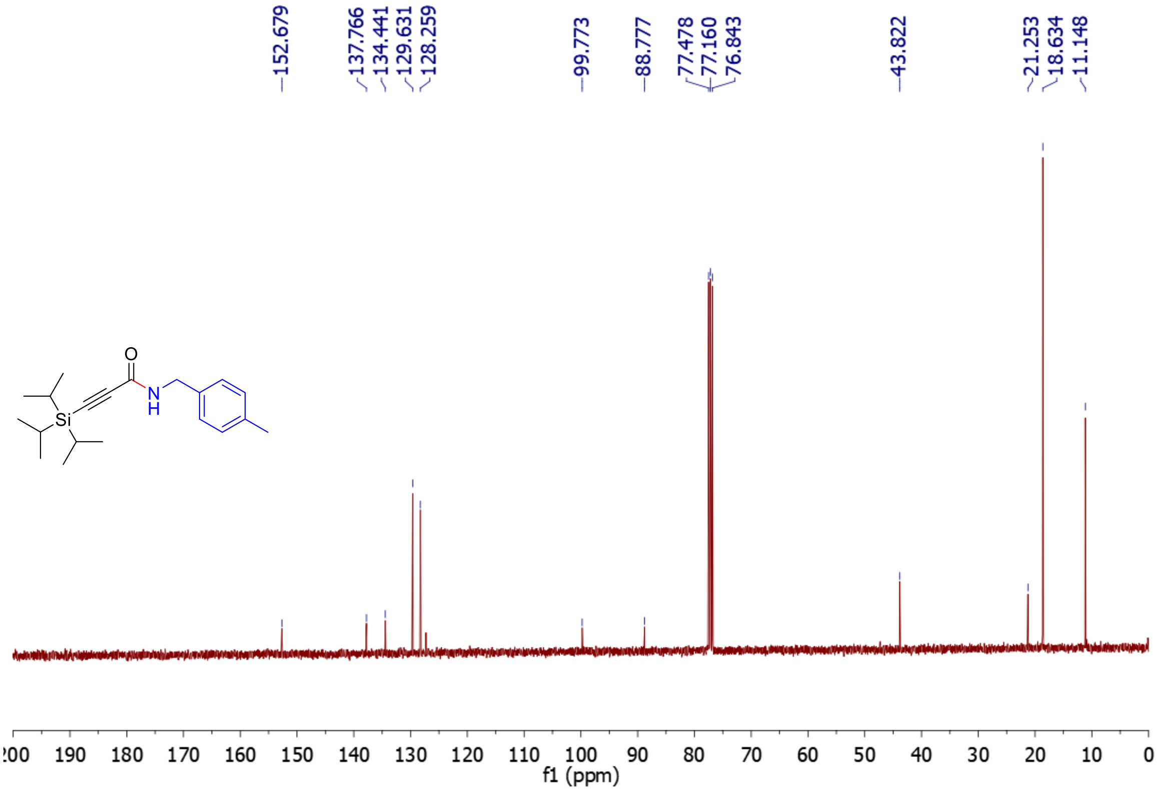

Fig. S42. ^{13}C NMR spectrum of N-(4-methylbenzyl)-3-(triisopropylsilyl)propiolamide **(3b)** in $CDCl_3$.

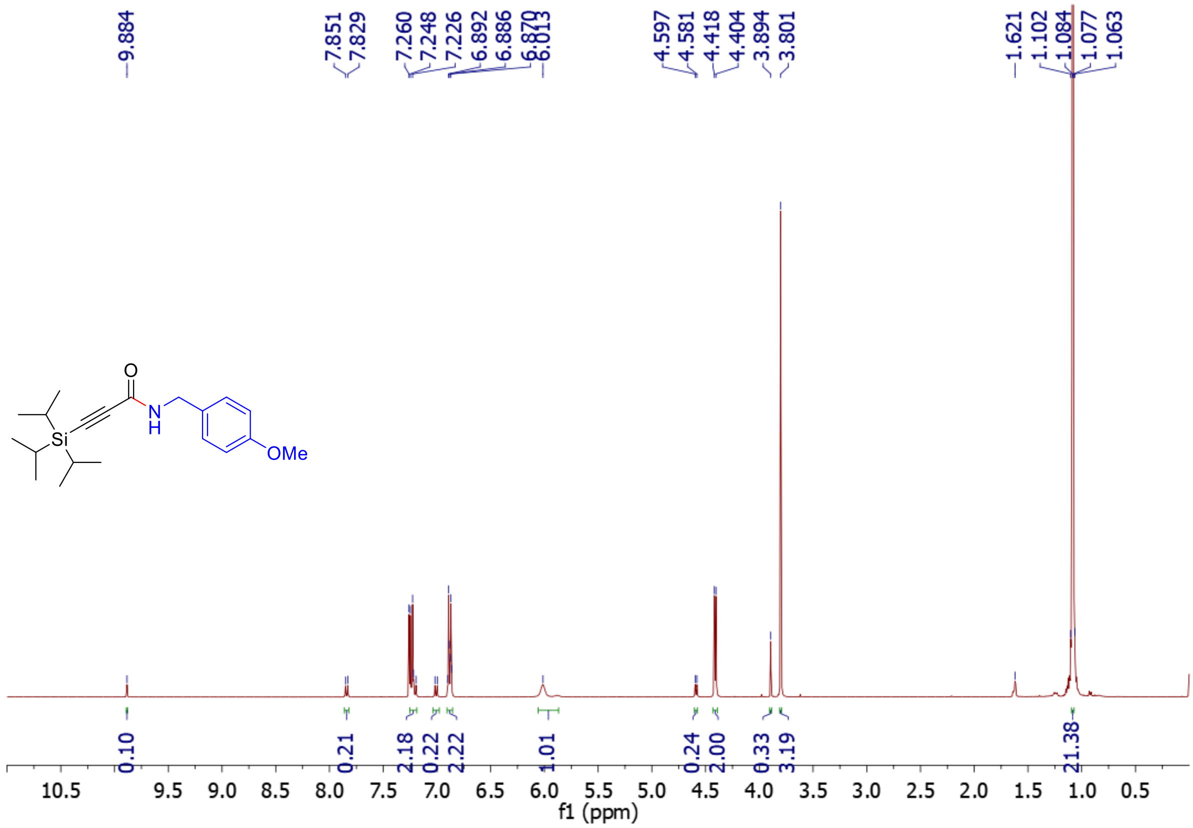

Fig. S43. ^{1}H NMR spectrum of N-(4-methoxybenzyl)-3-(triisopropylsilyl)propiolamide **(3c)** in $CDCl_3$.

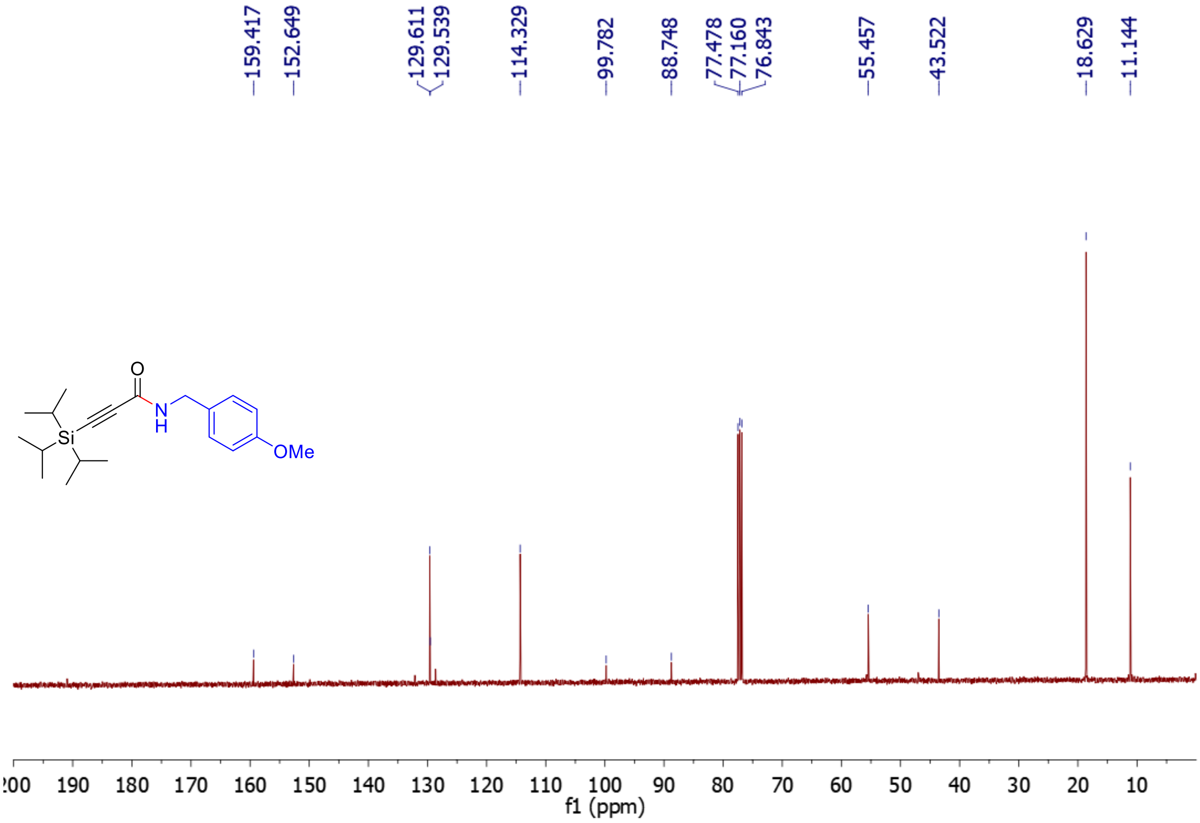

Fig. S44. ^{13}C NMR spectrum of N-(4-methoxybenzyl)-3-(triisopropylsilyl)propiolamide **(3c)** in $CDCl_3$.

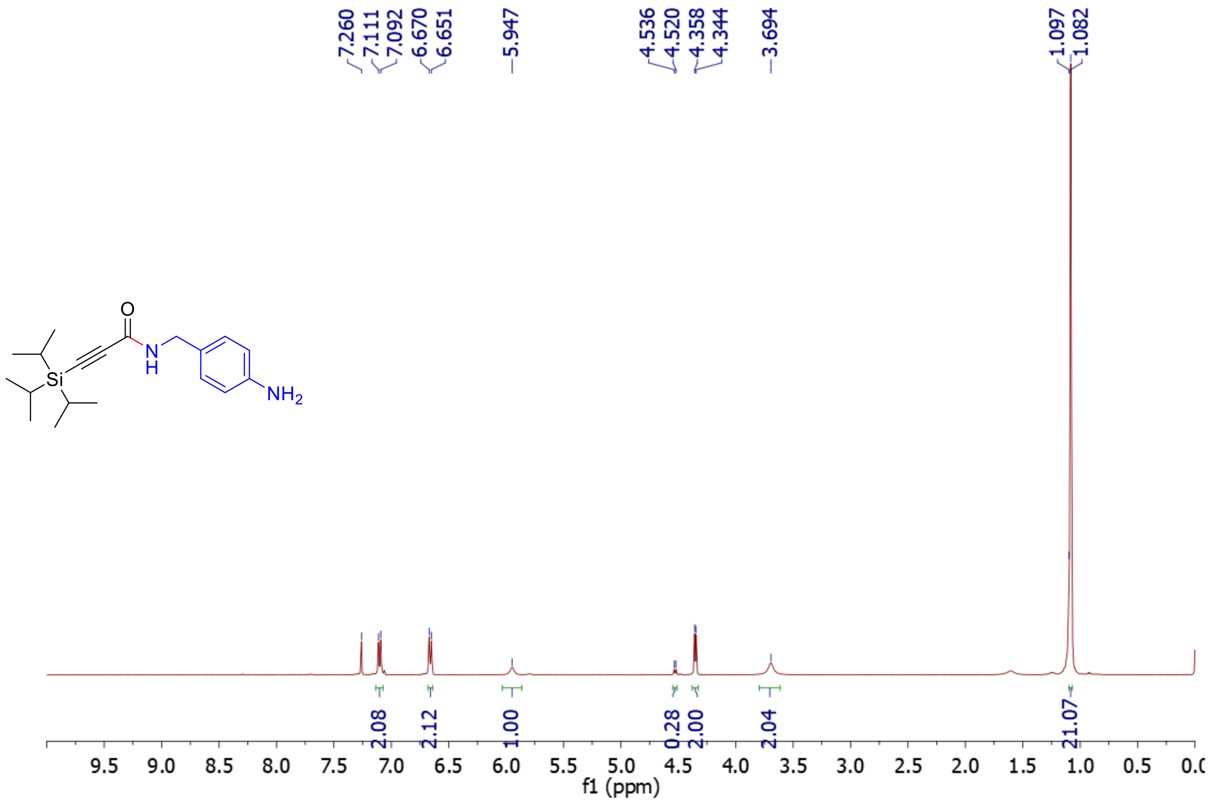

Fig. S45. ^{1}H NMR spectrum of N-(4-aminobenzyl)-3-(triisopropylsilyl)propiolamide **(3d)** in $CDCl_3$.

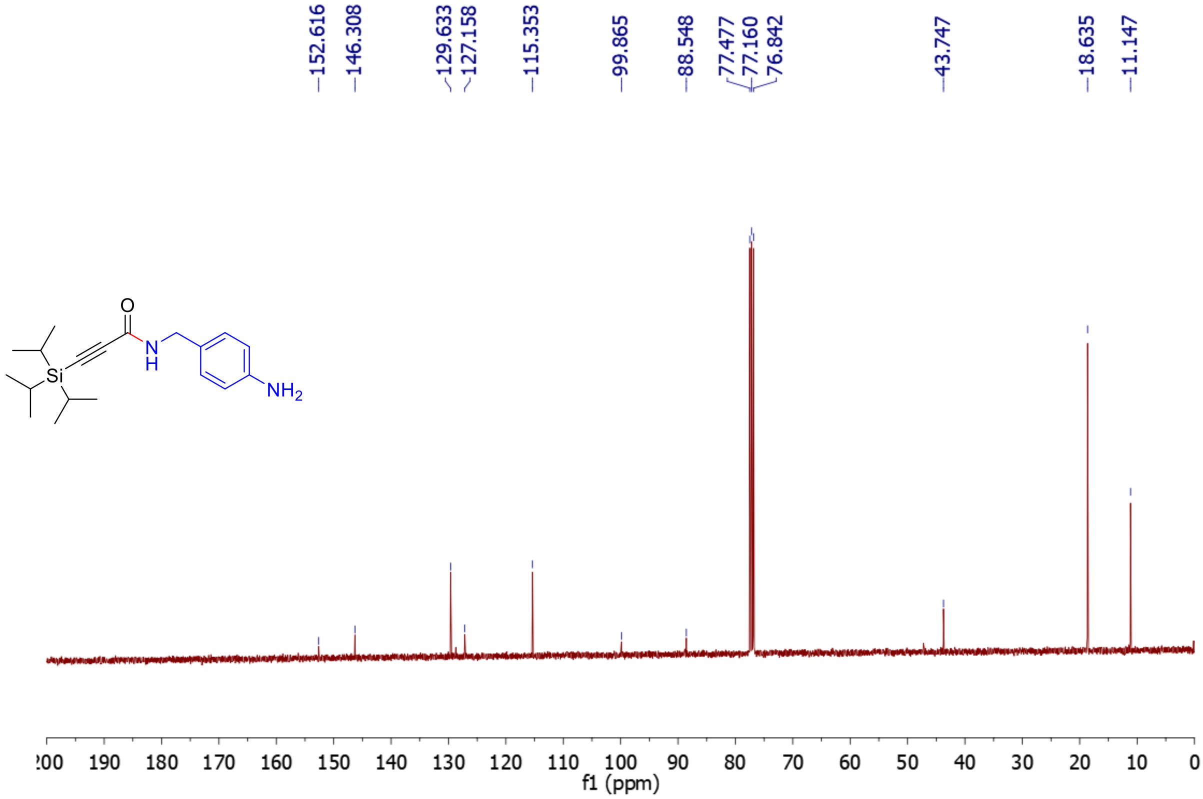

Fig. S46. ^{13}C NMR spectrum of N-(4-aminobenzyl)-3-(triisopropylsilyl)propiolamide **(3d)** in $CDCl_3$.

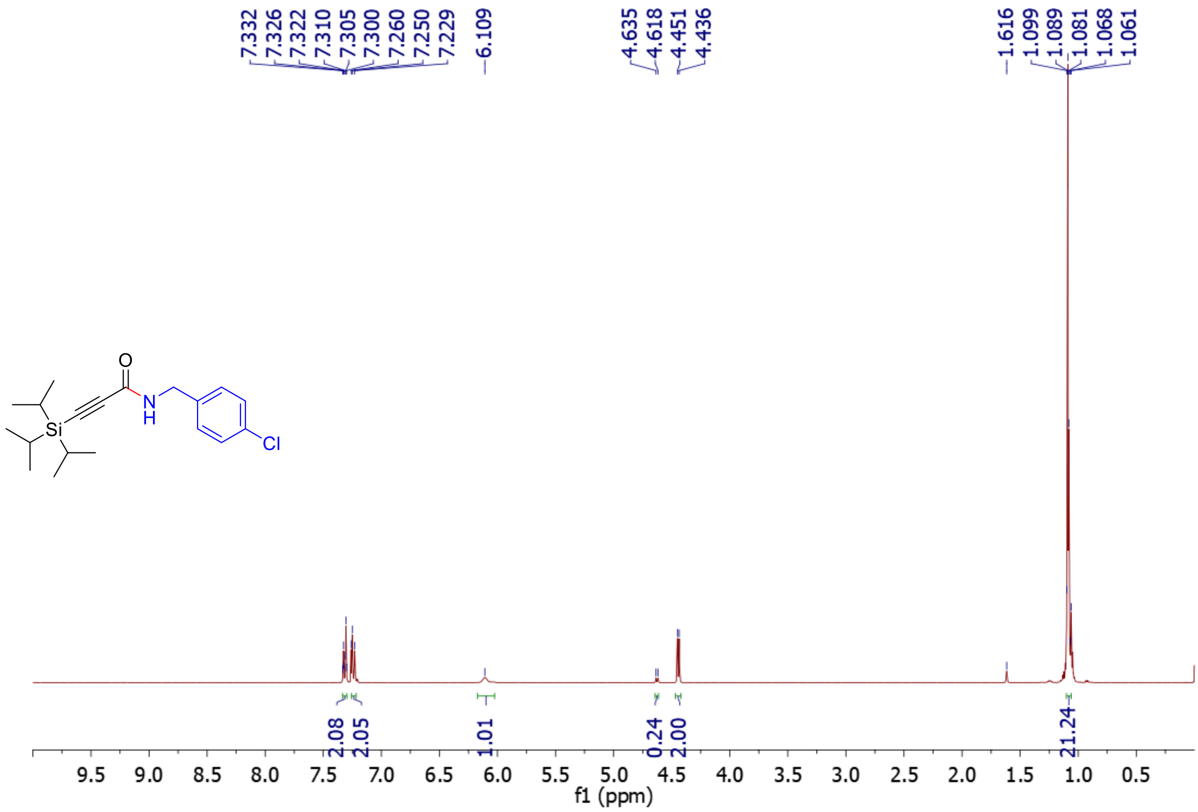

Fig. S47. ^{1}H NMR spectrum of N-(4-chlorobenzyl)-3-(triisopropylsilyl)propiolamide **(3e)** in $CDCl_3$.

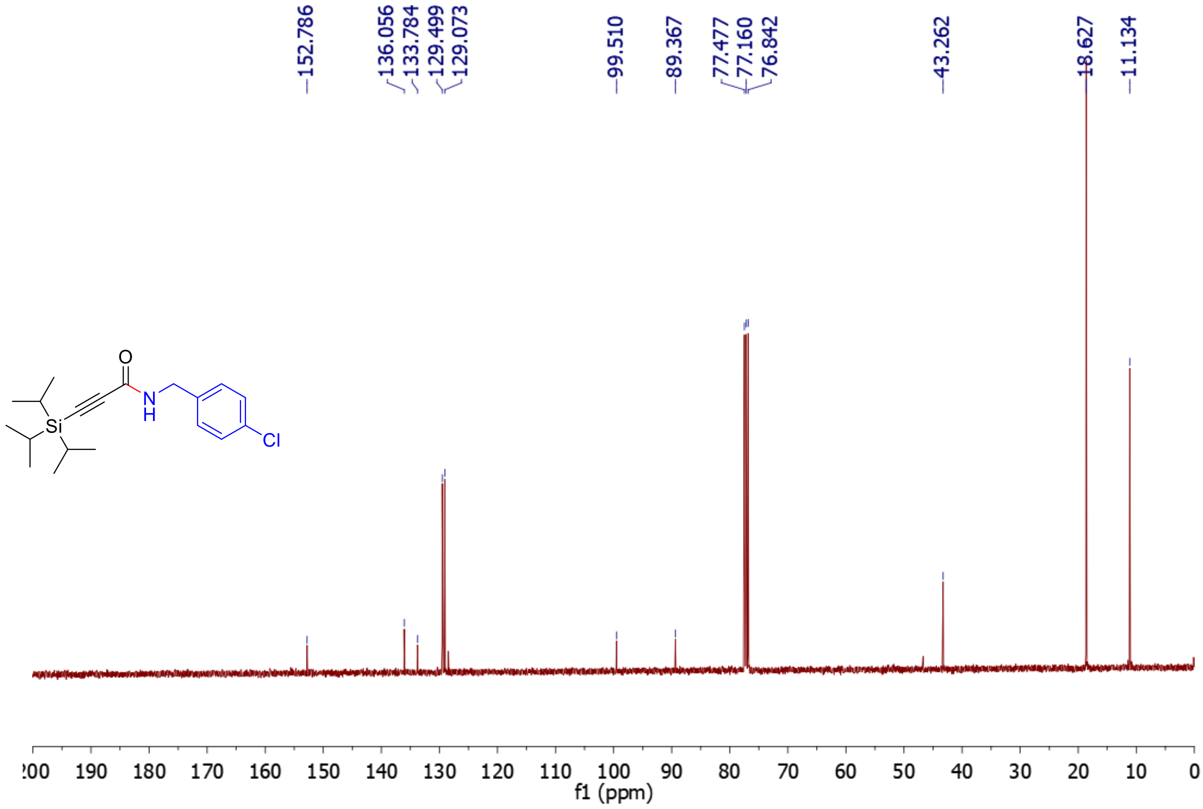

Fig. S48. ^{13}C NMR spectrum of N-(4-chlorobenzyl)-3-(triisopropylsilyl)propiolamide **(3e)** in $CDCl_3$.

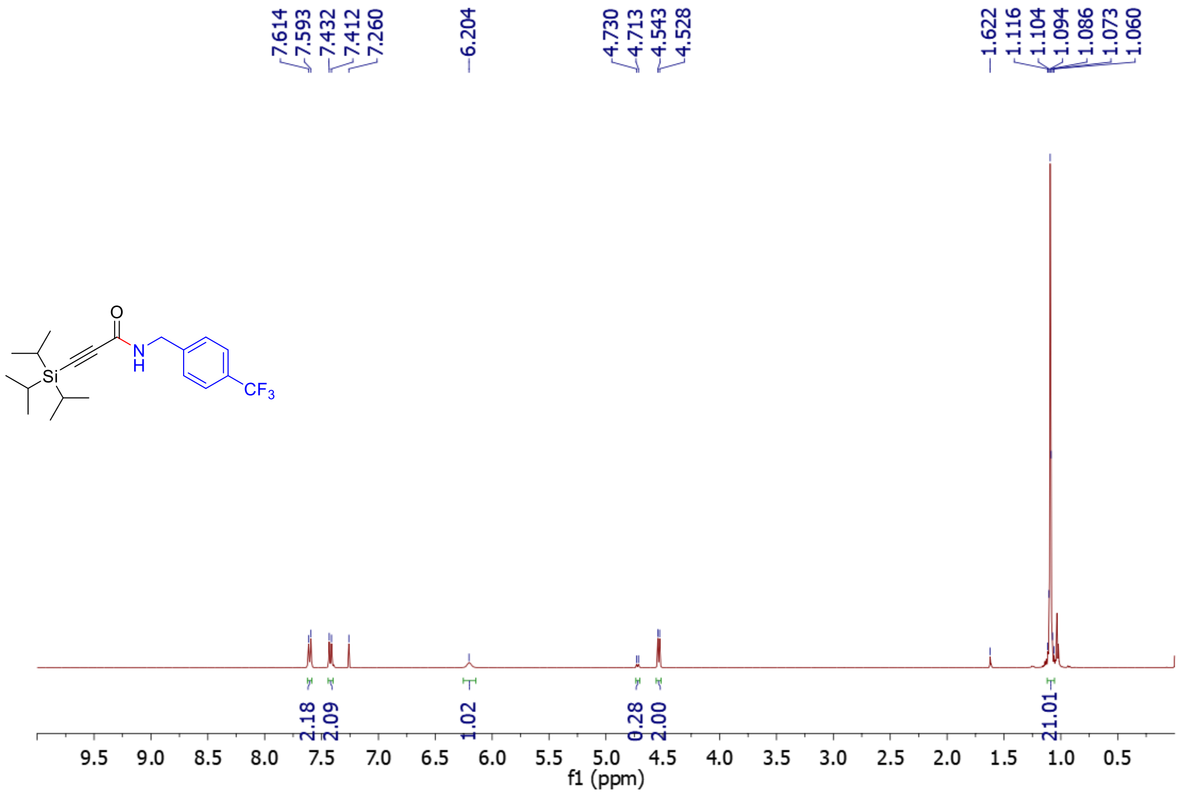

Fig. S49. ^{1}H NMR spectrum of N-(4-(trifluoromethyl)benzyl)-3-(triisopropylsilyl)propiolamide **(3f)** in $CDCl_3$.

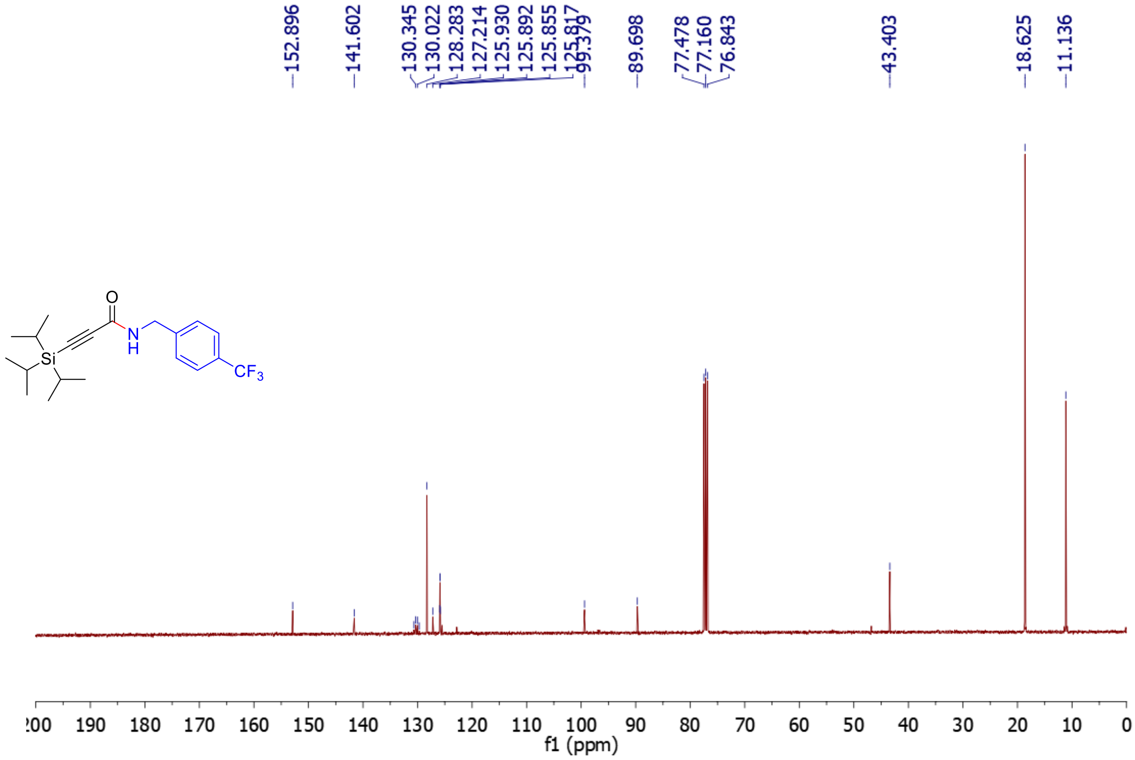

Fig. S50. ^{13}C NMR spectrum of N-(4-(trifluoromethyl)benzyl)-3-(triisopropylsilyl)propiolamide **(3f)** in $CDCl_3$.

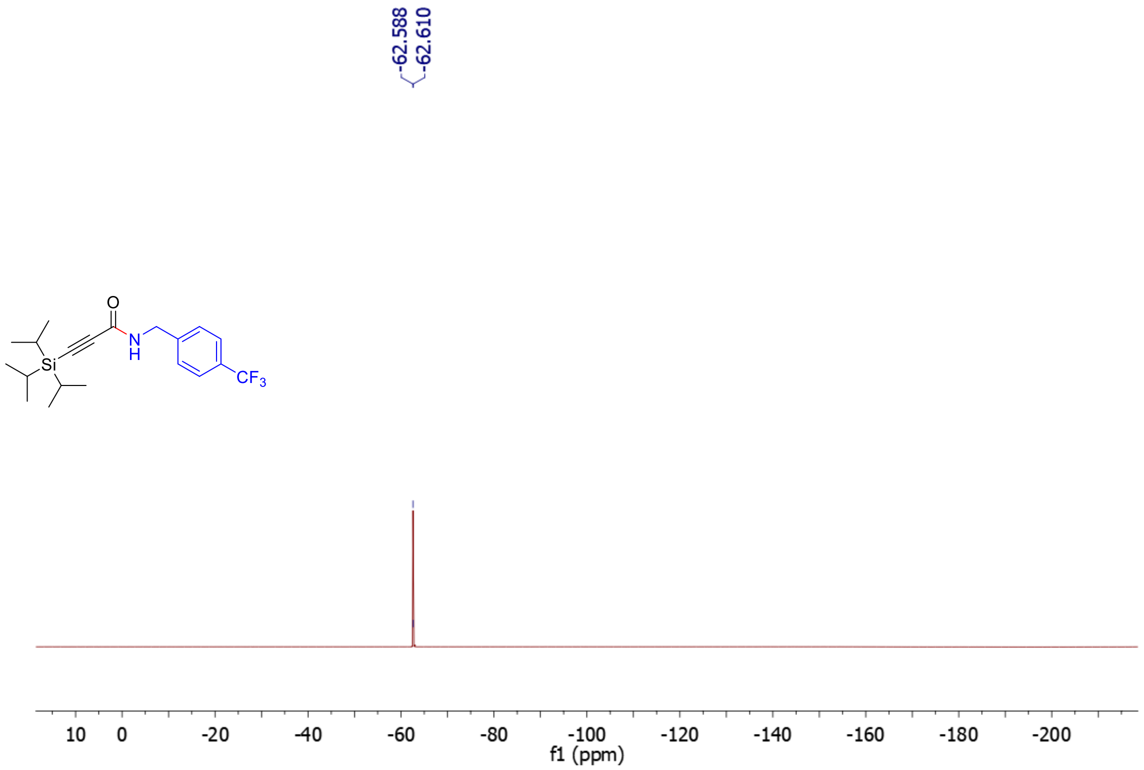

Fig. S51. ^{19}F NMR spectrum of N-(4-(trifluoromethyl)benzyl)-3-(triisopropylsilyl)propiolamide **(3f)** in $CDCl_3$.

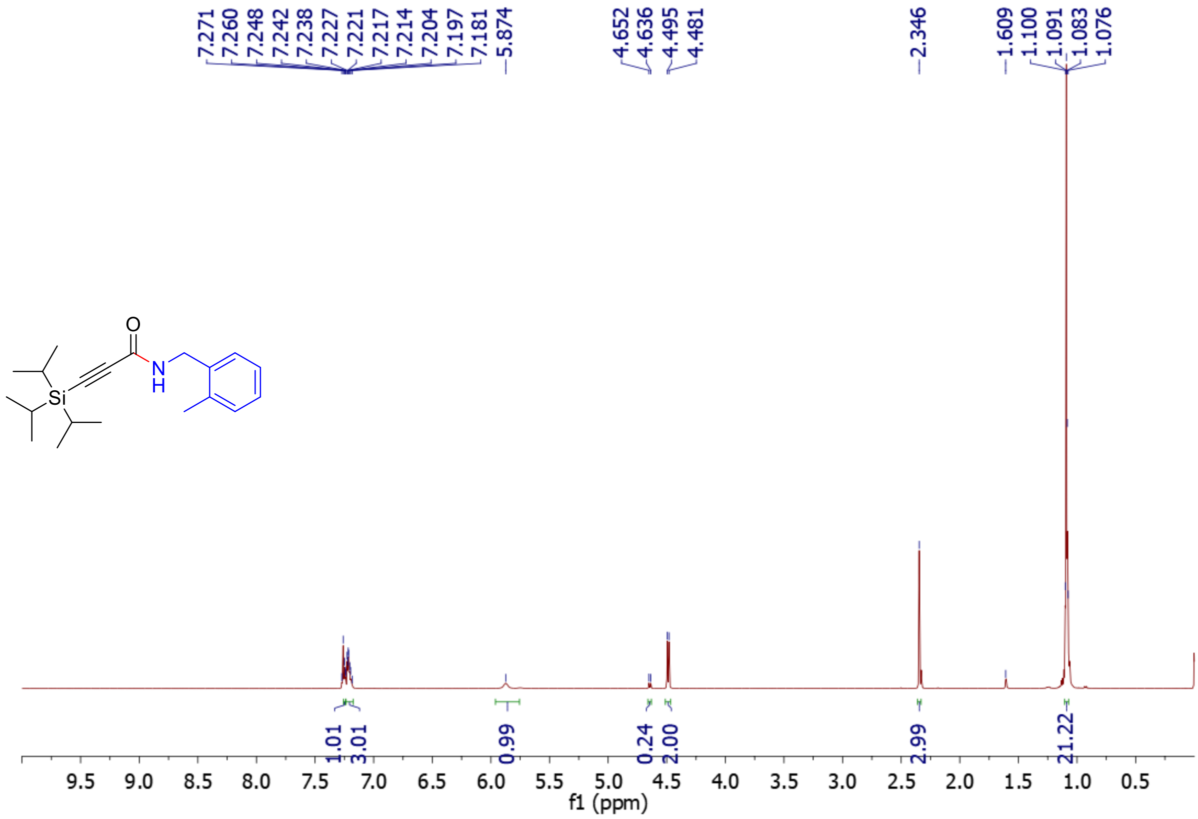

Fig. S52. ^{1}H NMR spectrum of N-(2-methylbenzyl)-3-(triisopropylsilyl)propiolamide **(3g)** in $CDCl_3$.

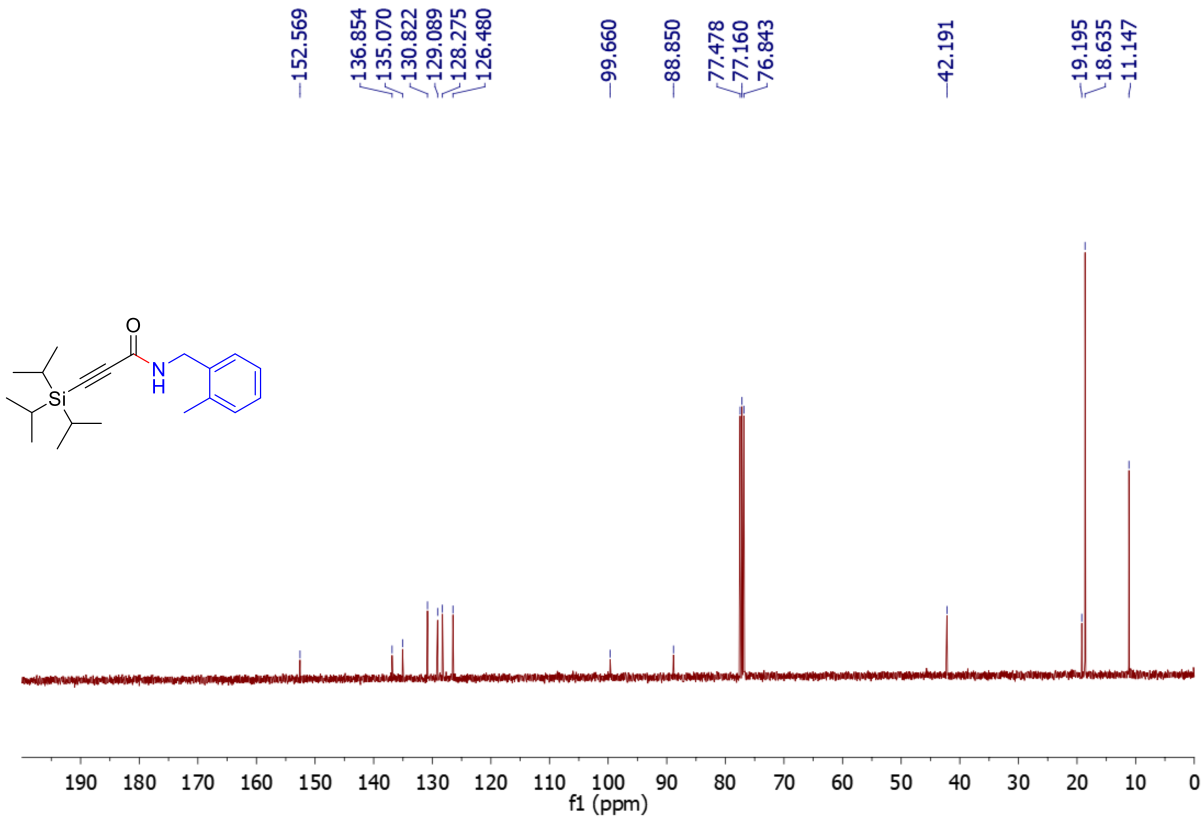

Fig. S53. ^{13}C NMR spectrum of N-(2-methylbenzyl)-3-(triisopropylsilyl)propiolamide **(3g)** in $CDCl_3$.

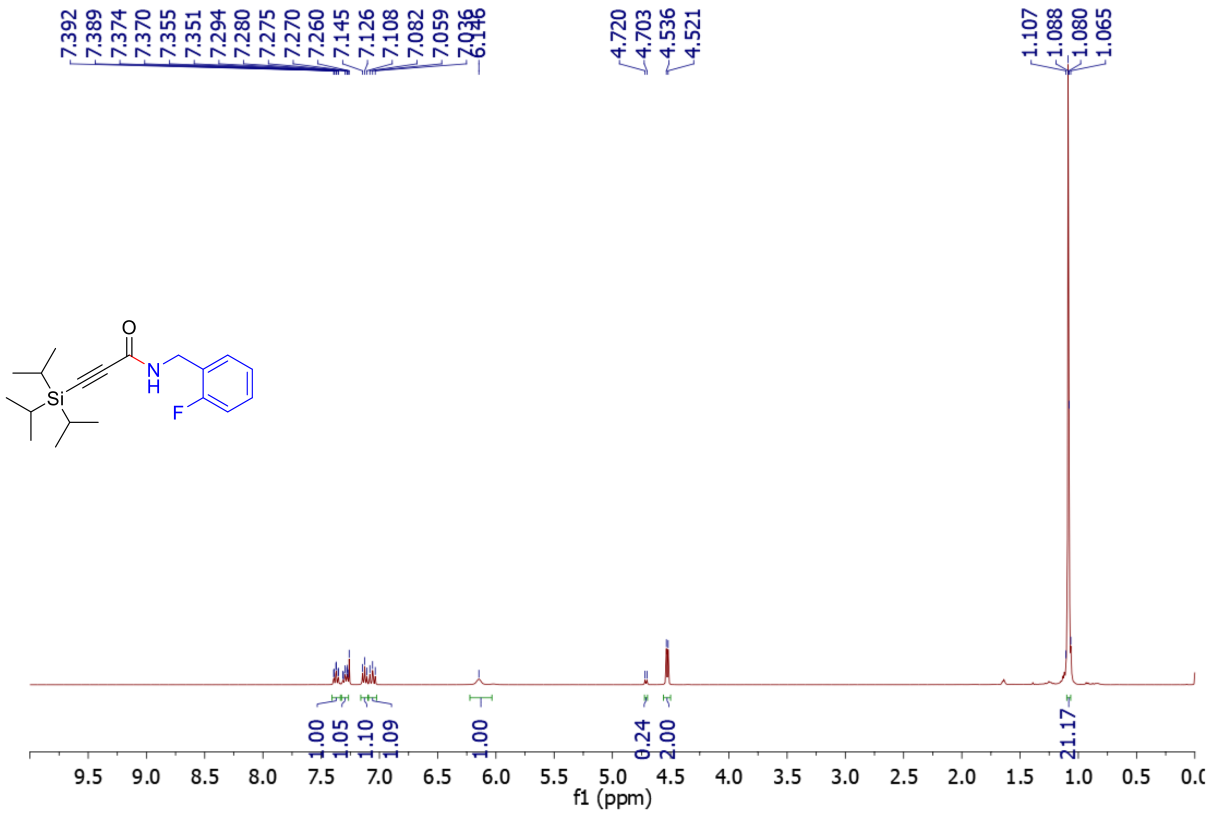

Fig. S54. ^{1}H NMR spectrum of N-(2-fluorobenzyl)-3-(triisopropylsilyl)propiolamide **(3h)** in $CDCl_3$.

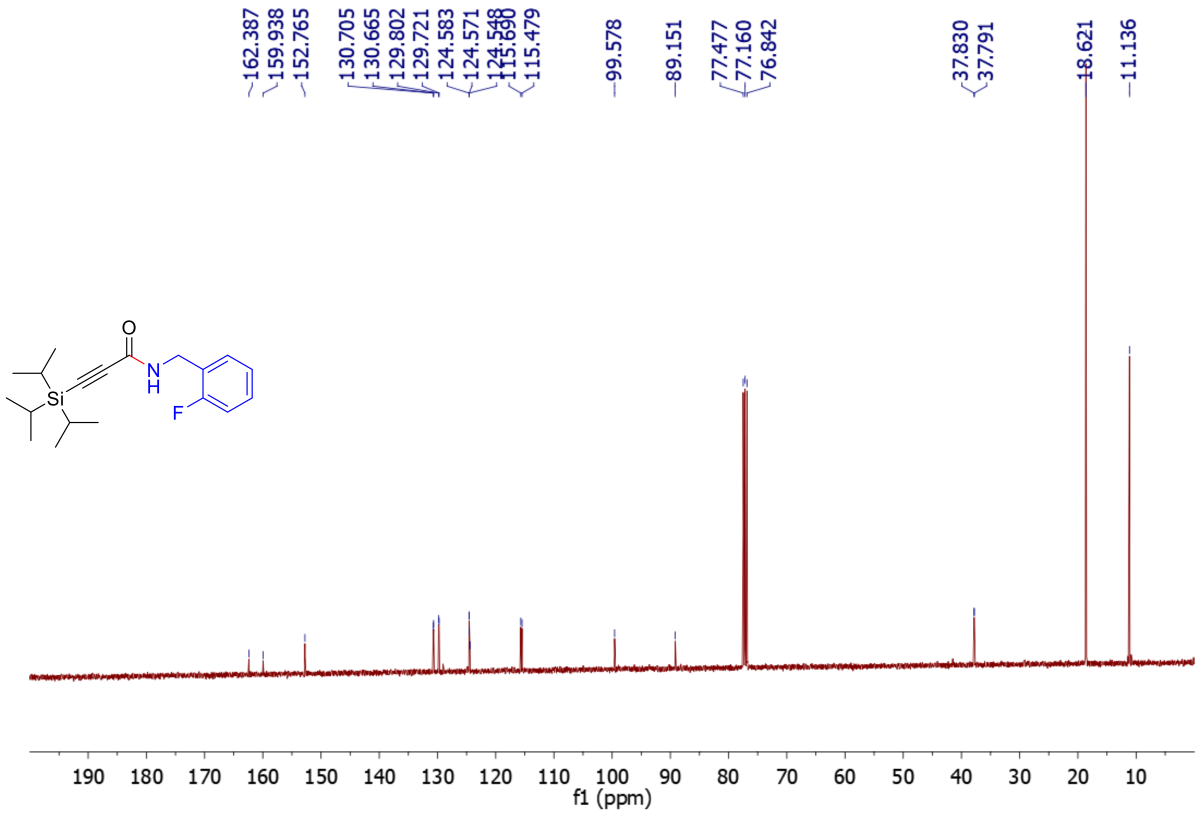

Fig. S55. ^{13}C NMR spectrum of N-(2-fluorobenzyl)-3-(triisopropylsilyl)propiolamide **(3h)** in $CDCl_3$.

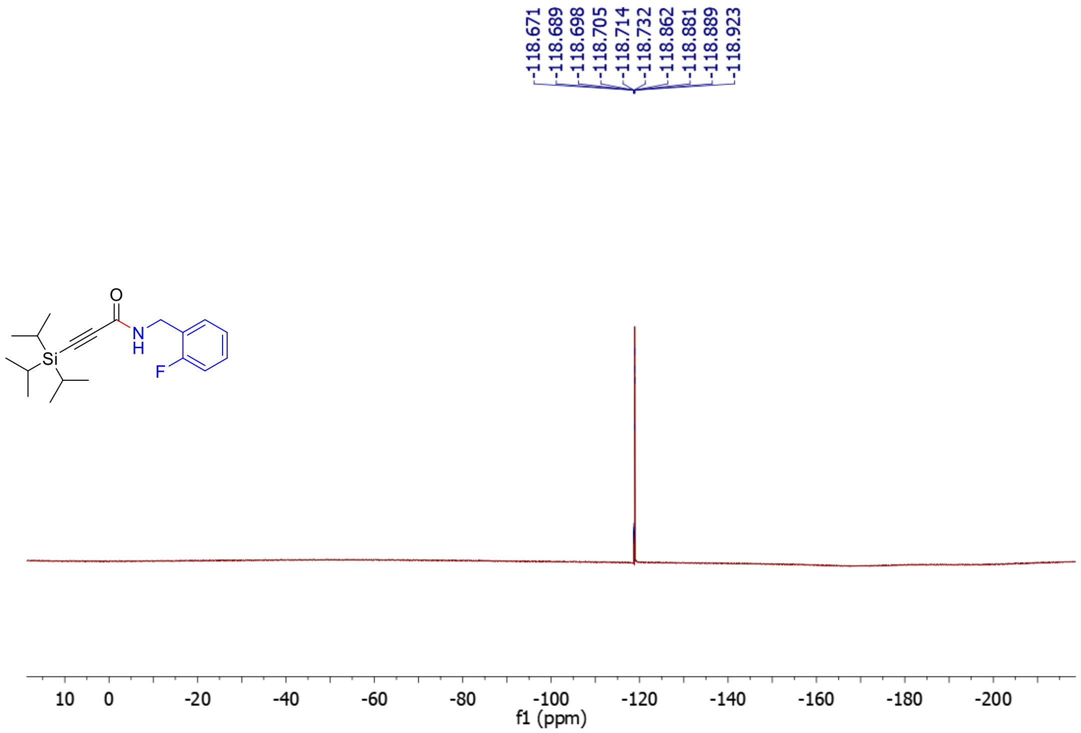

Fig. S56. ^{19}F NMR spectrum of N-(2-fluorobenzyl)-3-(triisopropylsilyl)propiolamide **(3h)** in $CDCl_3$.

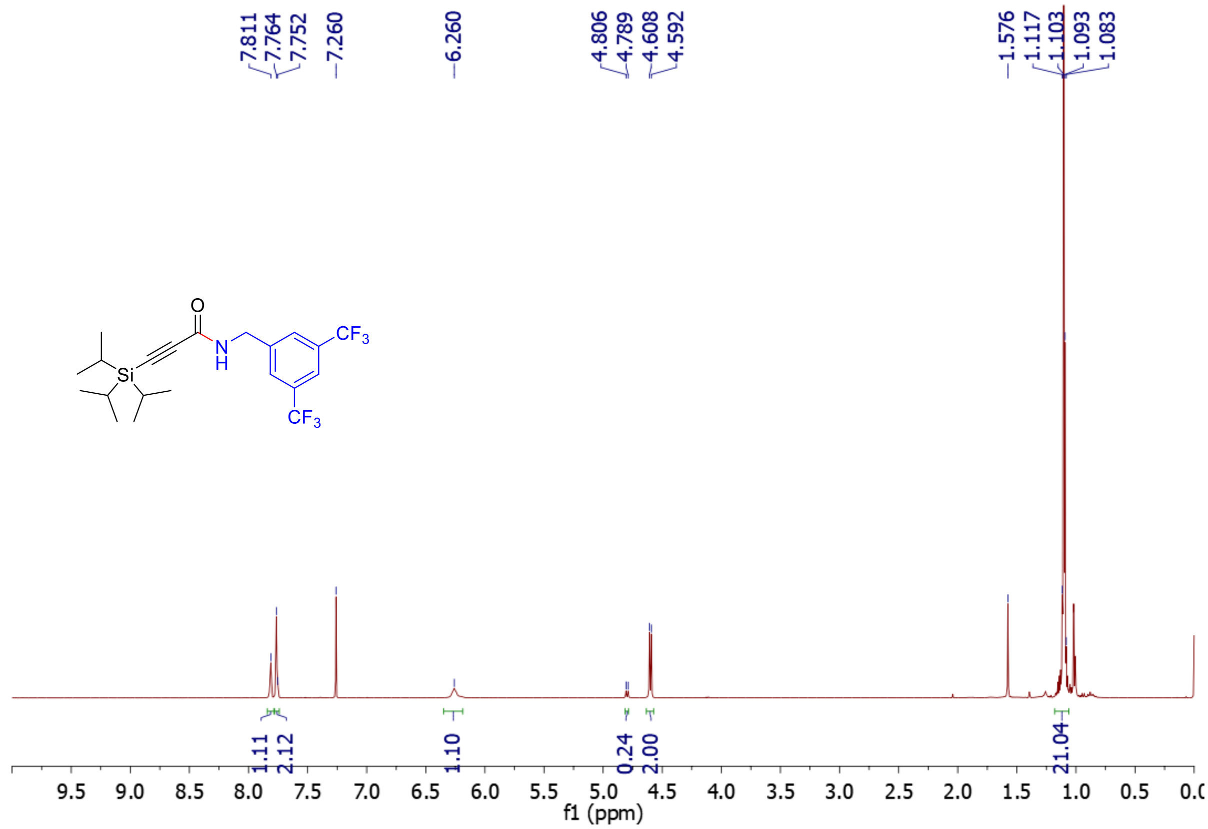

Fig. S57. ¹H NMR spectrum of N-(3,5-bis(trifluoromethyl)benzyl)-3-(triisopropylsilyl)propiolamide **(3i)** in $CDCl_3$.

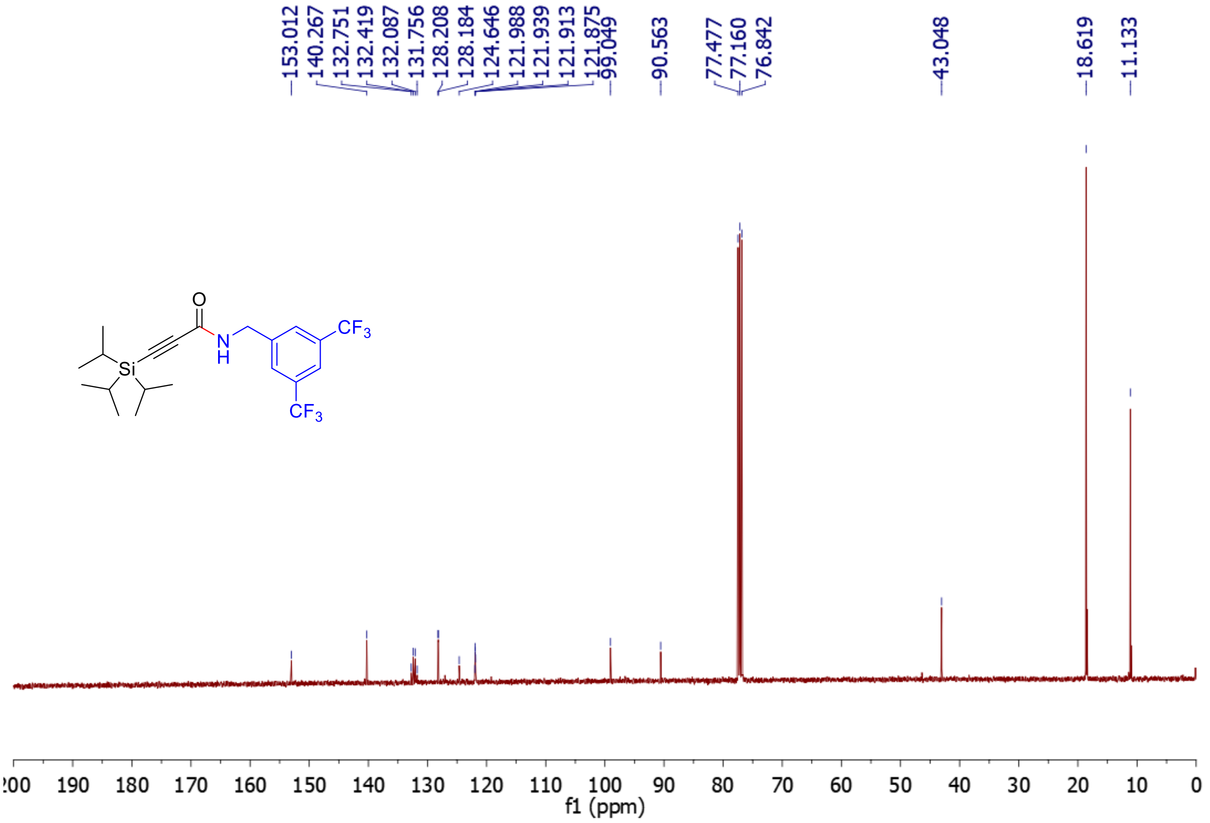

Fig. S58. ¹³C NMR spectrum of N-(3,5-bis(trifluoromethyl)benzyl)-3-(triisopropylsilyl)propiolamide **(3i)** in $CDCl_3$.

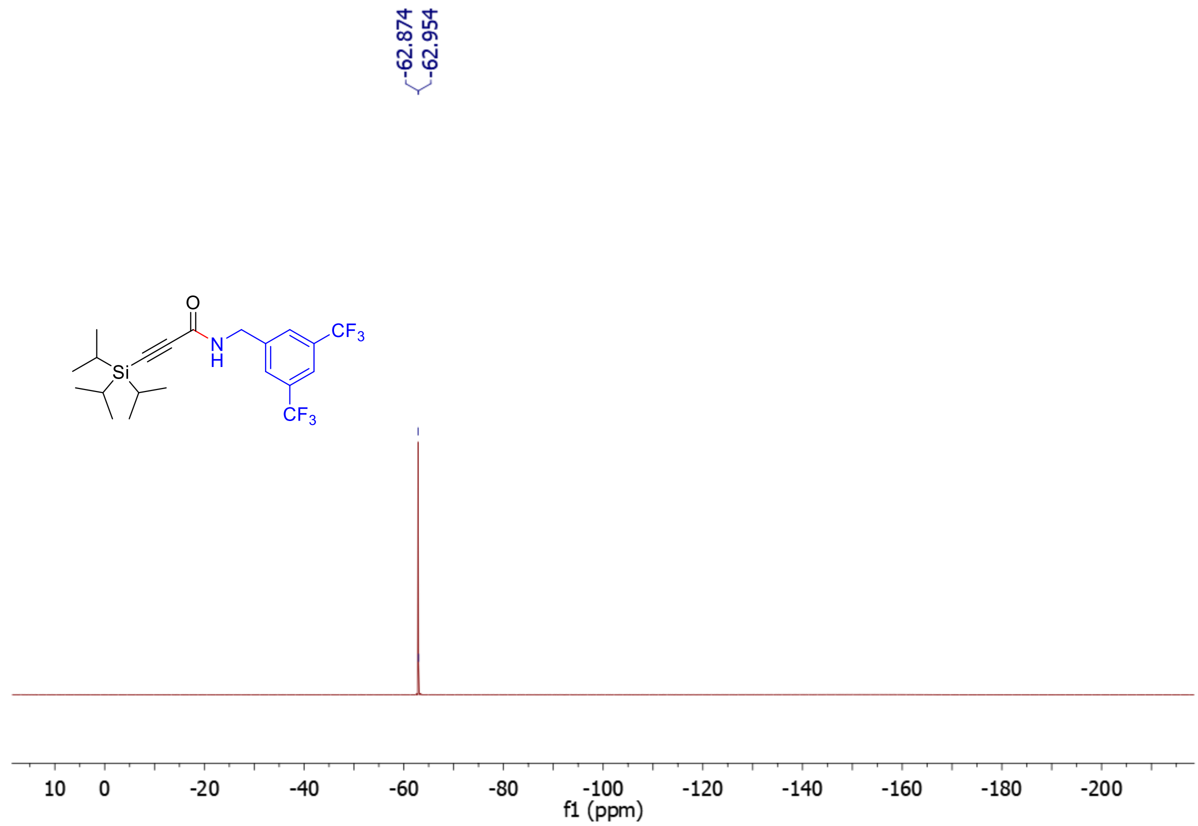

Fig. S59. ^{19}F NMR spectrum of N-(3,5-bis(trifluoromethyl)benzyl)-3-(triisopropylsilyl)propiolamide **(3i)** in $CDCl_3$.

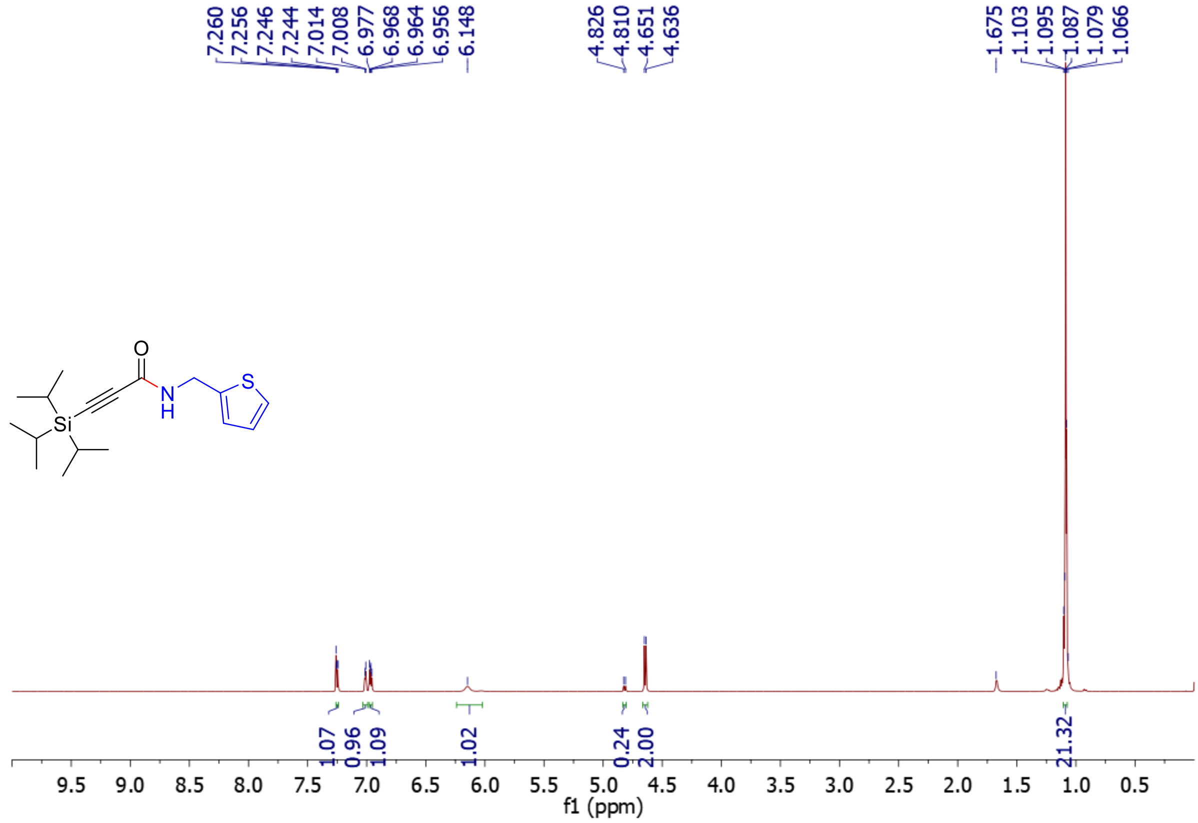

Fig. S60. ^{1}H NMR spectrum of N-(thiophen-2-ylmethyl)-3-(triisopropylsilyl)propiolamide **(3j)** in $CDCl_3$.

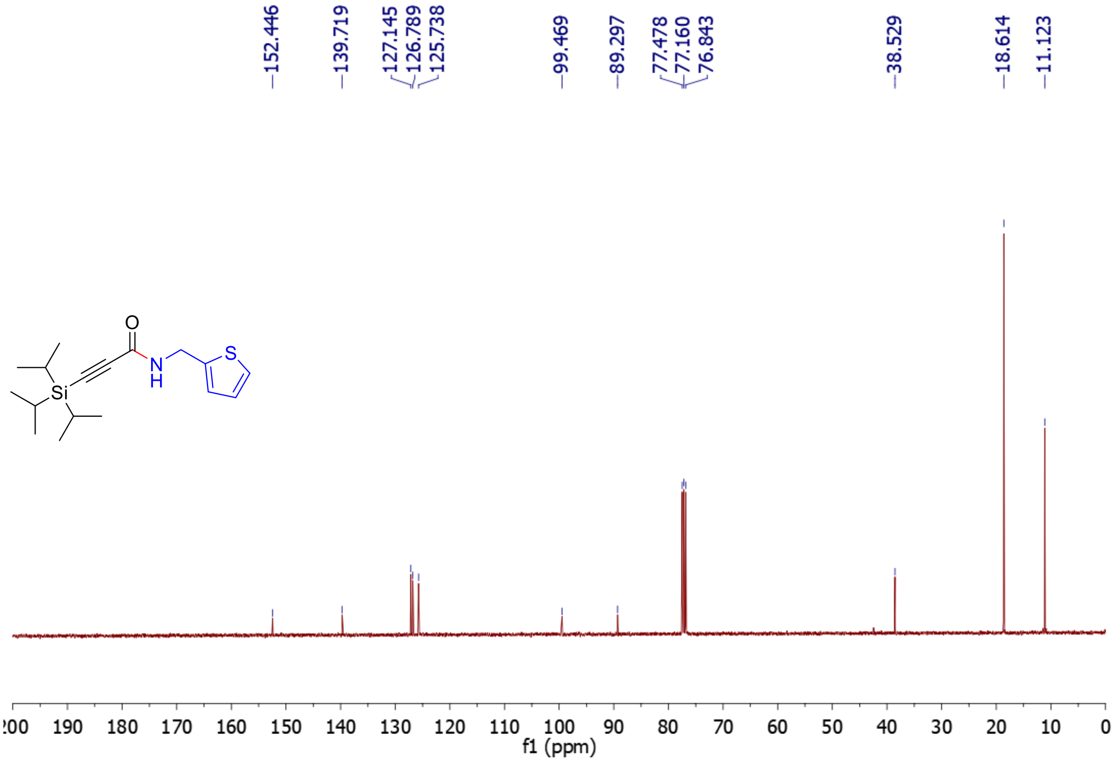

Fig. S61. ^{13}C NMR spectrum of N-(thiophen-2-ylmethyl)-3-(triisopropylsilyl)propiolamide **(3j)** in $CDCl_3$.

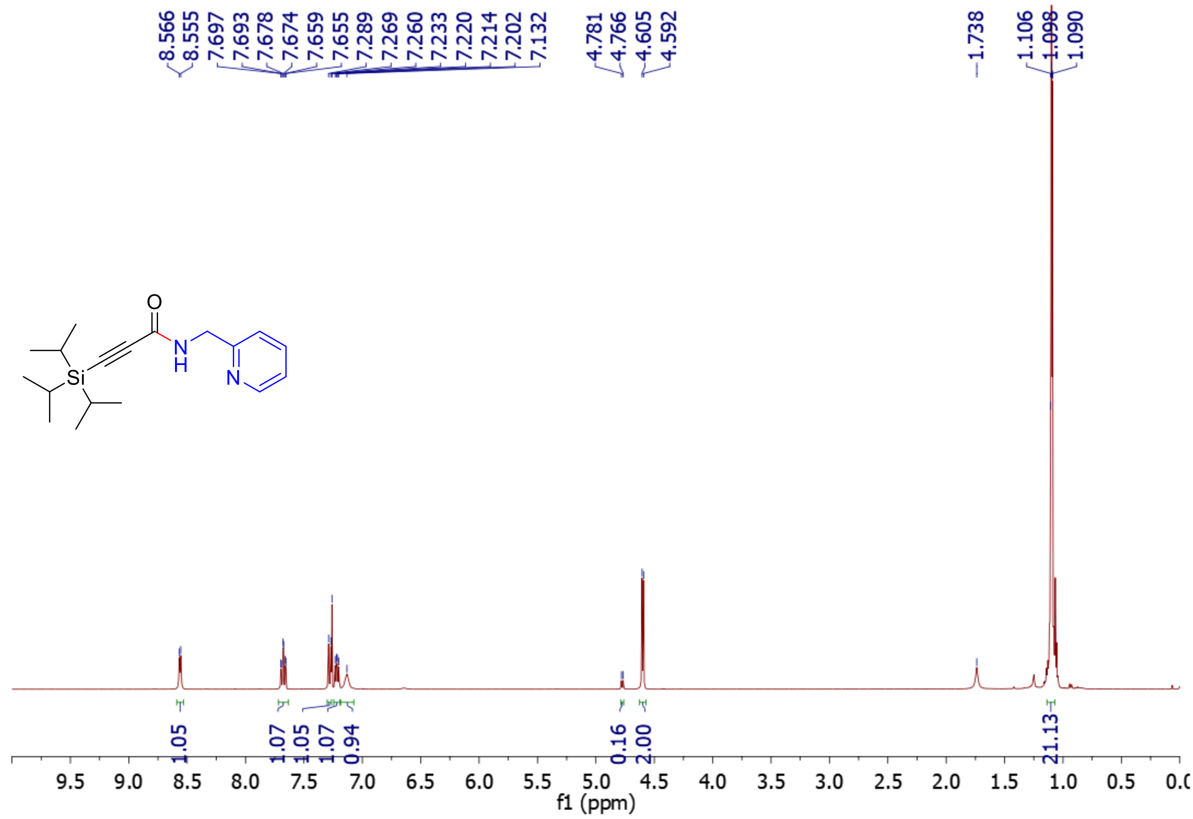

Fig. S62. ^{1}H NMR spectrum of N-(pyridin-2-ylmethyl)-3-(triisopropylsilyl)propiolamide **(3k)** in $CDCl_3$.

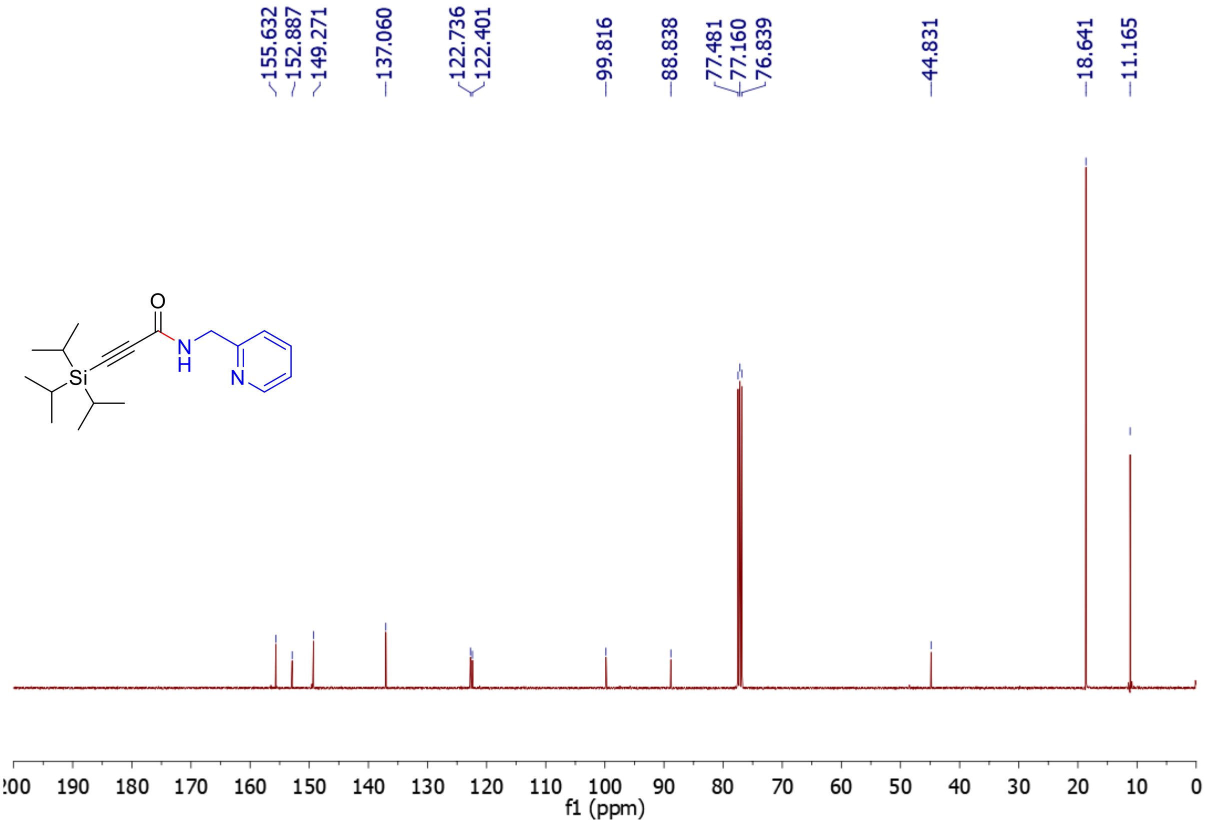

Fig. S63. ^{13}C NMR spectrum of N-(pyridin-2-ylmethyl)-3-(triisopropylsilyl)propiolamide **(3k)** in $CDCl_3$.

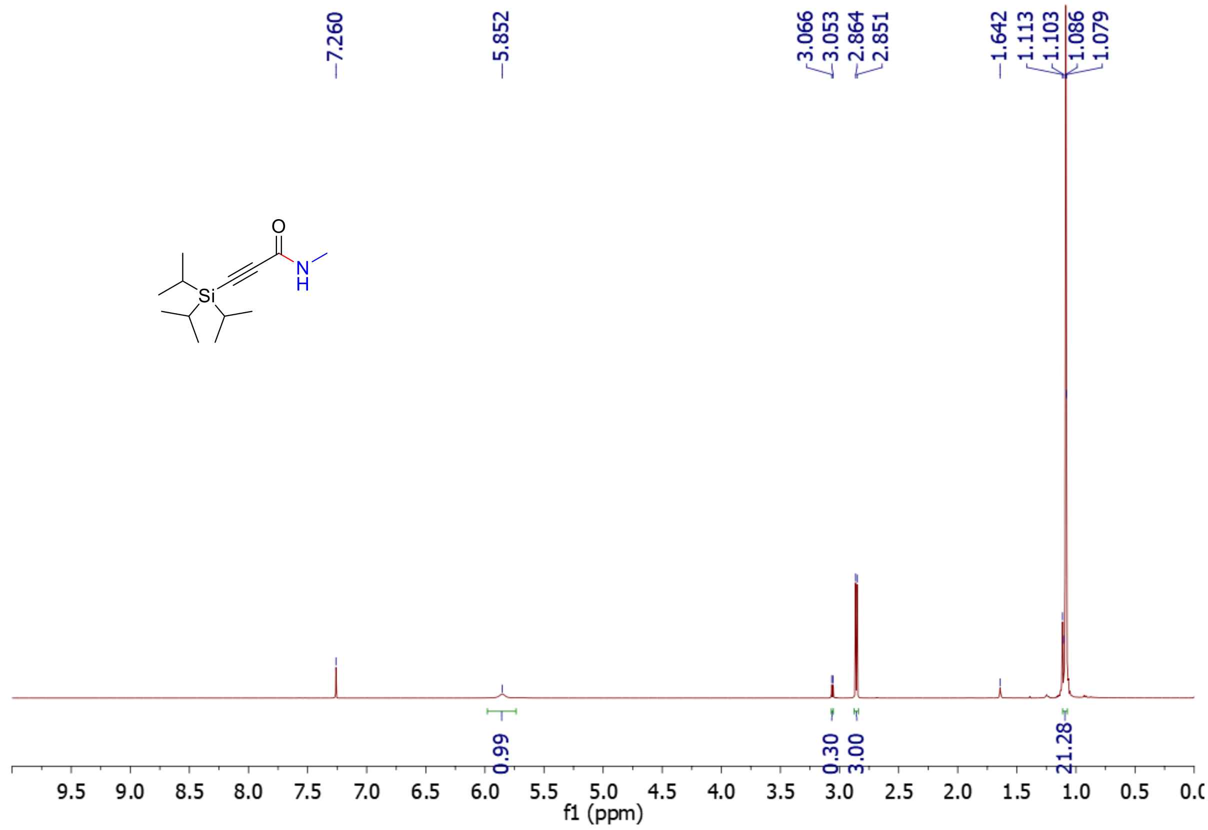

Fig. S64. ^{1}H NMR spectrum of N-methyl-3-(triisopropylsilyl)propiolamide **(3l)** in $CDCl_3$.

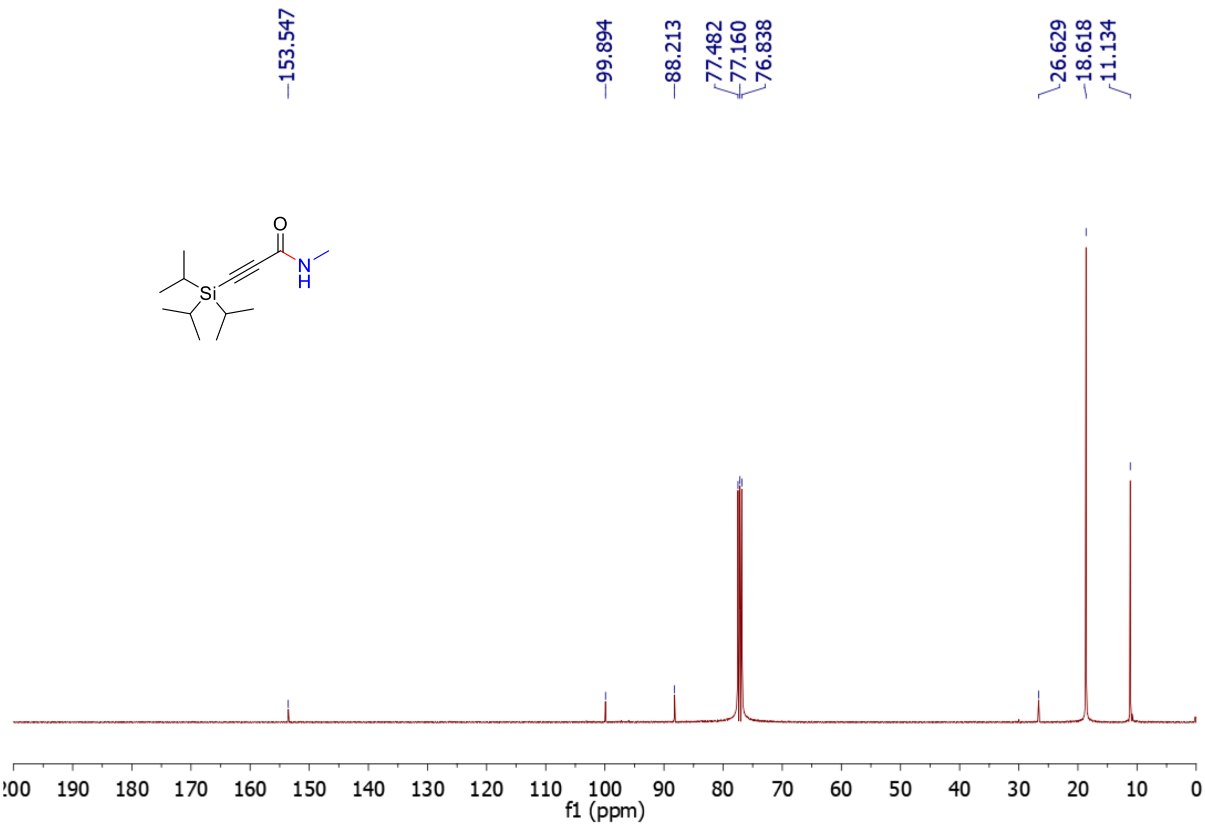

Fig. S65. ^{13}C NMR spectrum of N-methyl-3-(triisopropylsilyl)propiolamide **(3l)** in $CDCl_3$.

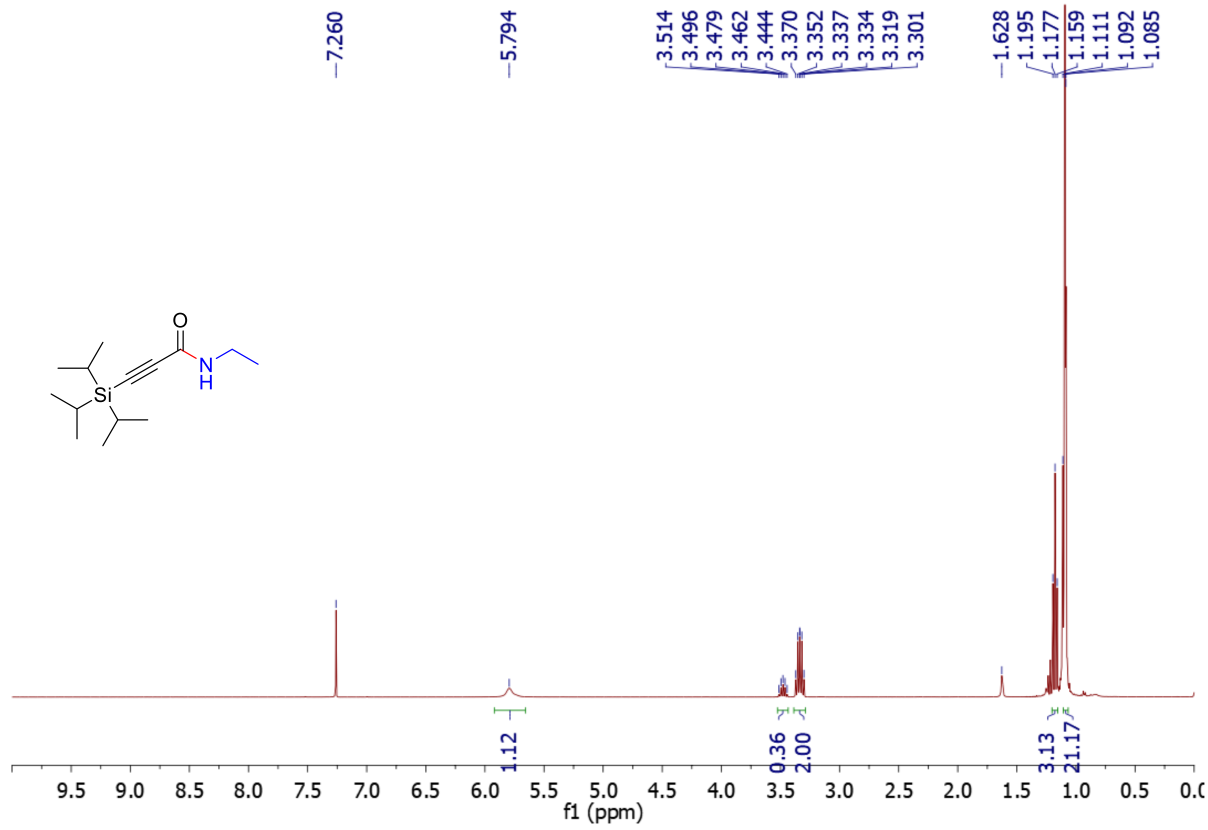

Fig. S66. ^{1}H NMR spectrum of N-ethyl-3-(triisopropylsilyl)propiolamide **(3m)** in $CDCl_3$.

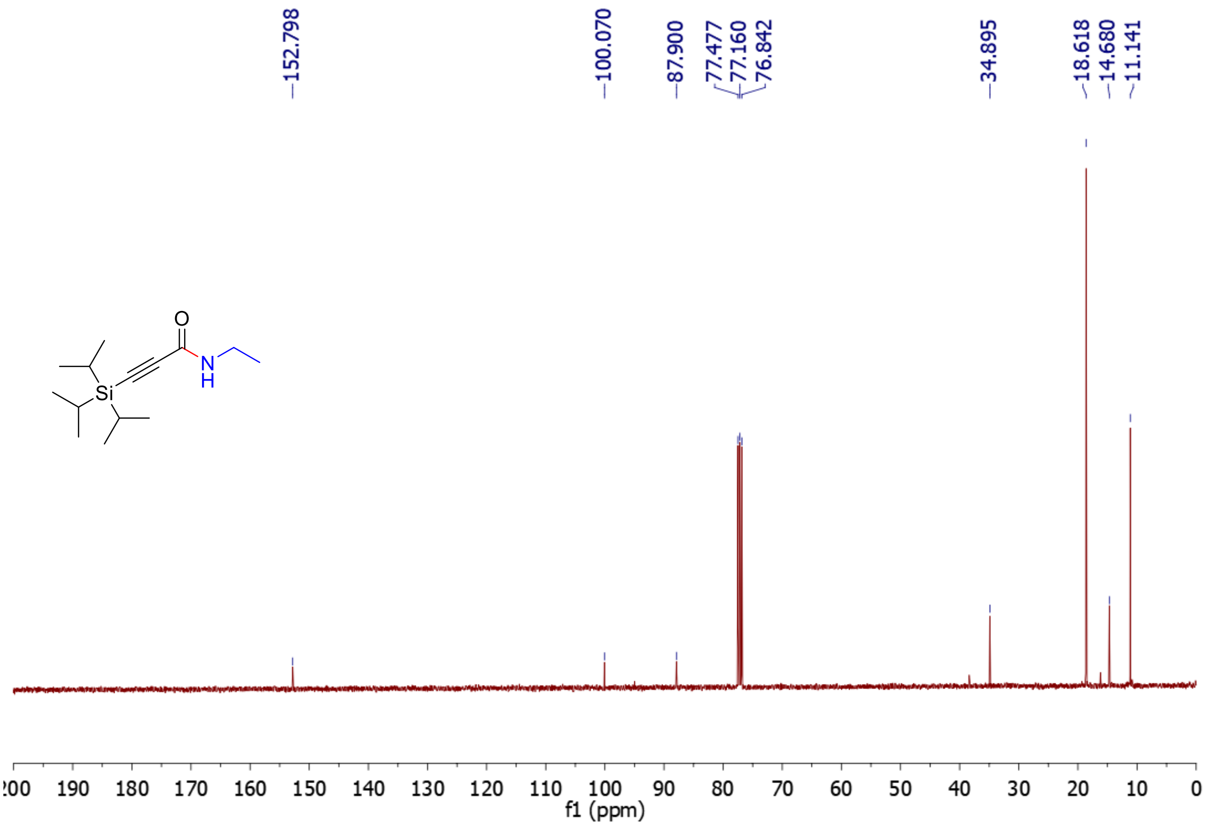

Fig. S67. ^{13}C NMR spectrum of N-ethyl-3-(triisopropylsilyl)propiolamide **(3m)** in $CDCl_3$.

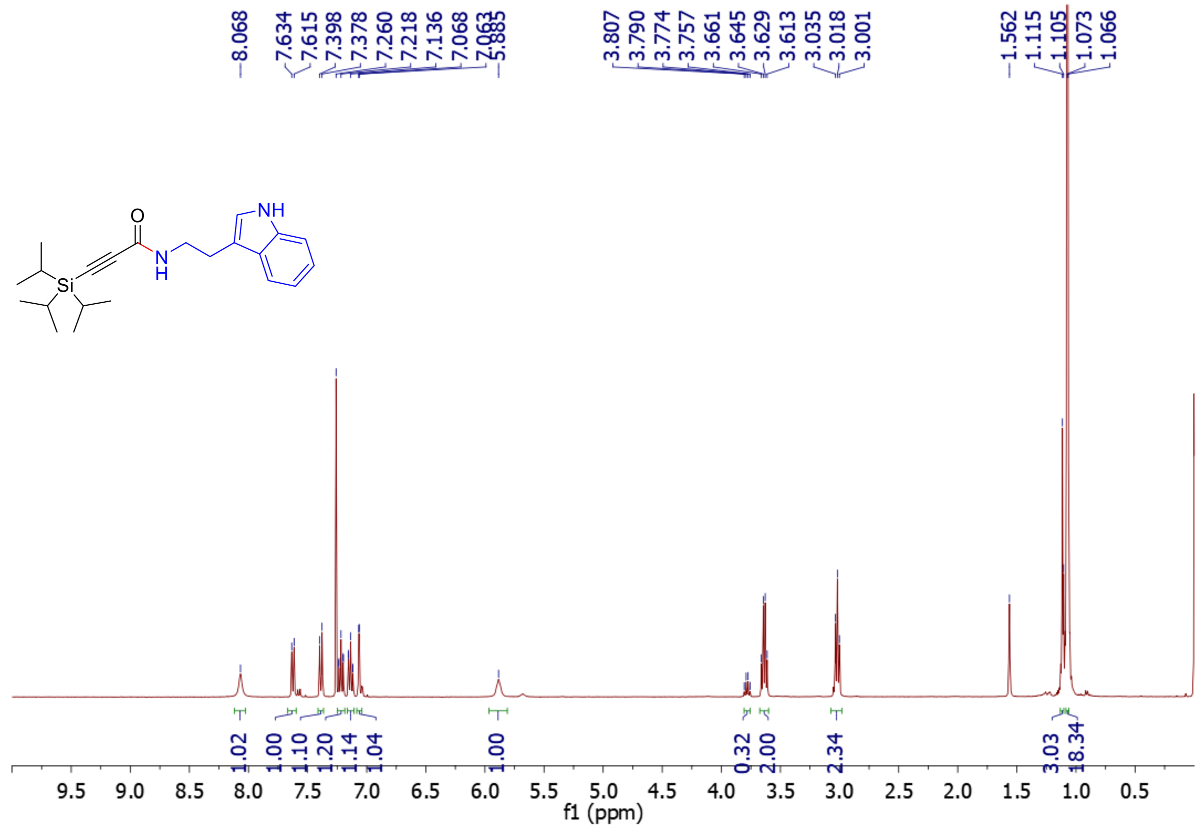

Fig. S68. ^{1}H NMR spectrum of N-(2-(1H-indol-3-yl)ethyl)-3-(triisopropylsilyl)propiolamide **(3n)** in $CDCl_3$.

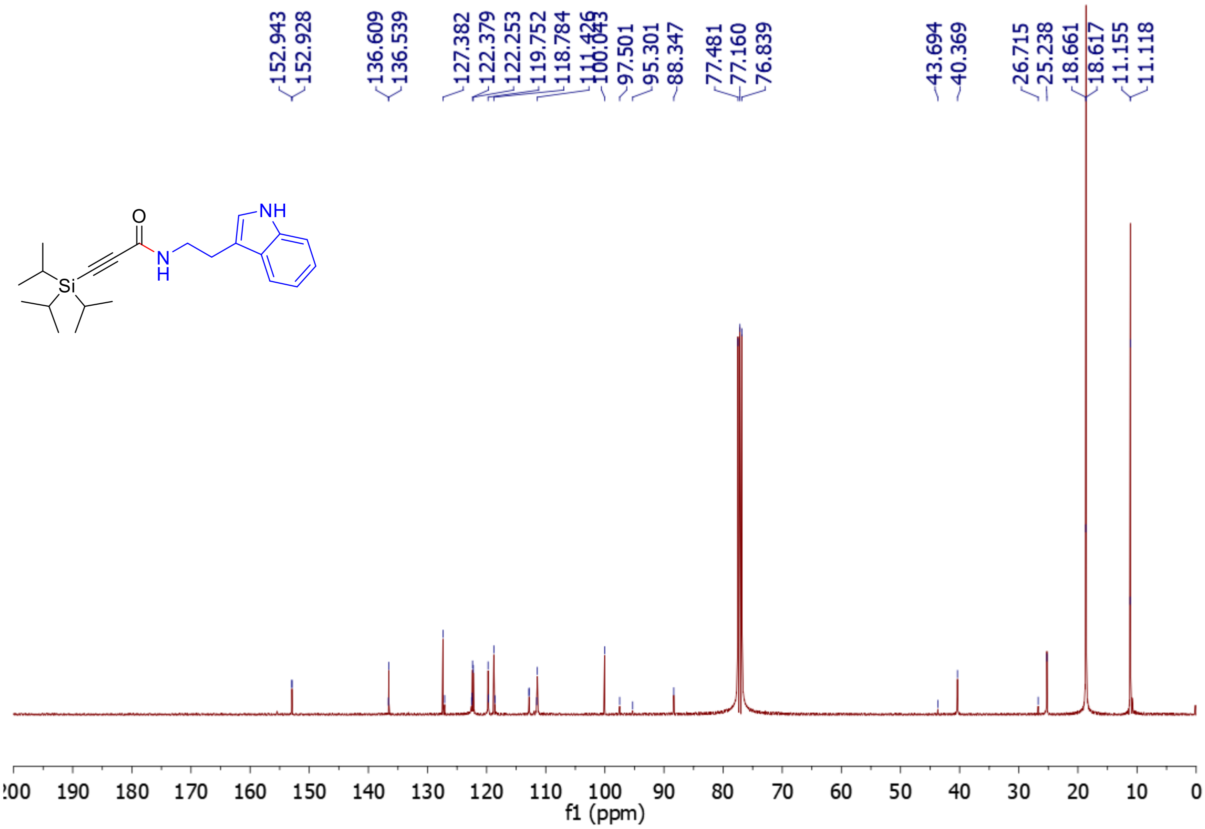

Fig. S69. ^{13}C NMR spectrum of N-(2-(1H-indol-3-yl)ethyl)-3-(triisopropylsilyl)propiolamide **(3n)** in $CDCl_3$.

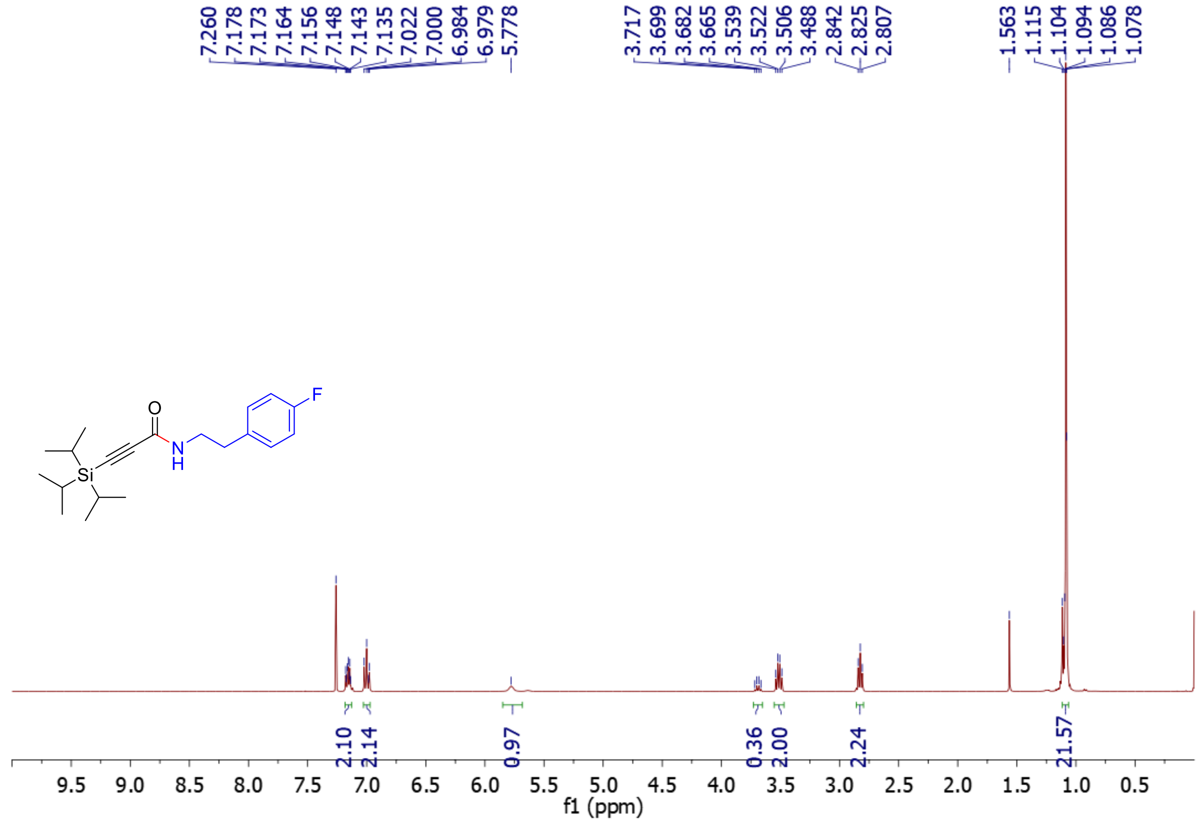

Fig. S70. ^{1}H NMR spectrum of N-(4-fluorophenethyl)-3-(triisopropylsilyl)propiolamide **(3o)** in $CDCl_3$.

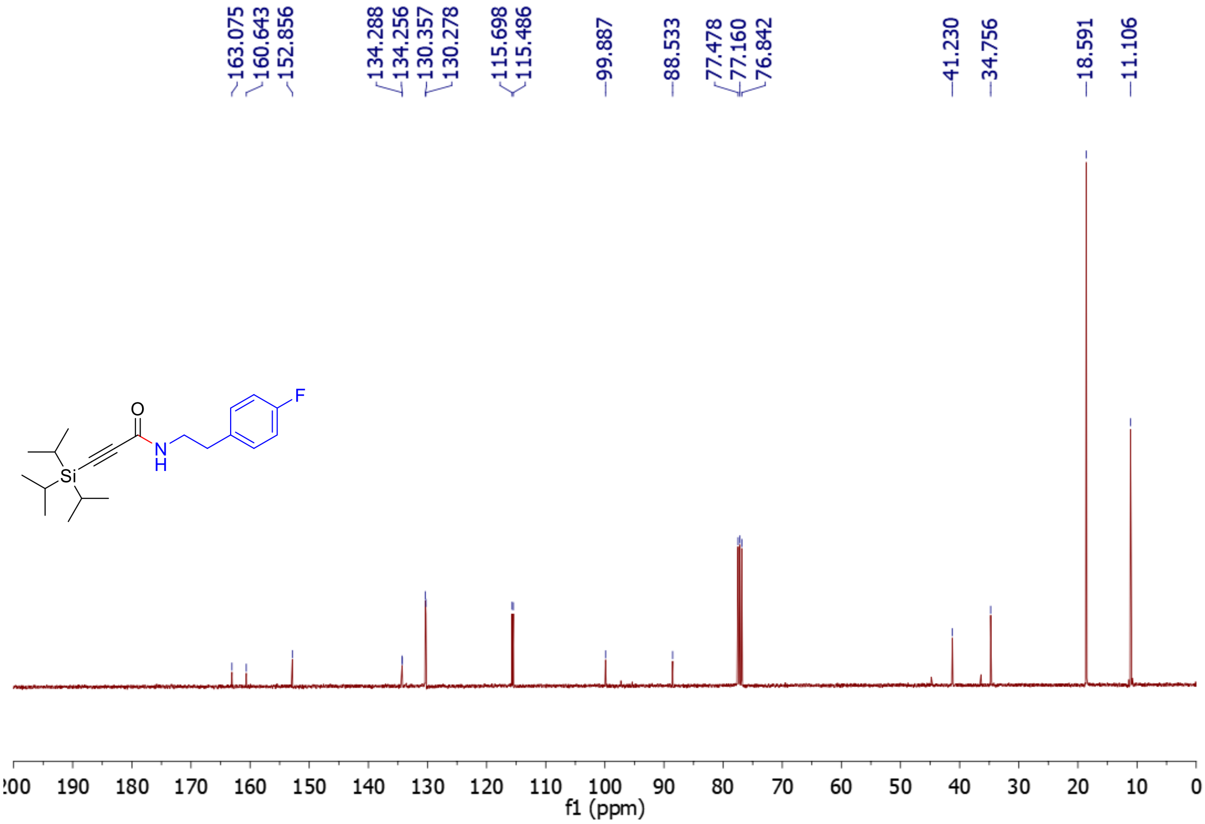

Fig. S71. ^{13}C NMR spectrum of N-(4-fluorophenethyl)-3-(triisopropylsilyl)propiolamide **(3o)** in $CDCl_3$.

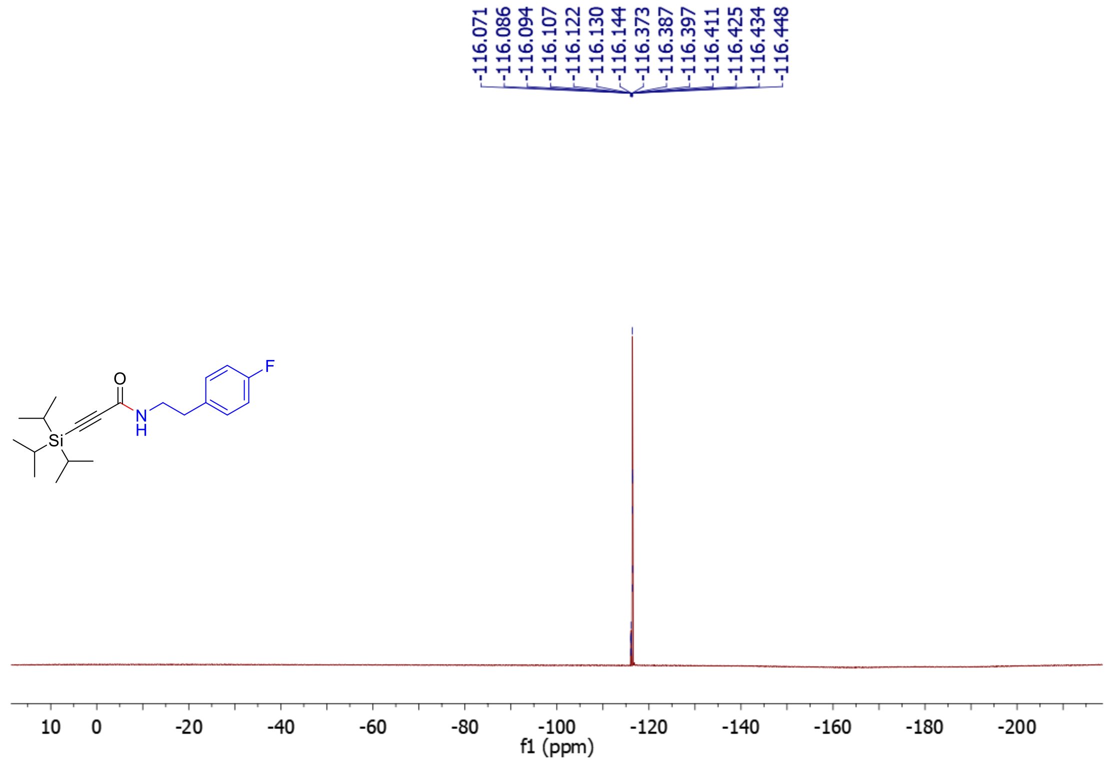

Fig. S72. ^{19}F NMR spectrum of N-(4-fluorophenethyl)-3-(triisopropylsilyl)propiolamide **(3o)** in $CDCl_3$.

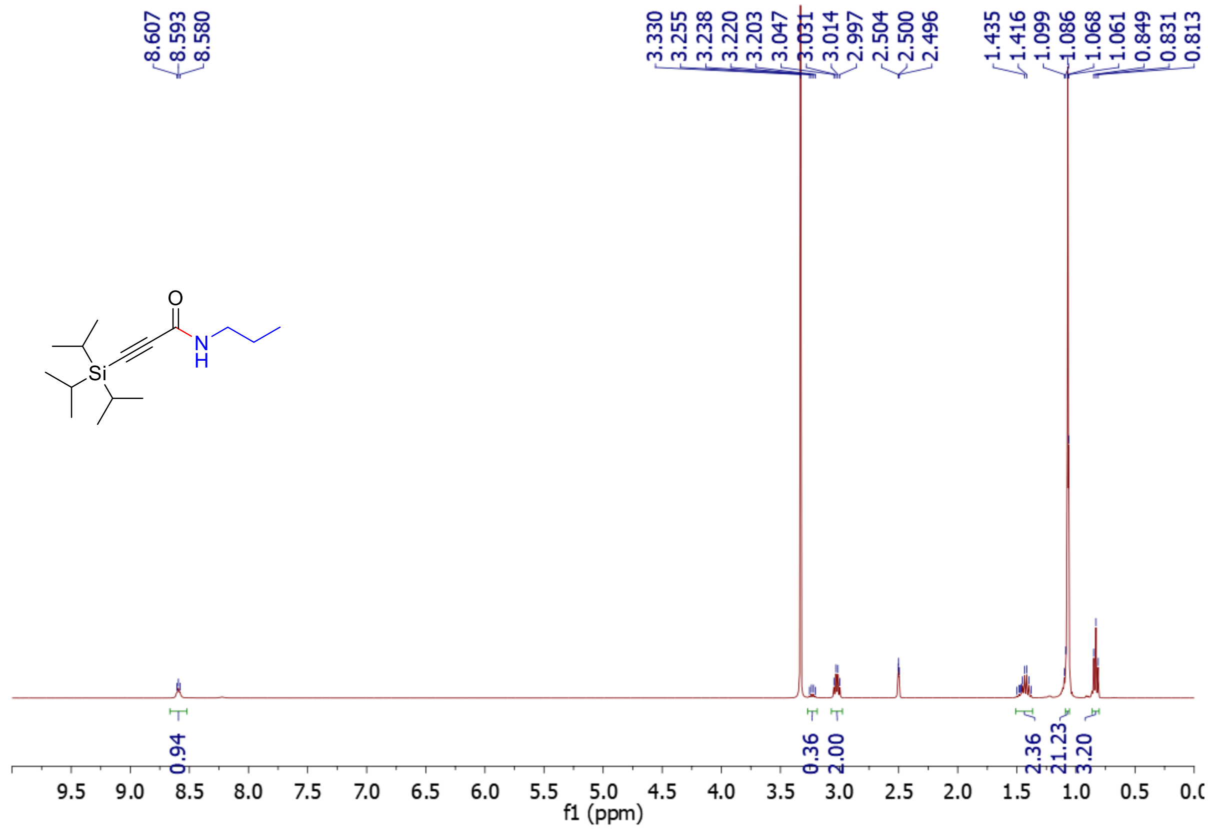

Fig. S73. ^{1}H NMR spectrum of N-propyl-3-(triisopropylsilyl)propiolamide **(3p)** in DMSO.

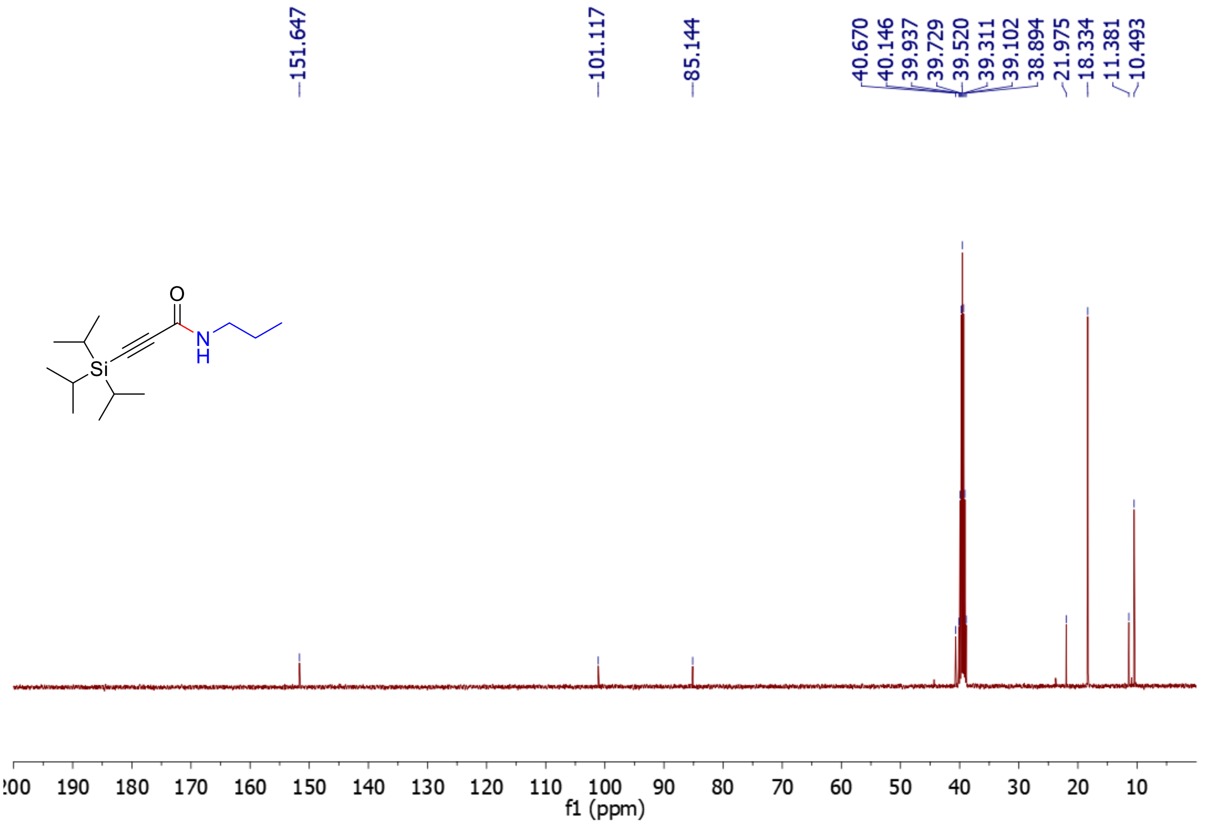

Fig. S74. ^{13}C NMR spectrum of N-propyl-3-(triisopropylsilyl)propiolamide **(3p)** in DMSO.

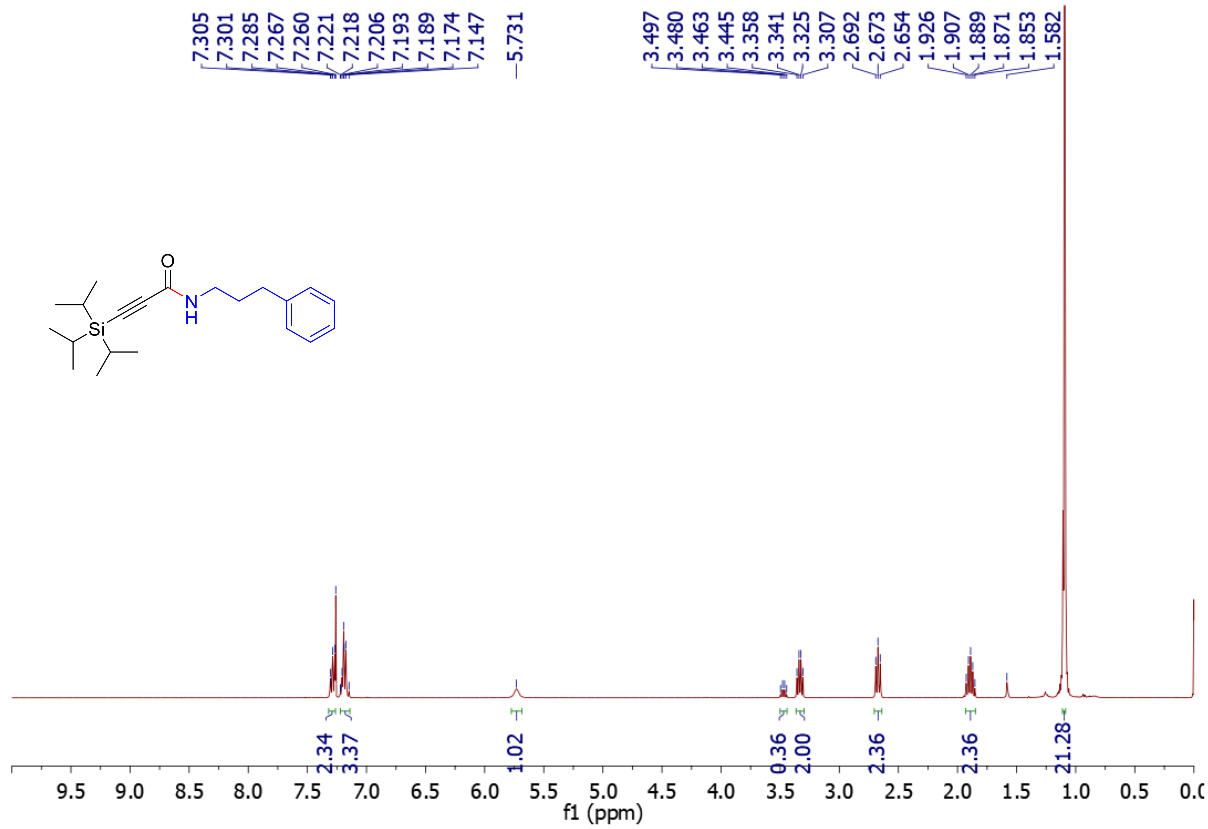

Fig. S75. ^{1}H NMR spectrum of N-(3-phenylpropyl)-3-(triisopropylsilyl)propiolamide **(3q)** in $CDCl_3$.

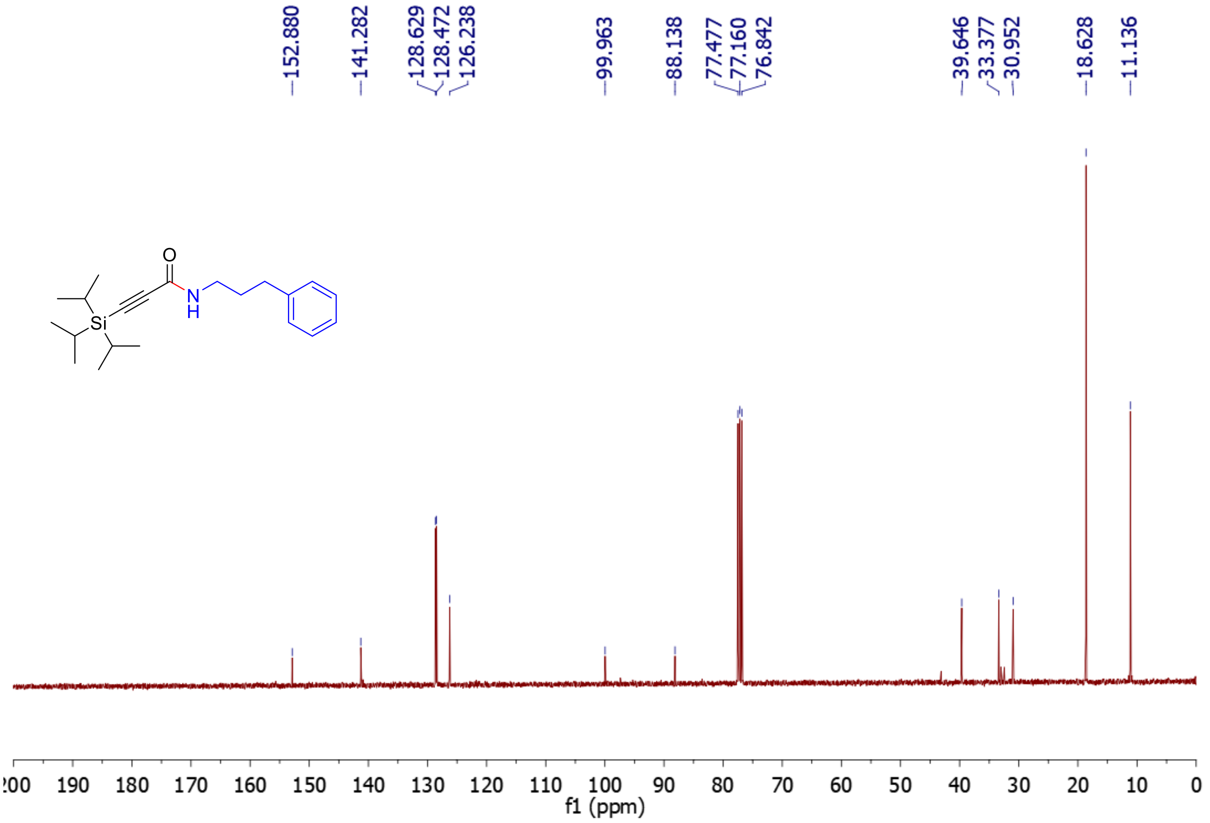

Fig. S76. ^{13}C NMR spectrum of N-(3-phenylpropyl)-3-(triisopropylsilyl)propiolamide **(3q)** in $CDCl_3$.

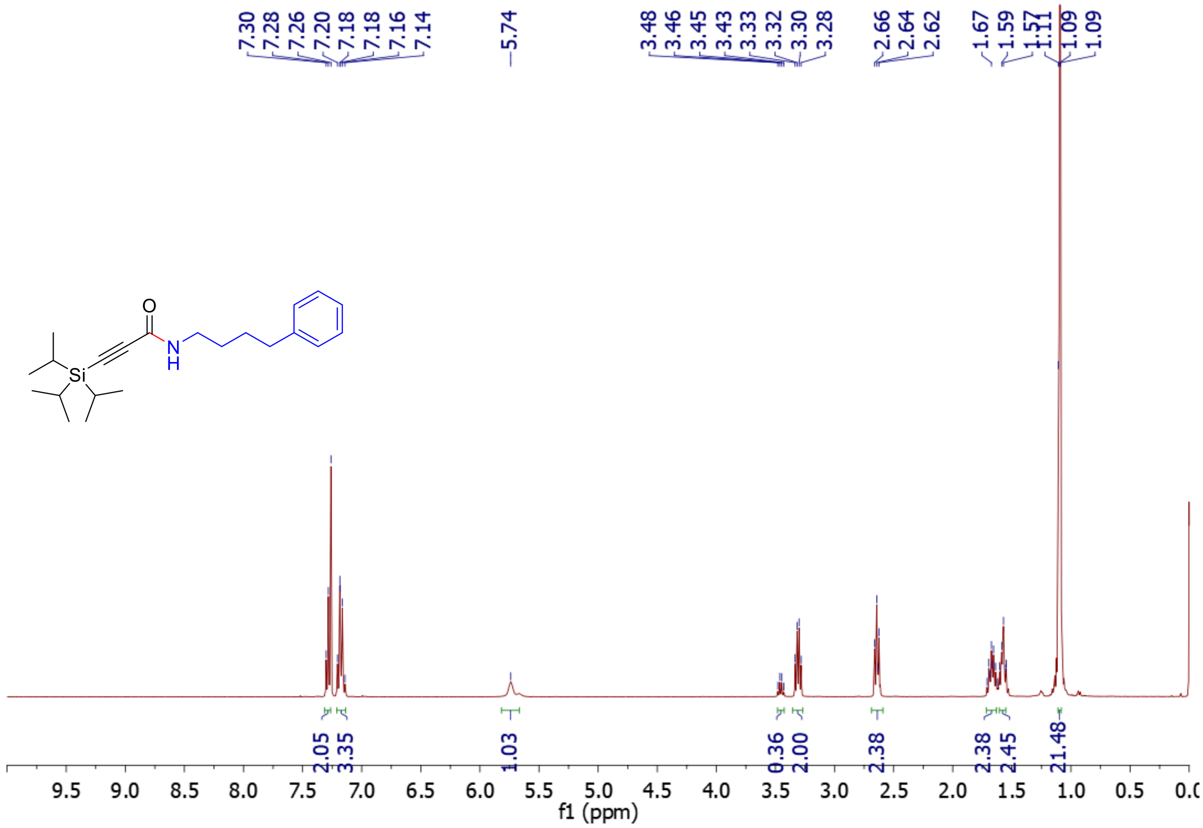

Fig. S77. ^{1}H NMR spectrum of N-(4-phenylbutyl)-3-(triisopropylsilyl)propiolamide **(3r)** in $CDCl_3$.

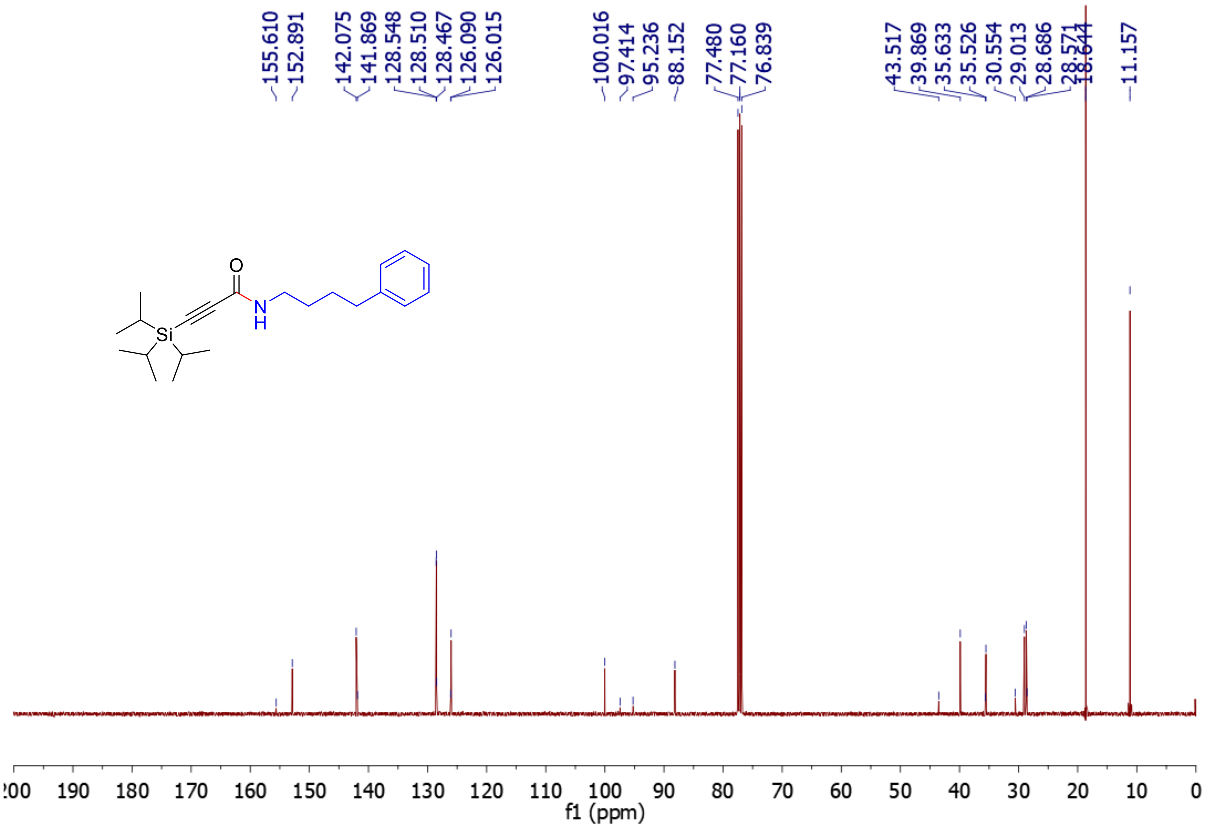

Fig. S78. ^{13}C NMR spectrum of N-(4-phenylbutyl)-3-(triisopropylsilyl)propiolamide **(3r)** in $CDCl_3$.

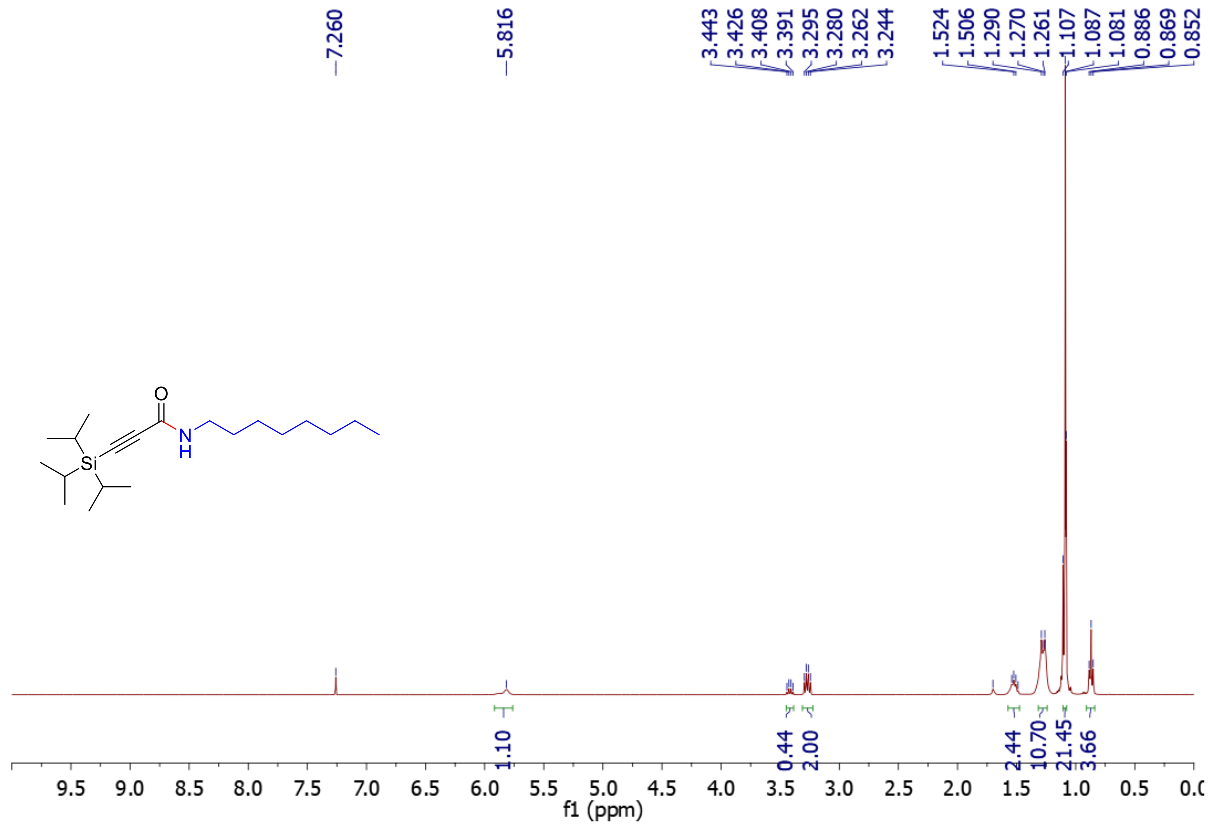

Fig. S79. ^{1}H NMR spectrum of N-octyl-3-(triisopropylsilyl)propiolamide **(3s)** in $CDCl_3$.

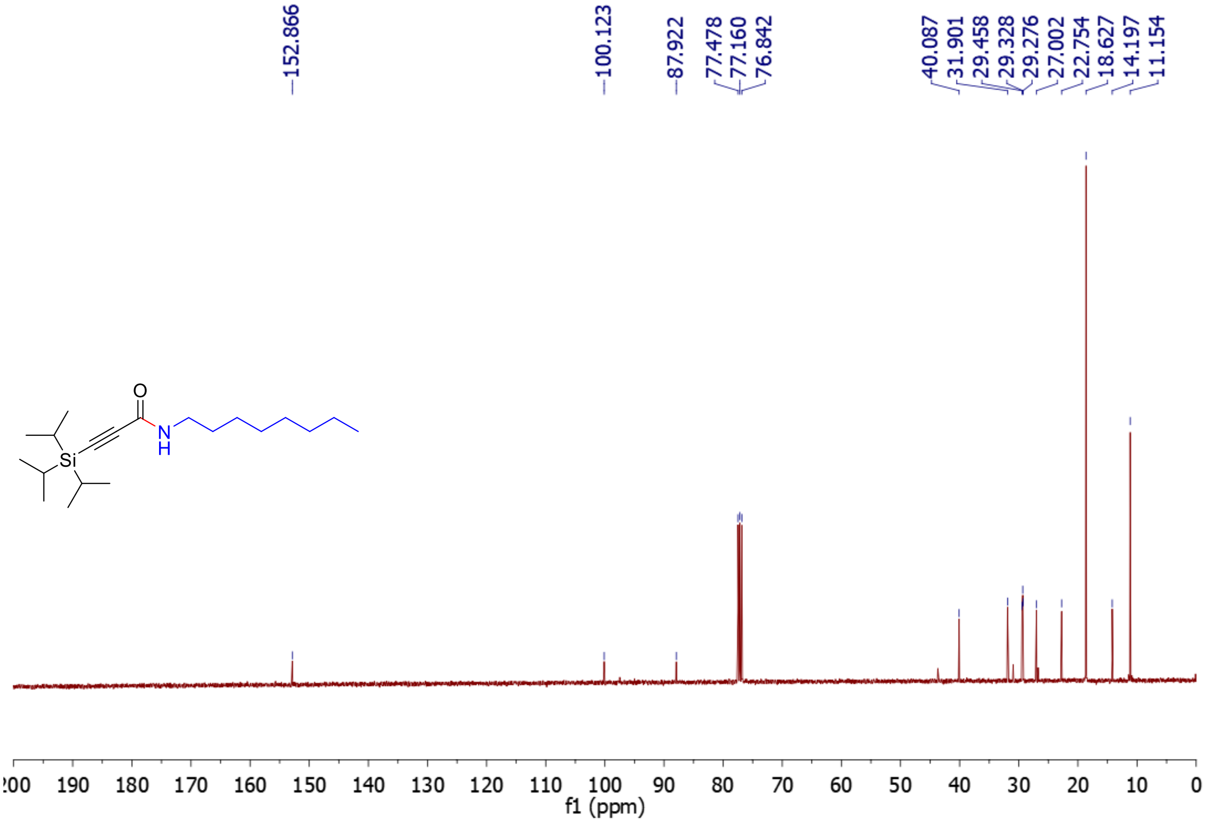

Fig. S80. ^{13}C NMR spectrum of N-octyl-3-(triisopropylsilyl)propiolamide **(3s)** in $CDCl_3$.

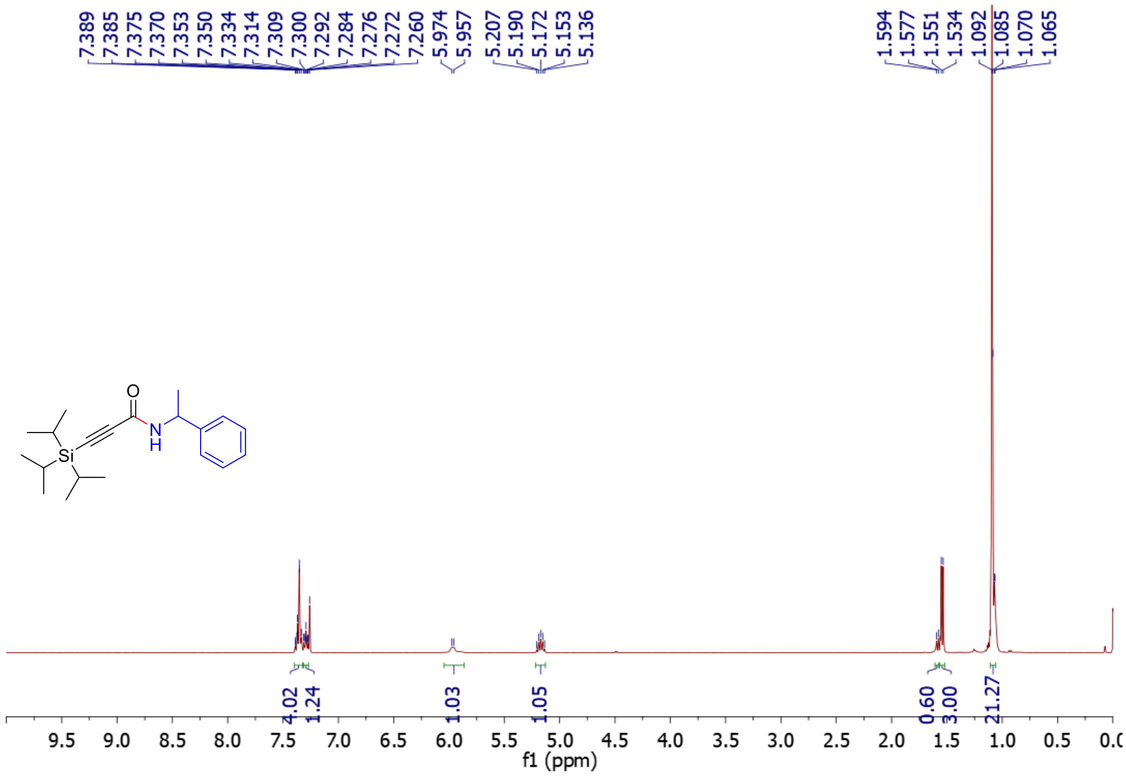

Fig. S81. ^{1}H NMR spectrum of N-(1-phenylethyl)-3-(triisopropylsilyl)propiolamide **(3t)** in $CDCl_3$.

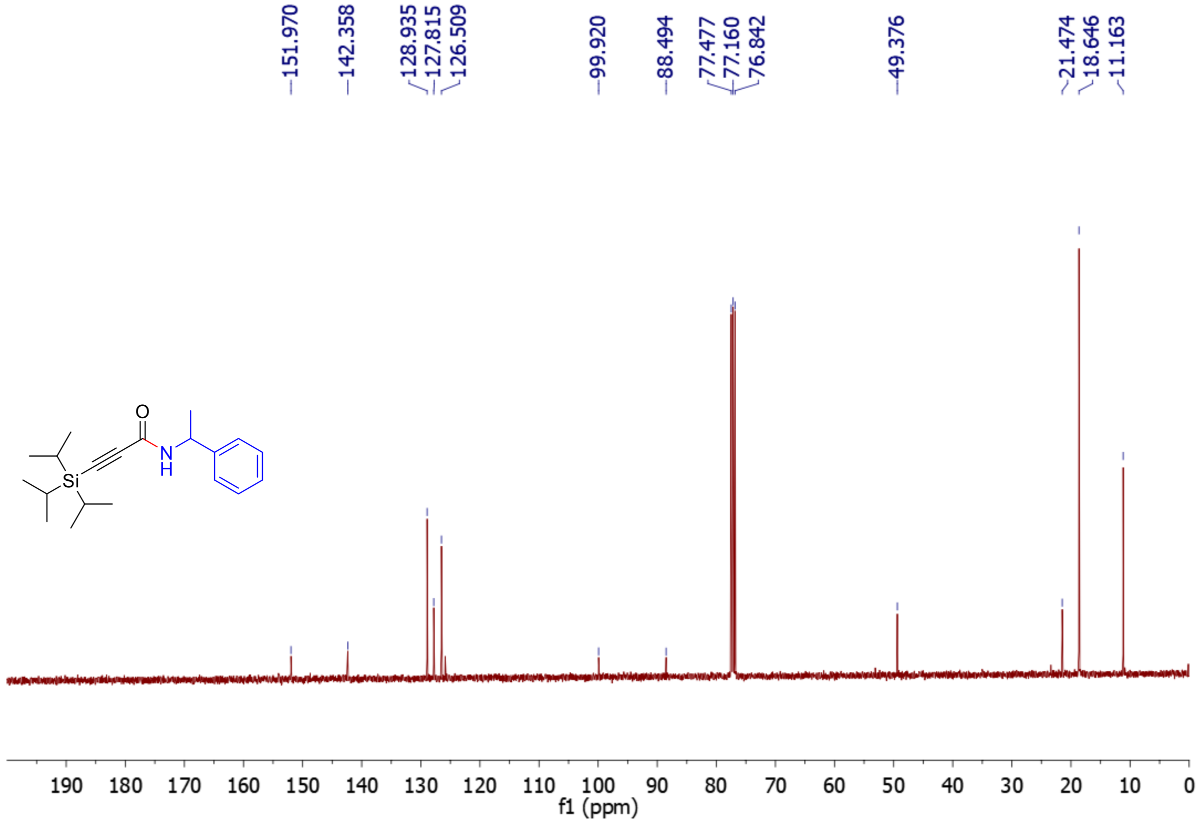

Fig. S82. ^{13}C NMR spectrum of N-(1-phenylethyl)-3-(triisopropylsilyl)propiolamide **(3t)** in $CDCl_3$.

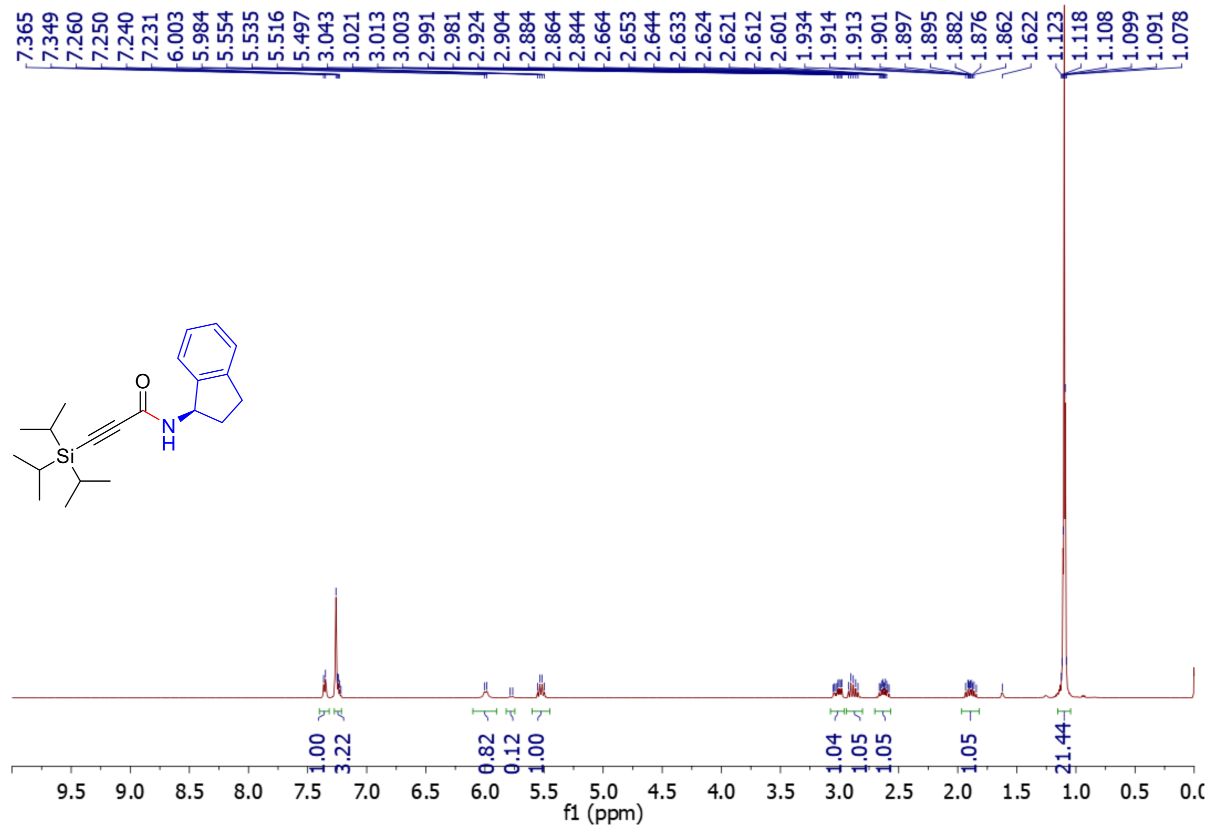

Fig. S83. ^{1}H NMR spectrum of (S)-N-(2,3-dihydro-1H-inden-1-yl)-3-(triisopropylsilyl)propiolamide **(3u)** in $CDCl_3$.

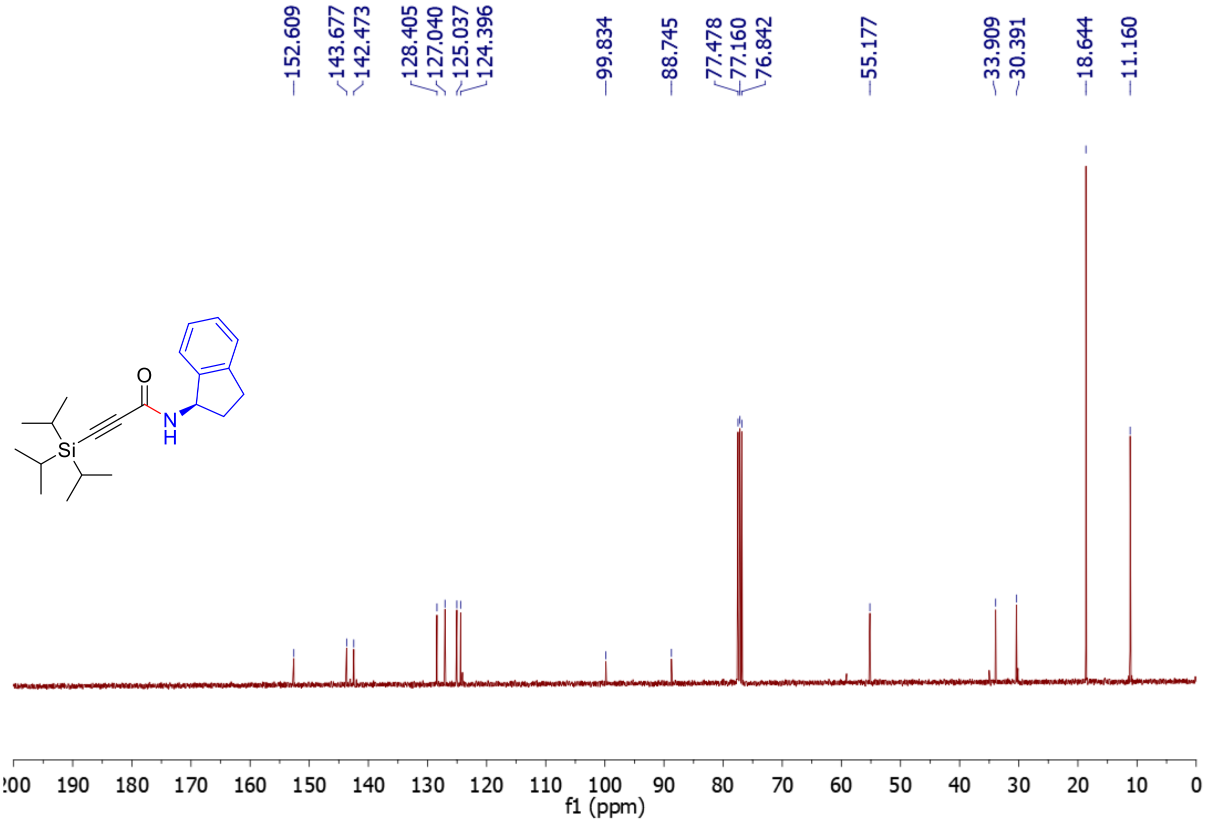

Fig. S84. ^{13}C NMR spectrum of (S)-N-(2,3-dihydro-1H-inden-1-yl)-3-(triisopropylsilyl)propiolamide **(3u)** in $CDCl_3$.

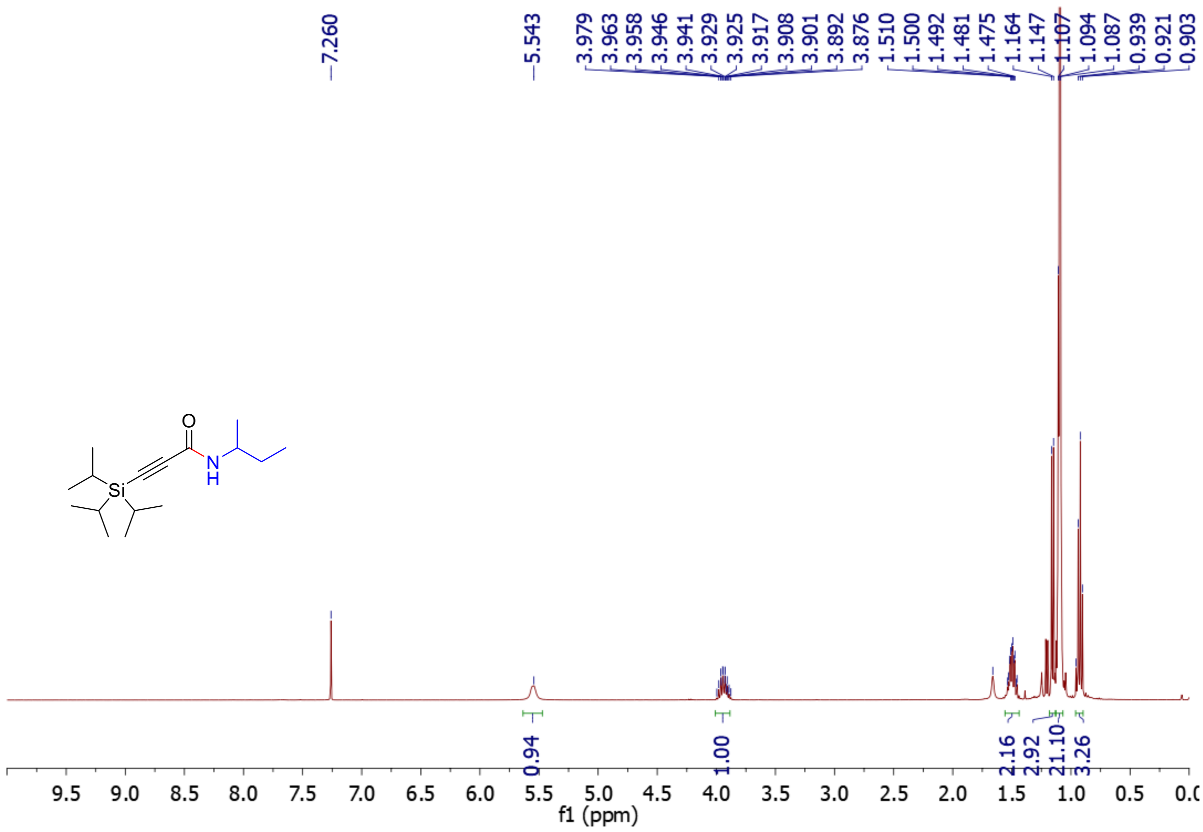

Fig. S85. ^{1}H NMR spectrum of N-(sec-butyl)-3-(triisopropylsilyl)propiolamide **(3v)** in $CDCl_3$.

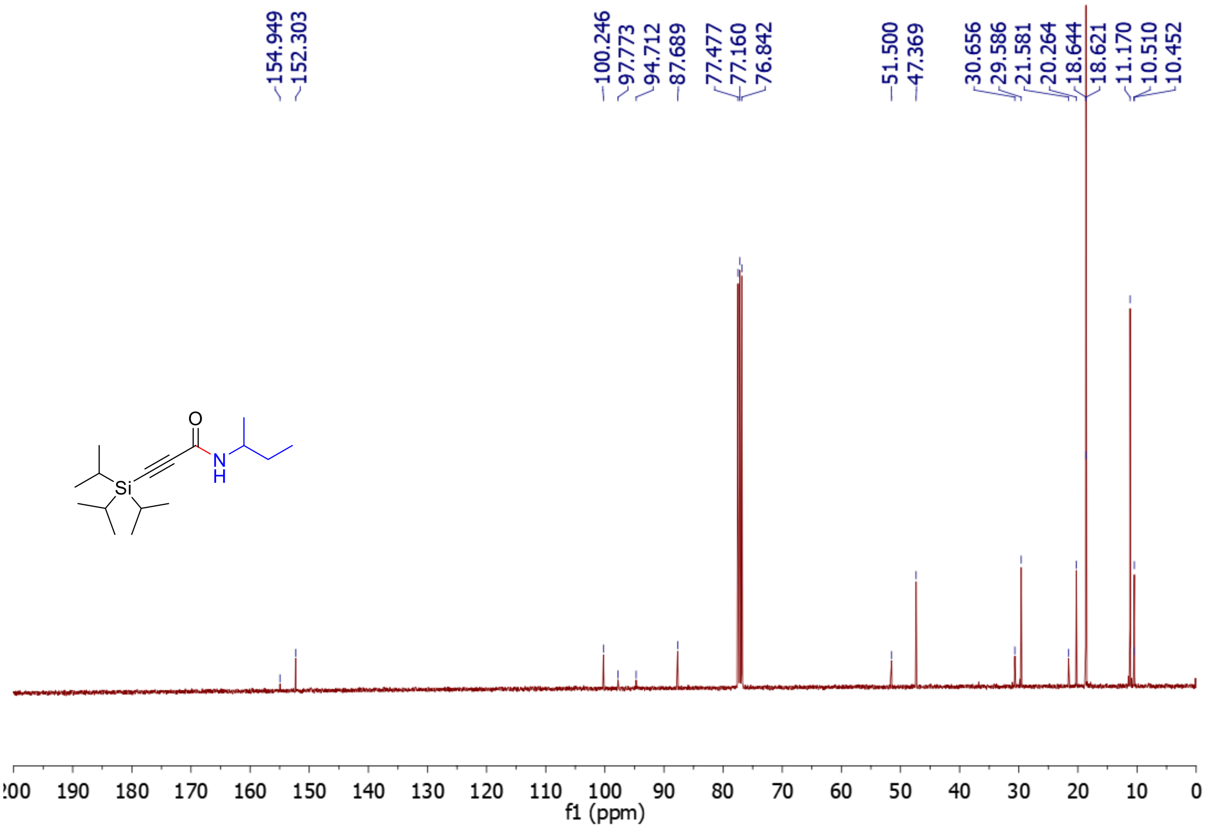

Fig. S86. ^{13}C NMR spectrum of N-(sec-butyl)-3-(triisopropylsilyl)propiolamide **(3v)** in $CDCl_3$.

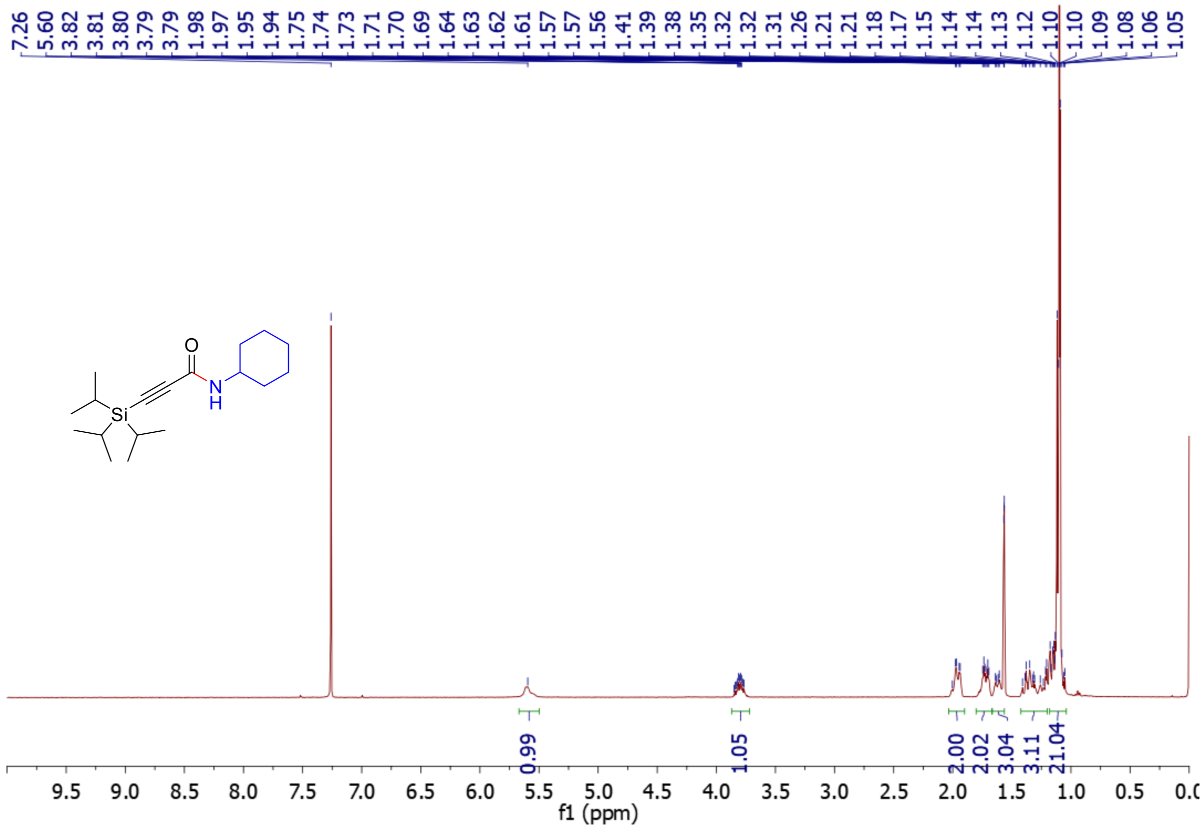

Fig. S87. ^{1}H NMR spectrum of N-cyclohexyl-3-(triisopropylsilyl)propiolamide **(3w)** in $CDCl_3$.

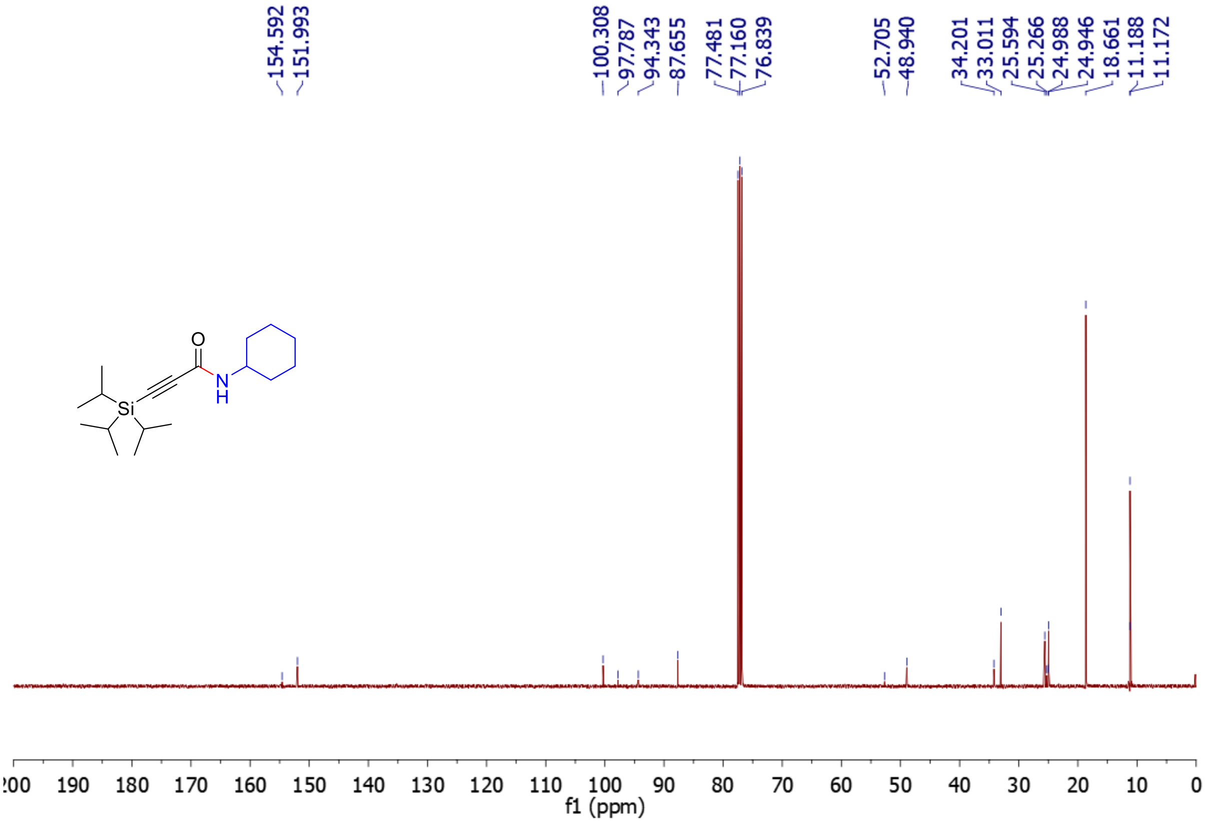

Fig. S88. ^{13}C NMR spectrum of N-cyclohexyl-3-(triisopropylsilyl)propiolamide **(3w)** in $CDCl_3$.

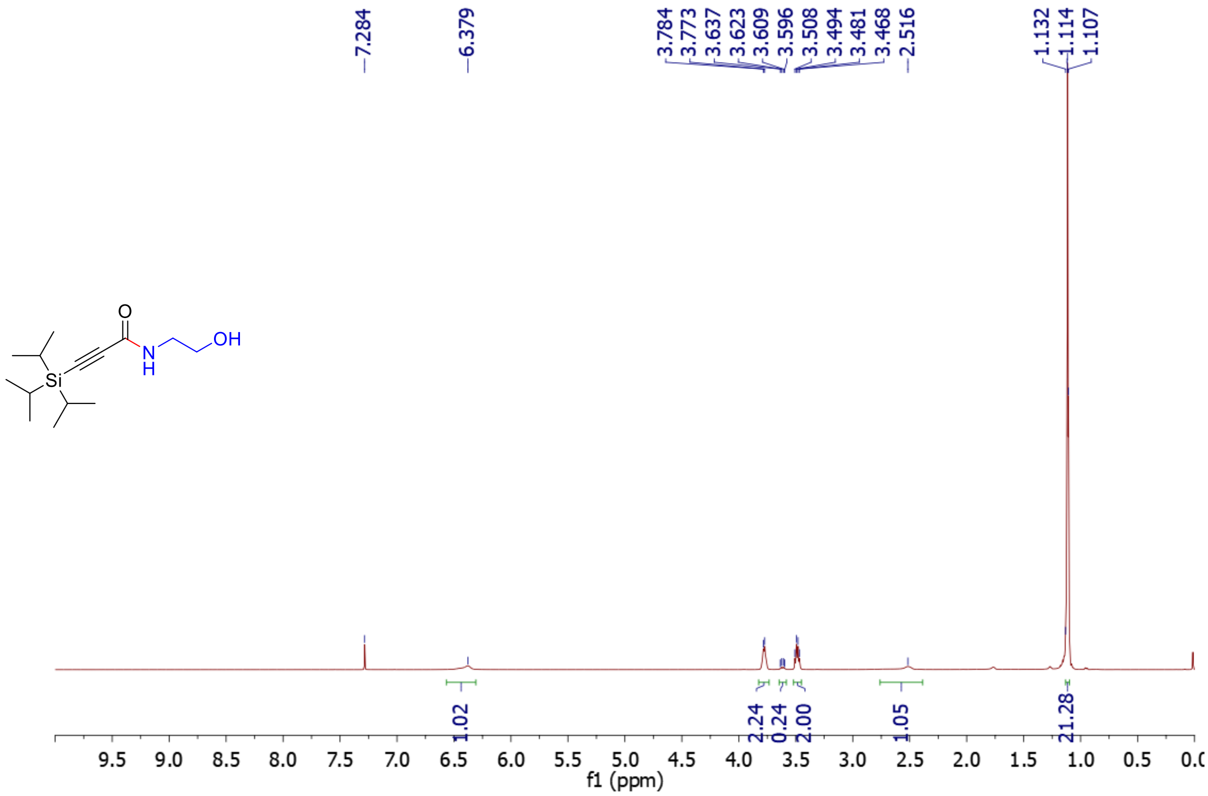

Fig. S89. ^{1}H NMR spectrum of N-(2-hydroxyethyl)-3-(triisopropylsilyl)propiolamide **(3x)** in $CDCl_3$.

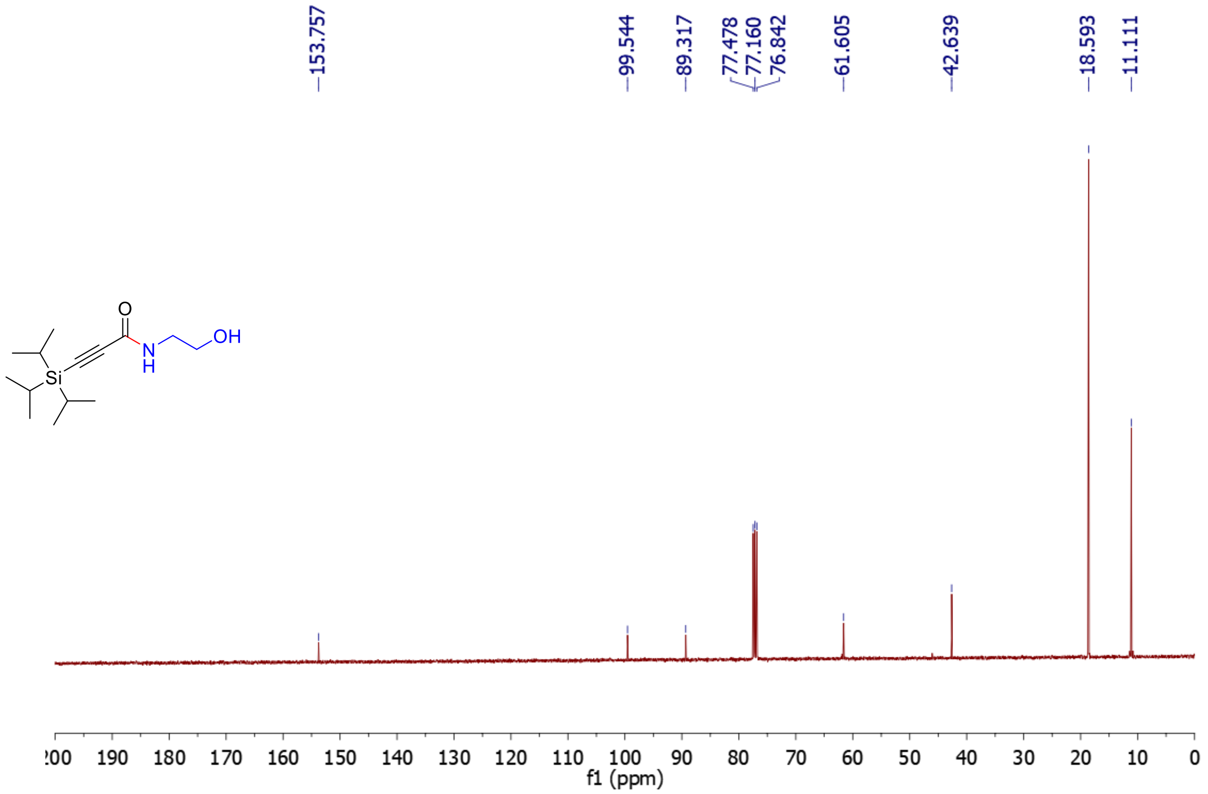

Fig. S90. ^{13}C NMR spectrum of N-(2-hydroxyethyl)-3-(triisopropylsilyl)propiolamide **(3x)** in $CDCl_3$.

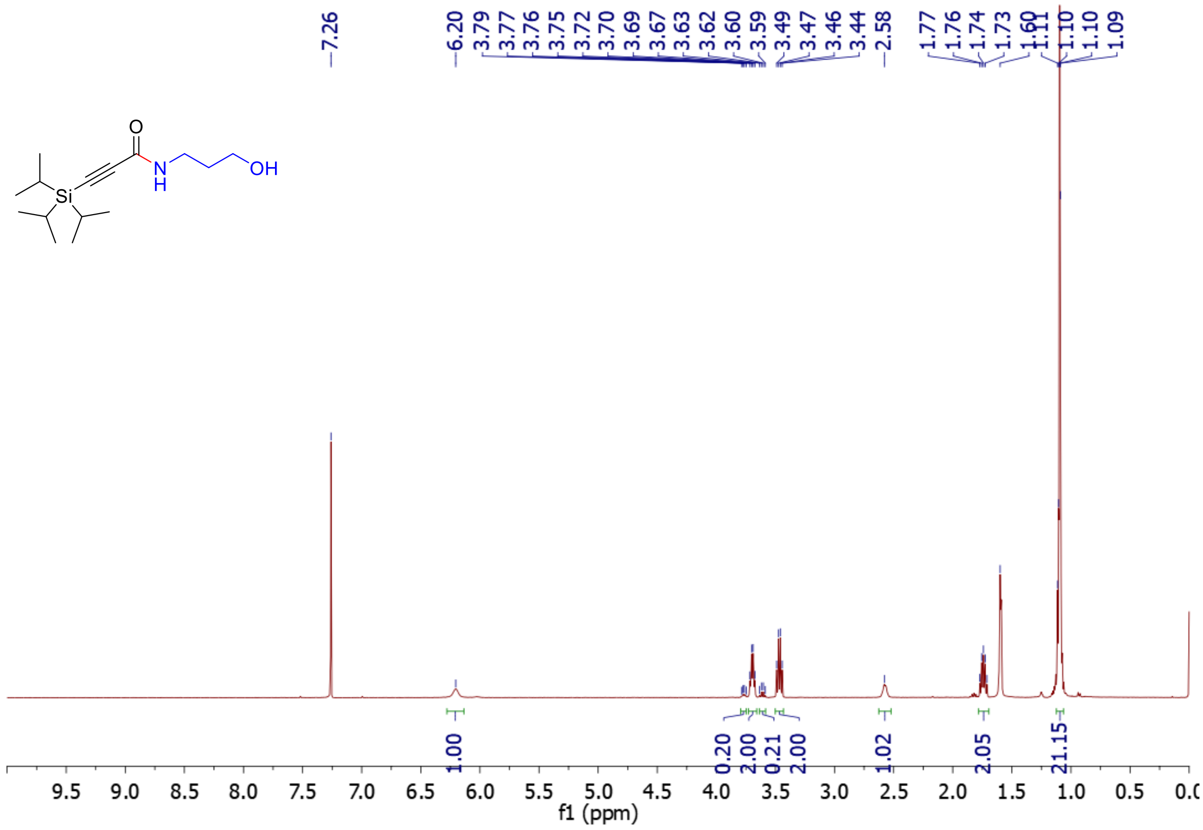

Fig. S91. ^{1}H NMR spectrum of N-(3-hydroxypropyl)-3-(triisopropylsilyl)propiolamide **(3y)** in $CDCl_3$.

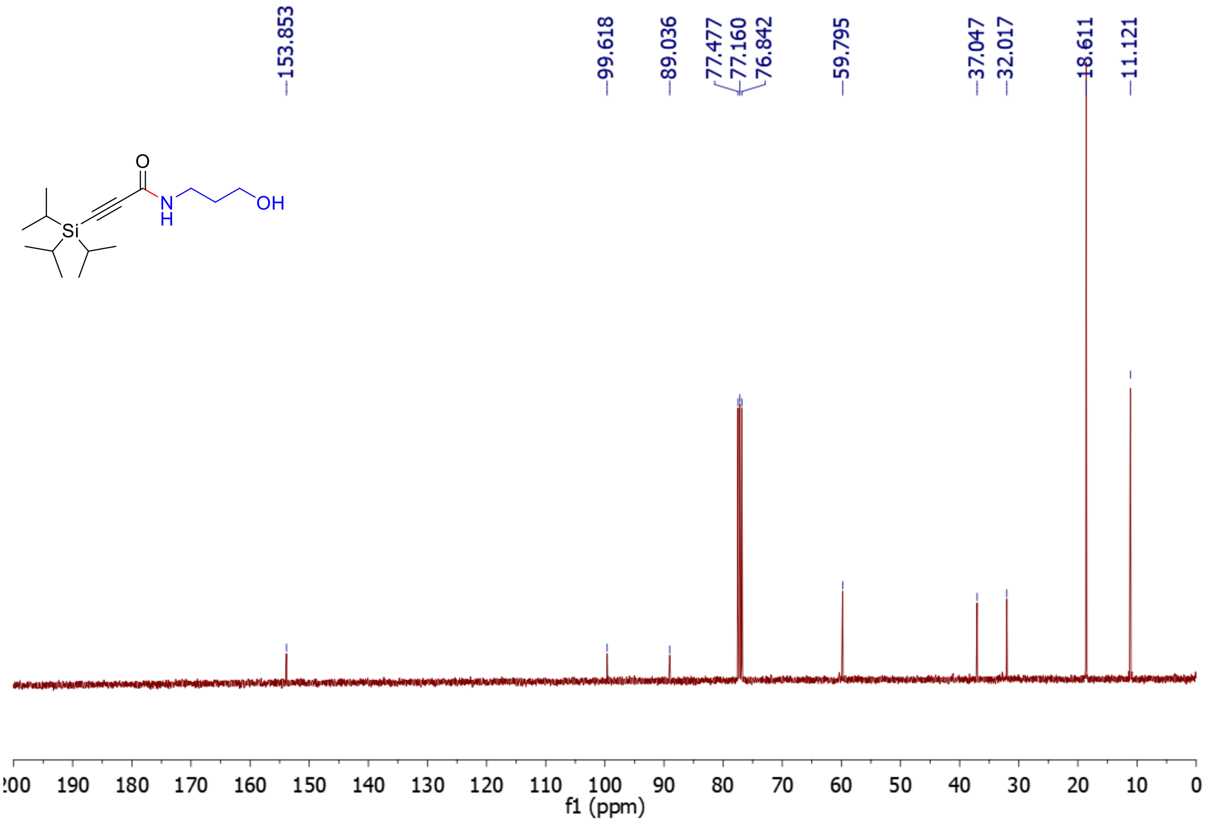

Fig. S92. ^{13}C NMR spectrum of N-(3-hydroxypropyl)-3-(triisopropylsilyl)propiolamide **(3y)** in $CDCl_3$.

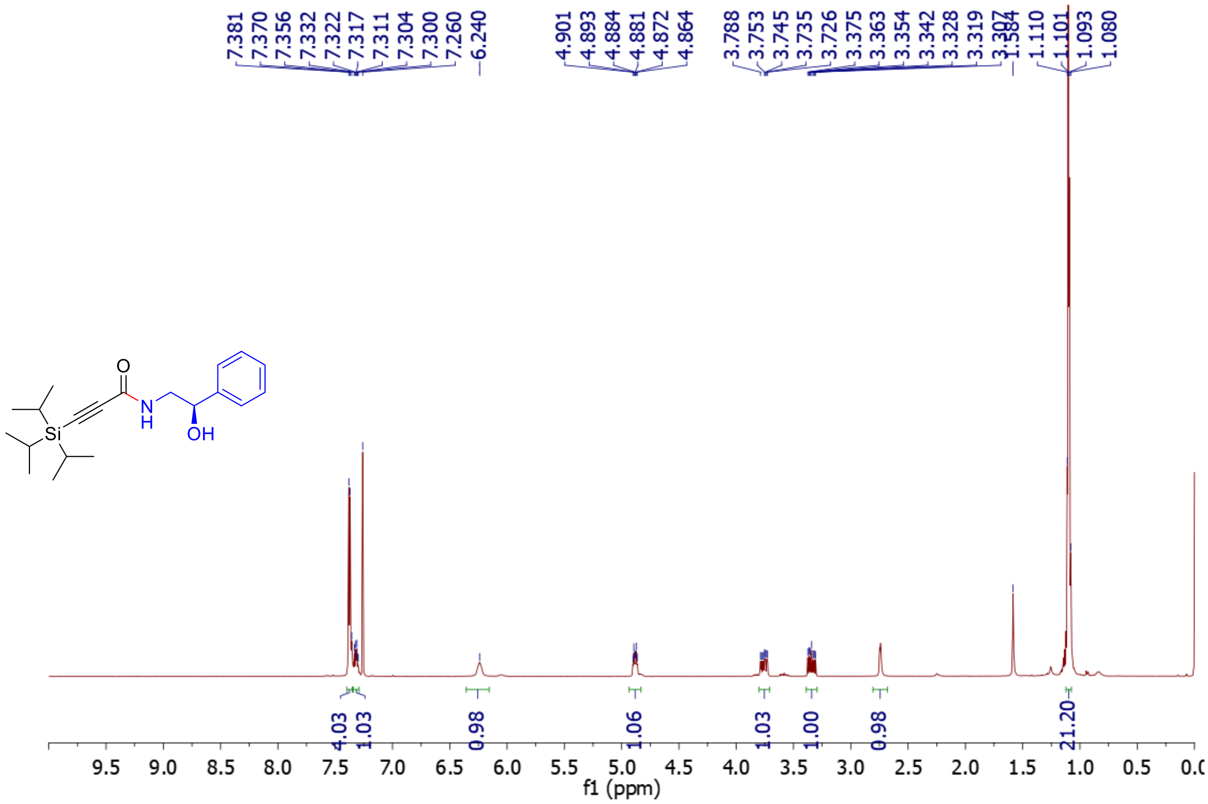

Fig. S93. ¹H NMR spectrum of (R)-N-(2-hydroxy-2-phenylethyl)-3-(triisopropylsilyl)propiolamide **(3z)** in $CDCl_3$.

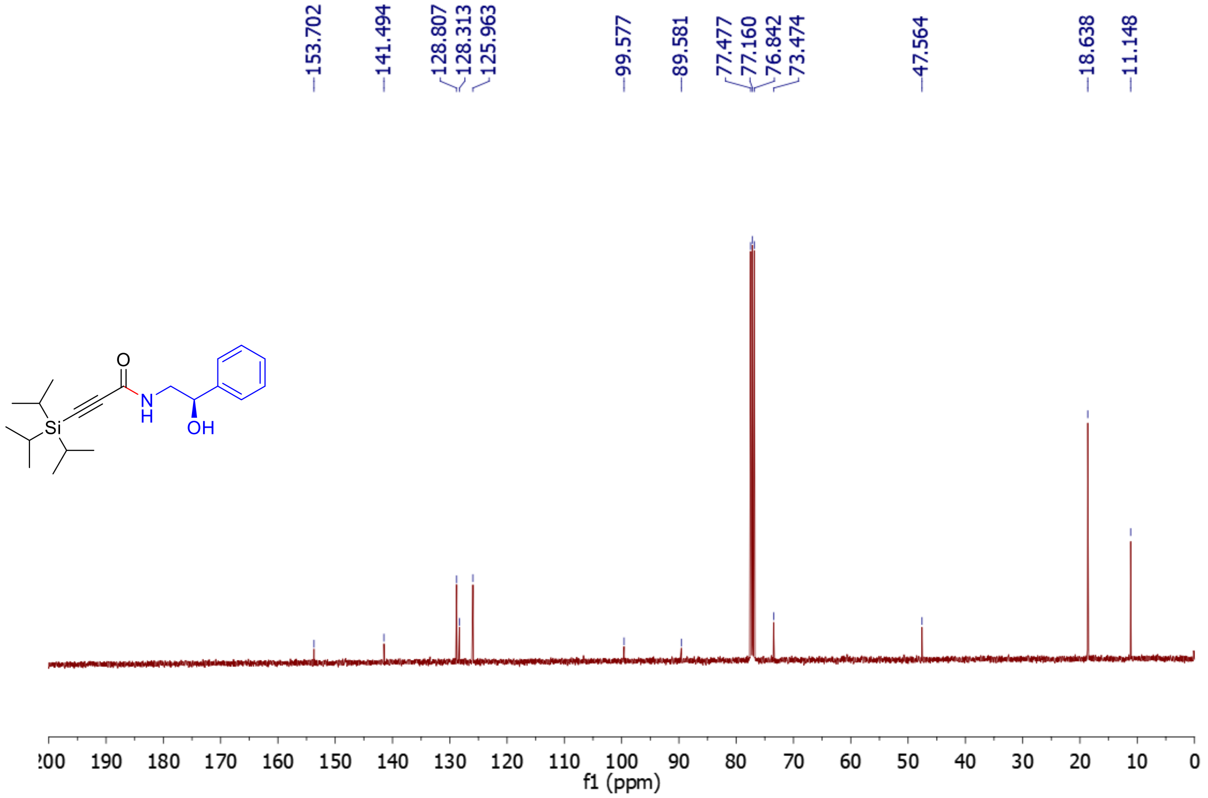

Fig. S94. ¹³C NMR spectrum of (R)-N-(2-hydroxy-2-phenylethyl)-3-(triisopropylsilyl)propiolamide **(3z)** in $CDCl_3$.

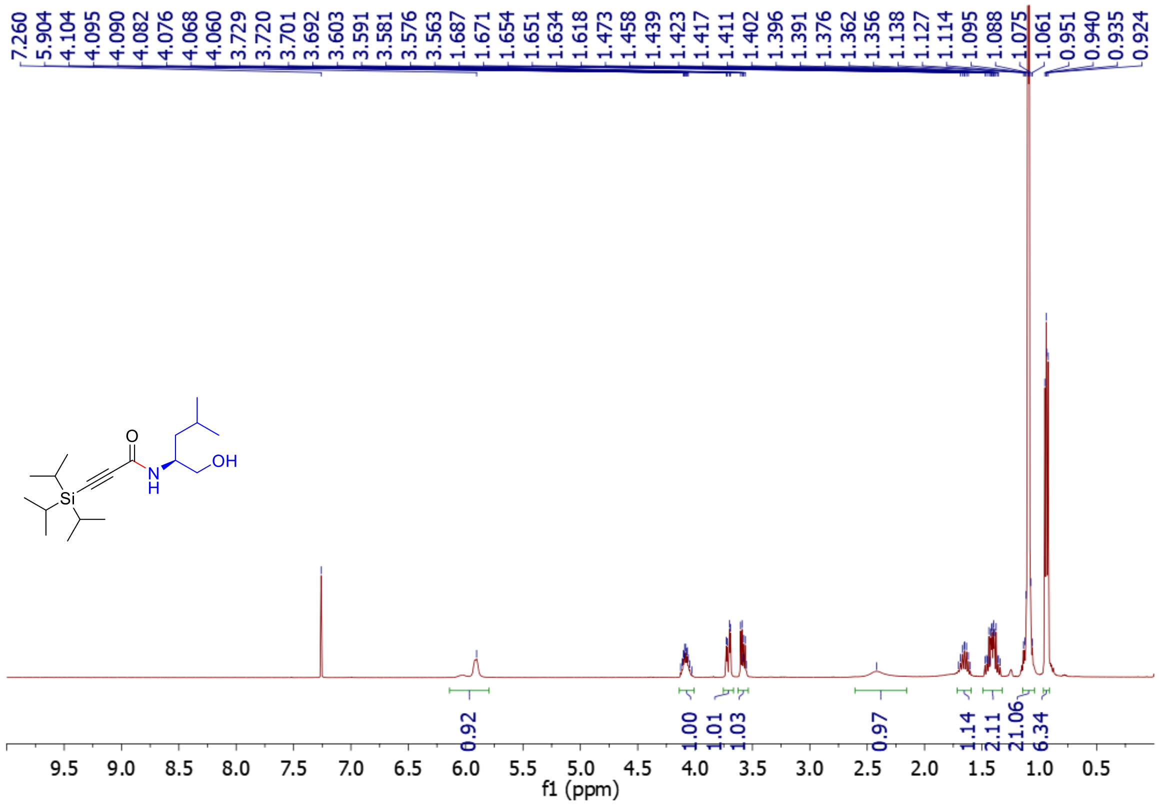

Fig. S95. ^{1}H NMR spectrum of (S)-N-(1-hydroxy-4-methylpentan-2-yl)-3-(triisopropylsilyl)propiolamide **(3ab)** in $CDCl_3$.

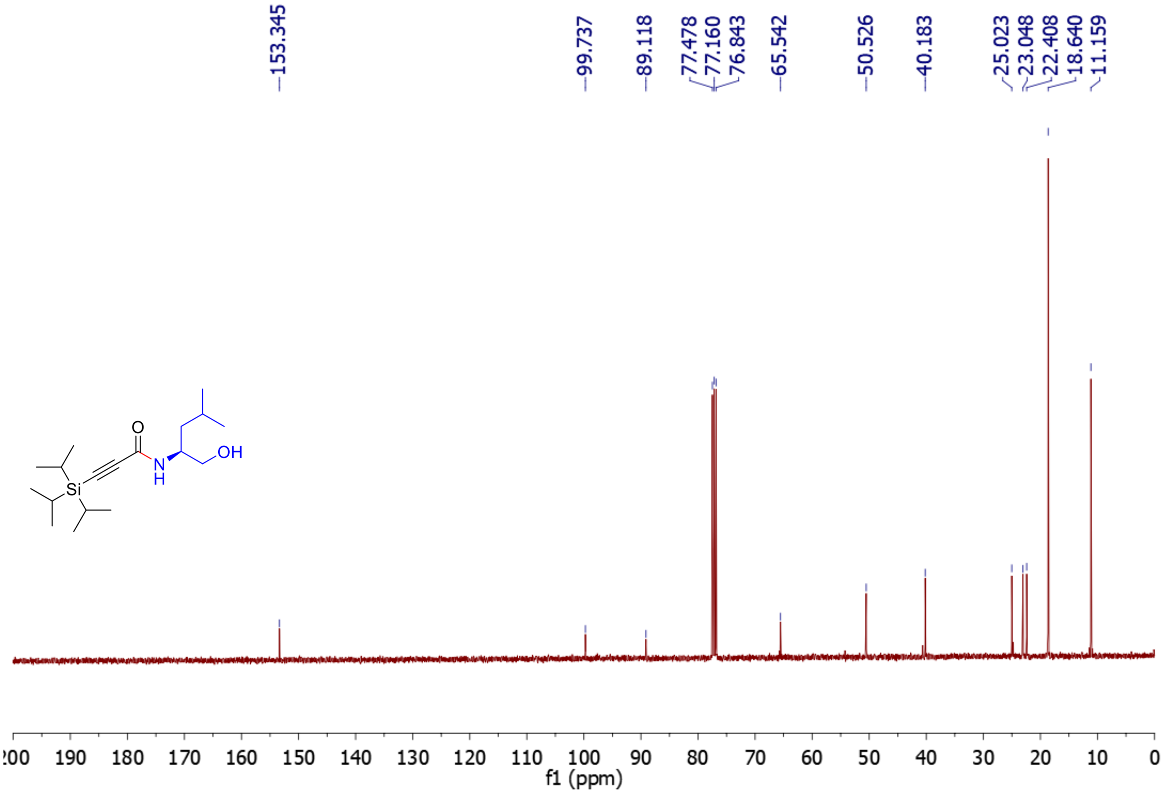

Fig. S96. ^{13}C NMR spectrum of (S)-N-(1-hydroxy-4-methylpentan-2-yl)-3-(triisopropylsilyl)propiolamide **(3ab)** in $CDCl_3$.

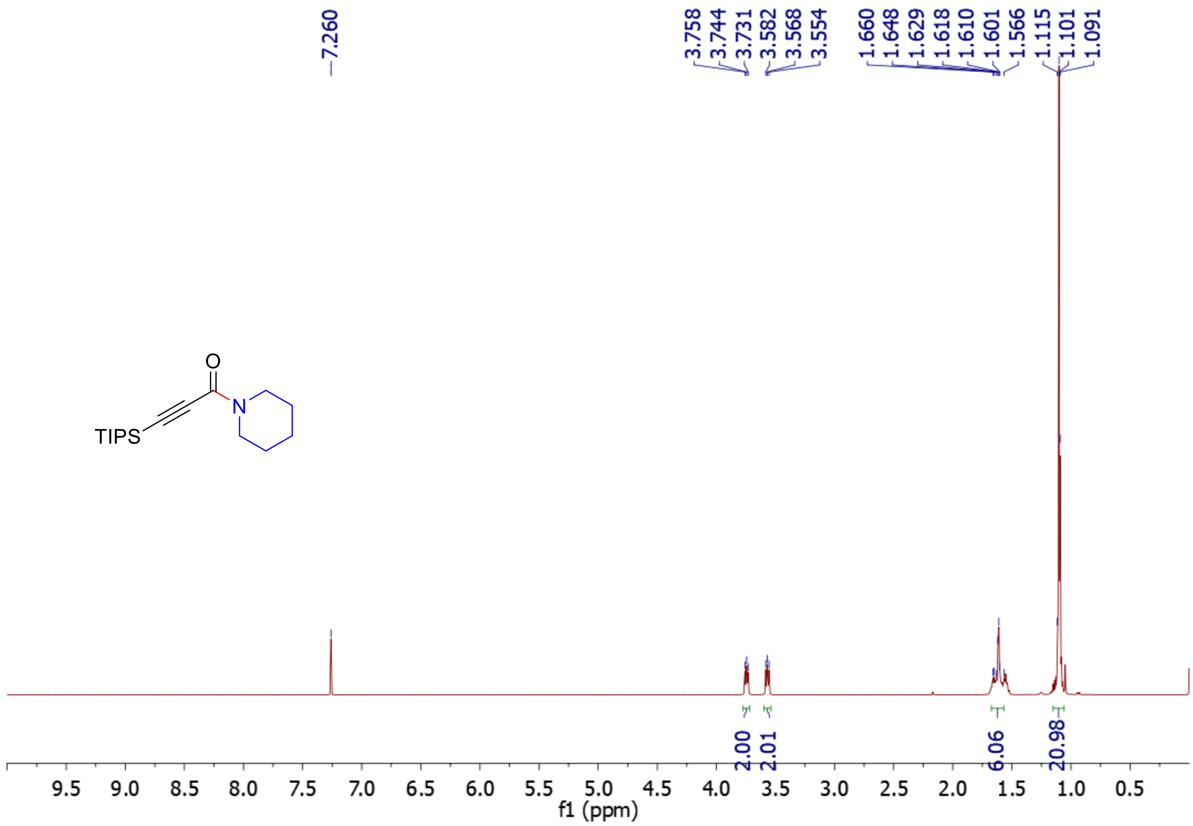

Fig. S97. ^{1}H NMR spectrum of 1-(piperidin-1-yl)-3-(triisopropylsilyl)prop-2-yn-1-one **(3ac)** in $CDCl_3$.

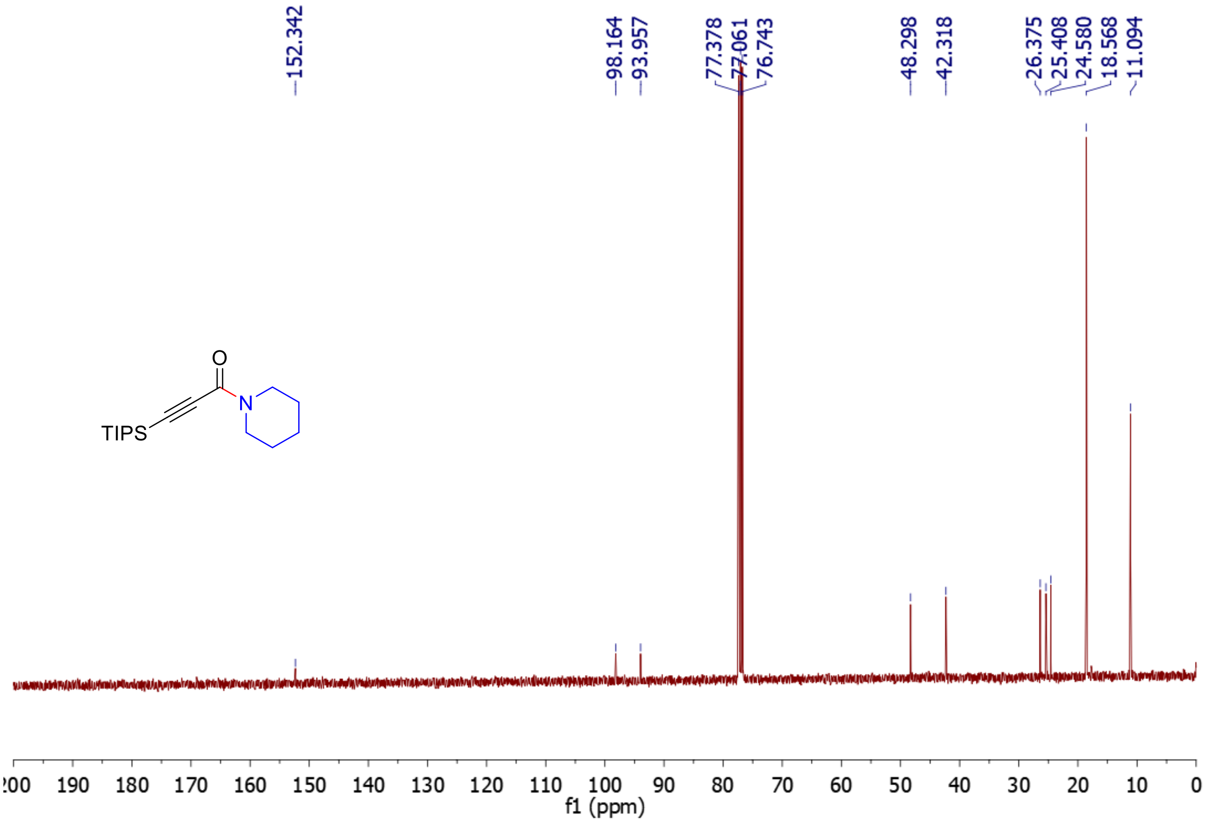

Fig. S98. ^{13}C NMR spectrum of 1-(piperidin-1-yl)-3-(triisopropylsilyl)prop-2-yn-1-one **(3ac)** in $CDCl_3$.

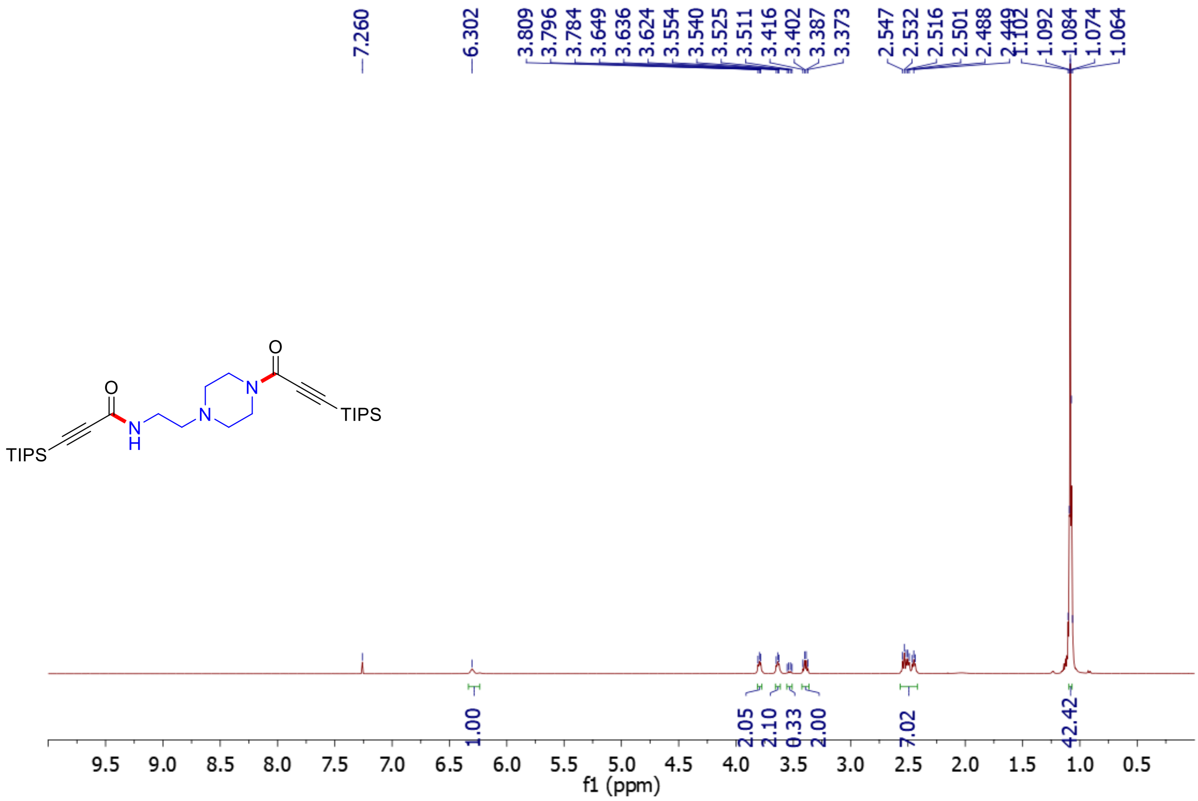

Fig. S99. ^{1}H NMR spectrum of 3-(triisopropylsilyl)-N-(2-(4-(3-(triisopropylsilyl)propioloyl)piperazin-1-yl)ethyl)propiolamide **(3ad)** in $CDCl_3$.

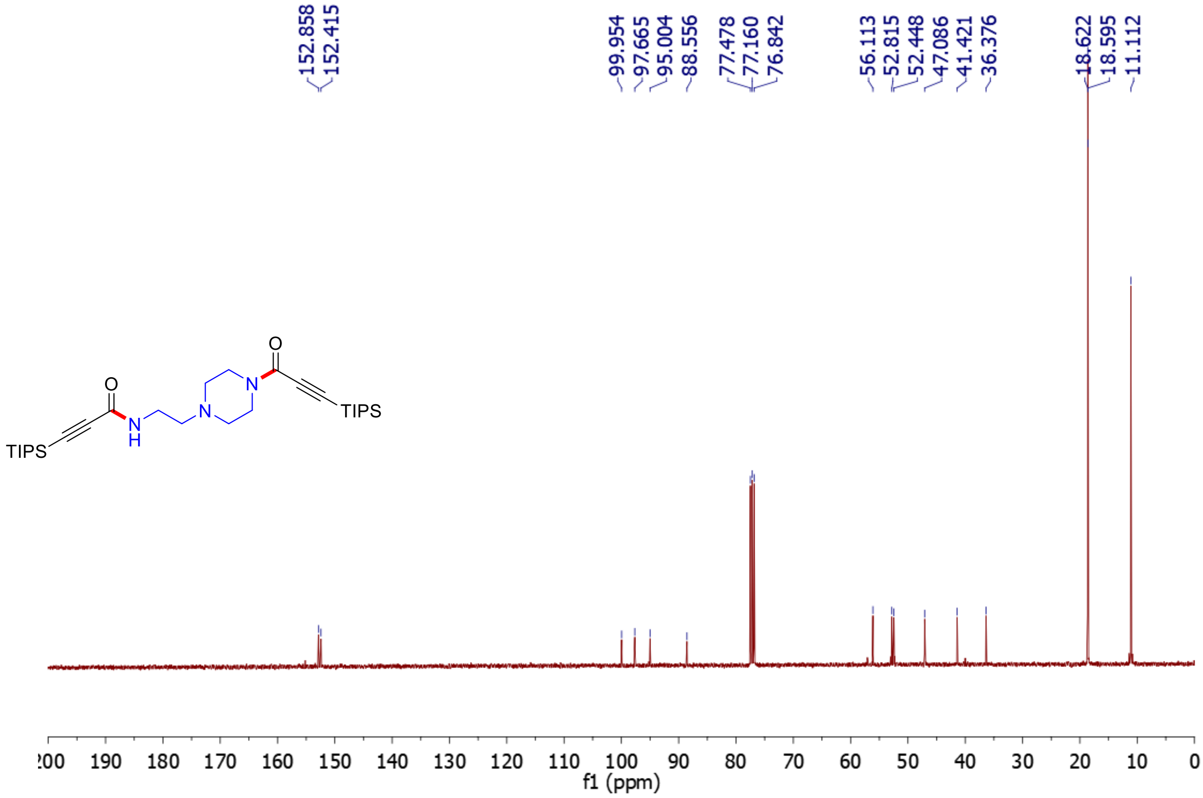

Fig. S100. ^{13}C NMR spectrum of 3-(triisopropylsilyl)-N-(2-(4-(3-(triisopropylsilyl)propioloyl)piperazin-1-yl)ethyl)propiolamide **(3ad)** in $CDCl_3$.

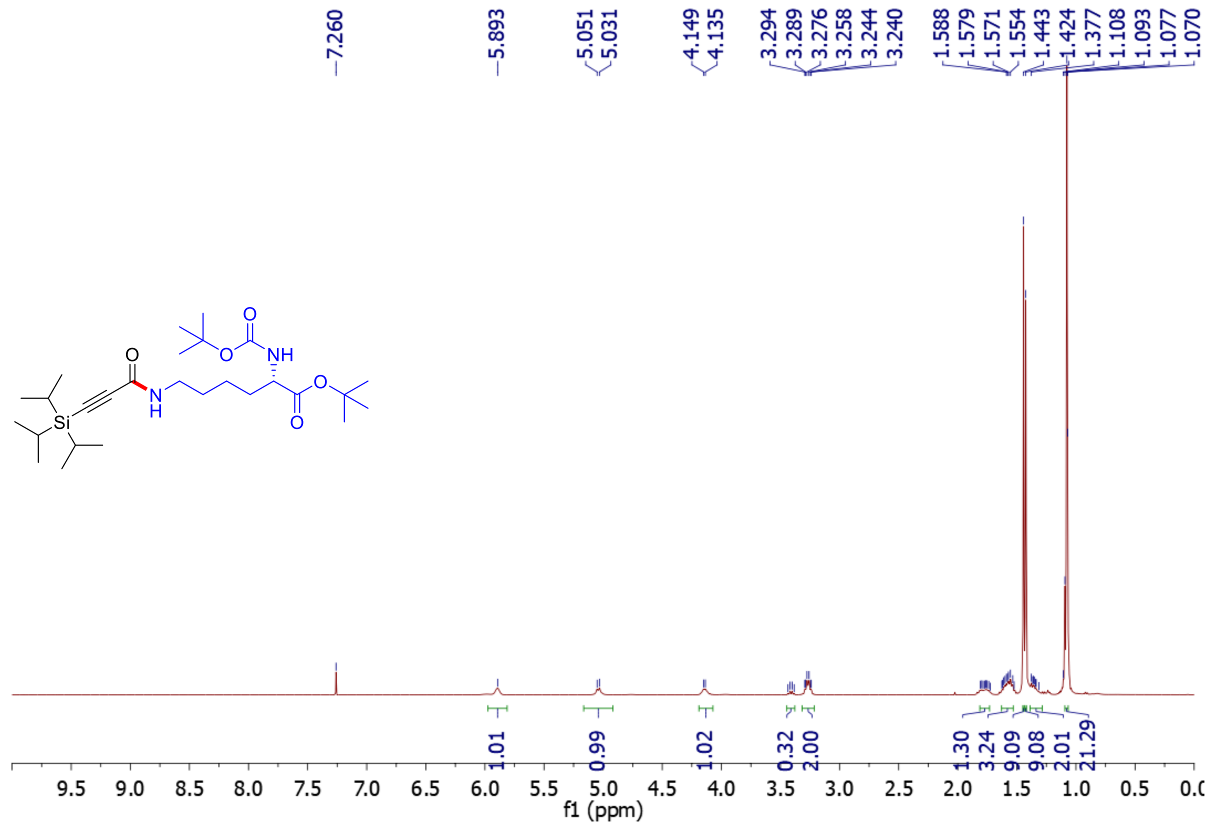

Fig. S101. ^{1}H NMR spectrum of tert-butyl *N*2-(tert-butoxycarbonyl)-*N*6-(3-(triisopropylsilyl)propioloyl)-L-lysinate **(3ae)** in $CDCl_3$.

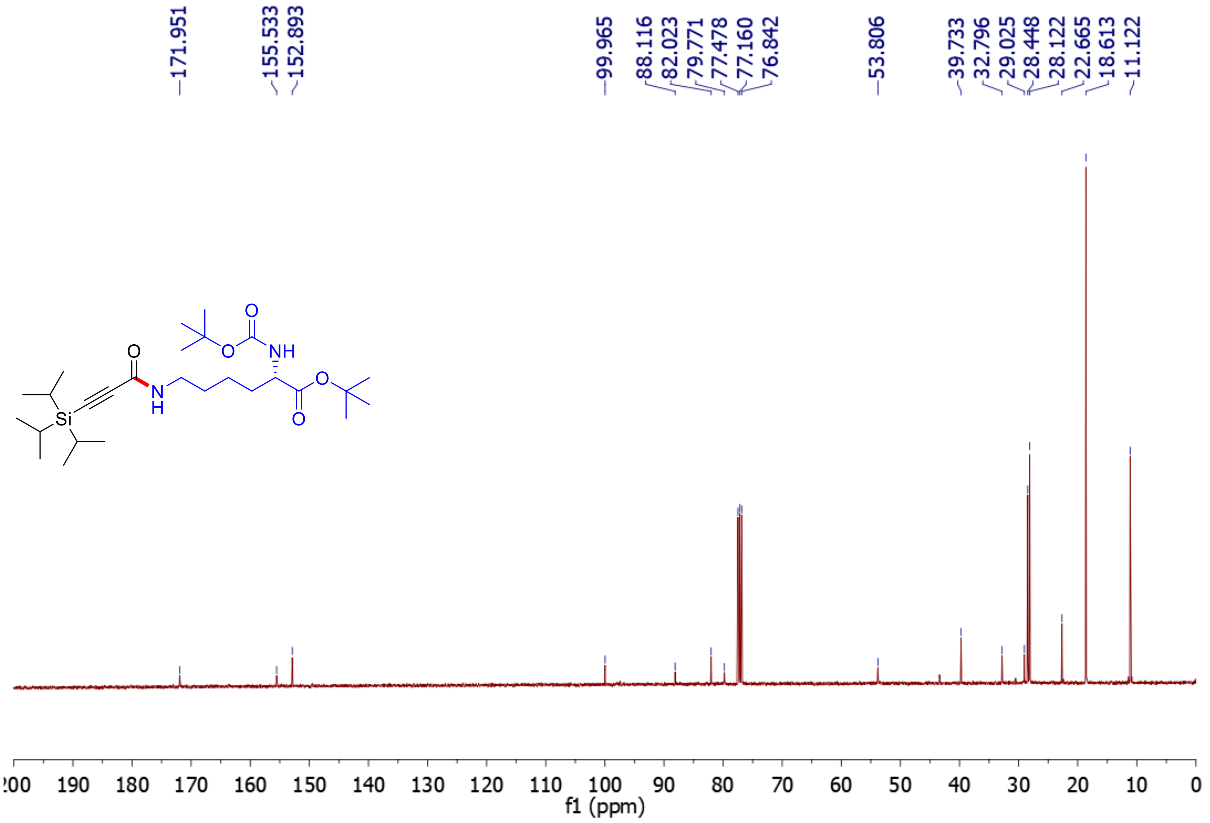

Fig. S102. ^{13}C NMR spectrum of tert-butyl *N*2-(tert-butoxycarbonyl)-*N*6-(3-(triisopropylsilyl)propioloyl)-L-lysinate **(3ae)** in $CDCl_3$.

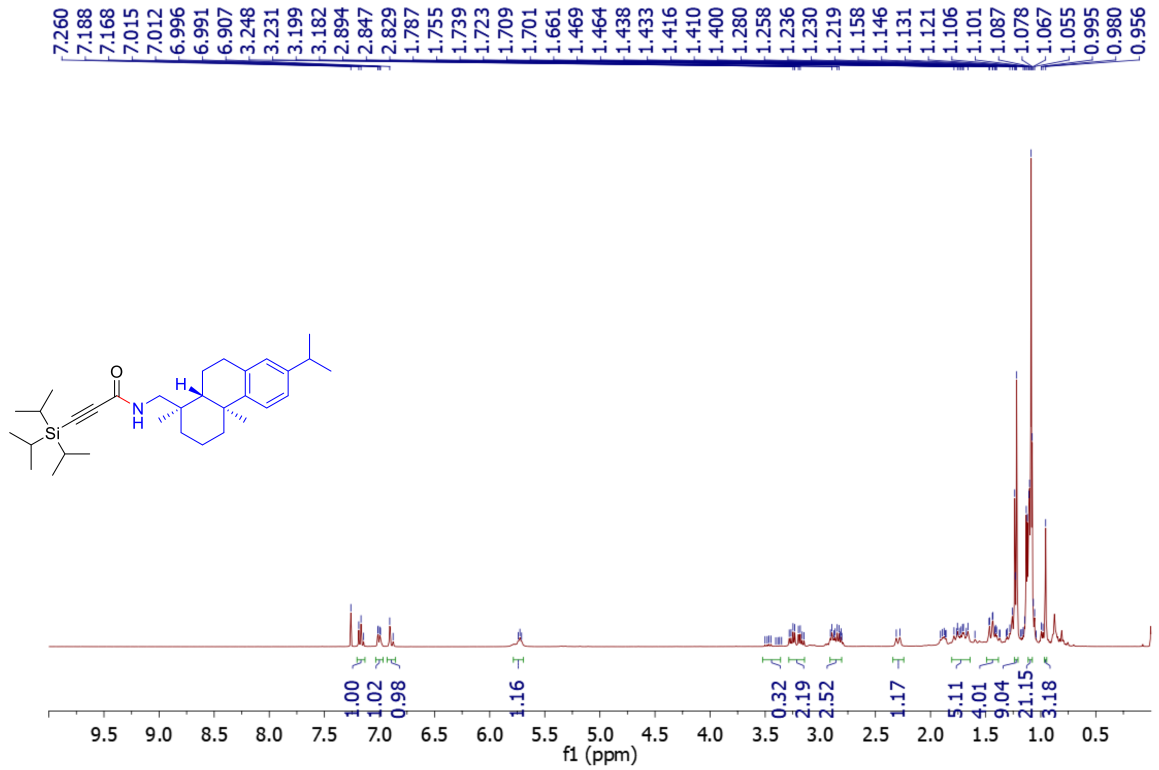

Fig. S103. ^{1}H NMR spectrum of N-(((1R,4aS,10aR)-7-isopropyl-1,4a-dimethyl-1,2,3,4,4a,9,10,10a-octahydrophenanthren-1-yl)methyl)-3-(triisopropylsilyl)propiolamide **(3af)** in $CDCl_3$.

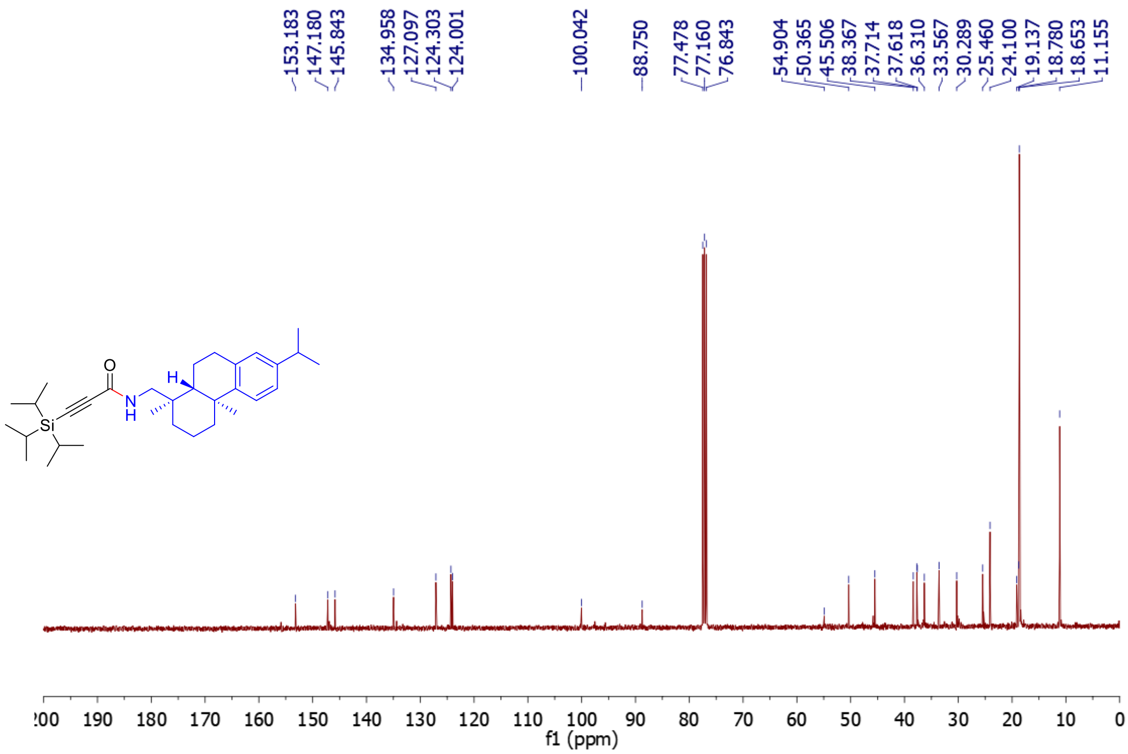

Fig. S104. ^{13}C NMR spectrum of N-(((1R,4aS,10aR)-7-isopropyl-1,4a-dimethyl-1,2,3,4,4a,9,10,10a-octahydrophenanthren-1-yl)methyl)-3-(triisopropylsilyl)propiolamide **(3af)** in $CDCl_3$.

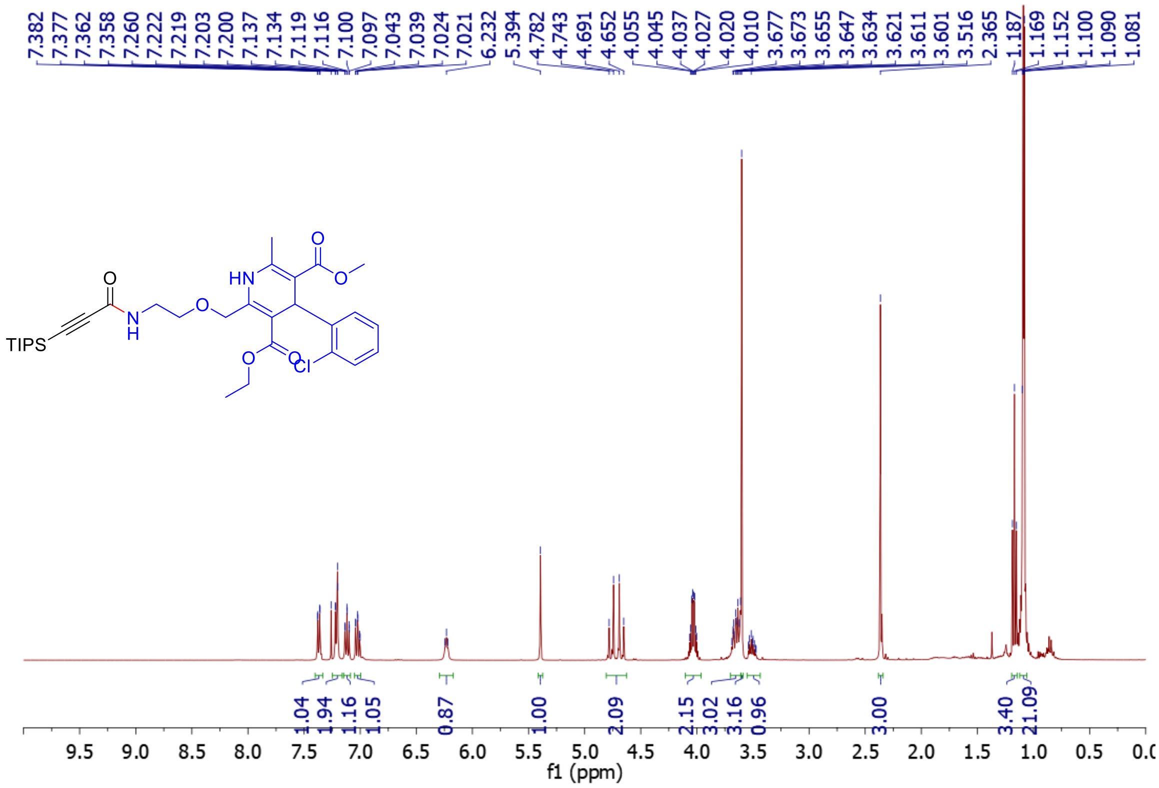

Fig. S105. ^{1}H NMR spectrum of 3-ethyl 5-methyl 4-(2-chlorophenyl)-6-methyl-2-((2-(3-(triisopropylsilyl)propiolamido)ethoxy)methyl)-1,4-dihydropyridine-3,5-dicarboxylate **(3ag)** in $CDCl_3$.

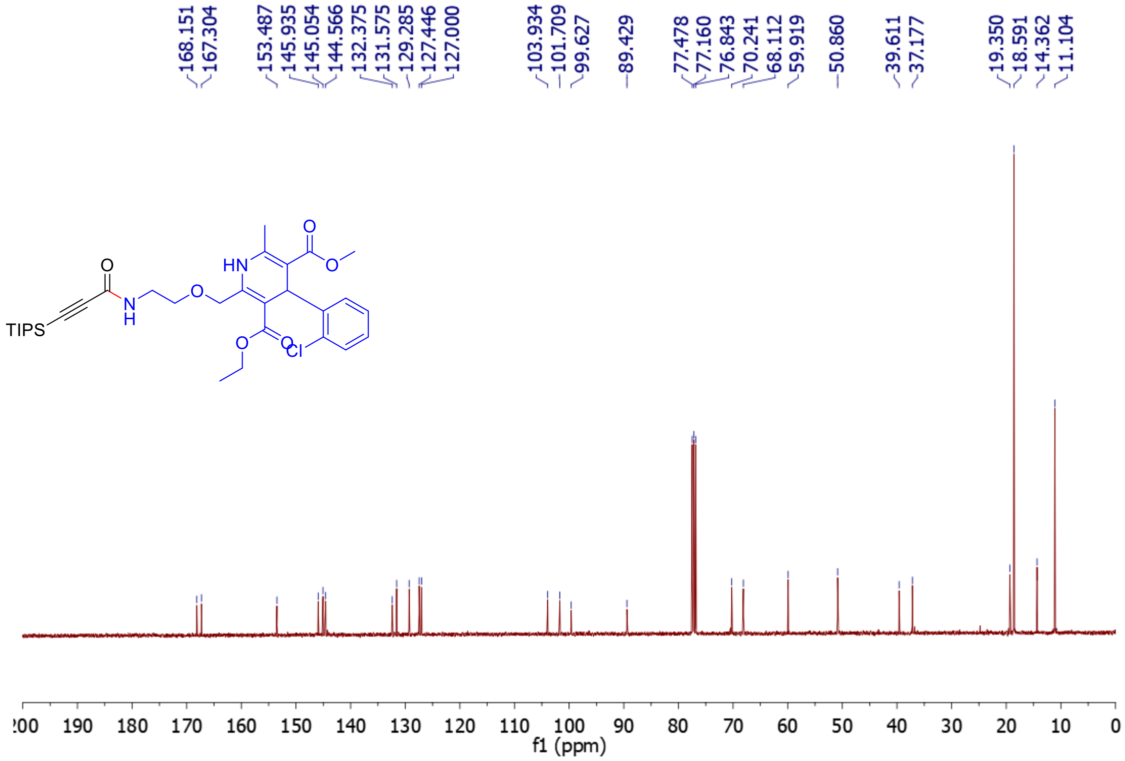

Fig. S106. ^{13}C NMR spectrum of 3-ethyl 5-methyl 4-(2-chlorophenyl)-6-methyl-2-((2-(3-(triisopropylsilyl)propiolamido)ethoxy)methyl)-1,4-dihydropyridine-3,5-dicarboxylate **(3ag)** in $CDCl_3$.

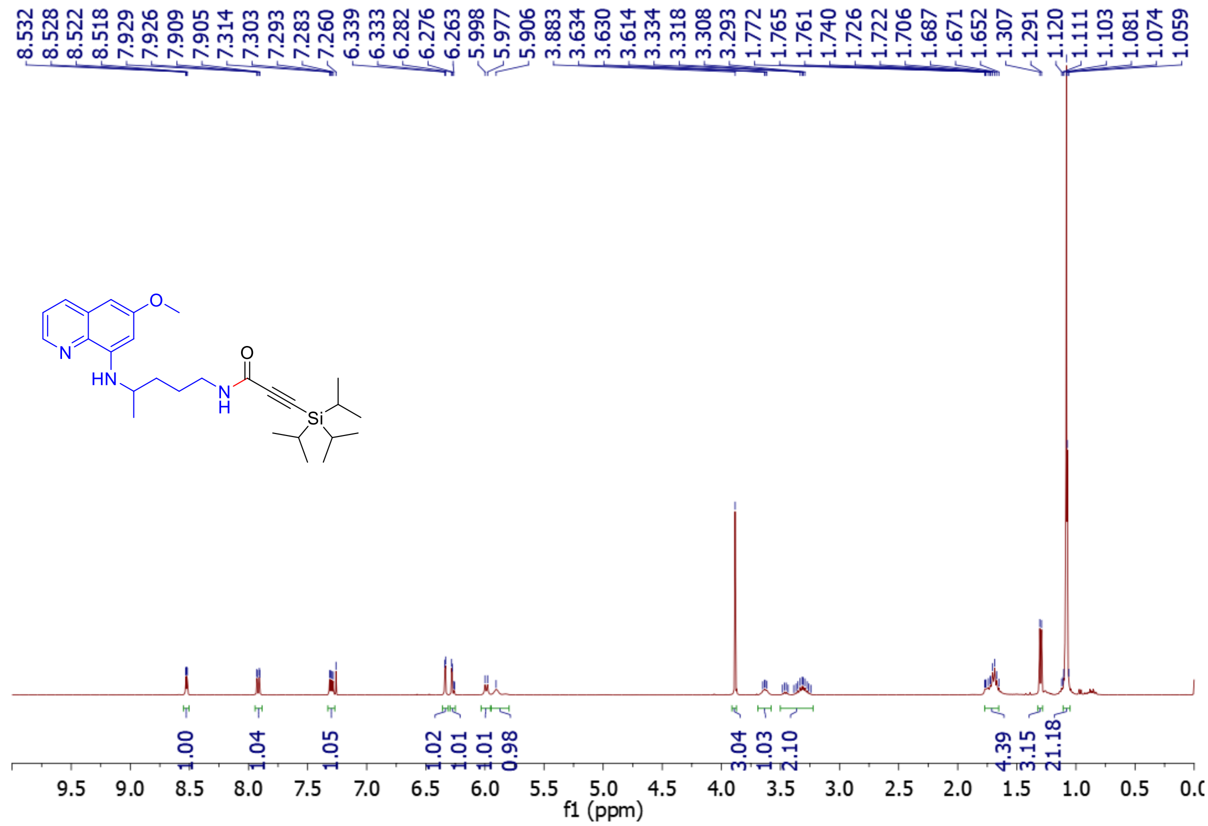

Fig. S107. ¹H NMR spectrum of N-(4-((6-methoxyquinolin-8-yl)amino)pentyl)-3-(triisopropylsilyl)propiolamide **(3ah)** in $CDCl_3$.

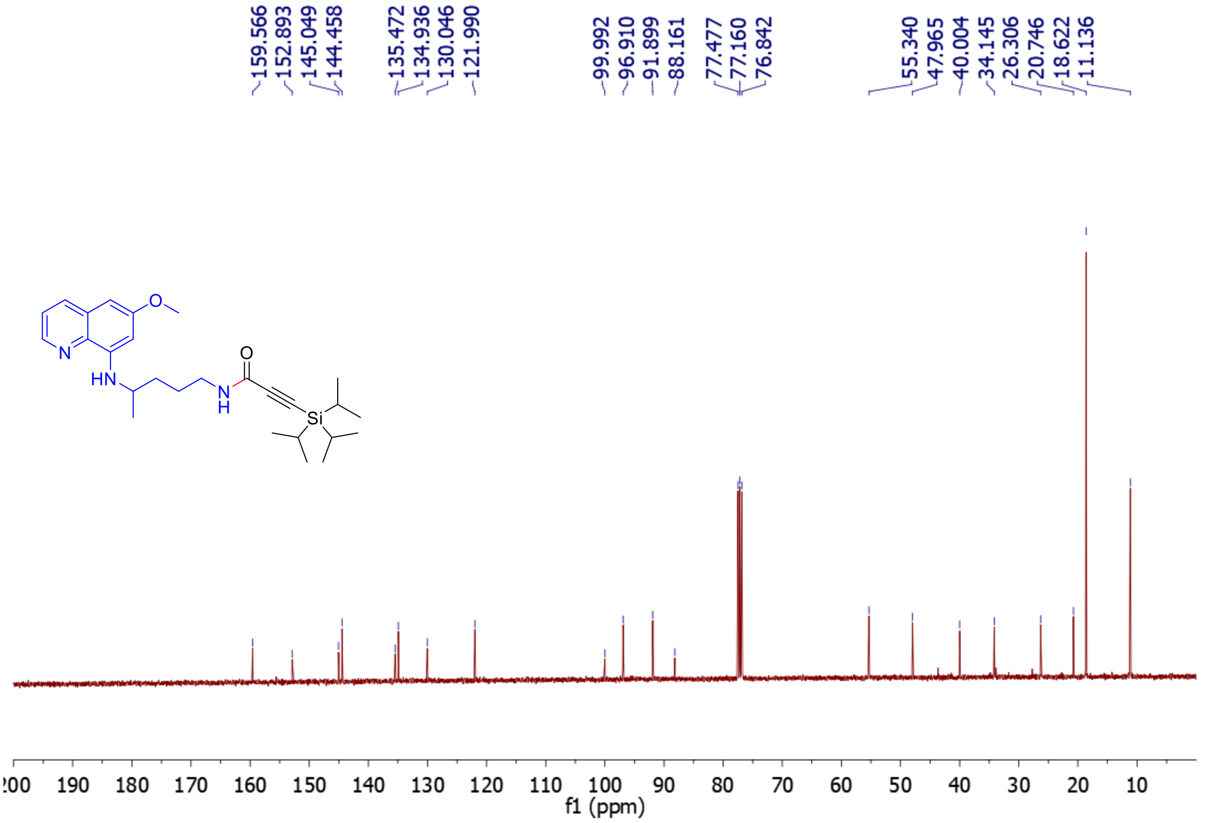

Fig. S108. ¹³C NMR spectrum of N-(4-((6-methoxyquinolin-8-yl)amino)pentyl)-3-(triisopropylsilyl)propiolamide **(3ah)** in $CDCl_3$.

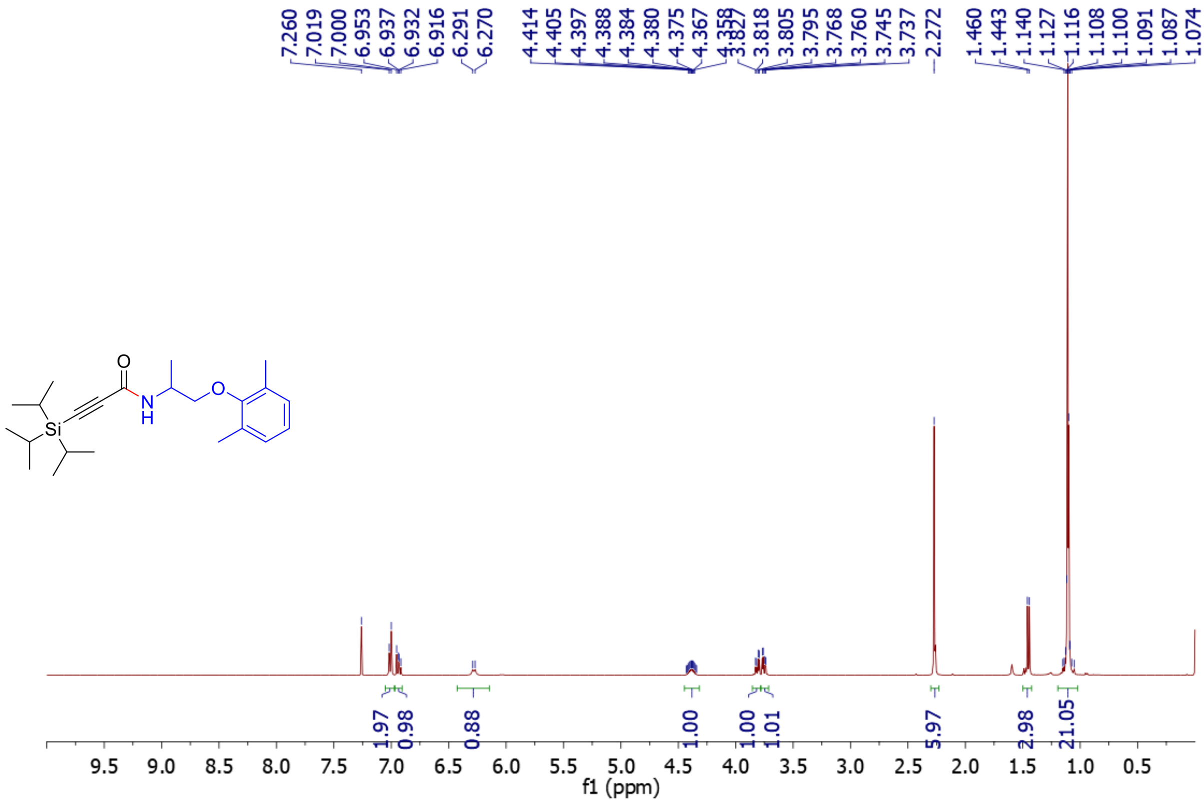

Fig. S109. ^{1}H NMR spectrum of N-(1-(2,6-dimethylphenoxy)propan-2-yl)-3-(triisopropylsilyl)propiolamide **(3ai)** in $CDCl_3$.

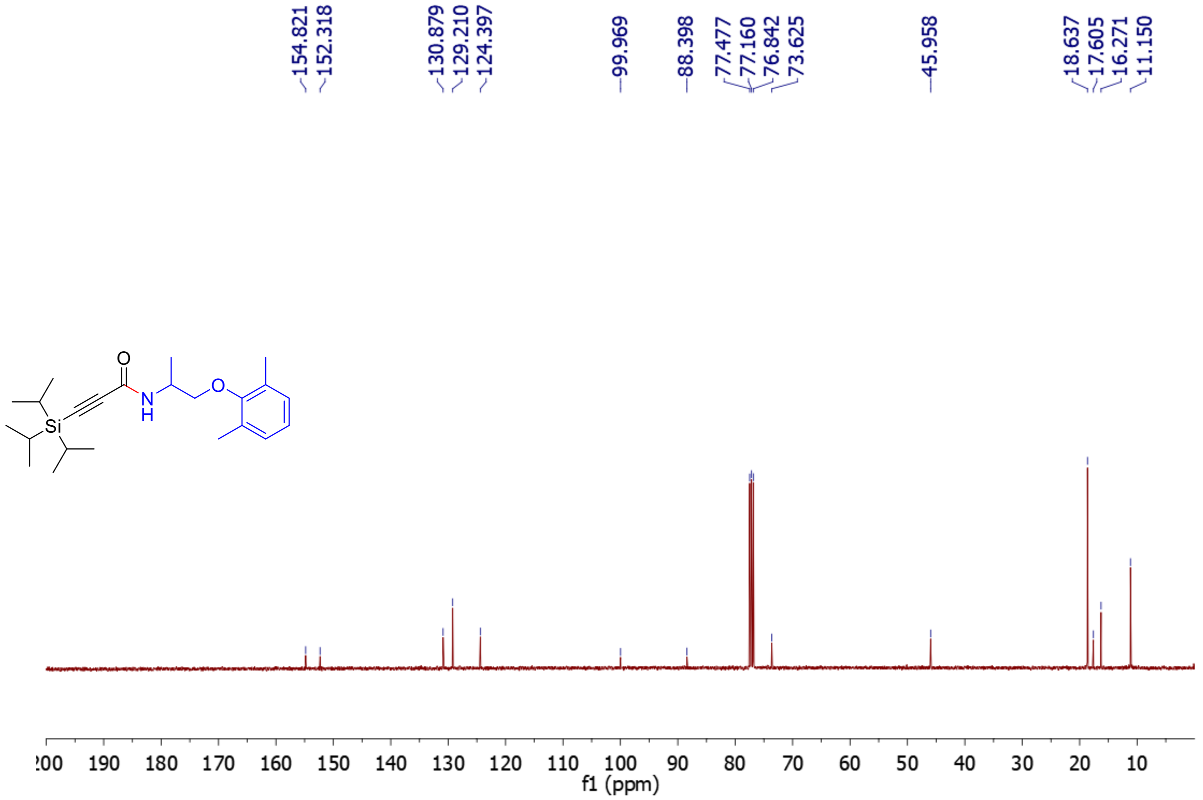

Fig. S110. ^{13}C NMR spectrum of N-(1-(2,6-dimethylphenoxy)propan-2-yl)-3-(triisopropylsilyl)propiolamide **(3ai)** in $CDCl_3$.

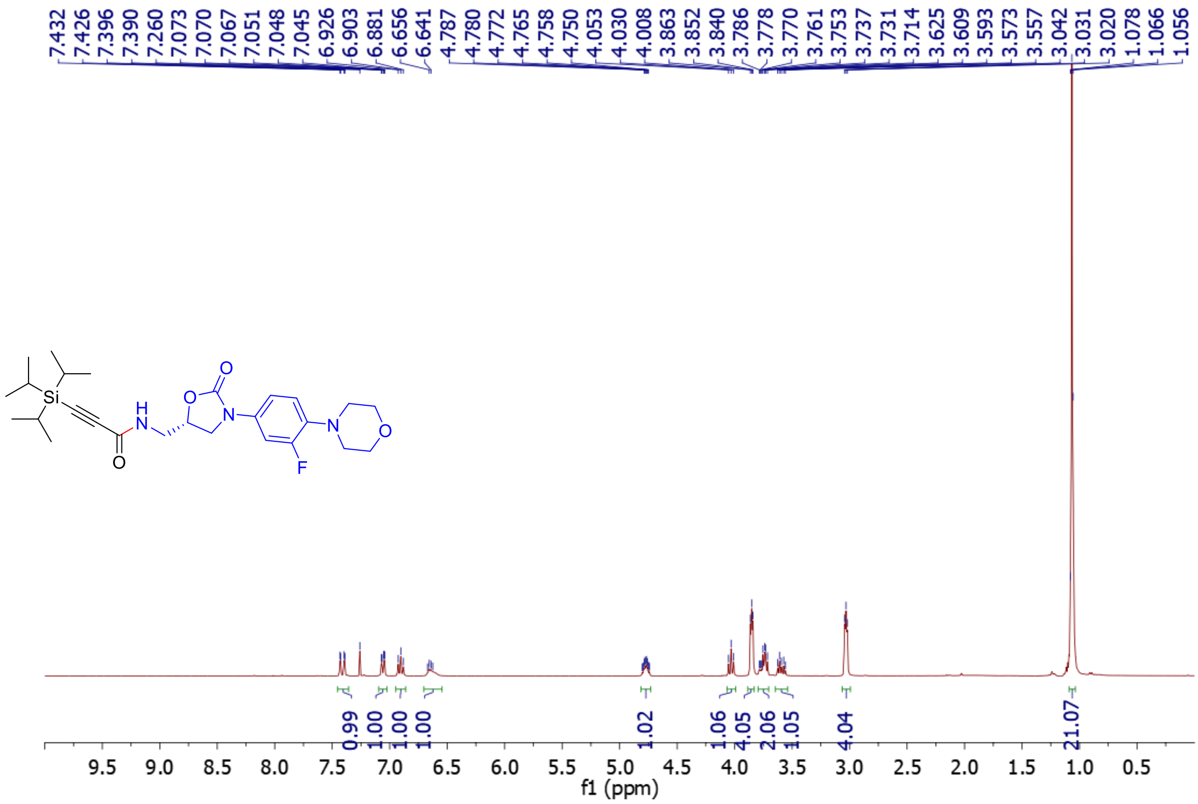

Fig. S111. ^{1}H NMR spectrum of (S)-N-((3-(3-fluoro-4-morpholinophenyl)-2-oxooxazolidin-5-yl)methyl)-3-(triisopropylsilyl)propiolamide **(3aj)** in $CDCl_3$.

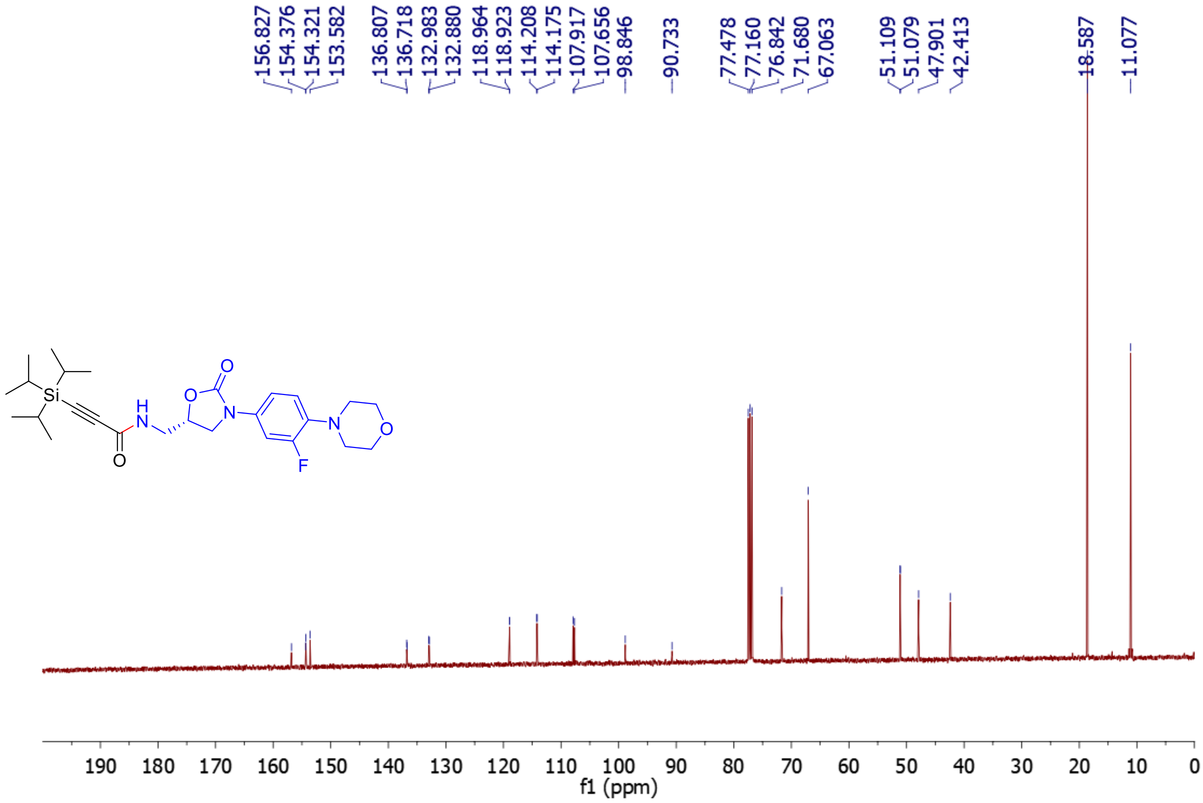

Fig. S112. ^{13}C NMR spectrum of (S)-N-((3-(3-fluoro-4-morpholinophenyl)-2-oxooxazolidin-5-yl)methyl)-3-(triisopropylsilyl)propiolamide **(3aj)** in $CDCl_3$.

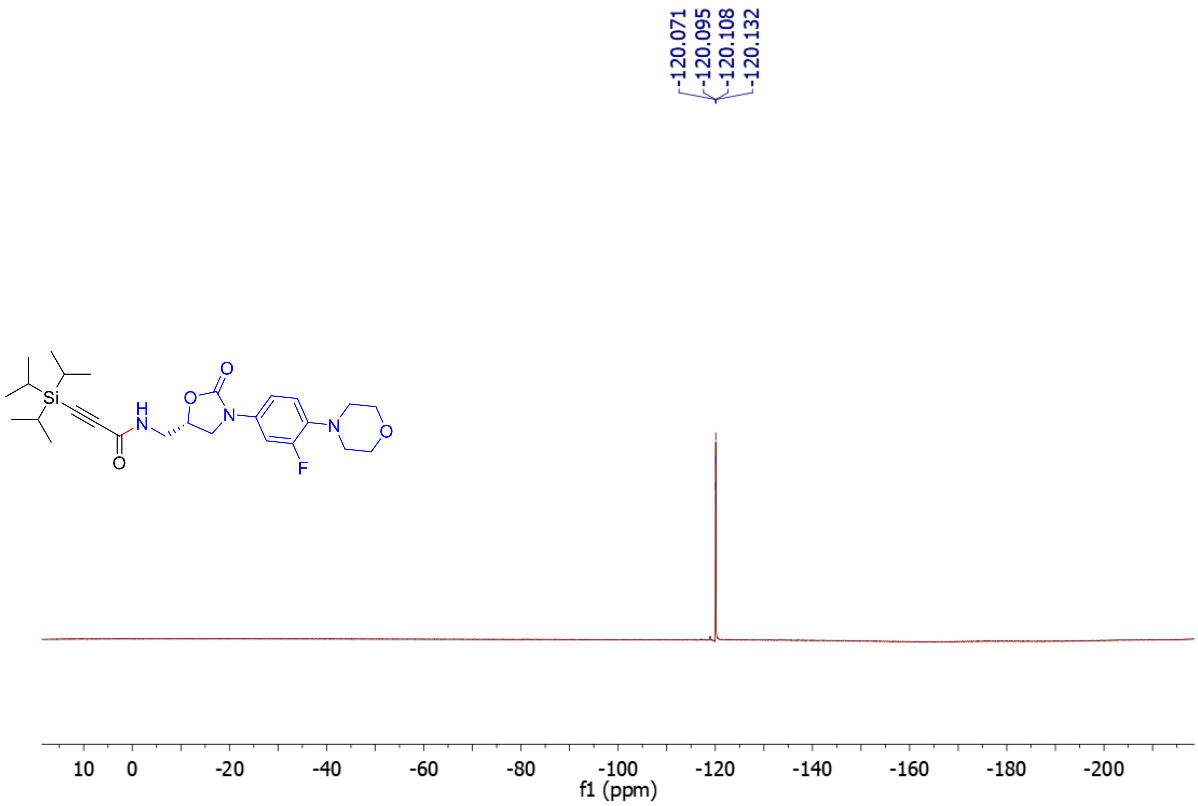

Fig. S113. ^{19}F NMR spectrum of (S)-N-((3-(3-fluoro-4-morpholinophenyl)-2-oxooxazolidin-5-yl)methyl)-3-(triisopropylsilyl)propiolamide **(3aj)** in $CDCl_3$.

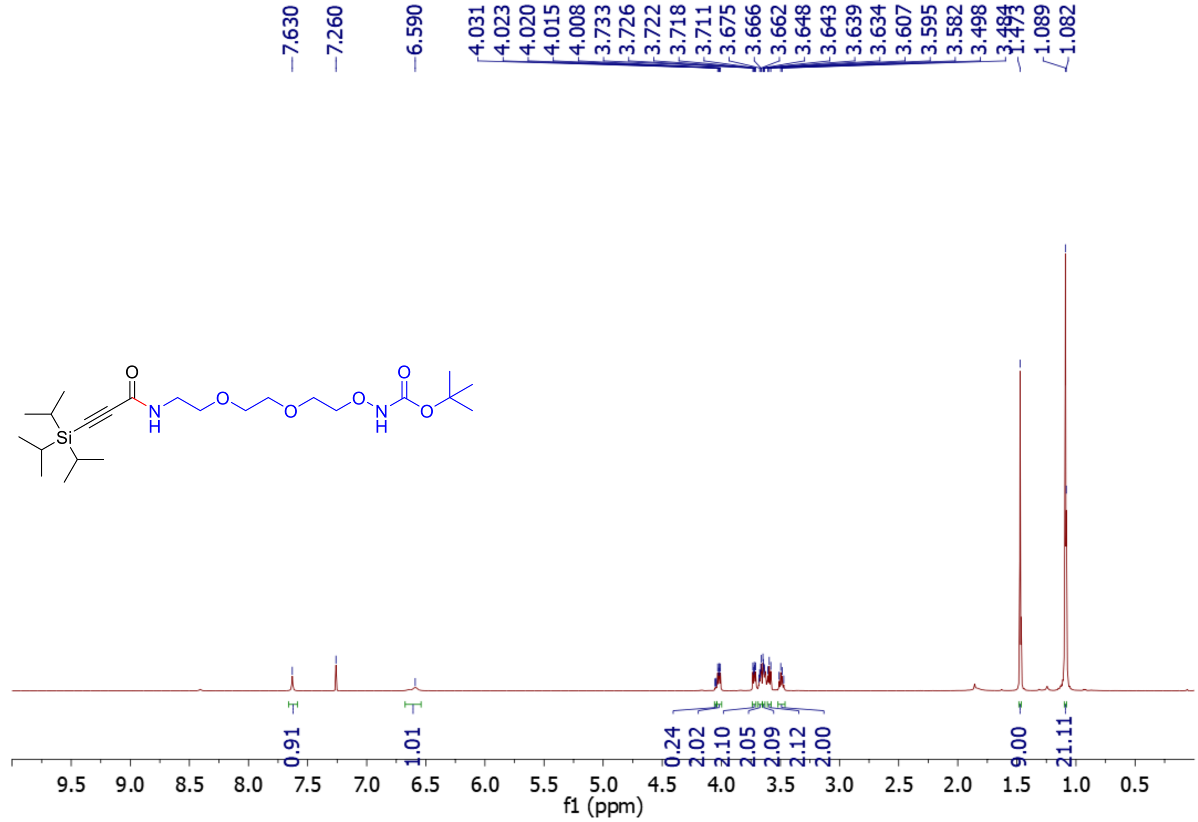

Fig. S114. ^{1}H NMR spectrum of tert-butyl ((13,13-diisopropyl-14-methyl-10-oxo-3,6-dioxa-9-aza-13-silapentadec-11-yn-1-yl)oxy)carbamate **(3ak)** in $CDCl_3$.

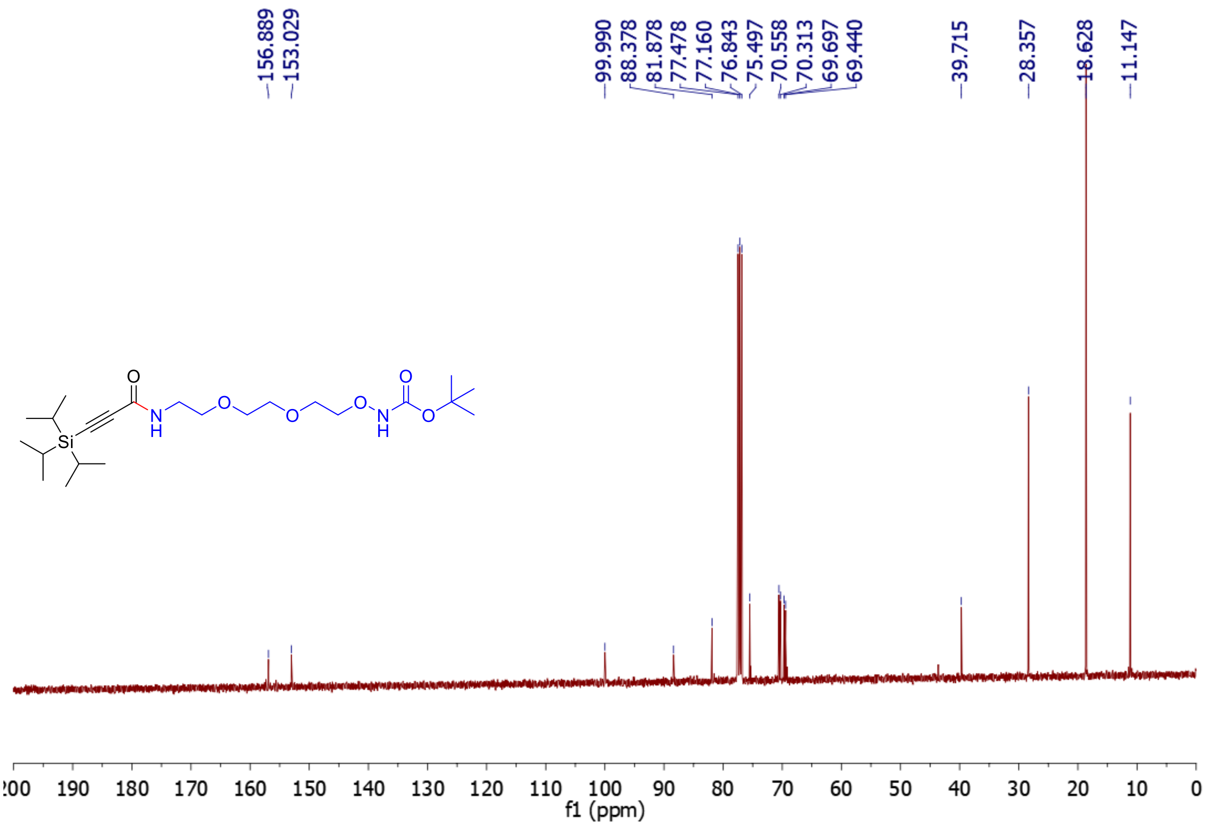

Fig. S115. ^{13}C NMR spectrum of tert-butyl ((13,13-diisopropyl-14-methyl-10-oxo-3,6-dioxa-9-aza-13-silapentadec-11-yn-1-yl)oxy)carbamate **(3ak)** in $CDCl_3$.

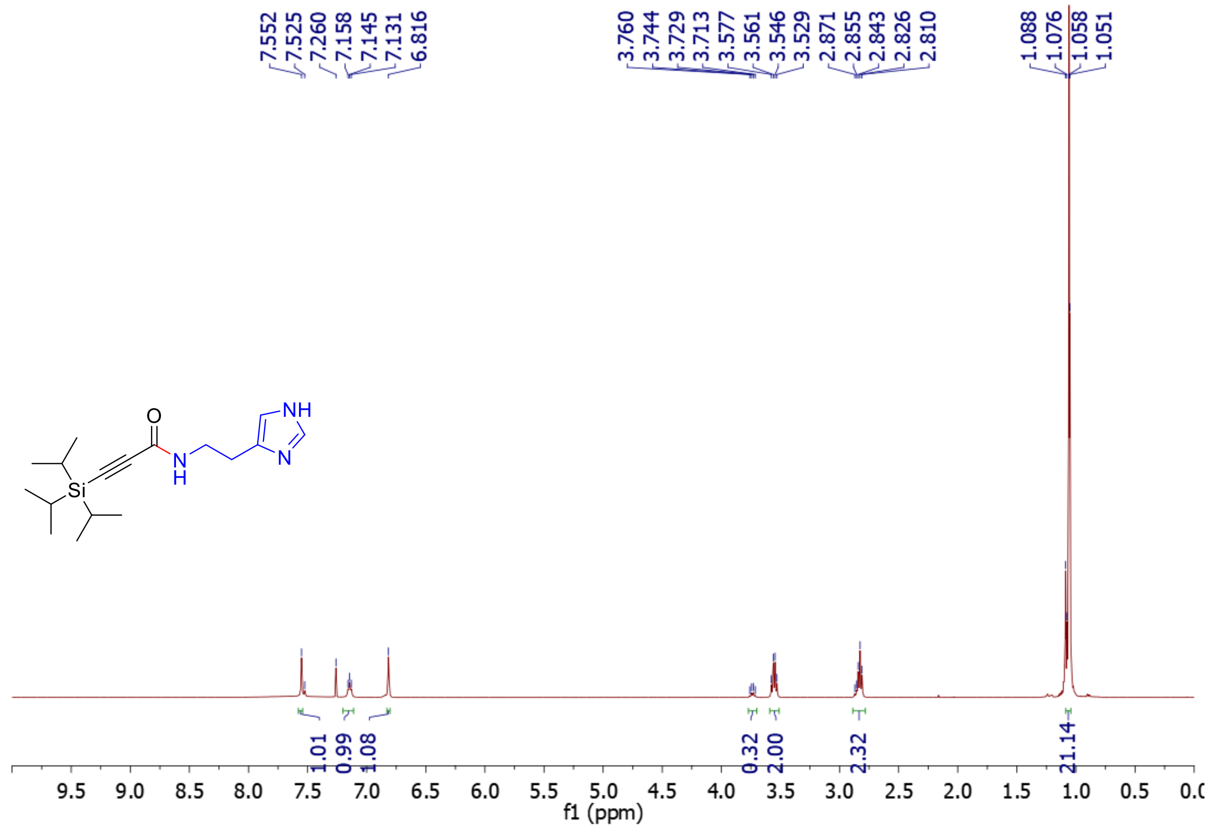

Fig. S116. ^{1}H NMR spectrum of N-(2-(1H-imidazol-5-yl)ethyl)-3-(triisopropylsilyl)propiolamide **(3al)** in $CDCl_3$.

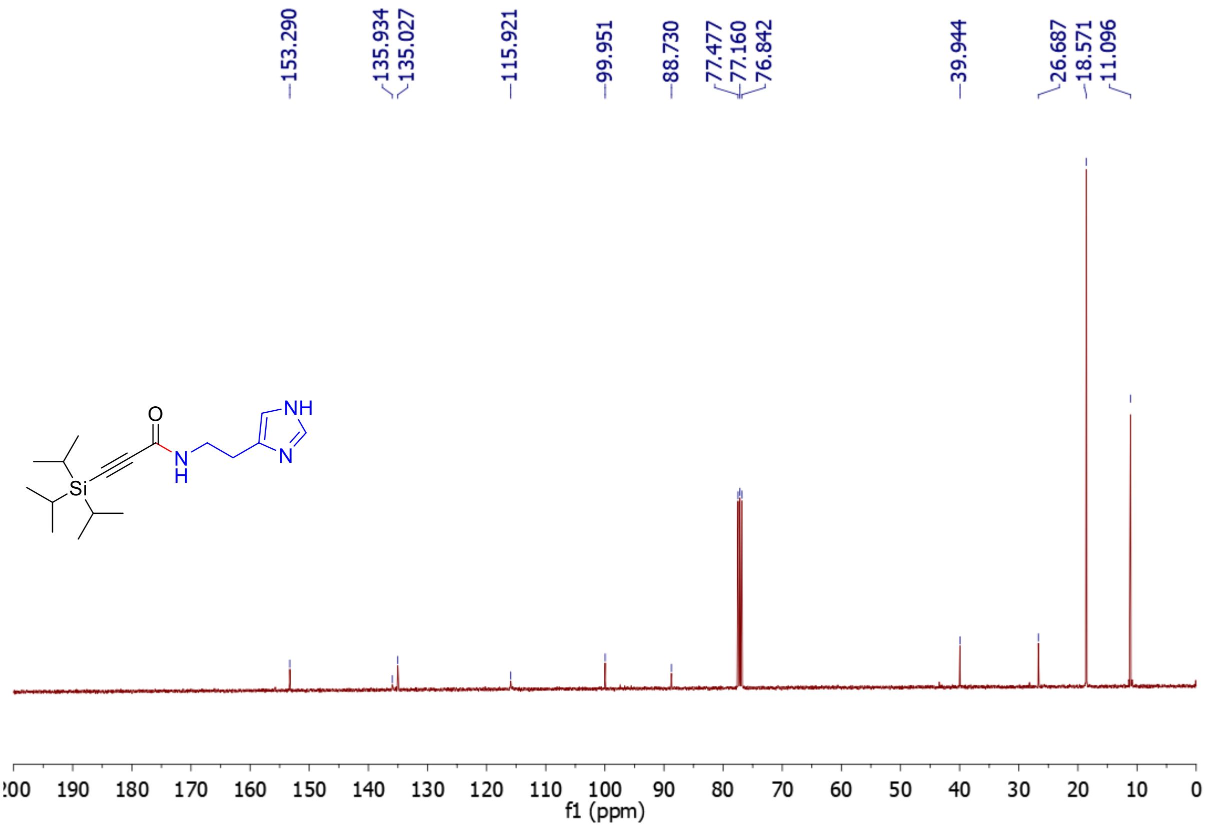

Fig. S117. ^{13}C NMR spectrum of N-(2-(1H-imidazol-5-yl)ethyl)-3-(triisopropylsilyl)propiolamide **(3al)** in $CDCl_3$.

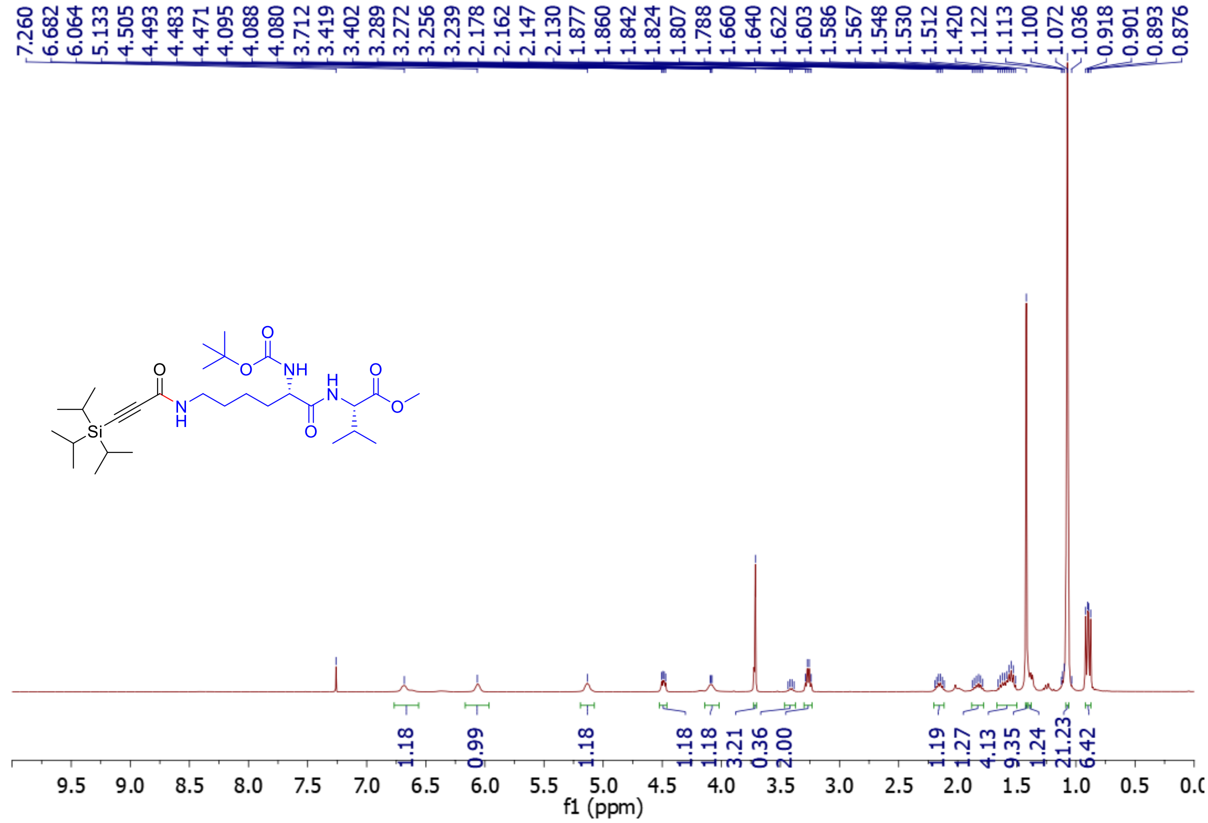

Fig. S118. ^{1}H NMR spectrum of methyl N2-(tert-butoxycarbonyl)-N6-(3-(triisopropylsilyl)propioloyl)-*L*-lysyl-*L*-valinate **(3am)** in $CDCl_3$.

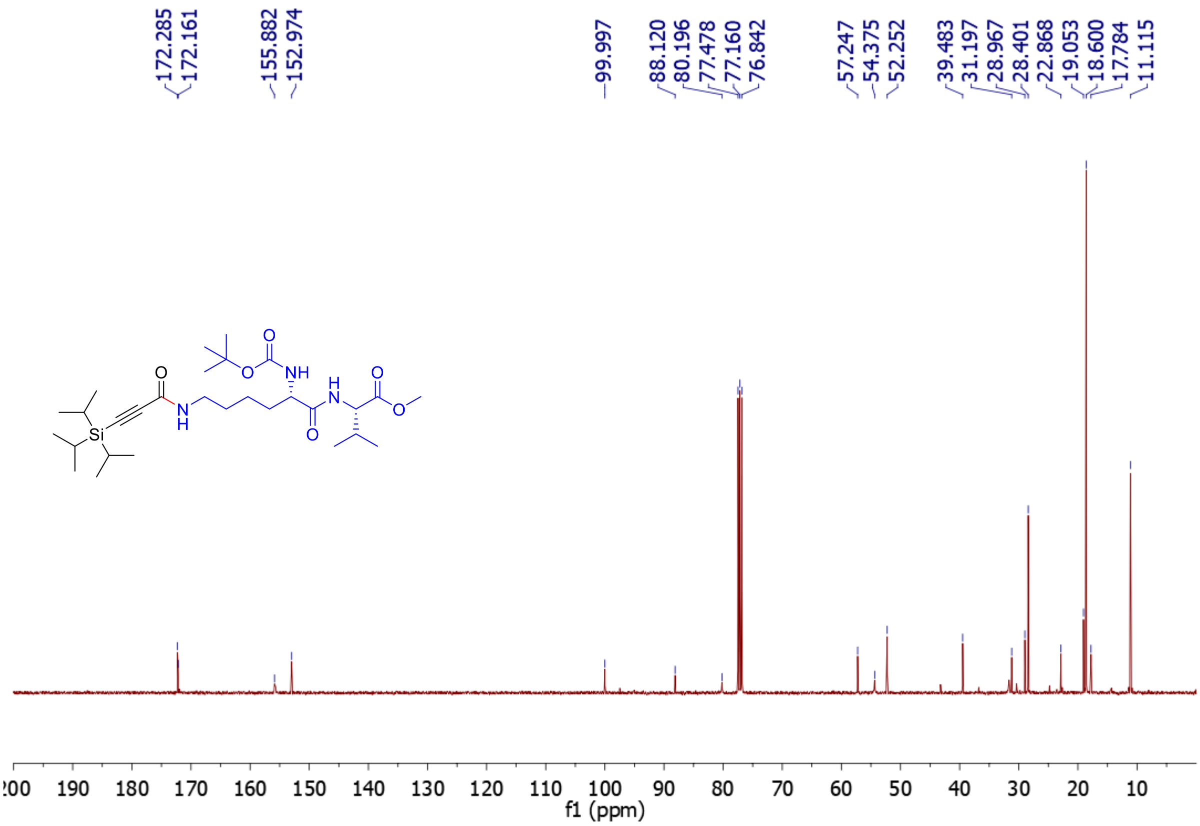

Fig. S119. ^{13}C NMR spectrum of methyl N2-(tert-butoxycarbonyl)-N6-(3-(triisopropylsilyl)propioloyl)-*L*-lysyl-*L*-valinate **(3am)** in $CDCl_3$.

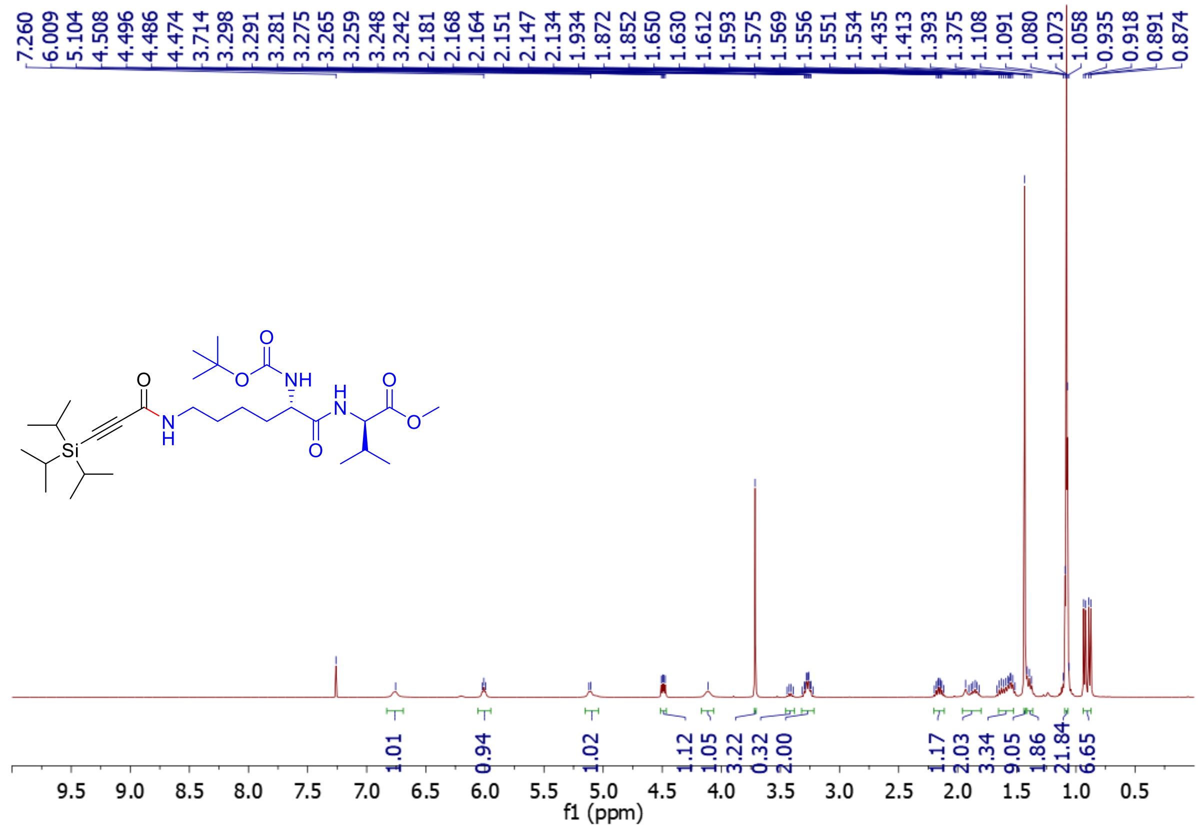

Fig. S120. ^{1}H NMR spectrum of methyl N2-(tert-butoxycarbonyl)-N6-(3-(triisopropylsilyl)propioloyl)-*L*-lysyl-*D*-valinate **(3am')** in $CDCl_3$.

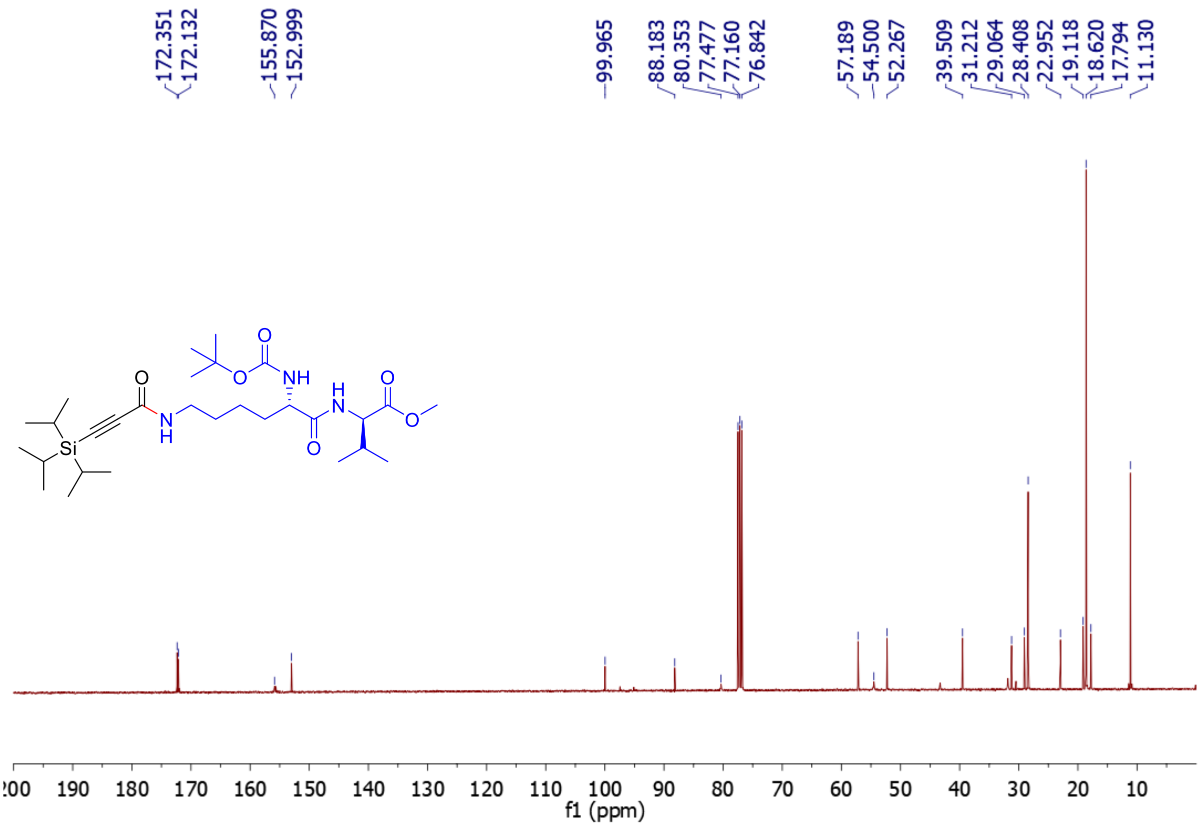

Fig. S121. ^{13}C NMR spectrum of methyl N2-(tert-butoxycarbonyl)-N6-(3-(triisopropylsilyl)propioloyl)-*L*-lysyl-*D*-valinate **(3am')** in $CDCl_3$.

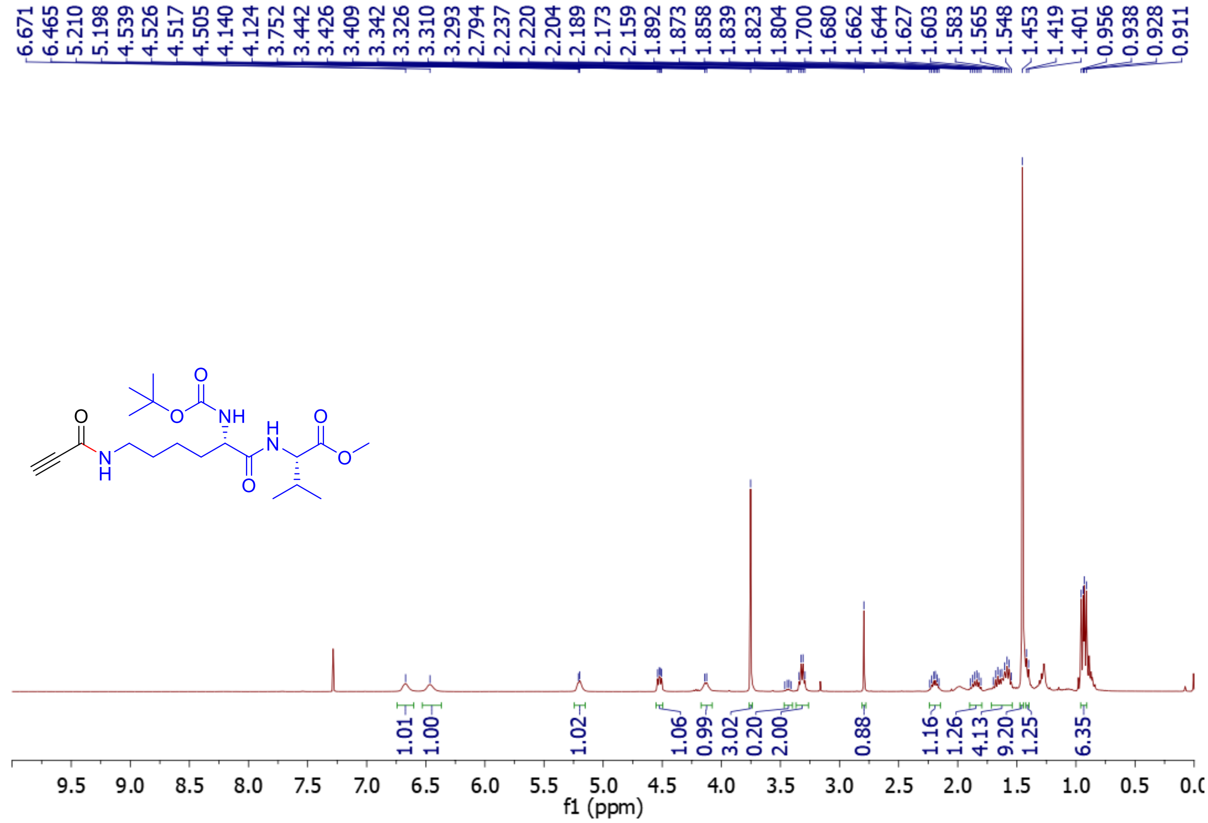

Fig. S122. ^{1}H NMR spectrum of methyl N2-(tert-butoxycarbonyl)-N6-propioloyl-*L*-lysyl-*L*-valinate **(5am)** in $CDCl_3$.

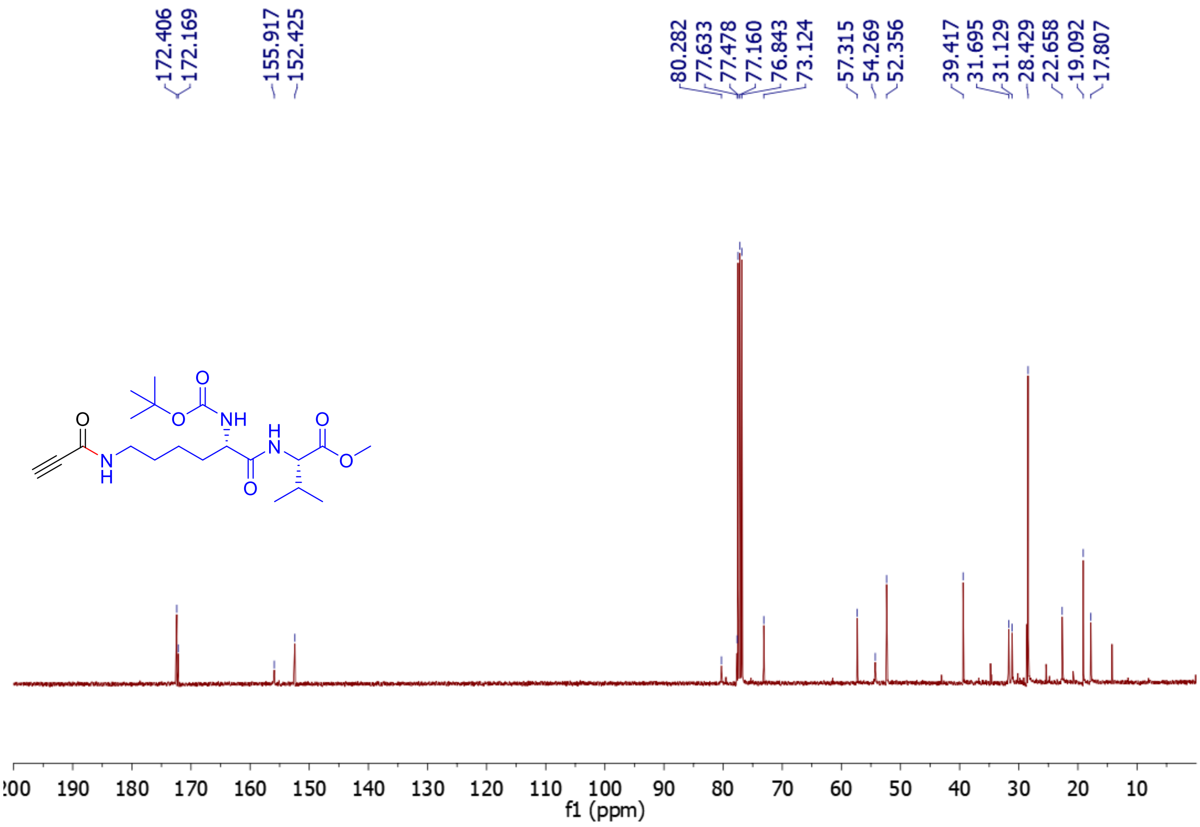

Fig. S123. ^{13}C NMR spectrum of methyl N2-(tert-butoxycarbonyl)-N6-propioloyl-*L*-lysyl-*L*-valinate **(5am)** in $CDCl_3$.

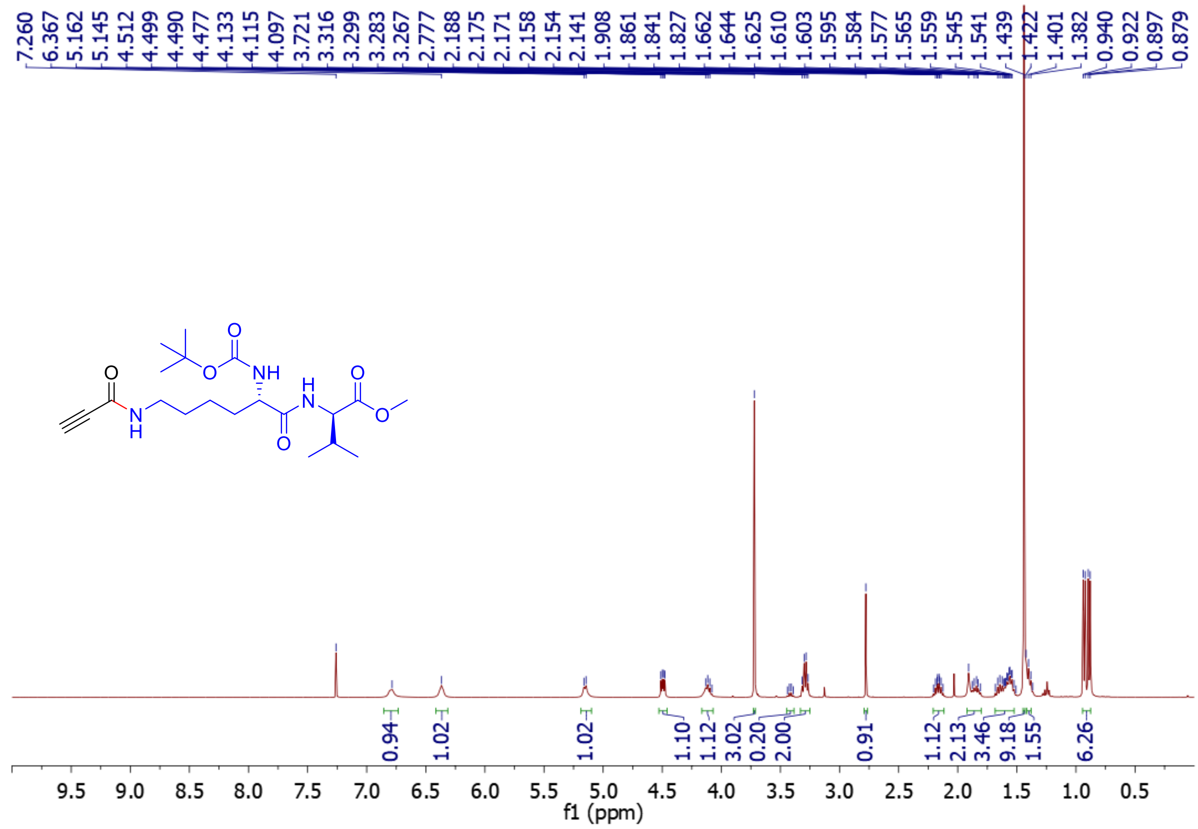

Fig. S124. ^{1}H NMR spectrum of methyl N2-(tert-butoxycarbonyl)-N6-propioloyl-*L*-lysyl-*D*-valinate **(5am')** in $CDCl_3$.

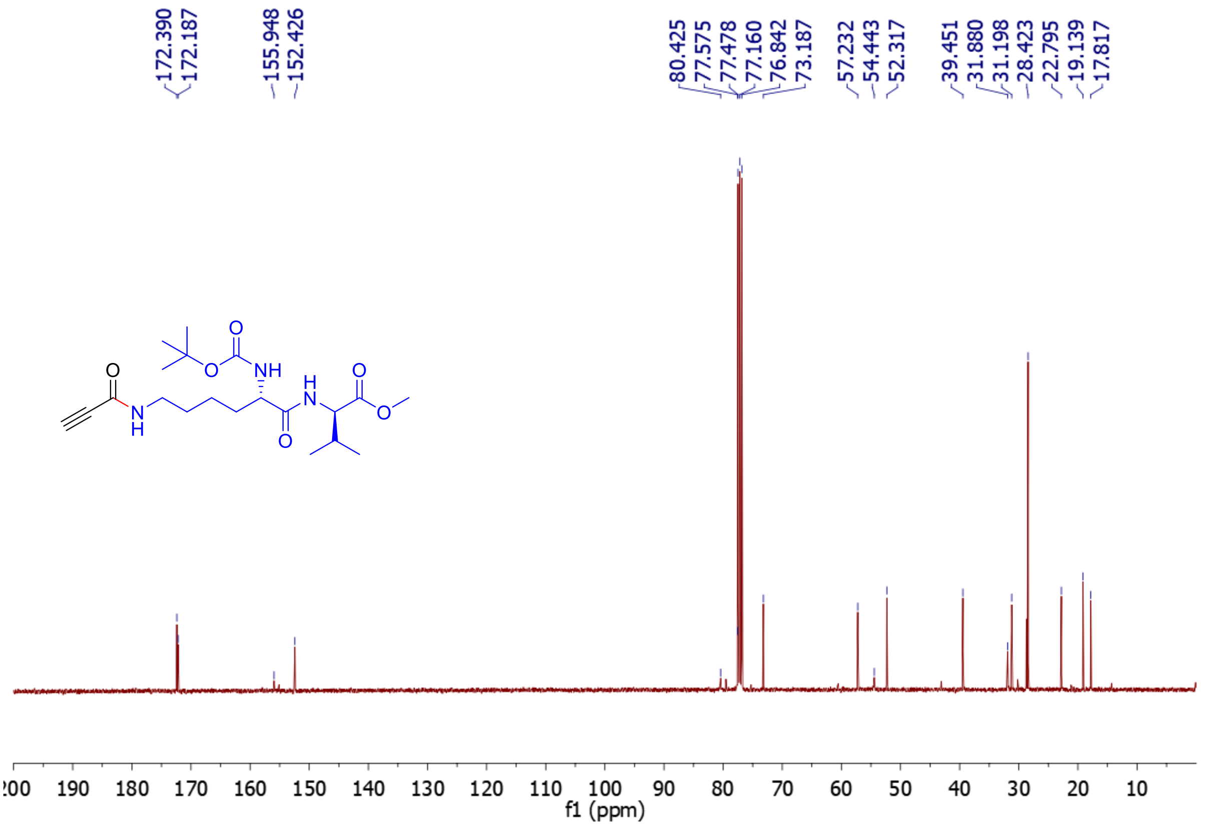

Fig. S125. ^{13}C NMR spectrum of methyl N2-(tert-butoxycarbonyl)-N6-propioloyl-*L*-lysyl-*D*-valinate **(5am')** in $CDCl_3$.

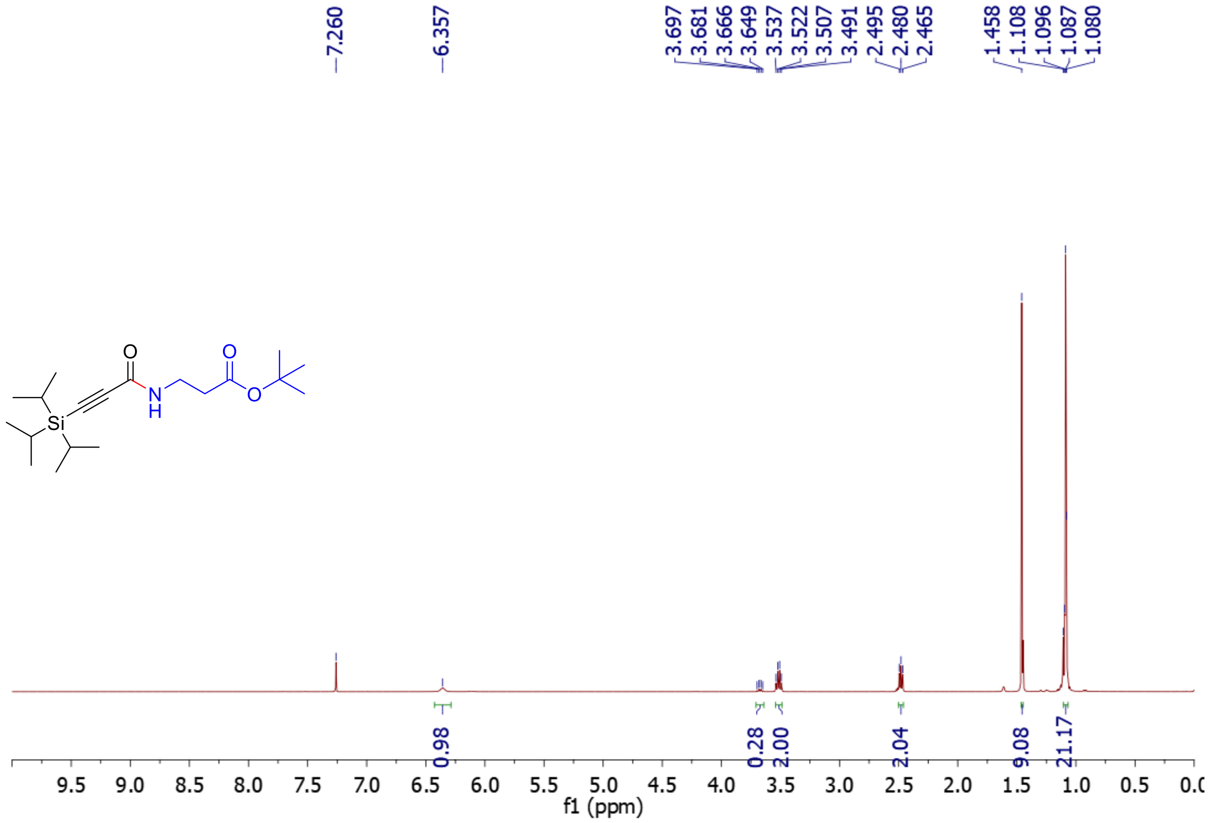

Fig. S126. ^{1}H NMR spectrum of tert-butyl 3-(3-(triisopropylsilyl)propiolamido)propanoate **(3ap)** in $CDCl_3$.

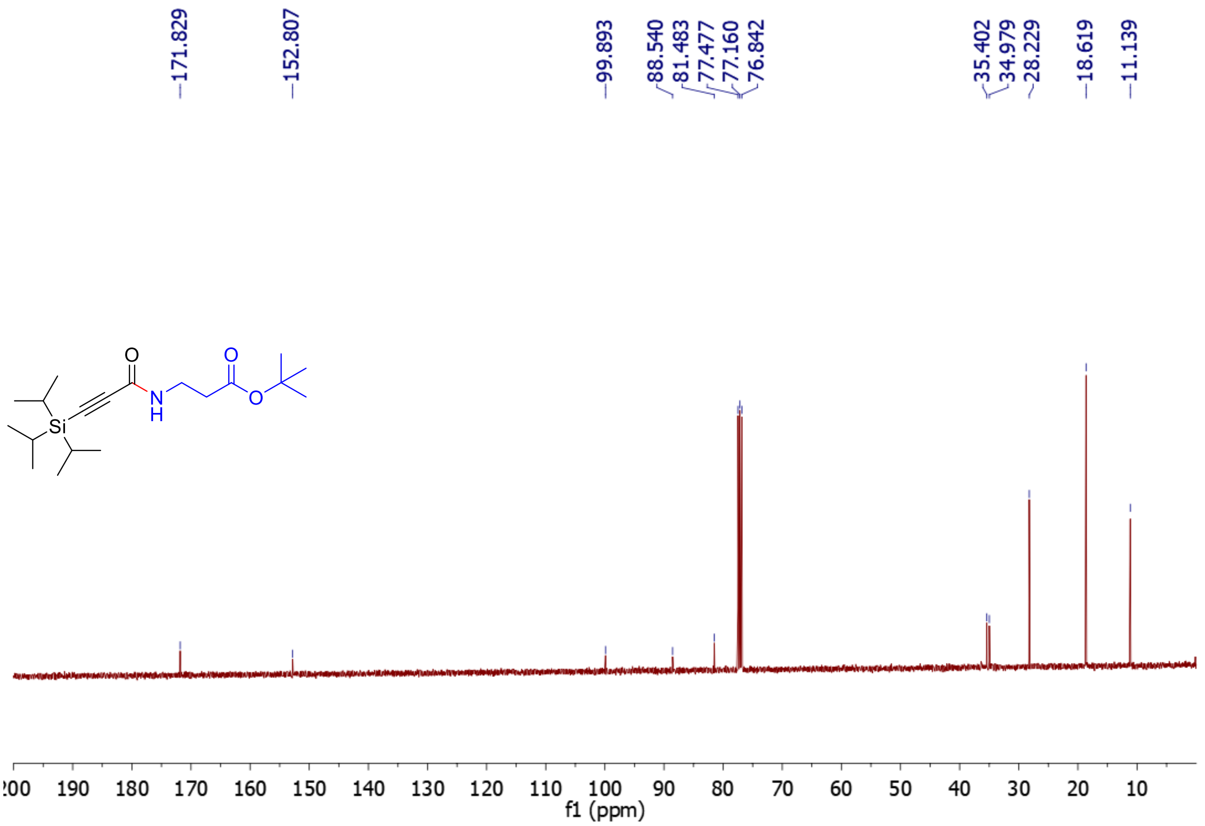

Fig. S127. ^{1}H NMR spectrum of tert-butyl 3-(3-(triisopropylsilyl)propiolamido)propanoate **(3ap)** in $CDCl_3$.

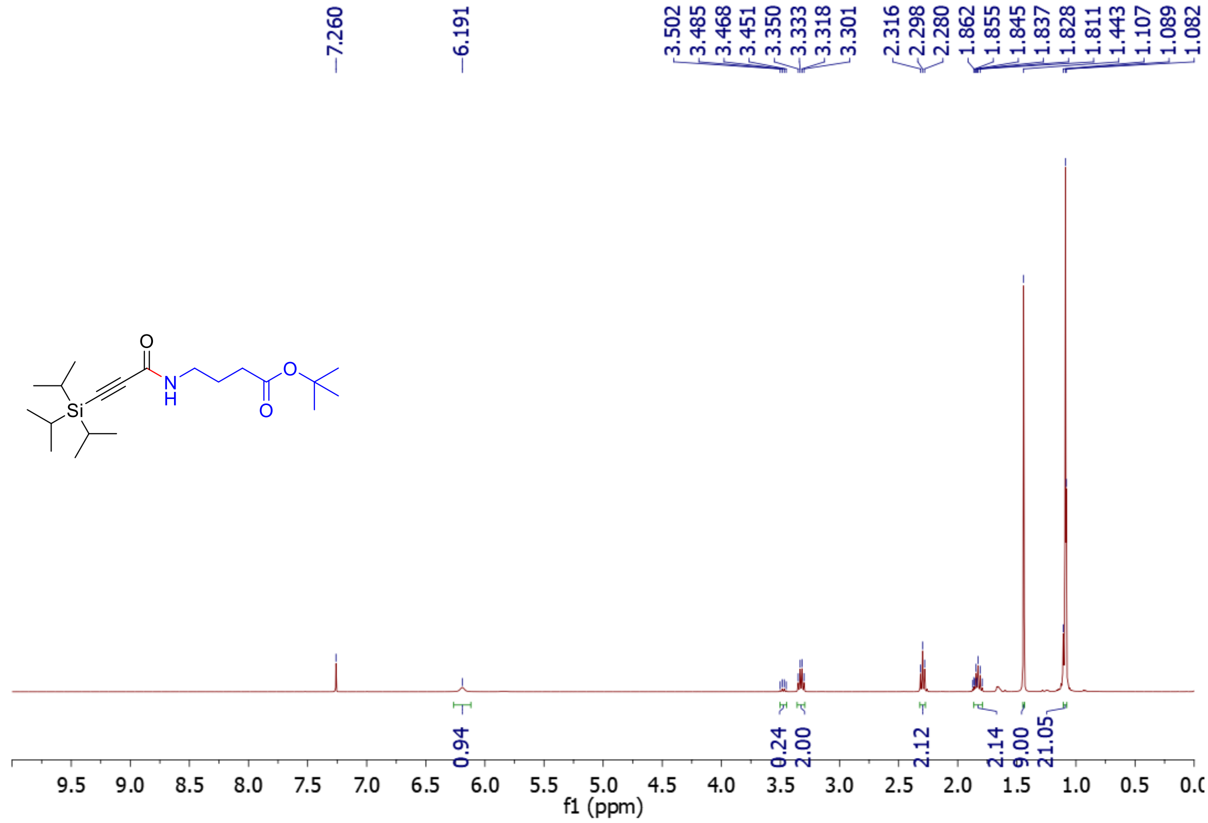

Fig. S128. ^{1}H NMR spectrum of tert-butyl 4-(3-(triisopropylsilyl)propiolamido)butanoate **(3aq)** in $CDCl_3$.

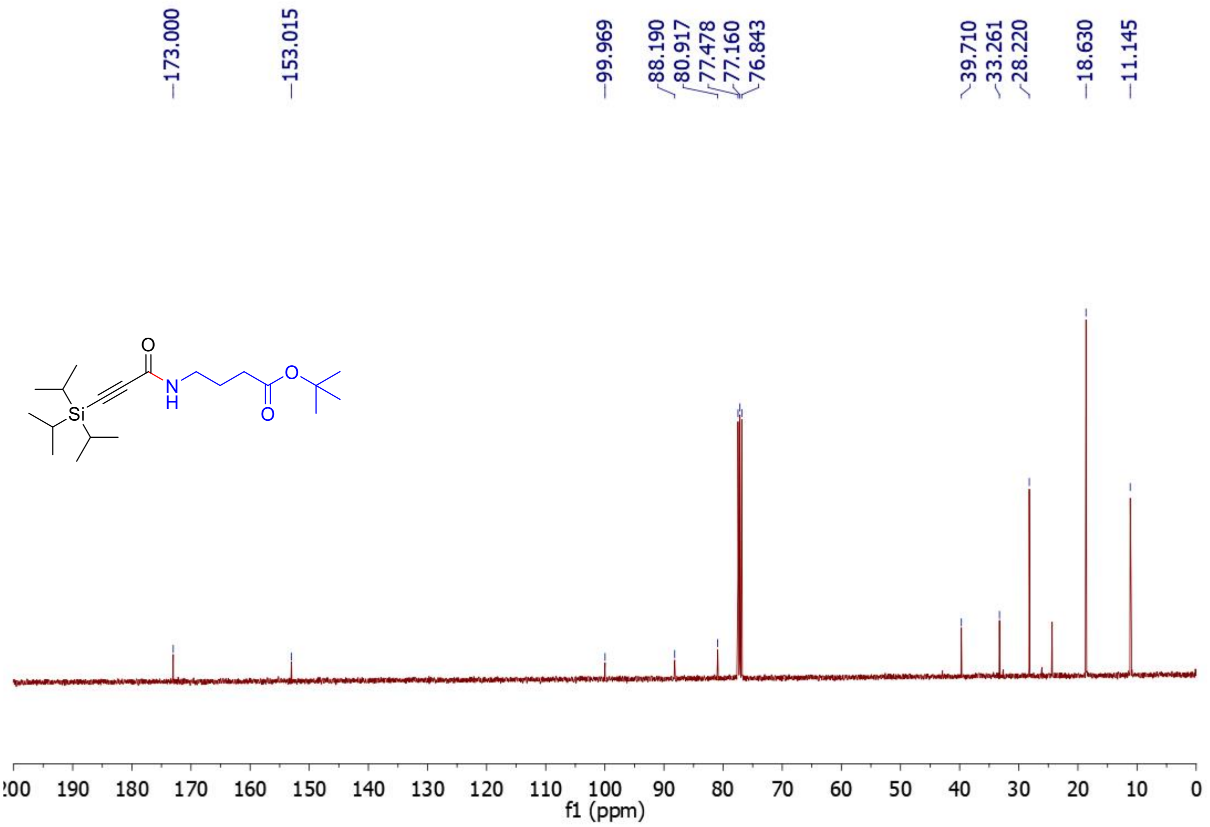

Fig. S129. ^{13}C NMR spectrum of tert-butyl 4-(3-(triisopropylsilyl)propiolamido)butanoate **(3aq)** in $CDCl_3$.

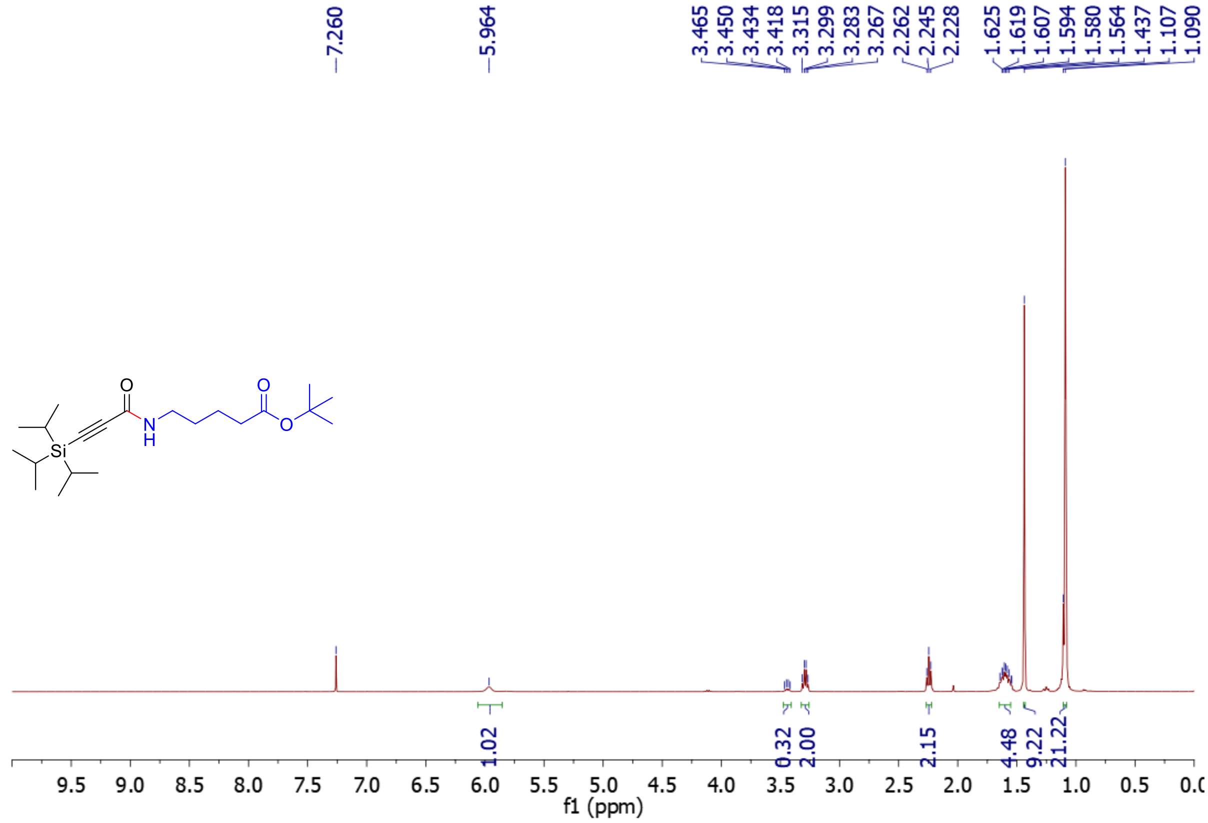

Fig. S130. ^{1}H NMR spectrum of tert-butyl 5-(3-(triisopropylsilyl)propiolamido)pentanoate **(3ar)** in $CDCl_3$.

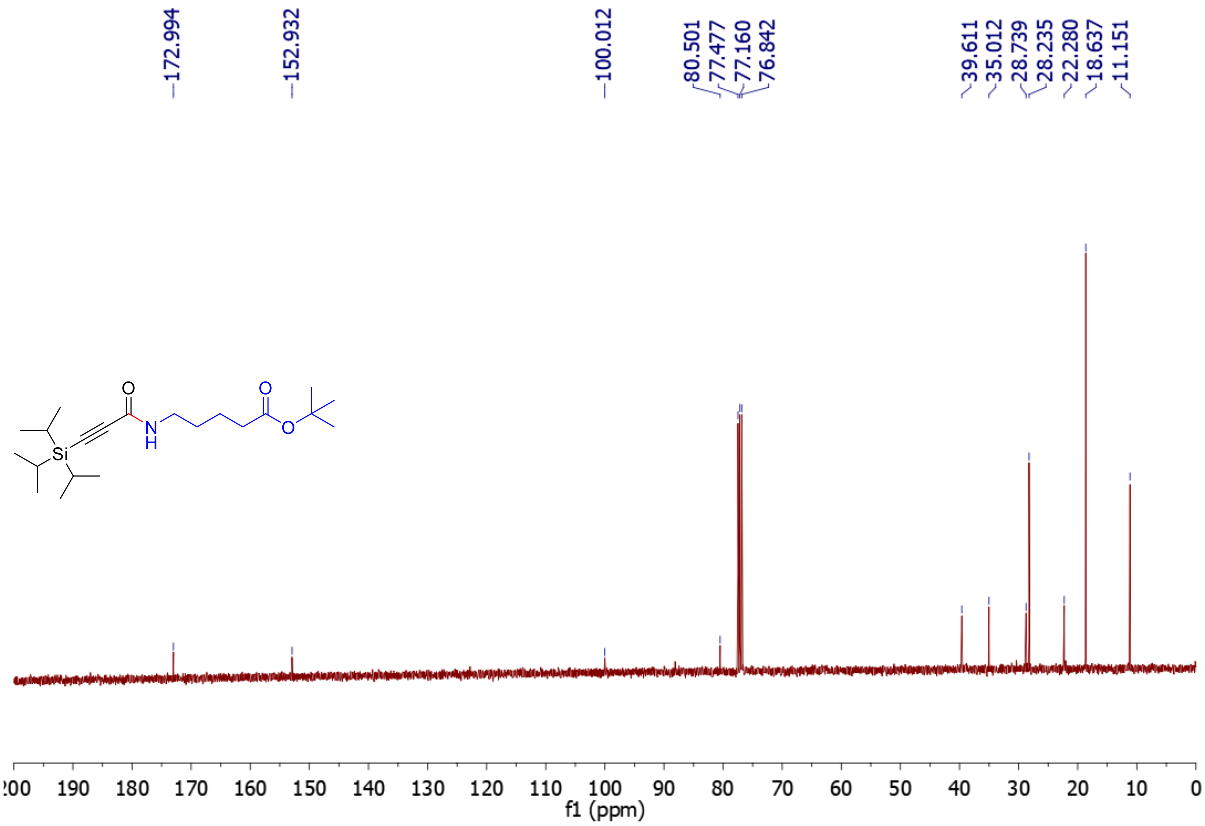

Fig. S131. ^{13}C NMR spectrum of tert-butyl 5-(3-(triisopropylsilyl)propiolamido)pentanoate **(3ar)** in $CDCl_3$.

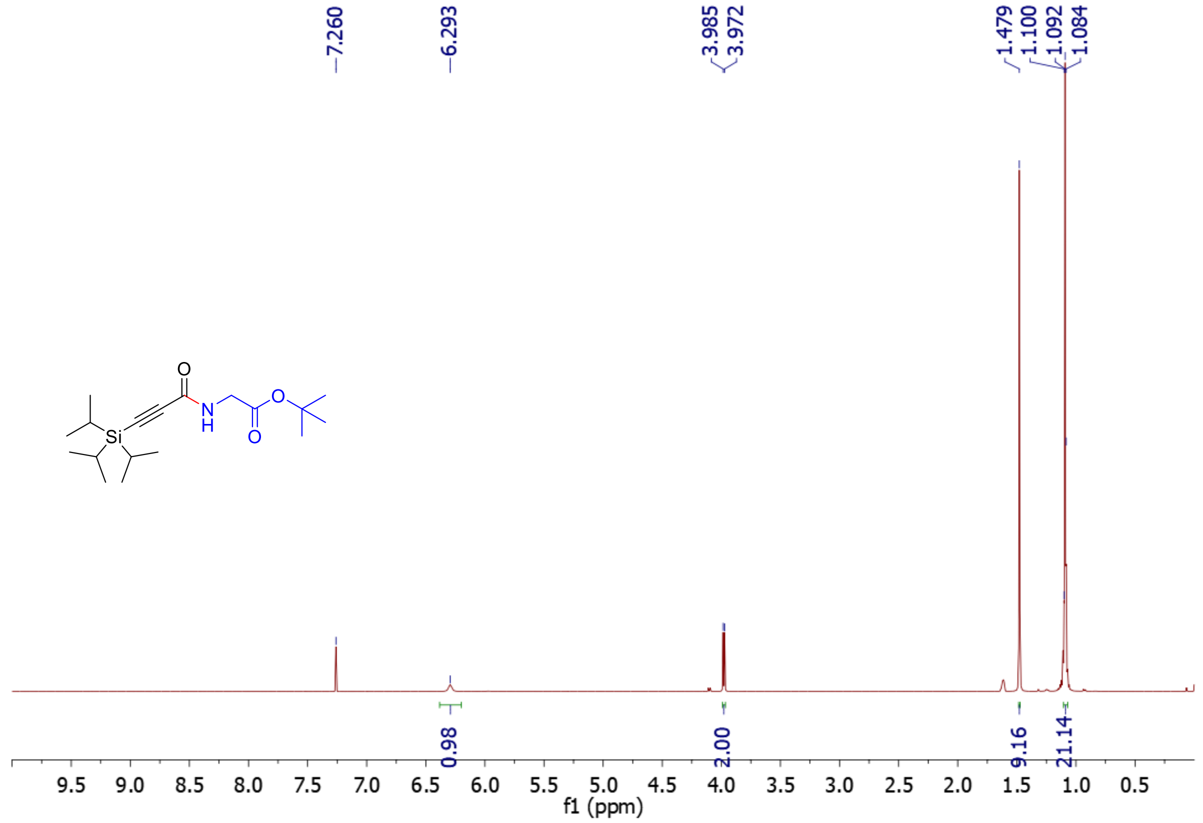

Fig. S132. ^{1}H NMR spectrum of tert-butyl (3-(triisopropylsilyl)propioloyl)glycinate **(3ao)** in $CDCl_3$.

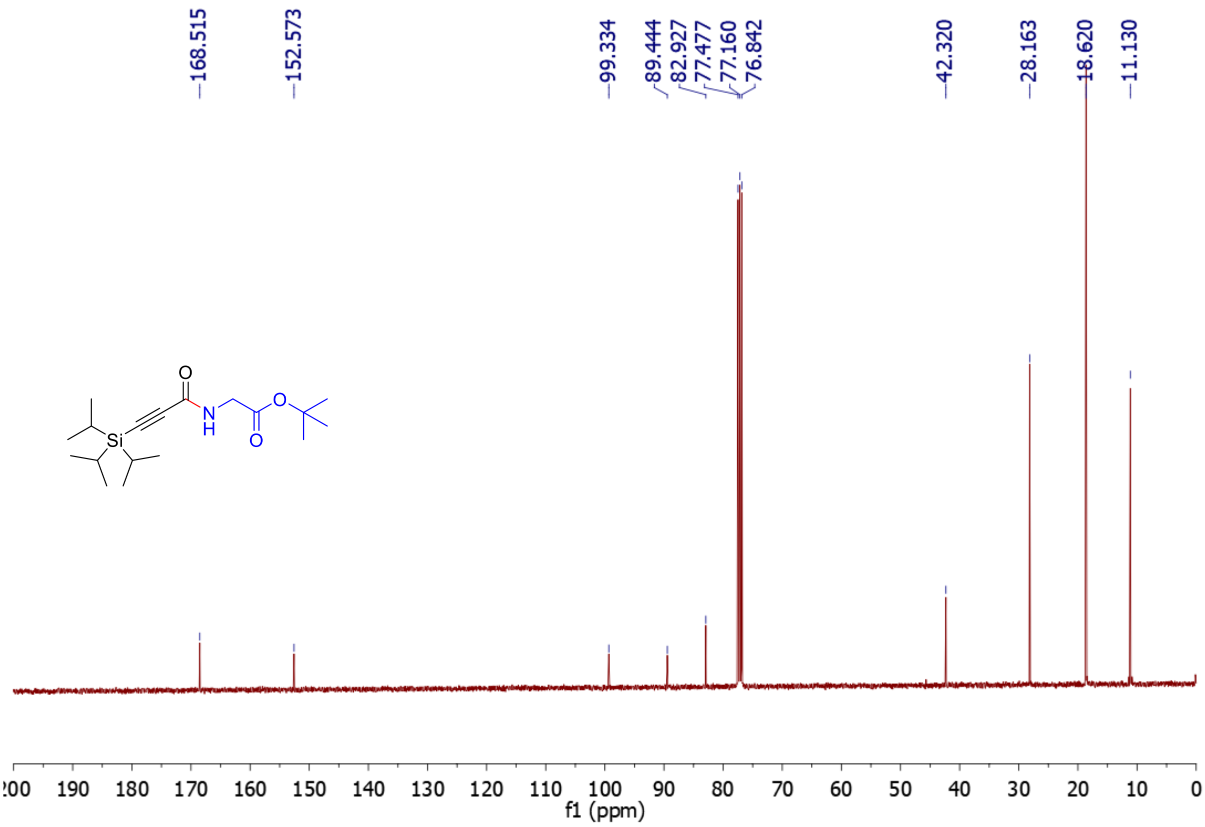

Fig. S133. ^{13}C NMR spectrum of tert-butyl (3-(triisopropylsilyl)propioloyl)glycinate **(3ao)** in $CDCl_3$.

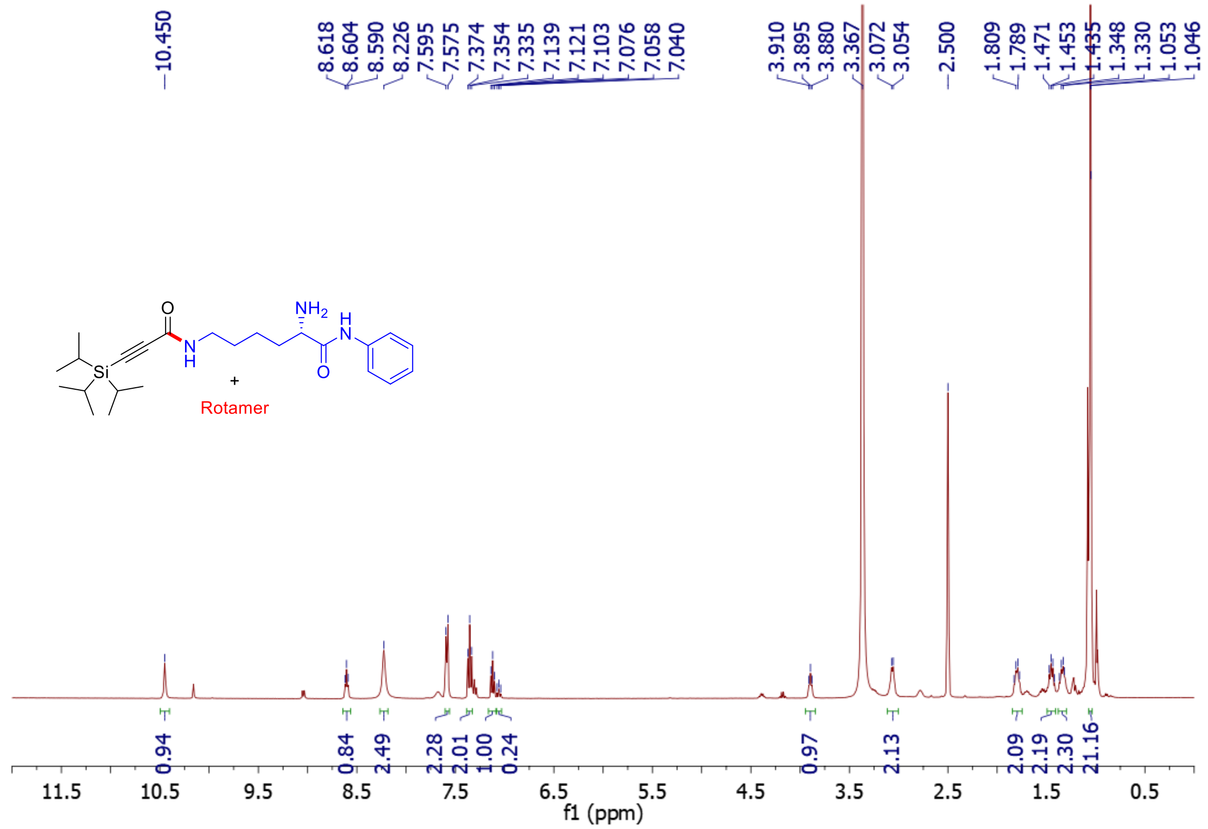

Fig. S134. ^{1}H NMR spectrum of (S)-2-amino-N-phenyl-6-(3-(triisopropylsilyl)propiolamido)hexanamide **(3as)** in DMSO-d_6.

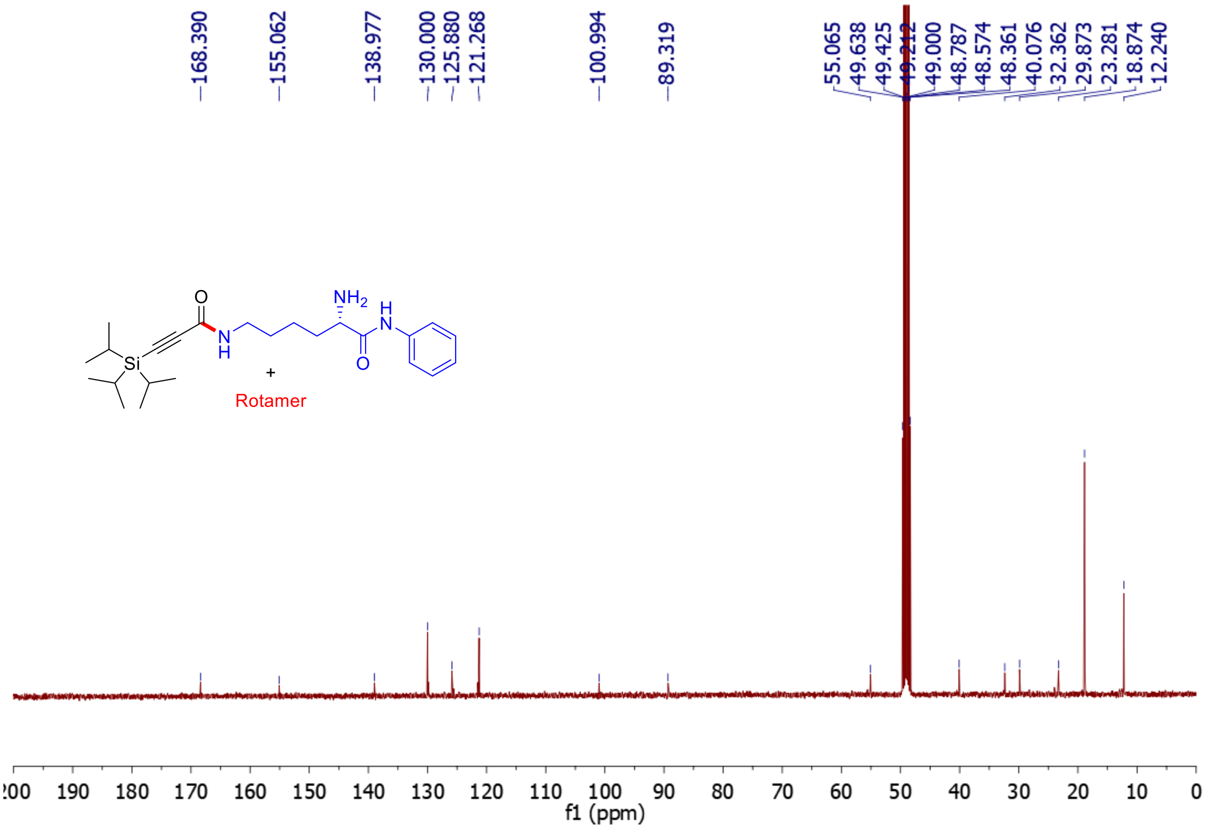

Fig. S135. ^{13}C NMR spectrum of (S)-2-amino-N-phenyl-6-(3-(triisopropylsilyl)propiolamido)hexanamide **(3as)** in MeOH-d_4.

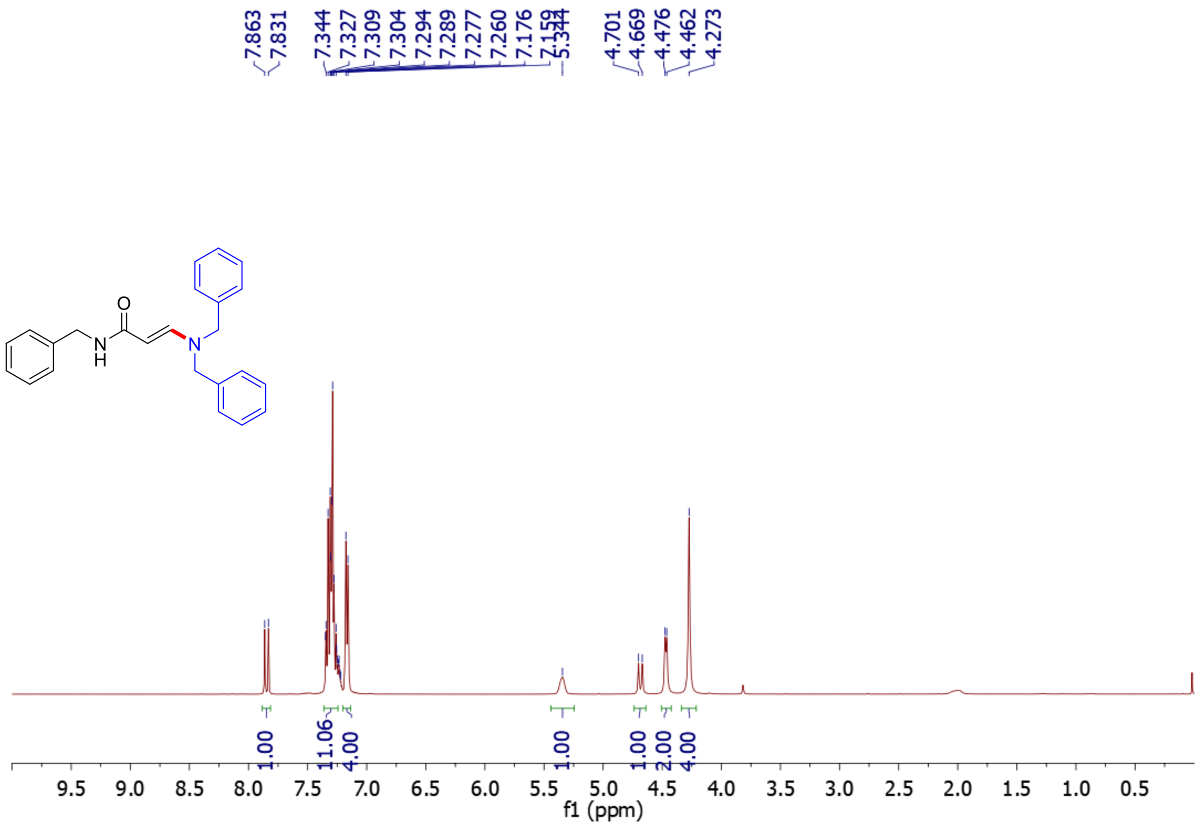

Fig. S136. ^{1}H NMR spectrum of (E)-N-benzyl-3-(dibenzylamino)acrylamide **(16a)** in $CDCl_3$.

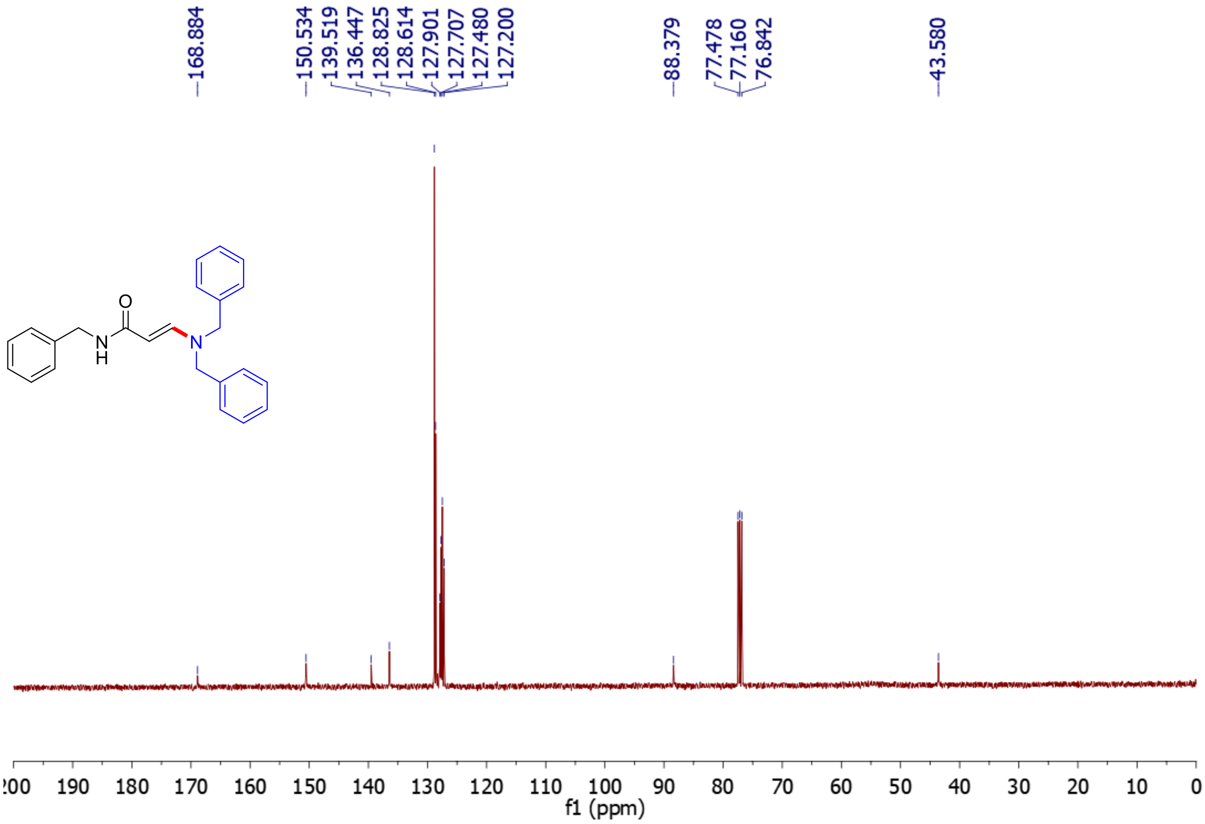

Fig. S137. ^{13}C NMR spectrum of (E)-N-benzyl-3-(dibenzylamino)acrylamide **(16a)** in $CDCl_3$.

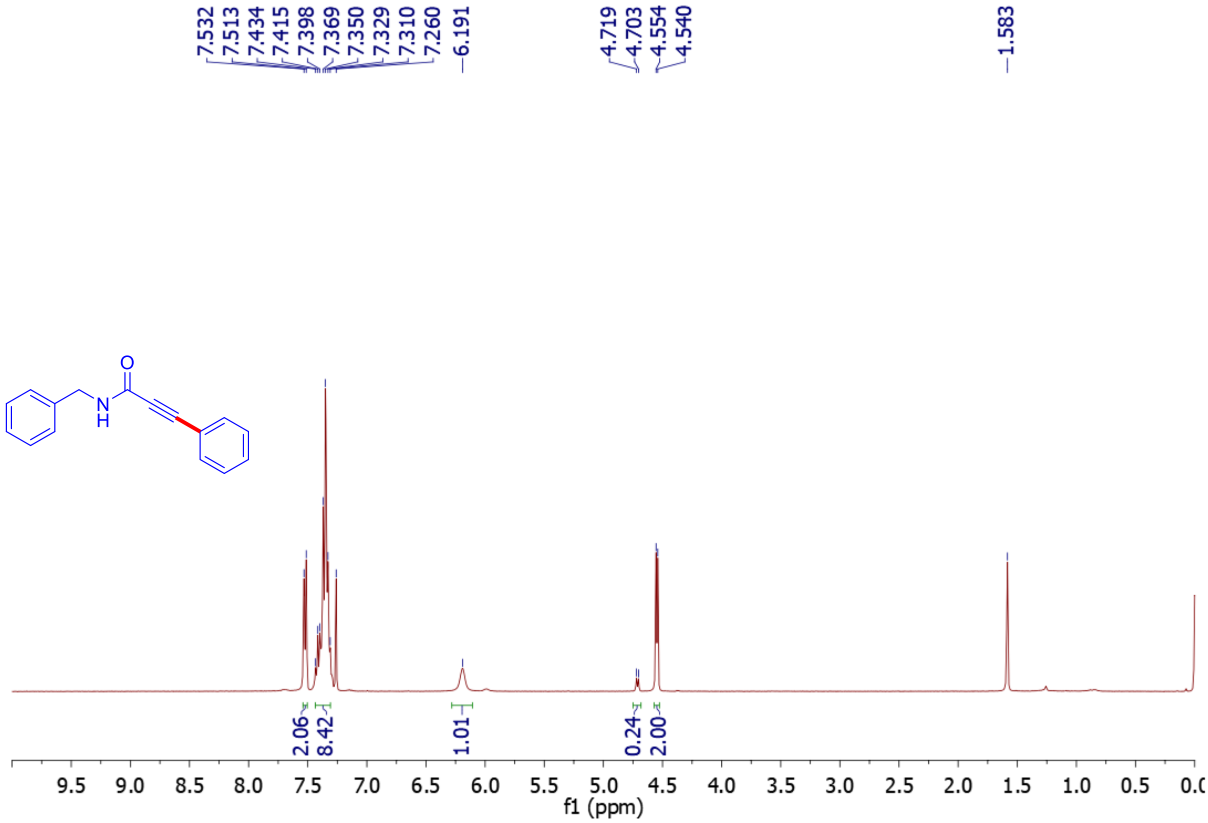

Fig. S138. ^{1}H NMR spectrum of N-benzyl-3-phenylpropiolamide **(17a)** in $CDCl_3$.

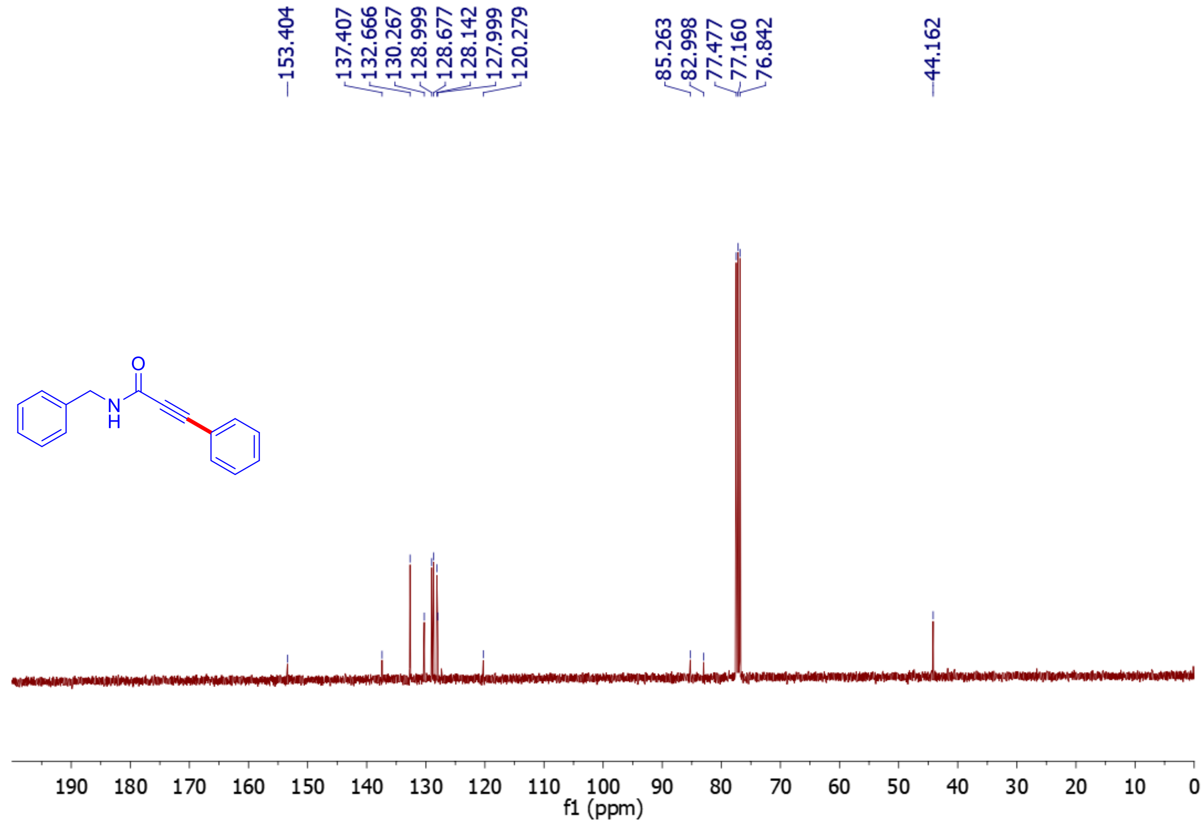

Fig. S139. ^{13}C NMR spectrum of N-benzyl-3-phenylpropiolamide **(17a)** in $CDCl_3$.

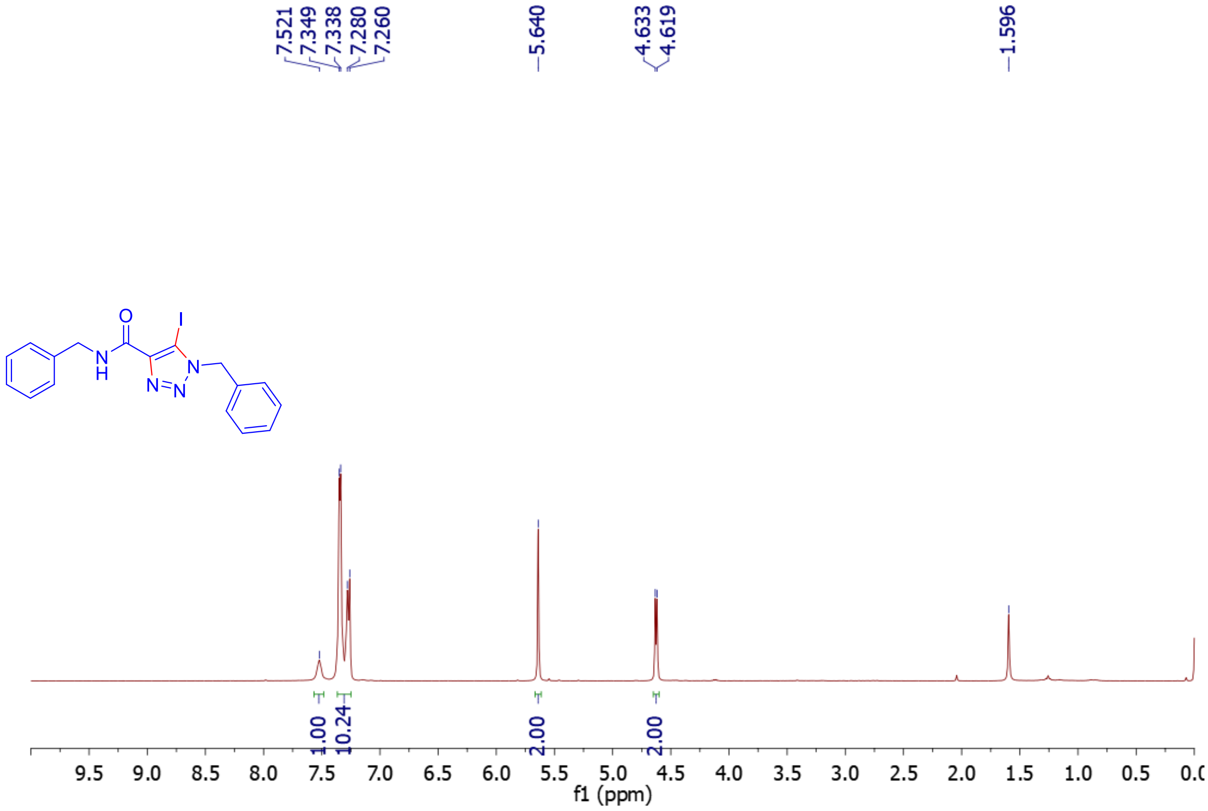

Fig. S140. ^{1}H NMR spectrum of N,1-dibenzyl-5-iodo-1H-1,2,3-triazole-4-carboxamide **(18a)** in $CDCl_3$.

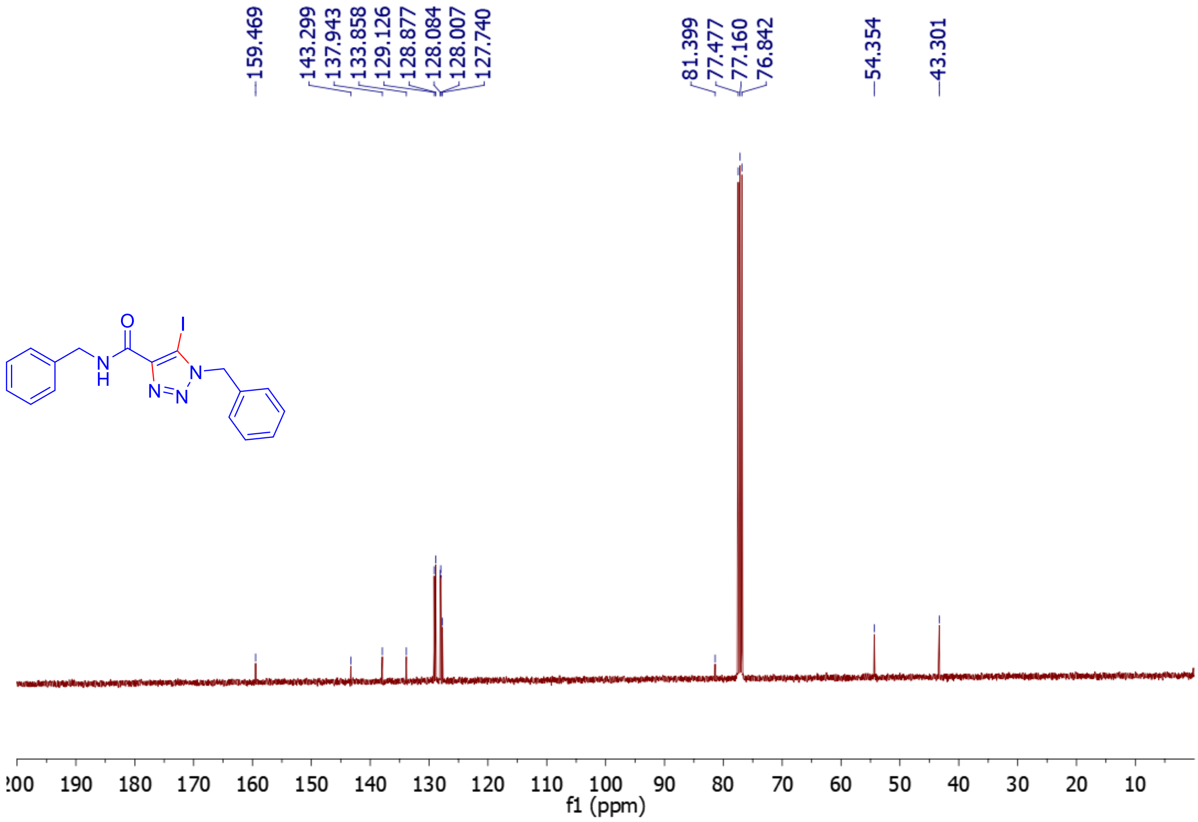

Fig. S141. ^{13}C NMR spectrum of N,1-dibenzyl-5-iodo-1H-1,2,3-triazole-4-carboxamide **(18a)** in $CDCl_3$.

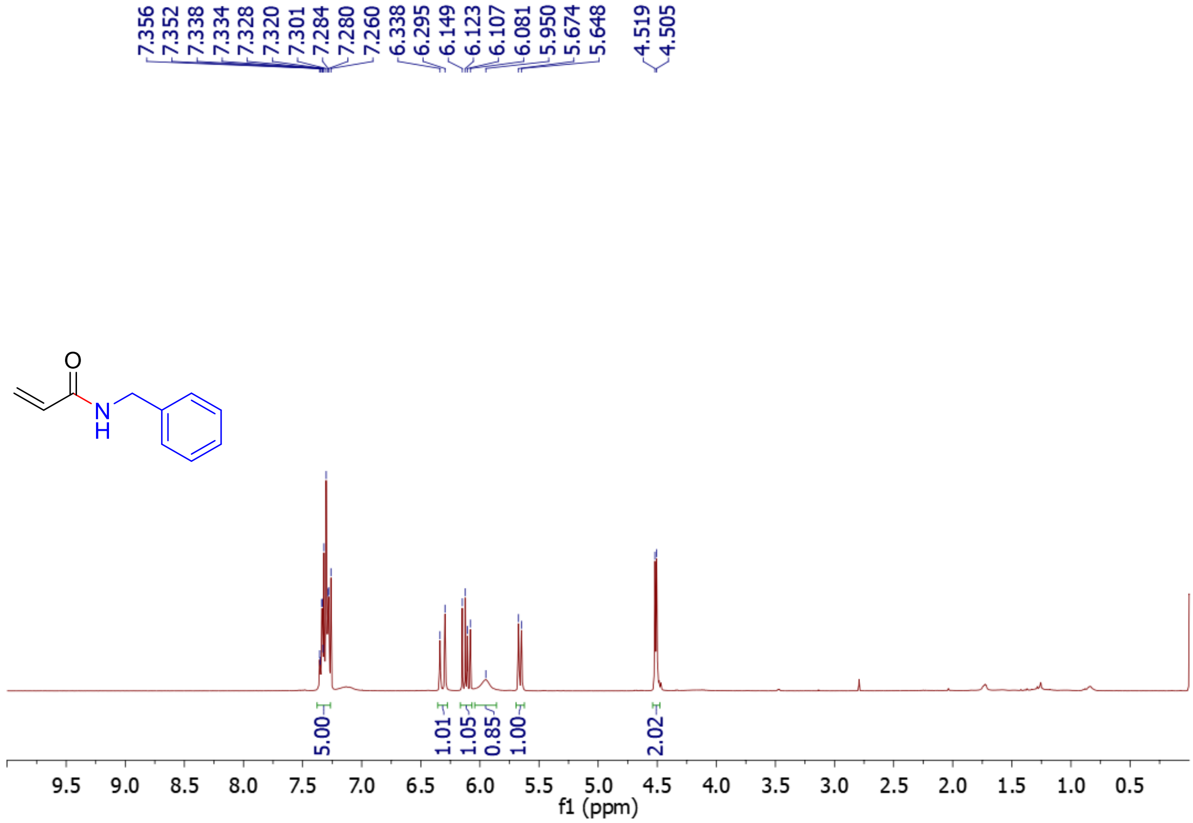

Fig. S142. ^{1}H NMR spectrum of N-benzylacrylamide **(19a)** in $CDCl_3$.

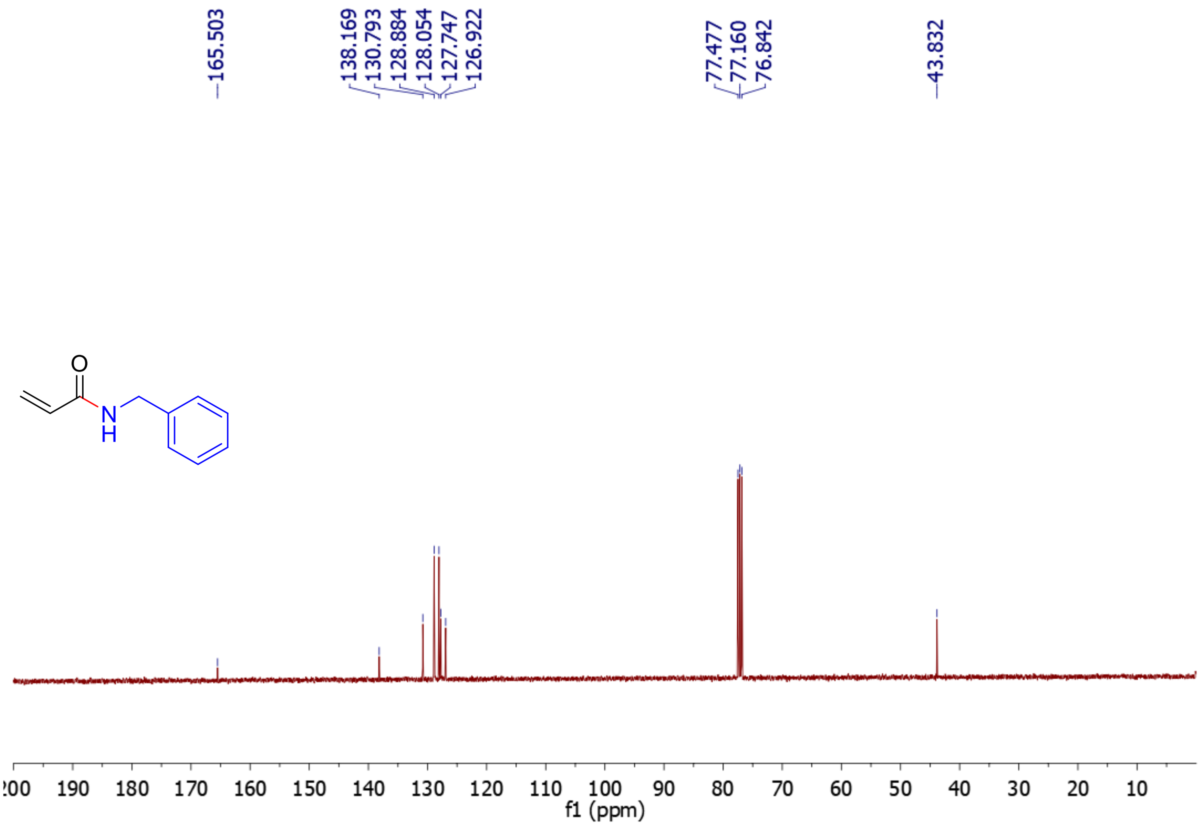

Fig. S143. ^{13}C NMR spectrum of N-benzylacrylamide **(19a)** in $CDCl_3$.

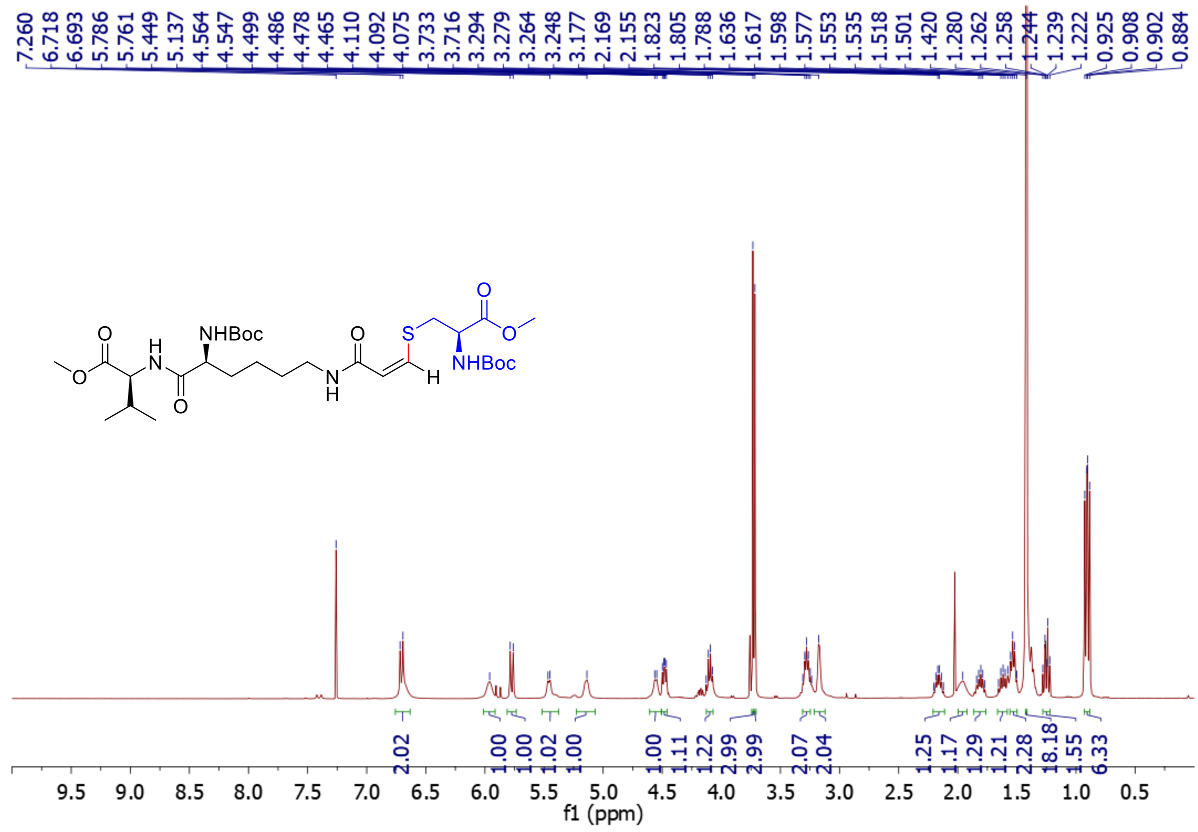

Fig. S144. ^{1}H NMR spectrum of methyl (6R,17S,20S,Z)-17-((tert-butoxycarbonyl)amino)-20-isopropyl-6-(methoxycarbonyl)-2,2-dimethyl-4,11,18-trioxo-3-oxa-8-thia-5,12,19-triazahenicos-9-en-21-oate **(20a)** in $CDCl_3$.

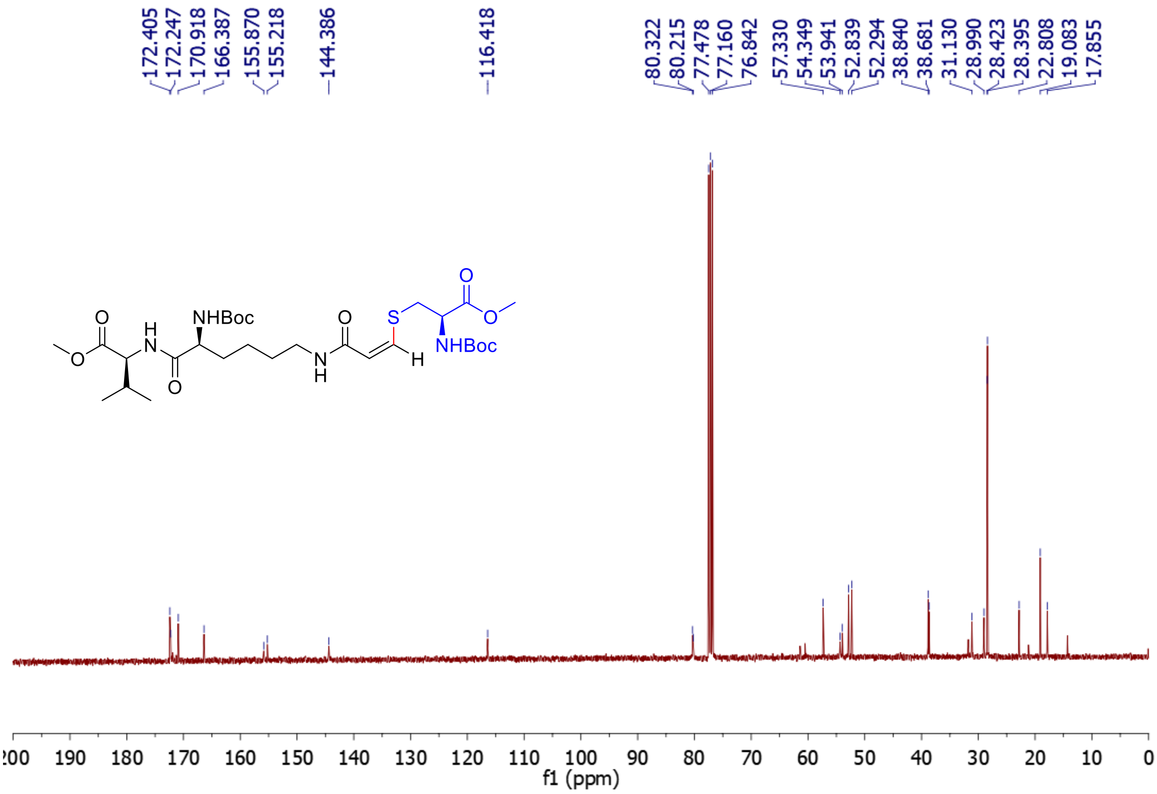

Fig. S145. ^{13}C NMR spectrum of methyl (6R,17S,20S,Z)-17-((tert-butoxycarbonyl)amino)-20-isopropyl-6-(methoxycarbonyl)-2,2-dimethyl-4,11,18-trioxo-3-oxa-8-thia-5,12,19-triazahenicos-9-en-21-oate **(20a)** in $CDCl_3$.

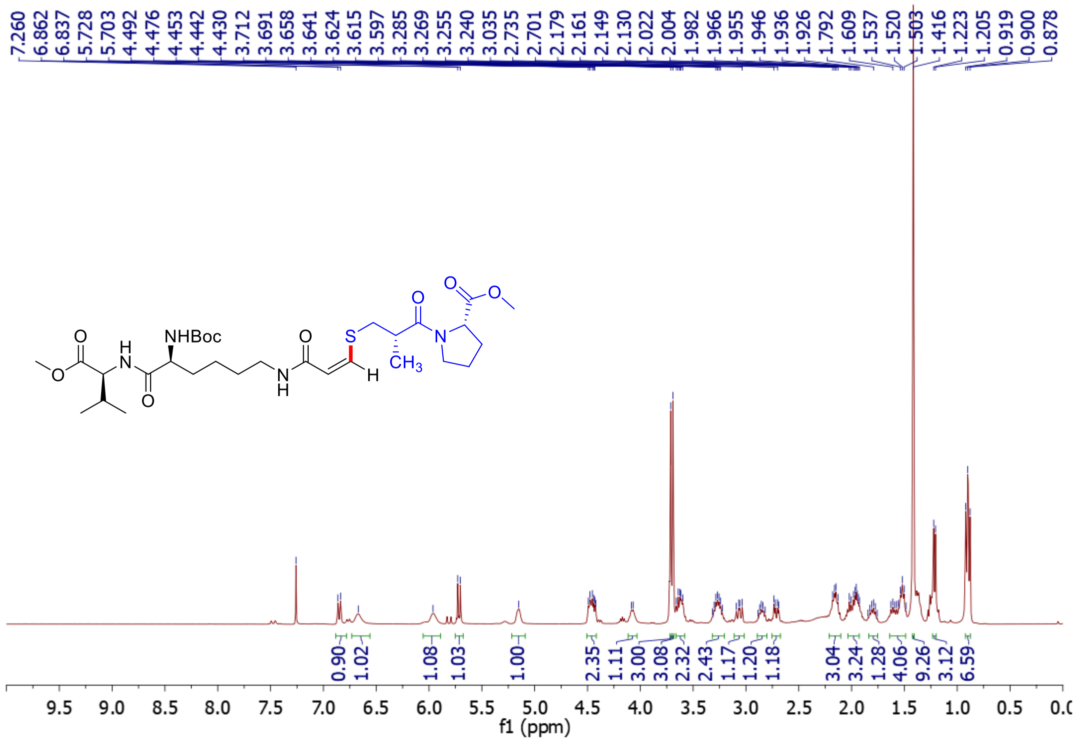

Fig. S146. ^{1}H NMR spectrum of methyl ((6S,17S,Z)-6-(((S)-1-methoxy-3-methyl-1-oxobutan-2-yl)carbamoyl)-2,2,17-trimethyl-4,12-dioxo-3-oxa-15-thia-5,11-diazaoctadec-13-en-18-oyl)-L-prolinate **(21a)** in $CDCl_3$.

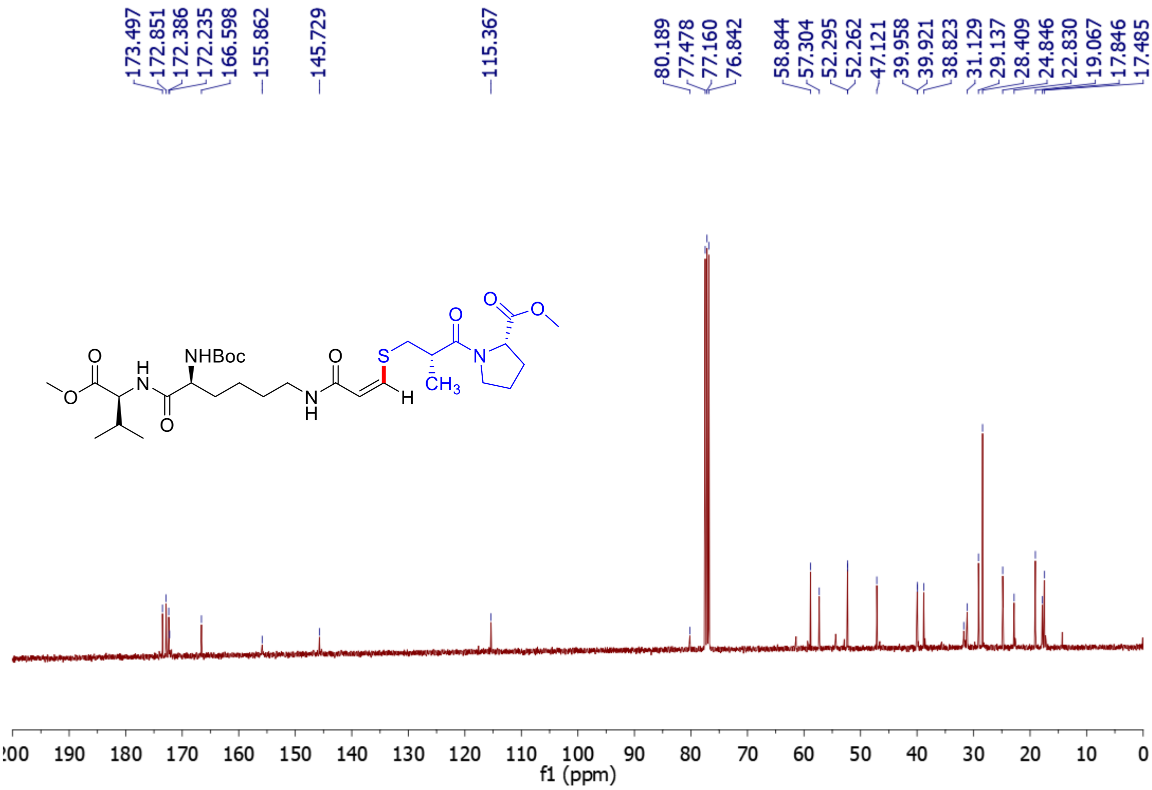

Fig. S147. ^{1}H NMR spectrum of methyl ((6S,17S,Z)-6-(((S)-1-methoxy-3-methyl-1-oxobutan-2-yl)carbamoyl)-2,2,17-trimethyl-4,12-dioxo-3-oxa-15-thia-5,11-diazaoctadec-13-en-18-oyl)-L-prolinate **(21a)** in $CDCl_3$.

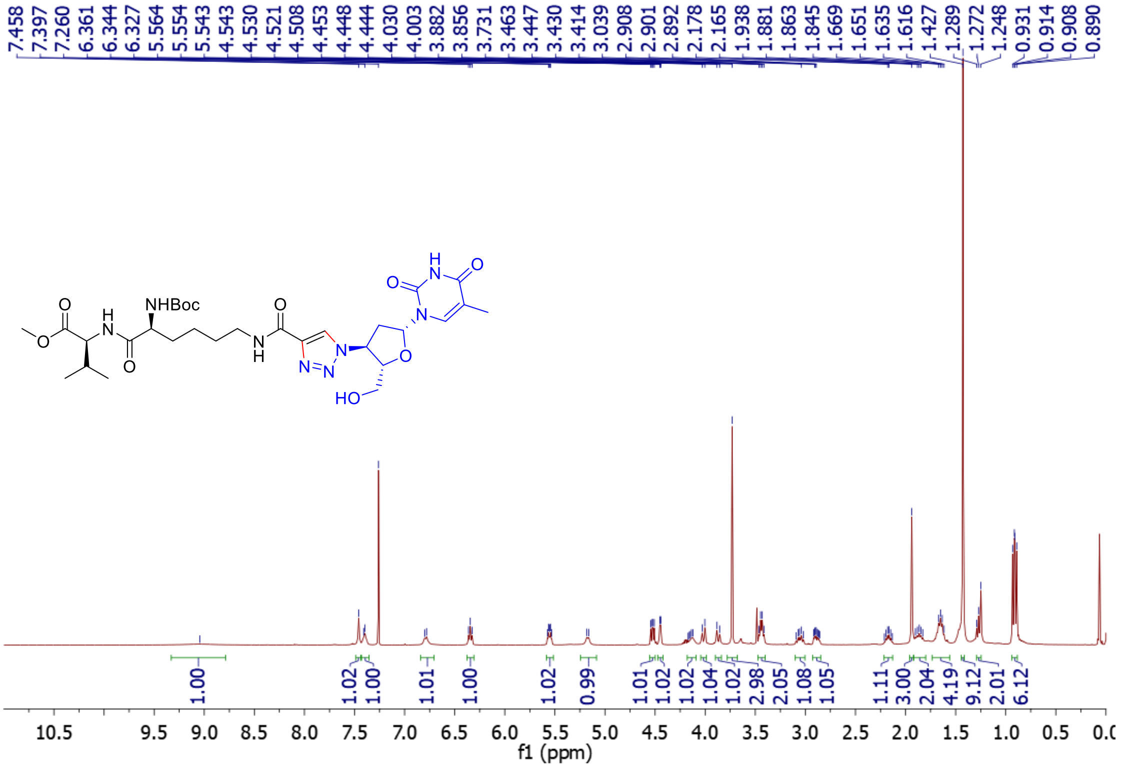

Fig. S148. ^{1}H NMR spectrum of methyl N2-(tert-butoxycarbonyl)-N6-(1-((2S,3S,5R)-2-(hydroxymethyl)-5-(5-methyl-2,4-dioxo-3,4-dihydropyrimidin-1(2H)-yl)tetrahydrofuran-3-yl)-1H-1,2,3-triazole-4-carbonyl)-L-lysyl-L-valinate **(22a)** in $CDCl_3$.

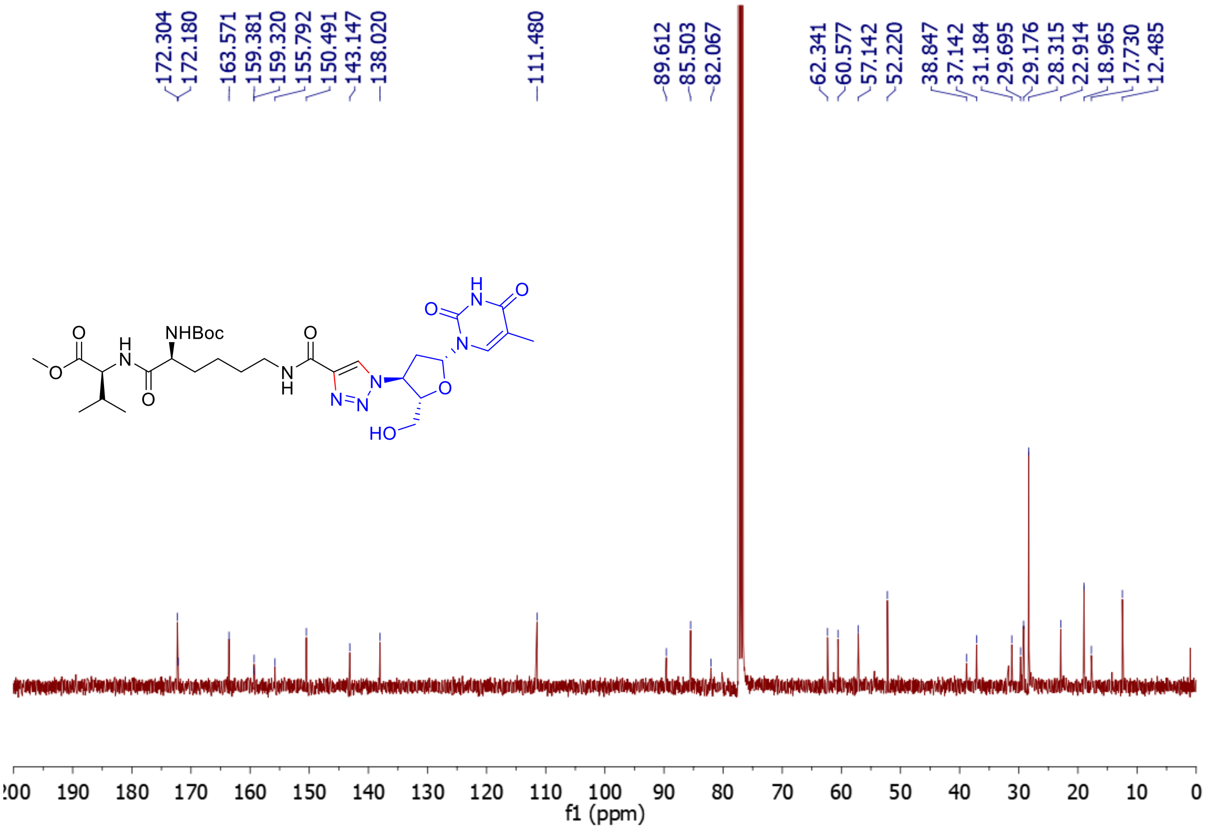

Fig. S149. ^{13}C NMR spectrum of methyl N2-(tert-butoxycarbonyl)-N6-(1-((2S,3S,5R)-2-(hydroxymethyl)-5-(5-methyl-2,4-dioxo-3,4-dihydropyrimidin-1(2H)-yl)tetrahydrofuran-3-yl)-1H-1,2,3-triazole-4-carbonyl)-L-lysyl-L-valinate **(22a)** in $CDCl_3$.

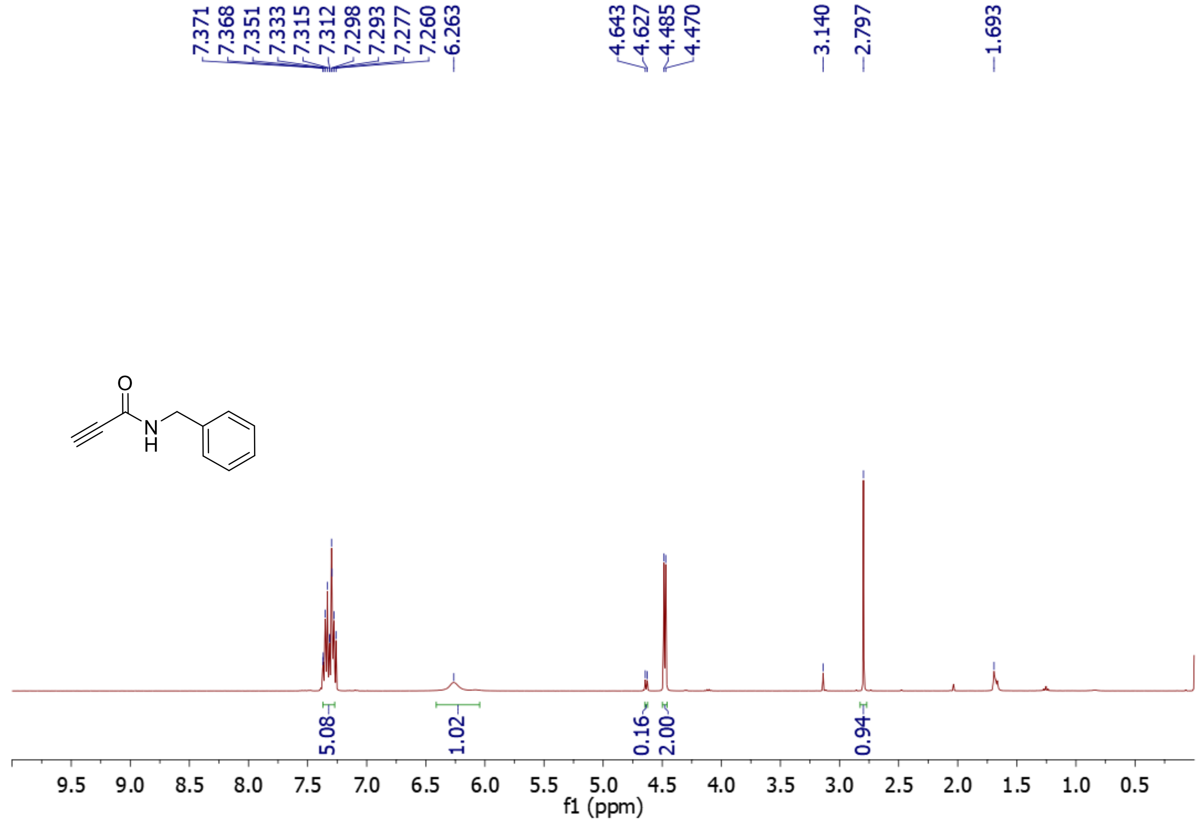

Fig. S150. ^{1}H NMR spectrum of N-benzylpropiolamide (**5a**) in $CDCl_3$.

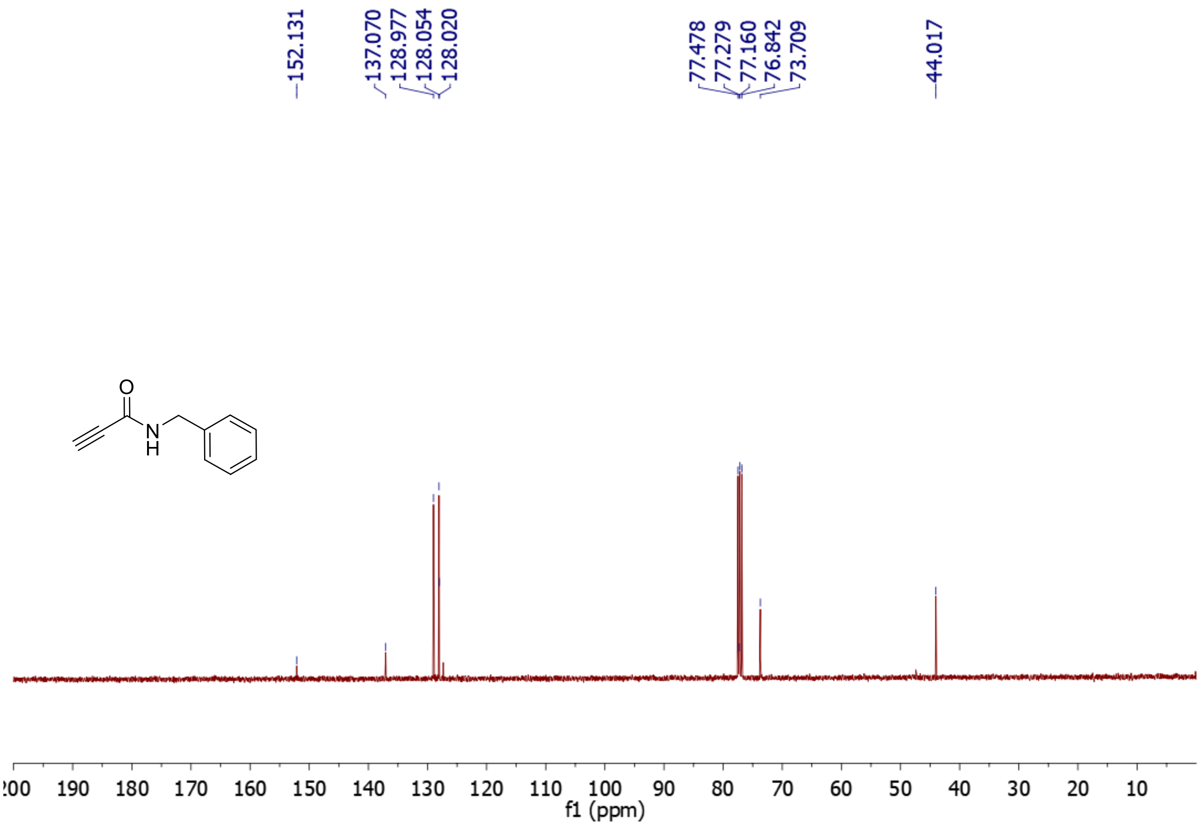

Fig. S151. ^{13}C NMR spectrum of N-benzylpropiolamide (**5a**) in $CDCl_3$.

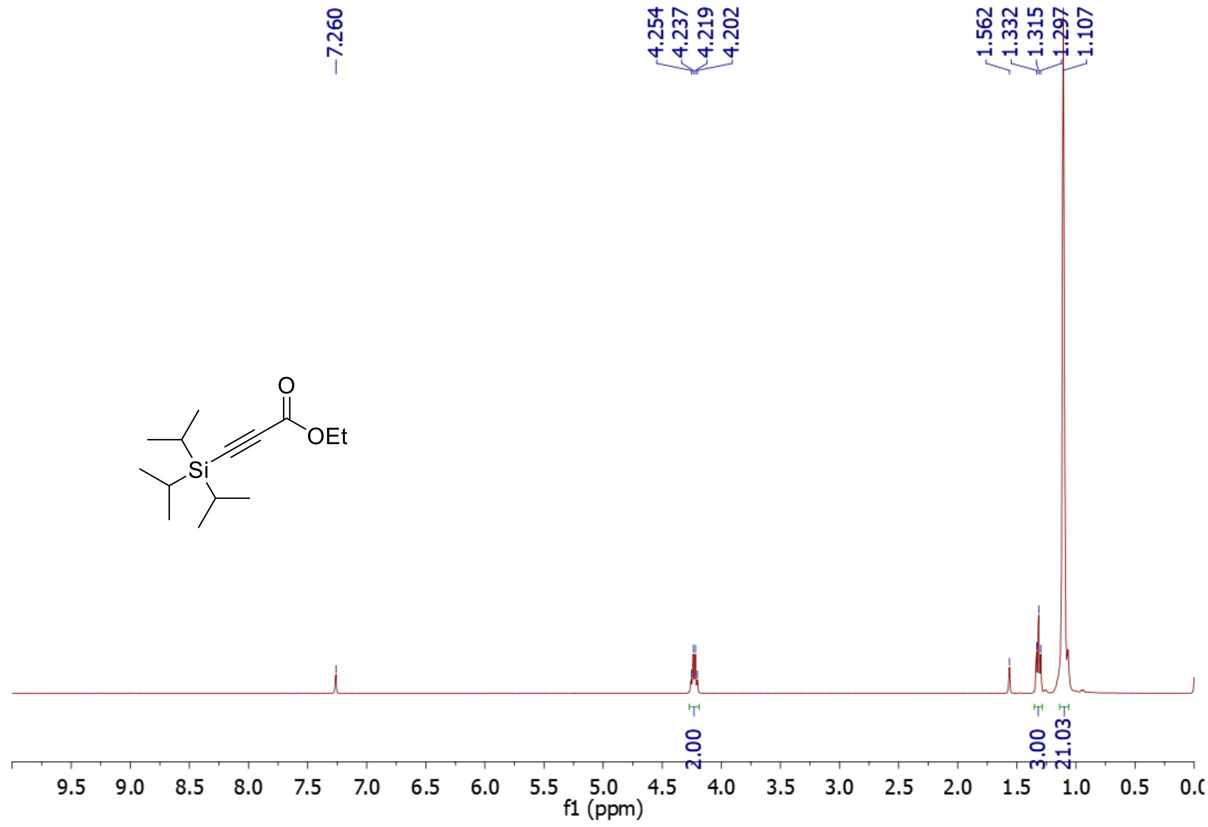

Fig. S152. ^{1}H NMR spectrum of ethyl 3-(triisopropylsilyl)propiolate in $CDCl_3$.

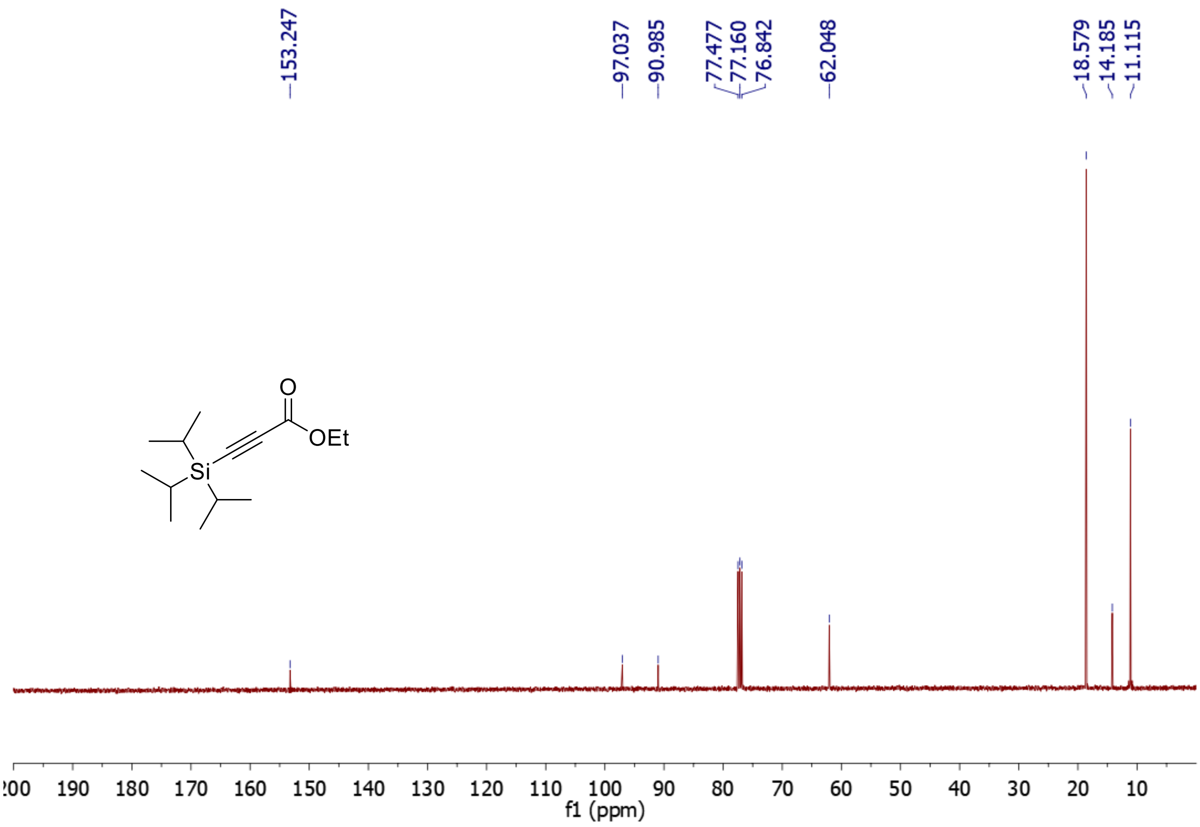

Fig. S153. ^{13}C NMR spectrum of ethyl 3-(triisopropylsilyl)propiolate in $CDCl_3$.

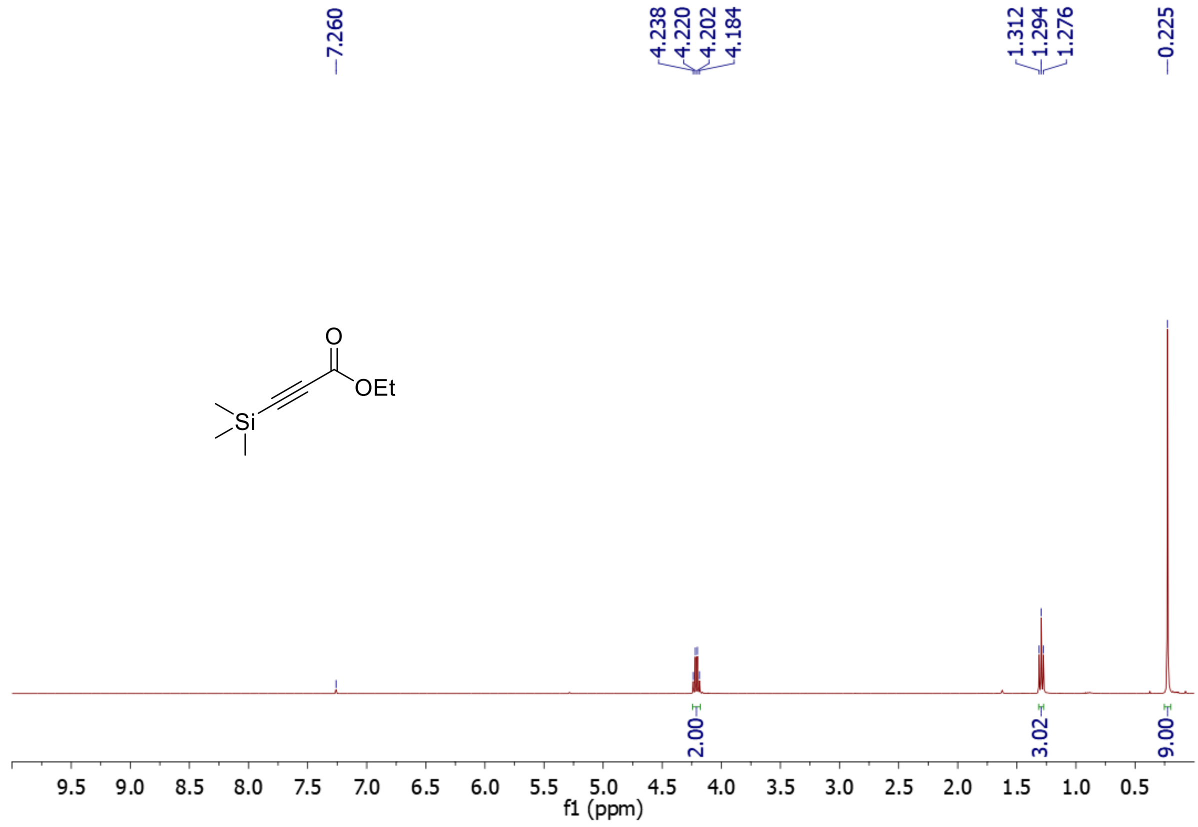

Fig. S154. ^{1}H NMR spectrum of ethyl 3-(trimethylsilyl)propiolate in $CDCl_3$.

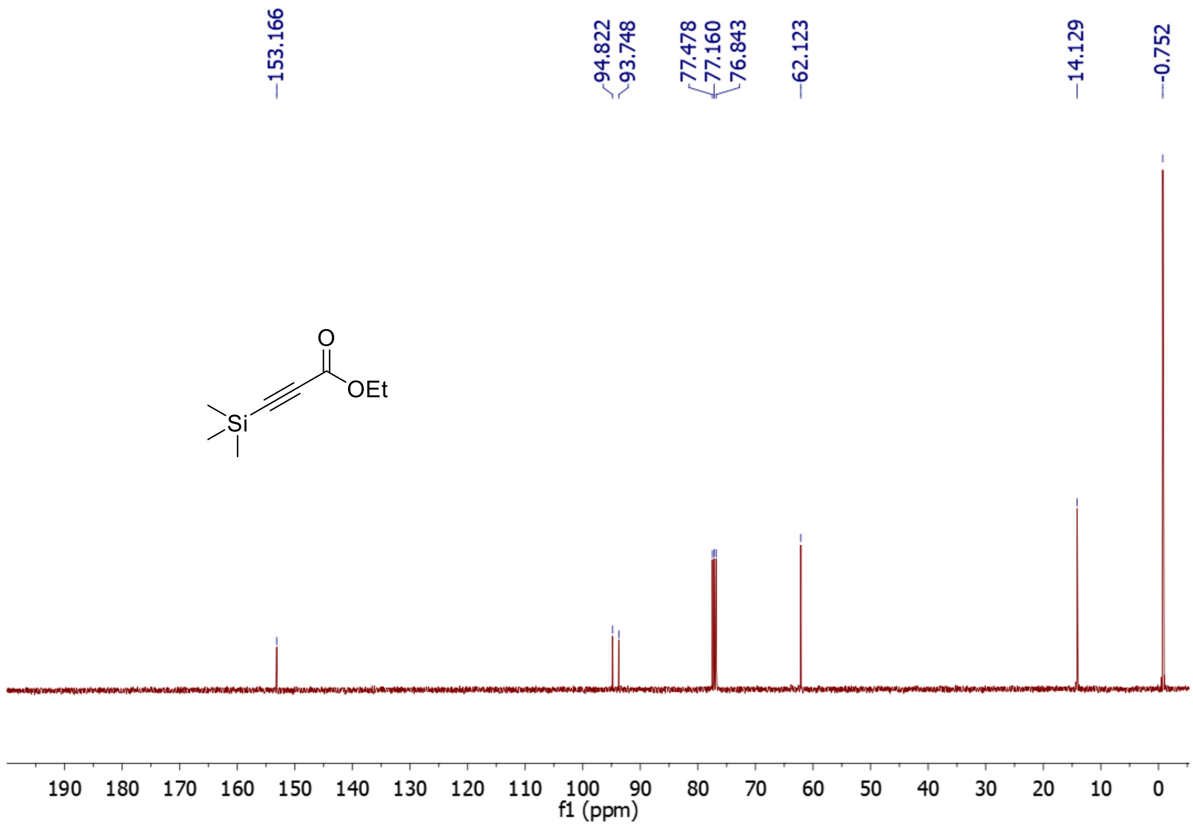

Fig. S155. ^{13}C NMR spectrum of ethyl 3-(trimethylsilyl)propiolate in $CDCl_3$.

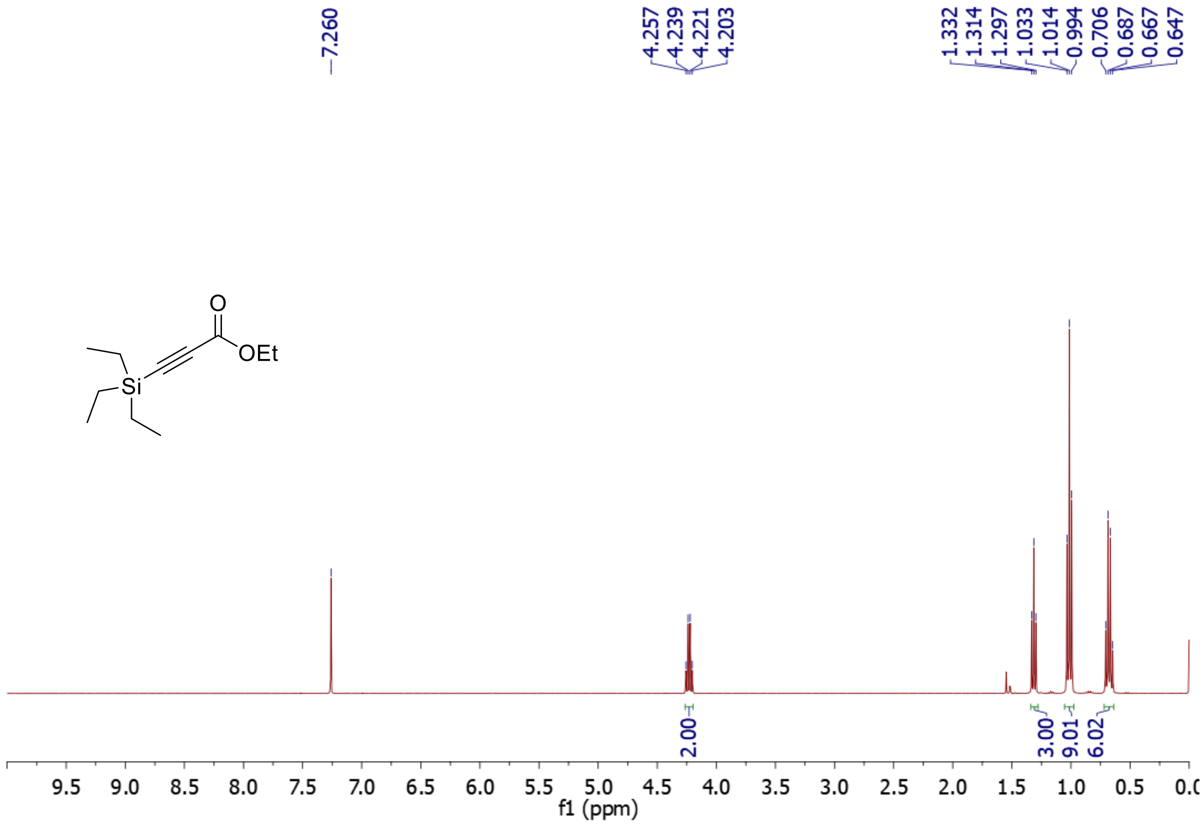

Fig. S156. ^{1}H NMR spectrum of ethyl 3-(triethylsilyl)propiolate in $CDCl_3$.

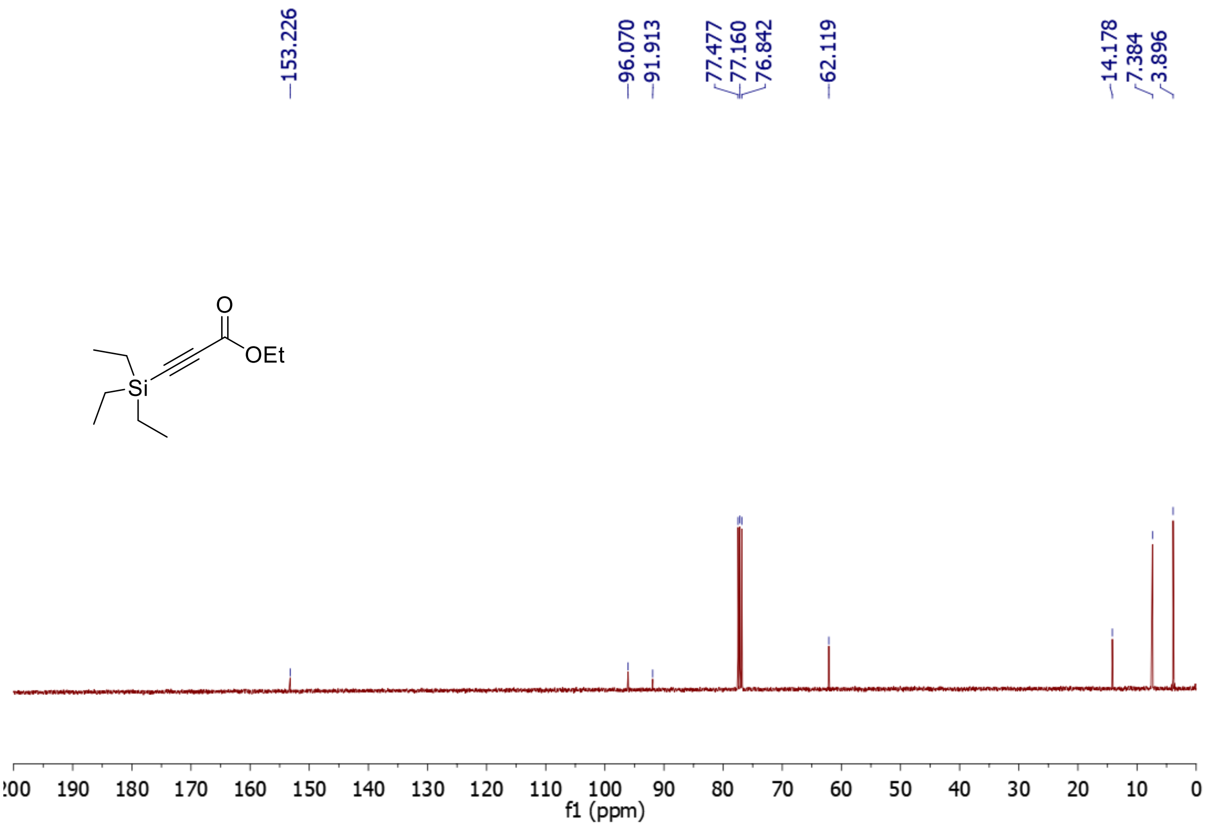

Fig. S157. ^{13}C NMR spectrum of ethyl 3-(triethylsilyl)propiolate in $CDCl_3$.

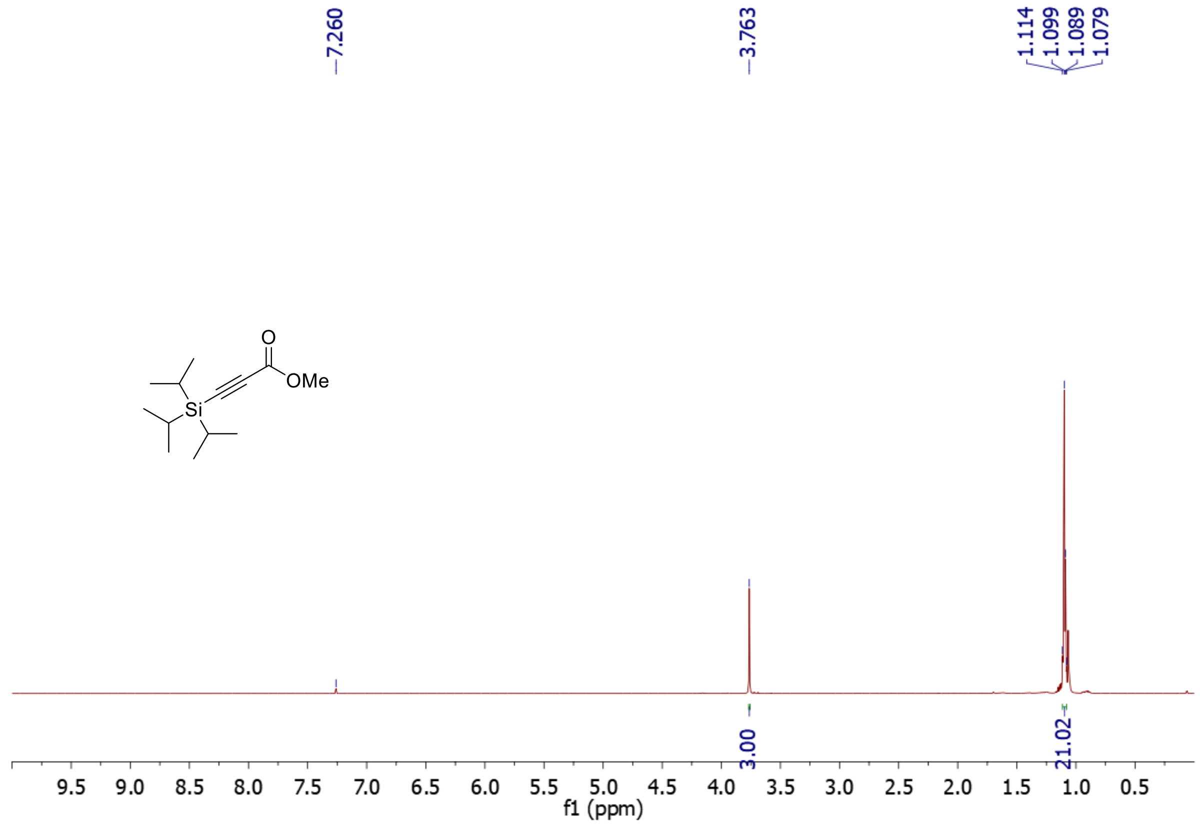

Fig. S158. ^{13}C NMR spectrum of methyl 3-(triisopropylsilyl)propiolate in $CDCl_3$.

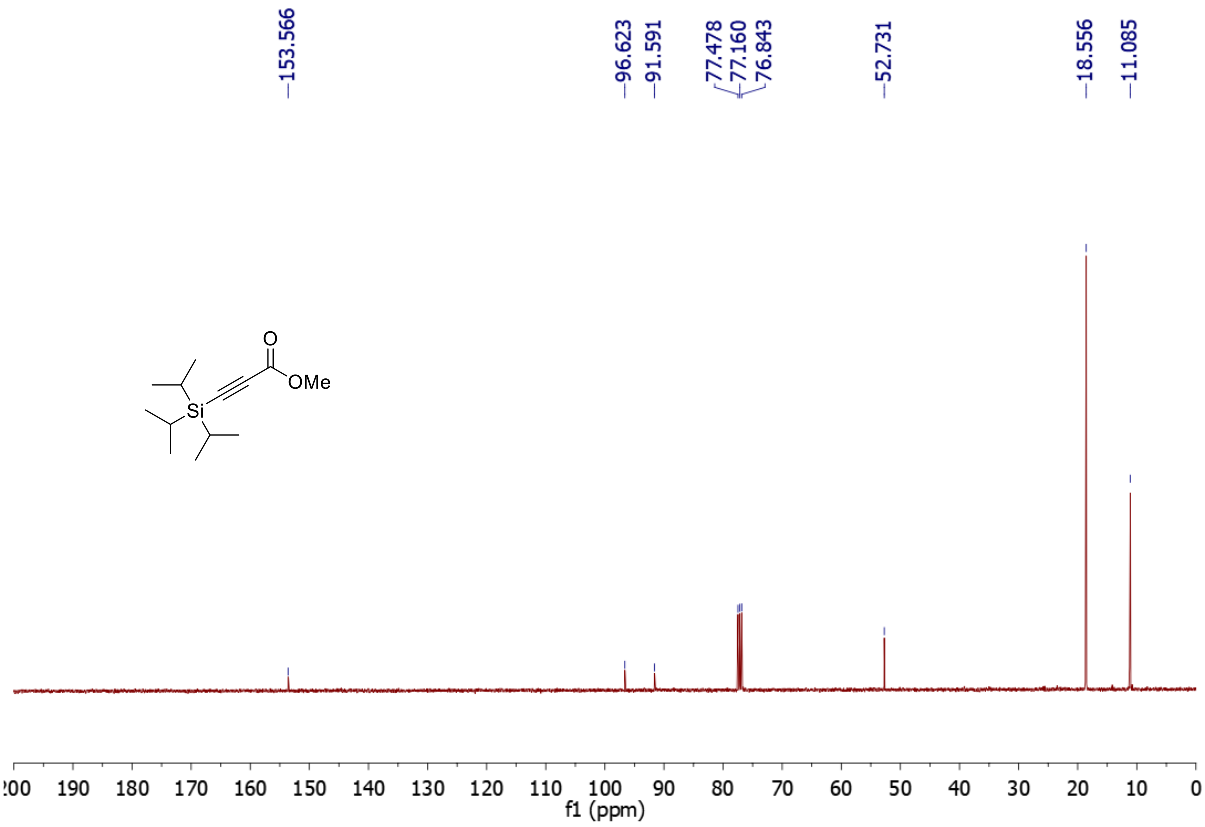

Fig. S159. ^{1}H NMR spectrum of methyl 3-(triisopropylsilyl)propiolate in $CDCl_3$.

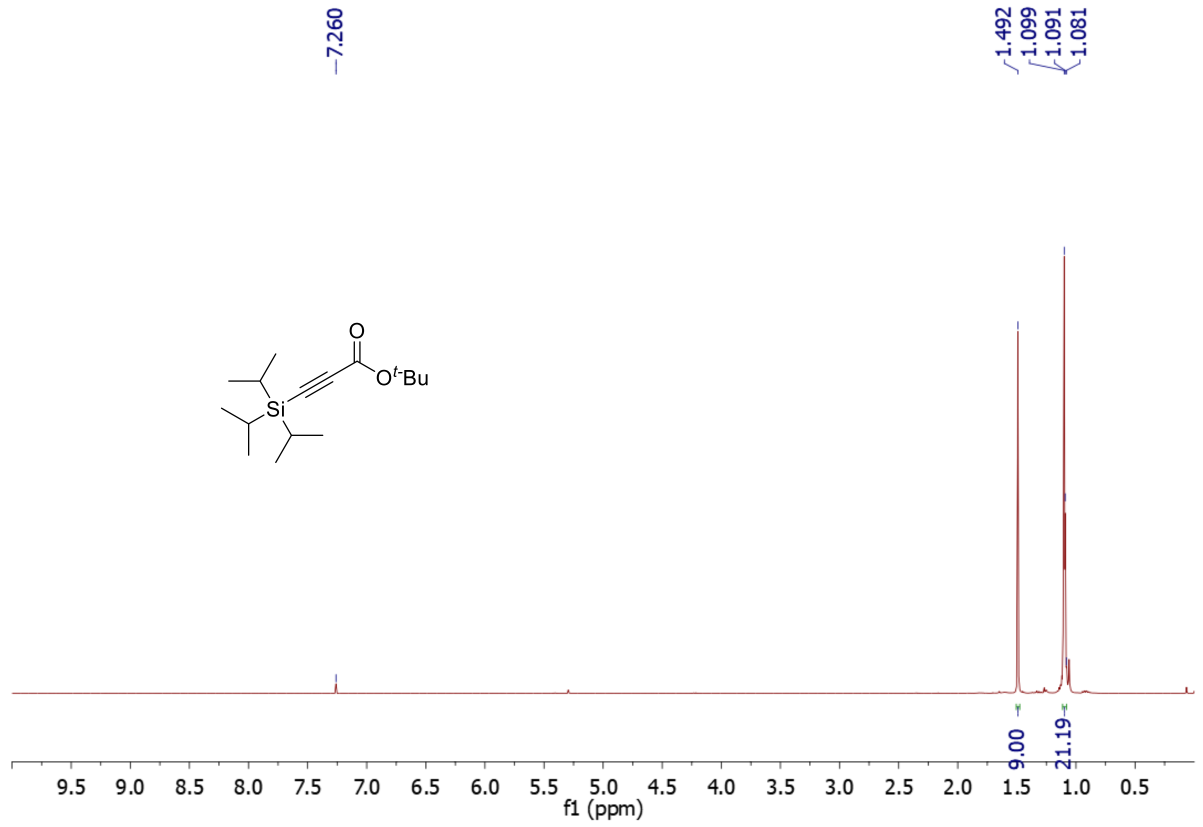

Fig. S160. ^{1}H NMR spectrum of tert-butyl 3-(triisopropylsilyl)propiolate in $CDCl_3$.

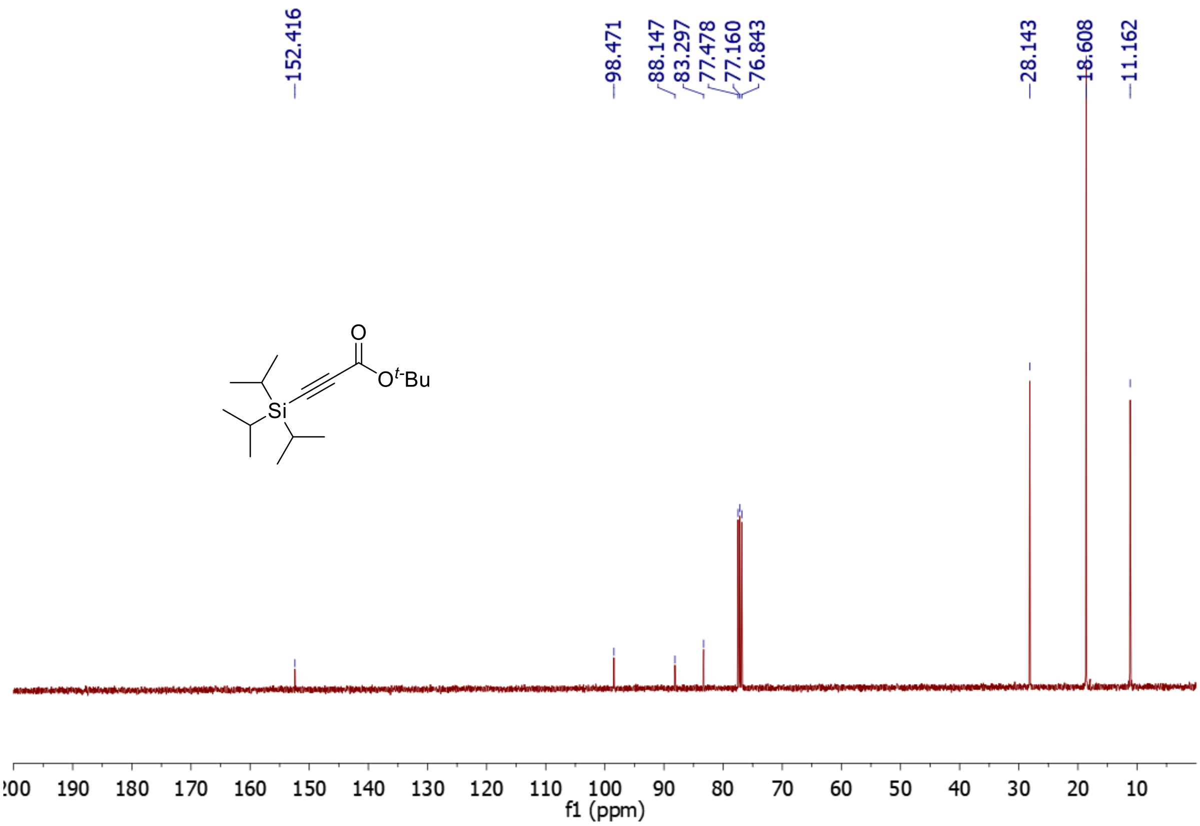

Fig. S161. ^{13}C NMR spectrum of tert-butyl 3-(triisopropylsilyl)propiolate in $CDCl_3$.

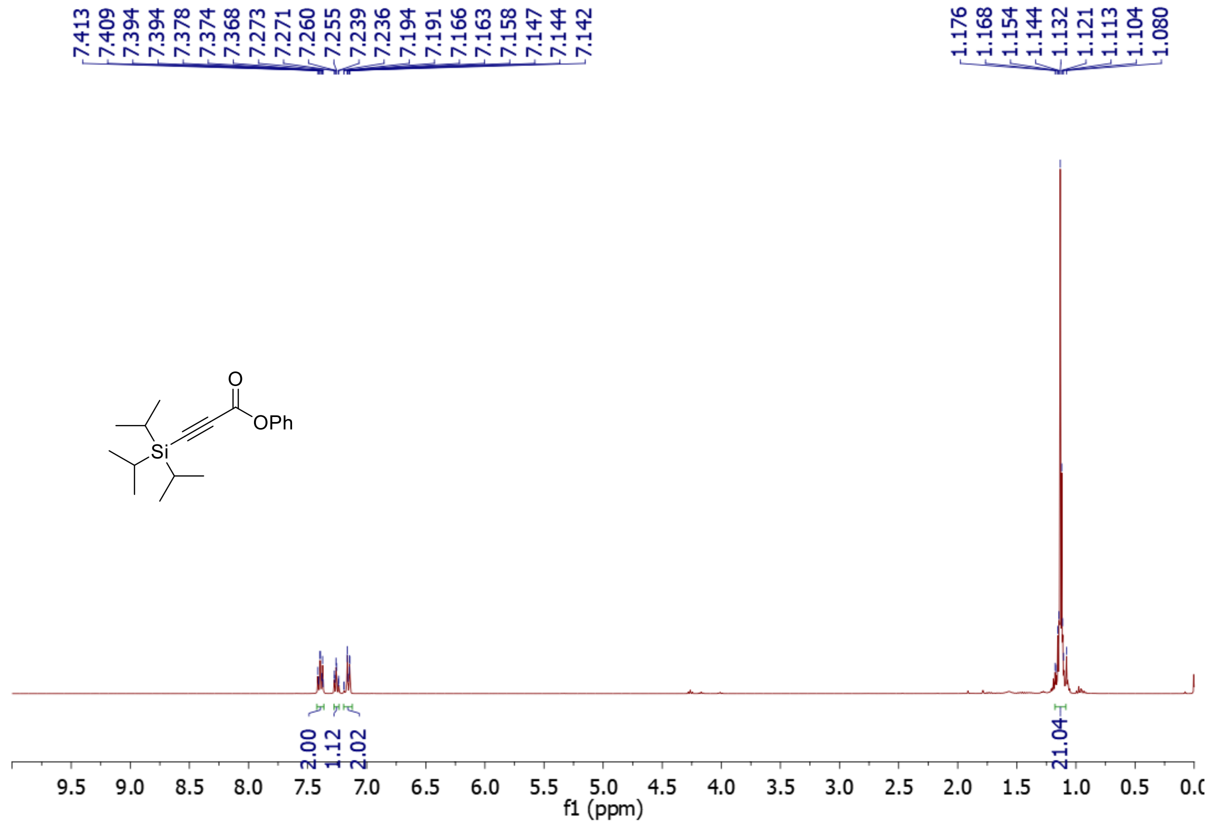

Fig. S162. ^{1}H NMR spectrum of phenyl 3-(triisopropylsilyl)propiolate in $CDCl_3$.

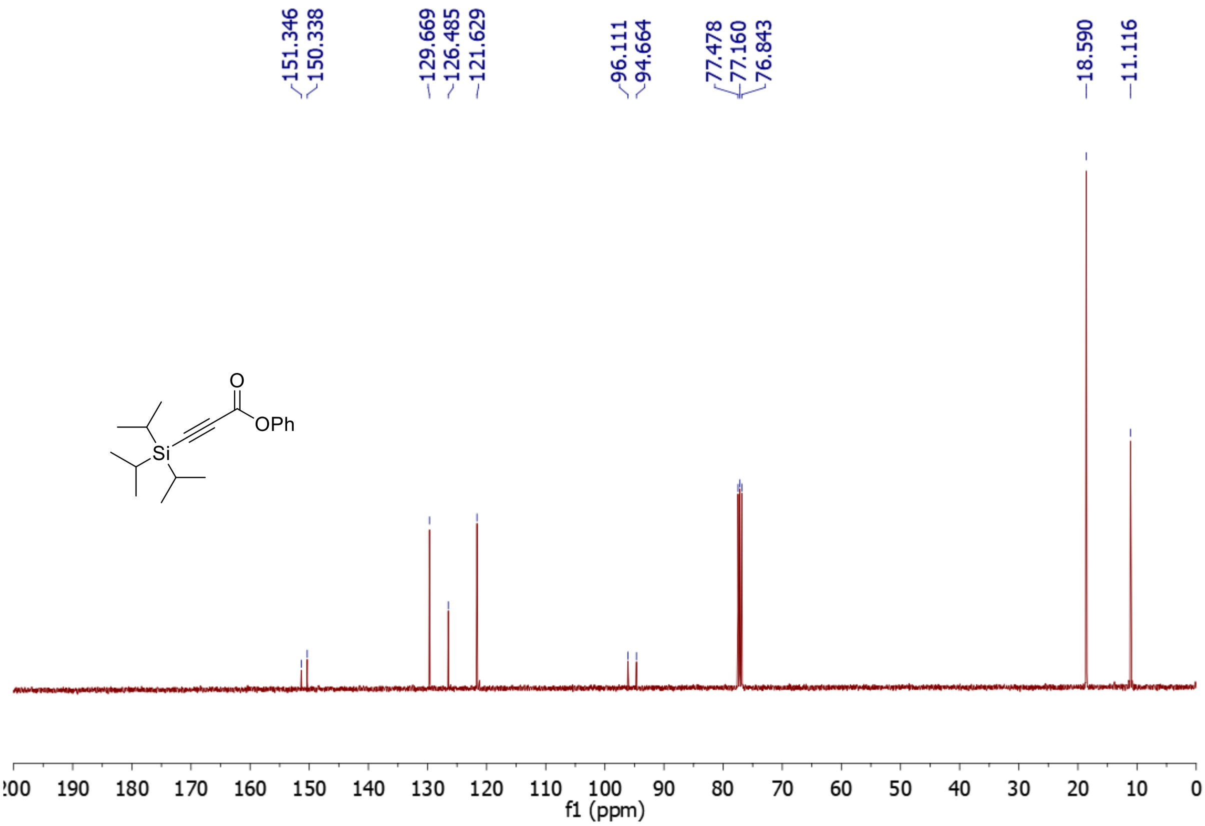

Fig. S163. ^{13}C NMR spectrum of phenyl 3-(triisopropylsilyl)propiolate in $CDCl_3$.

Data S1: Single crystal XRD data (Cif file) for **3b** compound.
